# Global Signaling Profiling in a Human Model of Tumorigenic Progression Indicates a Role for Alternative RNA Splicing in Cellular Reprogramming

**DOI:** 10.3390/ijms19102847

**Published:** 2018-09-20

**Authors:** Joseph A. Caruso, Nicholas J. Carruthers, Bryan Thibodeau, Timothy J. Geddes, Alan A. Dombkowski, Paul M. Stemmer

**Affiliations:** 1 Institute of Environmental Health Sciences, Wayne State University, Detroit, MI 48201, USA; aj7682@wayne.edu (N.J.C.); pmstemmer@wayne.edu (P.M.S.); 2 Beaumont BioBank and Molecular Core Laboratory, Royal Oak, MI 48073, USA; thibodeau.bryan@gmail.com (B.T.); timothy.geddes@beaumont.org (T.J.G.); 3 Department of Pediatrics, Wayne State University, Detroit, MI 48201, USA; adombkow@med.wayne.edu

**Keywords:** breast cancer, phosphoproteomics, alternative splicing, MCF10A

## Abstract

Intracellular signaling is controlled to a large extent by the phosphorylation status of proteins. To determine how human breast cells can be reprogrammed during tumorigenic progression, we profiled cell lines in the MCF10A lineage by phosphoproteomic analyses. A large cluster of proteins involved in RNA splicing were hypophosphorylated as cells progressed to a hyperplastic state, and then hyperphosphorylated after progression to a fully metastatic phenotype. A comprehensive transcriptomic approach was used to determine whether alterations in splicing factor phosphorylation status would be reflected in changes in mRNA splicing. Results indicated that the degree of mRNA splicing trended with the degree of tumorigenicity of the 4 cell lines tested. That is, highly metastatic cell cultures had the greatest number of genes with splice variants, and these genes had greater fluctuations in expression intensities. Genes with high splicing indices were mapped against gene ontology terms to determine whether they have known roles in cancer. This group showed highly significant associations for angiogenesis, cytokine-mediated signaling, cell migration, programmed cell death and epithelial cell differentiation. In summary, data from global profiling of a human model of breast cancer development suggest that therapeutics should be developed which target signaling pathways that regulate RNA splicing.

## 1. Introduction

Phosphorylation is the most common post-translational modification and plays a crucial role in regulating protein function [1]. Enzyme activity, protein:protein interaction, subcellular localization and protein stability are among the outcomes regulated by phosphorylation. These functions are controlled by the dynamic equilibrium between phosphorylation by kinases and dephosphorylation by phosphatases. Mass spectrometry after phosphopeptide enrichment yields a global snapshot of the phosphoproteome and gives insight into the status of signaling pathways of cells under varying conditions. To date, over 86,000 phosphosites have been identified in about 10,500 proteins, or about half the human proteome [2]. Thus, most phosphoproteins contain multiple phosphosites. We and others have shown that individual phosphosites can change in response to external stimuli independently and can control different functions within a protein [3,4]. Furthermore, phosphosites can occur in clustered regions within a protein, and different sites can have either stimulatory or inhibitory effects on function.

To determine how cell signaling becomes rewired during malignant transformation of human breast cells, we analyzed the MCF10A cell lineage (reviewed in Table S1). MCF10A cells (10A) are a spontaneously immortalized non-tumorigenic epithelial cell line obtained from a woman with fibrocystic breast disease [5]. This line displays characteristics of luminal ductal cells by electron microscopy but not myoepithelial cells. MCF10AT cells (AT) are a T24-*HRAS*-transformed derivate of 10A [6]. When implanted subcutaneously into nude mice, some of the animals develop a heterogeneous spectrum of histological changes which range from mild to moderate hyperplasia, to atypical ductal hyperplasia, to carcinomas in about 25% of animals. MCF10ATG3B cells (TG3B) were generated by serial transplantation of AT cells into nude/beige mice [7]. These premalignant cells progress to highly proliferative lesions (atypical hyperplasia, ductal carcinoma in situ, and invasive carcinoma) in greater than 50% of test animals. MCF10CA1a cells (CA1a) are also derived from AT cultures and were generated by serially growing a trocar transplantation [8]. This fully malignant cell line gives rise to rapidly growing tumors with 100% efficacy in nude mice.

Cellular models are a powerful tool for signaling research. Compared to clinical specimens, isogenic models have greatly reduced “noise” associated with individual and tumor genetic variability, and allow for multiple testing on a single cell type including treatment with drugs and/or inhibitors. Since malignant CA1a cultures are a derivative of non-tumorigenic 10A cells, researchers can directly probe the question of what biological features were gained or lost which have contributed to transformation. We have used the 10A lineage in the past to investigate alterations in signaling during tumorigenic progression via proteomic profiling of lipid rafts [9]. Results showed a relative decrease in G-protein and filamin A content in lipid rafts isolated from malignant cells, and increased levels of several intermediate filament proteins such as vimentin and keratins 5, 17 and 18.

In the current study, we have extended these findings by profiling phosphorylation alterations in the 10A lineage. Based on pathway analysis of the phosphoproteomic results we hypothesized that greater differences in mRNA splicing would be observed in malignant CA1a cultures relative to pre-malignant AT. To address this question we performed microarray analyses using a comprehensive probe set that quantifies relative mRNA expression at the exon level.

## 2. Results

To identify signaling pathways that may be associated with tumorigenic progression, phosphopeptides from 4 cell lines of the MCF10A lineage were enriched and analyzed by liquid chromatography/mass spectrometry (LC/MS). In total, 63,037 MS/MS phosphopeptide spectral identifications mapped to 1610 proteins, 58% of which had multiple phosphosite identifications. Neuroblast differentiation-associated protein AHNAK (AHNAK) had the highest number of individual phosphosite identifications with 36, followed by serine/arginine repetitive matrix protein 2 (SRRM2) with 35. In total, 4113 protein phosphosites were relatively quantified by spectral counting (Table S2). The first approach we used to identify significant pathways associated with tumorigenic progression in the MCF10A model was to group phosphoproteins with the greatest differences in spectral counts (Table 1). From these data it was determined that proteins associated with signaling, keratinization, lipid metabolism and mRNA splicing were highly represented.

**Figure 1:**
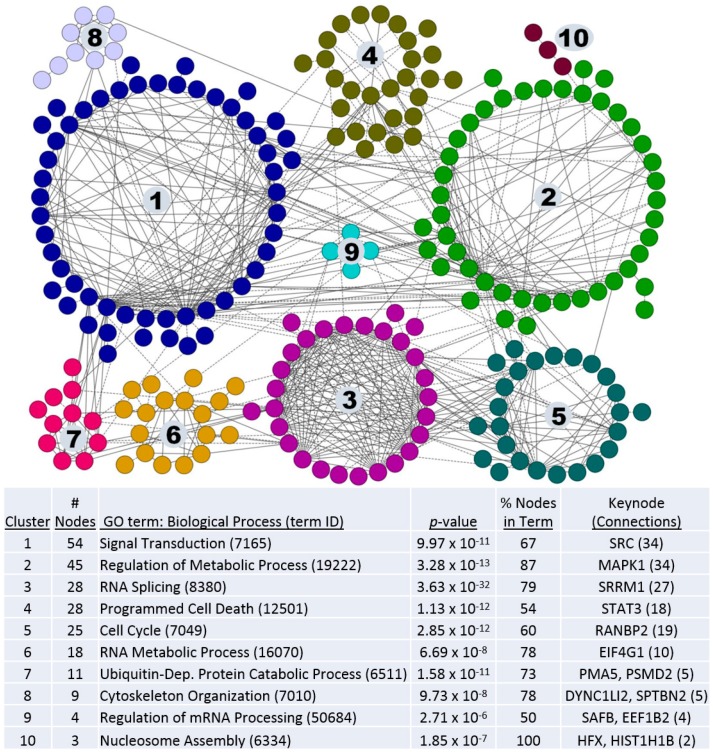
Pathway analysis of phosphoproteins involved in tumorigenic progression of the MCF10A lineage. Proteins with phosphosite differences of 3-fold or greater by spectral counting were analyzed by protein:protein interaction networking. Proteins are shown as nodes and interactions between proteins as edges. Protein names can be found in Table S3. Proteins are color-coded according to a network clustering algorithm. The most relevant gene ontology (GO) biological process terms for each cluster are listed, along with the keynode proteins (listed as gene names).

Our next approach was to analyze phosphorylation on a per site basis. Since AT cells were derived from 10A, and TG3B and CA1a were derived from AT, the 3 comparisons used in this study were AT versus 10A; TG3B versus AT; and CA1a versus AT. For these comparisons, phosphosites were included in the pathway analyses if there were ≥3-fold differences in spectral counts. 512 phosphoproteins that met this criterion were mapped to protein:protein interaction networks, and clusters with a minimum of 3 nodes were visualized (Figure 1). Proto-oncogene tyrosine-protein kinase Src (SRC) and mitogen-activated protein kinase 1 (MAPK1) signaling networks were the keynodes (i.e., most interconnected) proteins in the largest 2 clusters, which is consistent with the prominence of these pathways in cell and oncogenic signaling. The gene ontology (GO) biological process with the highest degree of significance was “RNA splicing” found in cluster 3 (*p* = 3.63 × 10^−32^). RNA processing proteins were also a major component of clusters 6 and 9. These data suggest that alternative splicing may have a key role in the transformation of MCF10A cells to a fully metastatic phenotype.

Most phosphoproteins are phosphorylated on multiple sites. For perspective on the overall phosphorylation status per protein, a phosphosite was scored as a −1 if there was a 3-fold decrease in spectral counts between cell line A and B, and a +1 if a 3-fold increase was observed. The phosphosite changes were then summed for each protein in order to determine how phosphorylation of clusters change with tumorigenic progression (Table 2). For the largest 3 clusters, results show that expression of oncogenic GTPase HRas (HRAS) in AT cells led to on overall hypophosphorylation compared to 10A, there was a slight reversal as AT cells transition to TG3B, whereas CA1a cells are highly hyperphosphorylated relative to AT. There are cluster-specific differences, but the overall trend suggests a net increase in global phosphorylation in malignant CA1a cells.

**Figure 2:**
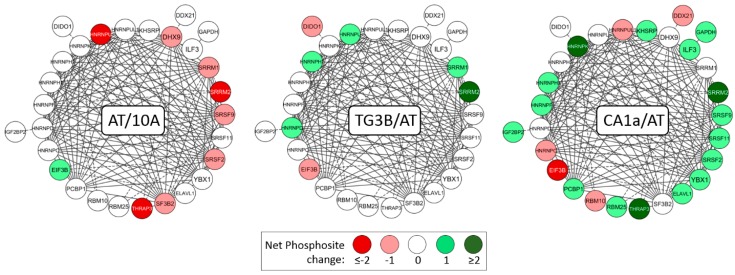
Phosphorylation status profiling of proteins involved in RNA splicing. Proteins (cluster #3 of Figure 1; labeled by gene name) were color-coded according to site-specific phosphorylation or dephosphorylation: nodes with net hypophosphorylation are colored red; nodes with no net change are white; and hyperphosphorylated nodes are in green. For example, SRRM2 protein in the CA1a/AT comparison had 6 phosphosites with ≥3-fold change in spectral counts, 4 of which increased in CA1a and 2 of which decreased for a net change of +2 and a dark green color-code.

Since RNA Splicing was the most significant GO association, the phosphorylation of cluster 3 was examined in more detail (Figure 2). Nodes were color-coded depending on their net change in phosphosite status, as either red (hypophosphorylated), white (no net change), or green (hyperphosphorylated). Most cluster 3 proteins had a net change and were hyperphosphorylated when comparing CA1a cells to AT, including serine and arginine rich splicing factors (SRSFs) 2, 9 & 11; heterogeneous nuclear ribonucleoproteins (HNRNPs) K, H1 & F; thyroid hormone receptor-associated protein 3 (THRAP3); and keynode SRRM2.

**Figure 3:**
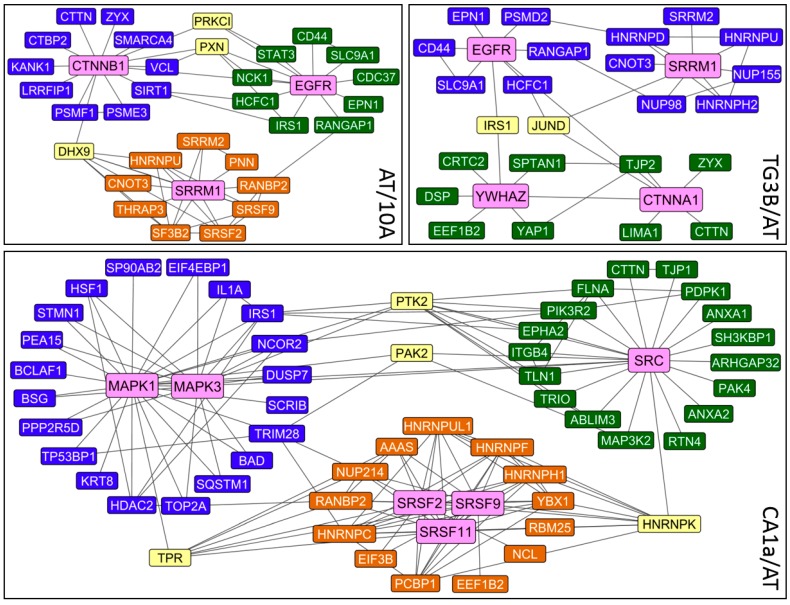
Keynode analysis of phosphoproteins with changes in phosphorylation status. Proteins with phosphosite differences of 3-fold or greater by spectral counting were mapped by protein:protein interaction networking. Keynodes (pink) were determined as proteins with the highest number of interactions among nodes with net phosphorylation status of ≤−1 or ≥+1. Primary keynodes have partners shaded in blue, secondary with partners shaded in green, and tertiary with partners shaded in orange. Proteins with connections to multiple keynodes are in yellow. Proteins are listed by gene name.

Keynodes are the most interconnected network proteins and are useful for systems analysis since they reduce the complexity of large networks and highlight important pathways. Keynode analysis in the 3 cell line comparisons independent of clustering is shown in Figure 3. β-Catenin (CTNNB1) and epidermal growth factor receptor (EGFR) are the primary keynodes in the AT/10A comparison, followed by the SRRM1 splicing factor. EGFR and SRRM1 are also found as keynodes for TG3B/AT, along with 14-3-3 protein zeta (YWHAZ) and α-catenin (CTNNA1) proteins. Note there are fewer overall connections for this comparison. In contrast, there is a large increase in the number of nodes and protein:protein interactions for the CA1a/AT comparison. MAPK1, MAPK2 and SRC [10] are the major keynodes, followed by splicing factors SRSF2, SRSF9 and SRSF11. These data show that splicing factors are altered at each stage of tumorigenic progression, and there is greater volume of phosphorylation changes as cells progress to a fully metastatic phenotype.

**Figure 4:**
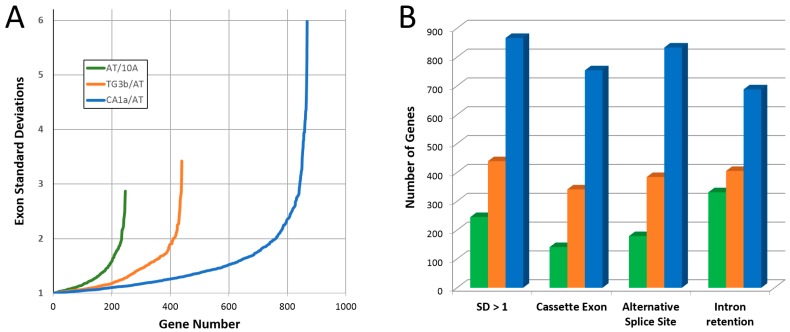
Estimate of mRNA splice variation between cell lines of the MCF10A lineage. (**A**) To calculate exon probe standard deviation (SD), microarray probe log2 intensities of cell line A were subtracted from cell line B for each exon within a gene (for coding genes with ≥3 exons). The SD was then calculated for each gene’s exon group as an estimate of mRNA splice variation. Genes with SD ≥ 1 are shown. (**B**) Comparison of exon SD ≥ 1 with alternate measures of alternative splicing: cassette exon (1 exon is spliced out along with its flanking introns); alternative splice site (an alternative 5′ donor or 3′ acceptor site is used); and intron retention.

Based on pathway analyses of phosphoproteomic data, we hypothesized that gene splicing would be altered during tumorigenic progression of 10A cells. To test for changes in splicing events, mRNA was extracted from the cell lines and profiled using microarray analysis. The human Clariom D assay uses over 540,000 probes to compare expression levels of genes (exons and splice junctions) as well as non-coding RNAs. To estimate RNA processing heterogeneity on a global basis, the standard deviation (SD) was calculated for exon probe expression levels between cell lines on a per gene basis (described in Figure S1). For the 3 comparisons, the vast majority of genes showed little exon probe variability as >95.8% of genes had probe SDs less than 1 (log2 scale, Table 3). However, the number of genes with exon SD ≥ 1 and the level of SD was much greater for the progression from AT→CA1a compared to either 10A→AT or AT→TG3B (Wilcoxon rank sum test *p* < 2.2 × 10^−16^, Figure 4A). The number of genes with exon SD ≥ 1 for each comparison were consistent with other measures of mRNA splice variation (Figure 4B). Taken together, these data suggest that CA1a cells have greater mRNA splice variation as predicted by signaling pathway analysis.

**Figure 5:**
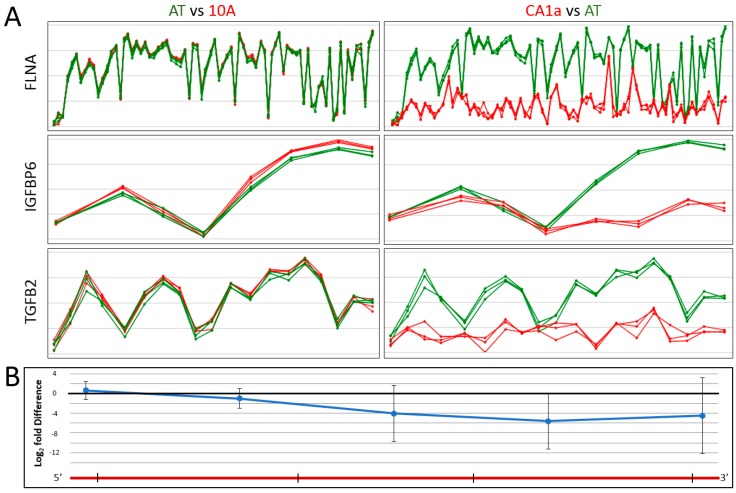
Tumorigenic progression of CA1a cells leads to specific patterns of mRNA splice variation. (**A**) Relative probe intensities for 3 representative genes: filamin A (*FLNA*), insulin-like growth factor binding protein 6 (*IGFBP*) and transforming growth factor beta 2 (*TGFB2*). Individual exon probes, shown as points along the gene, are ordered from 5′ of the mRNA on the left to 3′ on the right (*x*-axis). Probe intensities (log_2_ expression level) are on the *y*-axis. 3 independent replicates are shown for each cell line. Individual cell lines are either colored green or red as indicated in the figure. (**B**) Probe intensities were averaged for the top 10 genes with the highest exon SD values within the CA1a/AT comparison. The first point along the gene on the left represents the average probe intensities of the 5’-most probes, the last point on the right are the 3′-most probes, and the rest of the probes were grouped into thirds and averaged. Error bars represent standard deviation. Probe intensities’ distributions were different across the length of the gene (ANOVA, *p* = 0.0019).

There were specific trends observed when comparing exon probe intensities of genes with high SD in CA1a versus AT cultures. For example, the rate of gene transcription tended to be very consistent for the first exon or 5′-most probes, and then diverged by the midpoint of the mRNA (see Figure 5A for representative gene probe intensities and Figure 5B for the average of the top 10 genes with highest SD values). These data suggest that changes in phosphorylation of splicing factors may affect alternative splicing as well as the overall rate of transcription. 

**Figure 6:**
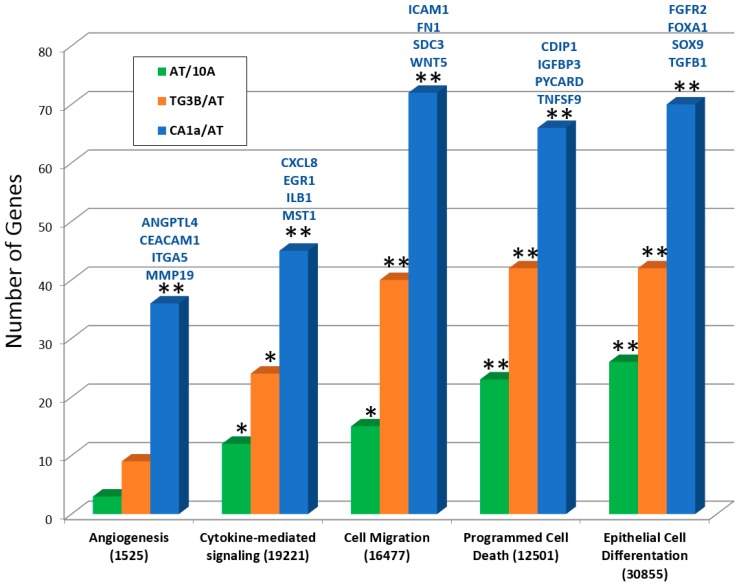
Gene ontology (GO) mapping of genes showing exon expression variation. For the three cell line comparisons, groups of genes with exon SD of ≥1 were compared against GO biological process groups for enrichment. Processes associated with tumorigenesis are indicated (GO search term numbers are in brackets). Genes above the column represent proteins found within the grouping that have been reported to be associated with breast cancer. *, *p* ≤ 0.05; **, *p* ≤ 0.001.

Our next query was to determine whether the genes with high degree of exon expression variability have known roles in tumorigenic progression and cancer. For this analysis, genes with exon SD of ≥1 were compared against GO biological process terms (Figure 6). Results showed highly significant correlations in CA1a/AT for tumorigenic processes such as angiogenesis, cytokine-mediated signaling, cell migration, apoptosis and epithelial cell differentiation. Specific proteins in this group which have known roles in breast cancer include fibroblast growth factor receptor 2 (FGFR2) [11], filamin A (FLNA) [12,13], forkhead box A1 (FOXA1) [14], integrin subunit α5 (ITGA5) [15], intercellular adhesion molecule 1 (ICAM1) [16], interleukin 1β (IL1B) [17], interleukin 8 (CXCL8) [18], protein tyrosine kinase 6 (PTK6) [19], transcription factor SOX-9 [20], transforming growth factor β1 (TGFB1) [21], and Wnt-5a [22]. Please note that all three comparisons have significant associations for epithelial cell differentiation, but only the malignant CA1a cells within the CA1a/AT comparison is significantly associated with angiogenesis. These results indicate that mRNA processing variation affects genes associated with breast cancer development in this model system.

**Figure 7:**
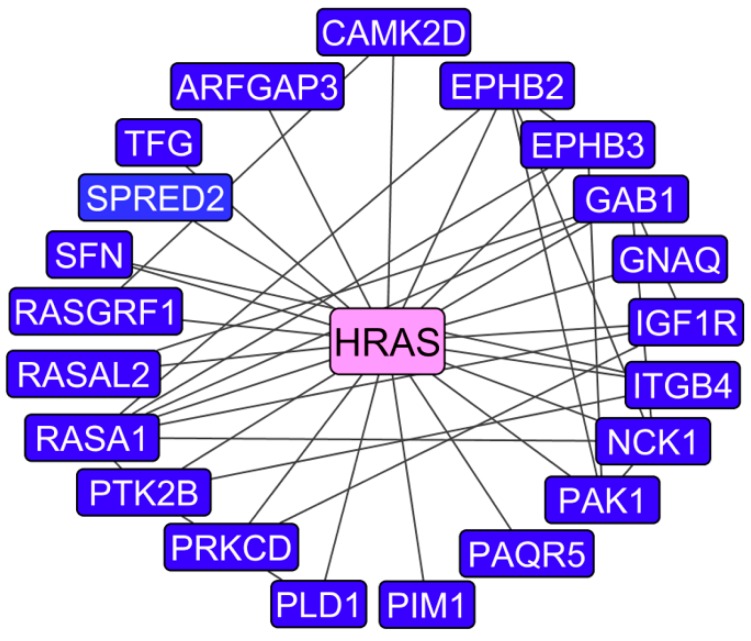
Keynode analysis for genes with high mRNA expression differences but little exon expression variation. For the CA1a/AT comparison, a gene was included in the grouping if (i) the fold difference in mRNA expression was at least 2-fold up or down, and (ii) the exon SD was between 0 and 1. Genes which met these thresholds were mapped to protein:protein interaction networks, and then the data was filtered to show the keynode (pink) and its putative interacting partners (blue).

Finally, we wanted to analyze the set of genes with low exon variation (SD < 1) and with high expression differences (≥2-fold). To get a perspective on the functionality of members of this group, genes were analyzed by keynode pathway analysis (Figure 7). Interestingly, *HRAS* was found to be the most interconnected gene/protein. As mentioned earlier, oncogenic *HRAS* was transfected into 10A cells to construct the AT cell line. It is not possible to determine the contributions of endogenous and oncogenic *HRAS* mRNAs with the Clariom D assay, but results showed a 2.7-fold increase in *HRAS* gene expression in the 10A→AT progression, and further 2.3- and 4.0-fold increases in *HRAS* in AT→TG32B and AT→CA1a, respectively. 

## 3. Discussion

In this study, a global systems approach was applied to determine cell signaling changes associated with tumorigenic progression in human breast cells. For this purpose phosphorylation profiling was performed in human model cell lines based on parental MCF10A. Deep proteomic analysis is exquisite in its ability to simultaneously identify thousands of unique proteins. However, relative levels of protein abundance span several orders of magnitude, and there is less confidence in quantitation of low abundance signaling molecules. This limitation can be mostly overcome in phosphoproteomic studies by (i) enrichment of phosphopeptides by selective chromatography; and (ii) use of pathway analysis to determine clusters of proteins that are altered in tandem. For example, if there is an uncertain level of confidence when looking for individual biomarkers of tumorigenic progression due to the stochastic nature of mass spectrometric peak selection for low abundance ions, we can be more certain of results when pathway analysis indicates that a group of proteins (either evolutionarily related, or in the same biochemical pathway, or as interacting partners) behave similarly.

In phoshoproteomic analysis of the 10A lineage, pathway analysis on proteins with differential phosphosite abundance showed 10 clustered regions with at least 3 nodes (Figure 1). Signaling networks within the largest two clusters, with SRC and MAPK1 as keynodes, have well documented roles in cancer but these signaling pathways control multiple intracellular processes including transcriptional regulation, proliferation, development, as well as tumor invasion and angiogenesis. Cluster 3 was of particular interest since most of the proteins had a role in a singular function: RNA splicing. This cluster also had the highest degree of interconnectedness based on protein:protein interaction mapping. Furthermore, alterations in RNA splicing factors had an early and persistent presence during tumorigenic progression, with changes observed in progression from 10A→AT, AT→TG3B, and most notably from AT→CA1a (Figure 3).

In recent years aberrant alternative RNA splicing has increasingly been recognized as a driver of cancer development, including breast cancer (reviewed in [23,24,25]). Phosphorylation and dephosphorylation of spliceosomal components regulates their subcellular distribution, protein:protein interactions, and activity [26]. Activation of cell signaling networks have been shown to affect alternative splicing of cancer-related genes. For example, hyperactivation of the RAC-alpha serine/threonine-protein kinase (AKT) pathway through Ras signaling can promote pro-survival splice variants of the KLF-6 and Caspase-9 genes [27,28]. The MAPK pathway, which is frequently hyperactivated in tumors, can enhance the invasiveness of tumor cells by modulating the alternative splicing of CD44 [29,30]. Specific kinases are known to phosphorylate splicing proteins, and upregulation of these proteins are often associated with cancer development. One such protein, SR-protein kinase 1 (SRPK1), is overexpressed in various human cancers including breast [31] and correlates with tumor progression and invasiveness [32]. Upregulation of SRPK1 has been shown to promote splice variants of the *MAP2K2* gene and enhance MAPK pathway signaling, and increase production of a pro-angiogenic splice variant of the *VEGFA* gene [33].

The 10A model of tumorigenic progression allows for predictions to be tested across different types of analyses, including proteomics and transcriptomics. Based on our preliminary results, we hypothesized that there would be a greater degree of alternative RNA splicing in CA1a cells compared to AT and TG3B cultures. Alternative splicing was investigated by microarray analysis using a chip designed for exon-level profiling, and results showed greater numbers of genes with spliced exon cassettes, alternative splice sites, and intron retention in the CA1a/AT comparison. Another measure we used to gauge RNA processing was to determine the variability in exon expression levels within a gene by determining the SD of the exon probe intensities between pairwise comparisons of cell lines. By this approach, results showed a greater number of genes with exon expression variability, and that these genes had a higher degree of SD, in the CA1a/AT comparison versus the AT/10A and TG3B/AT comparisons as would be predicted by their respective levels of tumorigenicity. To determine whether genes with high SD values had relevance to tumorigenic progression, we mapped these subgroups against GO terms known to be involved in cancer. These results showed highly significant associations for angiogenesis, cytokine-mediated signaling, cell migration, programmed cell death and epithelial cell differentiation for the CA1a/AT comparison.

The outcomes of alternative splicing can be varied, including changes in protein stability and expression level, alterations in protein function, and relocation to different subcellular areas [34]. We observed higher incidences of intron retention, alternative 5′ donor and 3′ acceptor sites, and elimination of exon cassettes with tumorigenic progression. However, we also observed very specific patterns in genes with the highest degrees of exon expression variability. Firstly, expression levels of the 5′-most probes tended to be similar for AT and CA1a cells, whereas downstream exons trended lower in CA1a. These data are consistent with previous reports showing that splicing is tightly coupled with transcription and mRNA stability [35,36,37]. Secondly, the mRNA expression level of genes with high splice variation (SD ≥ 2; Table 3) trended in synchronization with RNA Splicing cluster phosphorylation (Table 2). That is, overall hyperphosphorylation of splicing factors in CA1a versus AT led to a decrease in expression level of highly spliced mRNAs in CA1a (i.e., +14 sum of phosphosite changes = −137.1-fold change in gene expression), whereas hypophosphorylation had the opposite effect in AT versus 10A (−10 sum of phosphosite changes = 5.5-fold change in gene expression). Genes with high exon SD constitute a majority of the top genes with fold expression differences (Table 3), therefore qualitative differences in mRNA levels should be taken into careful consideration when making quantitative estimates. 

Previously, we investigated signaling in the 10A lineage by profiling protein abundance within lipid rafts [9]. That study showed that filamin A, an actin cross-linking protein and scaffold for over 90 binding partners, had 10-fold lower abundance in lipid rafts of CA1a cells compared to parental 10A, and this decrease was found to correlate with reduced total cellular levels. Here we show that the mechanism for decreased cellular content is likely the result of altered RNA processing of *FNLA* (Figure 5A). In comparing CA1a mRNAs to 10A, the first 3 probes of *FLNA* overlap in expression, which is followed by a steep drop in expression for subsequent probes, and a 572-fold decrease in relative CA1a mRNA expression overall. Multiple splicing events (alternative splice sites; cassette exon omissions; intron retentions) were observed for the CA1a/AT comparison. Down-regulation of filamin A in breast and lung cancer cell models has been shown to stimulate proliferation, migration, invasion and metastasis formation [38,39], and alternative splicing of filamin A has been reported to have a role in differentiation and organogenesis [13,40]. It is plausible that altered phosphorylation of splicing factors during tumorigenic progression in the 10A model leads to alternative splicing of genes such as *FLNA* that are associated with differentiation and metastasis, but further work is required to directly link splicing factors with specific gene products.

Microarray analyses in the 10A model showed that genes with high exon expression variation were associated with greater fold changes in expression level, and that many of these genes/proteins have known roles in cancer. We also posed that question of whether the genes with high fold change differences and low exon SD are also linked with tumorigenic progression. Keynode analysis on this subset showed a network of genes associated with HRAS signaling. HRAS encodes a protein involved in transducing growth and differentiation signals from the plasma membrane to intracellular signal cascades such as the MAPK and AKT pathways [41]. AT cells were transfected with a constitutively active *HRAS* gene, but these types of mutations are rare in human breast cancer, where specific genetic polymorphisms [42,43] and higher expression levels [44,45] are more prevalent. For the 10A lineage, in addition to an activating mutation, *HRAS* gene expression levels were found to continuously rise with tumorigenic progression which underscores the importance of this signaling protein in the context of this model. One of the limitations of this study was the use of a single in vitro model of human tumorigenic progression. Future studies will focus on other models that are not driven by HRAS for comparison of alternative splicing outcomes.

## 4. Materials and Methods

### 4.1. Cell Culture

Description of the MCF10A lineage (MCF10A, MCF10AT, MCF10ATG3B and MCF10CA1a) and cell culture conditions are described elsewhere [9], and a summary of phenotypes can be found in Table S1. Briefly, cells were cultured in Dulbecco’s Modified Eagle Medium/F-12 medium (DMEM/F-12, Invitrogen, Waltham, MA, USA) supplemented with 10 µg/mL of human insulin (Invitrogen), 20 ng/mL of epidermal growth factor (Invitrogen), 0.5 μg/mL of hydrocortisone (Sigma, St. Louis, MO, USA), 5% horse serum (Invitrogen), 100 U/mL of penicillin (Invitrogen), and 100 μg/mL of streptomycin (Invitrogen). Cells were maintained in a humidified environment of 5% CO_2_/95% air at 37 °C.

### 4.2. Phosphopeptide Enrichment

Cell cultures growing in exponential phase growth on 100 mm plates were washed 3× with ice cold HBSS buffer (Thermo Fisher, Waltham, MA, USA) and then lysed with 10 M urea (Invitrogen), 50 mM ammonium bicarbonate (J. T. Baker, Waltham, MA, USA), 0.5 mM EDTA (Gibco, Waltham, MA, USA), 1 mM NaF (Sigma), 1 mM sodium orthovanadate (Sigma) and 2 mM β-glycerophosphate (Sigma). Lysates were scraped and transferred to Eppendorf tubes, vortexed and pelleted. Protein concentrations of the supernatants were estimated by BCA assay (Thermo Fisher) and stored at −80 °C until further use. 1 mg of protein was reduced with 5 mM TCEP (Fluka, Waltham, MA, USA) in 0.025% ProteaseMax (Promega, Madison, WI, USA) for 30 min, followed by alkylation with 15 mM iodoacetamide (Sigma) for 30 min. Samples were digested overnight at 37 °C with 7.5 mg trypsin (Promega) in 10% acetonitrile (Sigma), 25 mM ammonium bicarbonate and 1 M urea. Peptides were desalted with a Sep-Pak Plus C18 Cartridge (Waters Corporation, Milford, MA, USA) and dried. Phosphopeptides were enriched using 4 mg of 5 μm Titansphere TiO_2_ beads (GL Sciences, Tokyo, Japan) in 65% acetonitrile, 2% trifluoroacetic acid (Thermo Fisher) with glutamic acid saturation. Phosphopeptides were washed, eluted from the beads and dried for long term storage.

### 4.3. LC/MS/MS

For each sample, 10% of peptides were separated by reverse phase C18 chromatography and analyzed on a LTQ-XL mass spectrometer (Thermo Fisher) over a 70 min gradient. MS1 was performed in enhanced mode over 400–1800 *m*/*z*. MS2 included fragmentation of the top 7 ions with CID (35% collision energy; 0.25 activation Q). MS3 was triggered if a neutral loss of phosphoric acid (98 Da) was detected in the top 3 fragment ions. 3 technical replicates were run for each biological triplicate.

### 4.4. Data Analysis

Peak lists were generated with Proteome Discoverer (Thermo; v1.3). MS2 and MS3 spectra were scored with Mascot (Matrix Science; v2.3) against a human protein database from Uniprot (20,323 sequences). Search criteria included up to 1 missed tryptic cleavage, dynamic modifications of oxidation (M), phosphorylation (S,T,Y), acetylation (protein N-terminal); and a static modification of cysteine (carbamidomethylation). Data was imported in Scaffold (Proteome Software; v3.3), filtered to 1.0% peptide FDR, and then imported into ScaffoldPTM (v1.1) for organization of phosphosites by location and site probability. Relative quantitation was performed by spectral counting, and counts were summed for each of the 9 runs per cell line. Pathway analysis was performed by processing filtered data through the Reactome FI plugin for Cytoscape (v3.5) which included a clustering algorithm, and through enrichment analysis against GO Biological Process terms (available online: www.geneontology.org). Significance of GO search terms were calculated as *p*-values using the Fisher’s exact test with FDR multiple test correction. The proteomics datasets generated and/or analyzed during the current study are available in the Zenodo repository, http://doi.org/10.5281/zenodo.1308091.

### 4.5. Alternative Splicing and Relative Gene Expression

Cell cultures growing in exponential growth phase on 100 mm plates were washed 3× with ice-cold HBSS, excess liquid removed, and total RNA was extracted using Trizol reagent (Invitrogen). RNA splicing was assessed using the Clariom D Human assay (Applied Biosystems, Foster City, CA, USA) using biological triplicate samples for each cell line. Data was analyzed using the Transcriptome Analysis Console (Thermo Fisher; v4.01). Variations in global mRNA splicing were gauged, per gene, by subtracting the average log2 exon probe expression level of cell line 1 versus cell line 2, and then determining the standard deviation. This was performed for coding genes with at least 3 exon probes only, and junction probes were excluded. This is explained more clearly in Figure S1. Comparative relative fold differences in mRNA expression levels and exon events (i.e., alternative splice sites, exon cassettes and intron retention) were computed by the Transcriptome Analysis Console (Applied Biosystems).

## 5. Conclusions

In summary, a systems approach identified RNA splicing as a significant intracellular signaling pathway that is altered during tumorigenic progression of human breast cell cultures. Changes in alternative mRNA splicing was validated using a transcriptomic approach which showed a large increase in splicing changes when cells progressed from hyperplastic to metastatic in the context of this model system. Splicing factor phosphorylation trended in synchronization with overall gene splicing and mRNA expression levels of alternatively spliced genes. The MCF10A lineage and similar human breast cancer models could be used to test novel cancer therapeutics which target reprogramming of splicing factor networks [46,47,48]. 

## Figures and Tables

**Table 1:** Spectral count profiling of phosphoproteins in the MCF10A lineage.

Protein Name	Uniprot Accession Number	10A	AT	TG3B	CA1a	Grouping
Chromodomain-helicase-DNA-binding protein 3*	CHD3_HUMAN	13	2	8	0	Chromatin
Histone H1.3	H13_HUMAN	18	8	10	0	Chromatin
Histone H1.5	H15_HUMAN	46	55	49	0	Chromatin
Epidermal growth factor receptor	EGFR_HUMAN	0	12	1	13	EGFR signaling
Integrin beta-4	ITB4_HUMAN	0	0	0	19	EGFR signaling
SH3 domain-containing kinase-binding protein 1	SH3K1_HUMAN	0	5	1	11	EGFR signaling
Ras-assoc. & pleckstrin homology domains-containing prot. 1	RAPH1_HUMAN	0	2	0	10	EGFR signaling
Phosphatidylinositol 3-kinase regulatory subunit beta	P85B_HUMAN	0	0	0	9	EGFR signaling
Mitogen-activated protein kinase 3	MK03_HUMAN	0	0	2	8	EGFR signaling
Protein diaphanous homolog 1	DIAP1_HUMAN	0	2	3	8	EGFR signaling
Epiplakin	EPIPL_HUMAN	1	7	6	27	keratinization
Keratin, type II cytoskeletal 6A	K2C6A_HUMAN	4	19	7	81	keratinization
Keratin, type II cytoskeletal 8	K2C8_HUMAN	0	0	1	52	keratinization
Keratin, type I cytoskeletal 15	K1C15_HUMAN	0	2	20	129	keratinization
Keratin, type I cytoskeletal 17	K1C17_HUMAN	0	11	0	35	keratinization
Acetyl-coenzyme A synthetase, cytoplasmic	ACSA_HUMAN	3	6	2	92	lipid metabolism
ATP-citrate synthase	ACLY_HUMAN	0	0	0	60	lipid metabolism
Fatty acid synthase	FAS_HUMAN	10	6	9	87	lipid metabolism
Hydroxymethylglutaryl-CoA synthase, cytoplasmic	HMCS1_HUMAN	41	51	31	290	lipid metabolism
E3 ubiquitin-protein ligase rififylin	RFFL_HUMAN	2	3	2	21	protein degradation
LIM domain only protein 7	LMO7_HUMAN	1	0	0	20	protein degradation
Proteasome subunit alpha type-5	PSA5_HUMAN	0	0	0	13	protein degradation
C-Jun-amino-terminal kinase-interacting protein 4	JIP4_HUMAN	2	2	0	15	signaling
Ephrin type-A receptor 2	EPHA2_HUMAN	0	1	1	33	signaling
Interleukin-1 alpha	IL1A_HUMAN	0	2	0	13	signaling
Major vault protein	MVP_HUMAN	0	0	2	34	signaling
PDZ and LIM domain protein 4	PDLI4_HUMAN	66	39	50	0	signaling
Proline-rich AKT1 substrate 1	AKTS1_HUMAN	12	0	0	0	signaling
Protein phosphatase 1 regulatory subunit 1B	PPR1B_HUMAN	0	0	0	18	signaling
Protein phosphatase 1 regulatory subunit 14B	PP14B_HUMAN	0	3	1	10	signaling
Rho GTPase-activating protein 29	RHG29_HUMAN	0	0	2	83	signaling
Rho GTPase-activating protein 32	RHG32_HUMAN	0	0	1	12	signaling
ADP-ribosylation factor-like protein 6-interacting protein 4	AR6P4_HUMAN	12	2	5	0	mRNA splicing
BUD13 homolog	BUD13_HUMAN	1	0	0	8	mRNA splicing
Heterogeneous nuclear ribonucleoproteins C1/C2	HNRPC_HUMAN	10	4	7	0	mRNA splicing
Protein PRRC2A	PRC2A_HUMAN	0	4	2	12	mRNA splicing
Putative RNA-binding protein 15	RBM15_HUMAN	1	1	0	9	mRNA splicing
Serine/arginine-rich splicing factor 11	SRS11_HUMAN	0	1	1	18	mRNA splicing
YTH domain-containing protein 1	YTDC1_HUMAN	1	0	0	8	mRNA splicing

* Proteins with increased and decreased phosphorylation during tumorigenic progression are shown in black and red fonts, respectively.

**Table 2:** Phosphorylation profiling of protein:protein interaction clusters.

Cluster	GO Term: Biological Process	10A→AT			AT→TG3B			AT→CA1a		
		a	b	c	a	b	c	a	b	c
1	Signal Transduction	−9	−0.17	51.9	2	0.04	38.9	45	0.83	75.9
2	Regulation of Metabolic Process	−7	−0.16	44.4	2	0.04	37.8	18	0.40	68.9
3	RNA Splicing	−10	−0.36	35.7	4	0.14	25.0	14	0.50	75.0
4	Programmed Cell Death	9	0.32	42.9	−12	−0.43	50.0	13	0.46	75.0
5	Cell Cycle	−9	−0.36	52.0	−2	−0.08	48.0	8	0.32	72.0
6	RNA Metabolic Process	−2	−0.12	55.6	−2	−0.12	33.3	2	0.11	61.1
7	Ubiquitin-Dep. Protein Catabolic Process	1	0.09	36.4	−1	−0.09	27.3	11	1.00	81.8
8	Cytoskeleton Organization	2	0.22	44.4	−4	−0.44	66.7	3	0.33	66.7
9	Regulation of mRNA Processing	−2	−0.50	50.0	−1	−0.33	25.0	−1	−0.25	50.0
10	Nucleosome Assembly	0	0	0	1	0.33	33.3	0	0	66.7

a = Sum of phosphosite changes; b = Phosphosite changes per node; c = % of nodes in cluster with phosphosite changes.

**Table 3:** Pairwise comparison of mRNA expression changes for different levels of alternative splicing in the 10A model of tumorigenic progression.

Exon SD Range	Average Fold Gene Expression (% of Total)		
	AT/10A	TG3B/AT	CA1a/AT
0–1	0.2 ± 1.3 (98.81)	0.1 ± 1.4 (97.88)	0.2 ± 1.5 (95.83)
1–2	1.8 ± 5.0 (1.13)	−2.6 ± 8.1 (1.98)	0.1 ± 8.2 (3.65)
≥2	5.5 ± 19.6 (0.06)	−11.7 ± 15.7 (0.14)	−137.1 ± 993.6 (0.53)
**Top 100 Genes with Highest Expression Changes**			
Proportion of Genes with Exon SD ≥ 1	67% *	87% *	98% *
Average Fold Gene Expression (±SD)	5.9 ± 13.2	−13.0 ± 17.1	−149.9 ± 1037.1

* Significant enrichment of genes with SD > 1, *p* < 2.2 × 10^−16^.

## Supplementary Materials

Supplementary materials can be found at http://www.mdpi.com/1422-0067/19/10/2847/s1.
