# Supplementary material for: Global Signaling Profiling in a Human Model of Tumorigenic Progression Indicates a Role for Alternative RNA Splicing in Cellular Reprogramming
Source: Int J Mol Sci. 2018 Sep 20;19(10):2847. doi: 10.3390/ijms19102847 (PMC6213538; doi:10.3390/ijms19102847)
Supplement: Supplementary file 1 [file ijms-19-02847-s001.pdf]

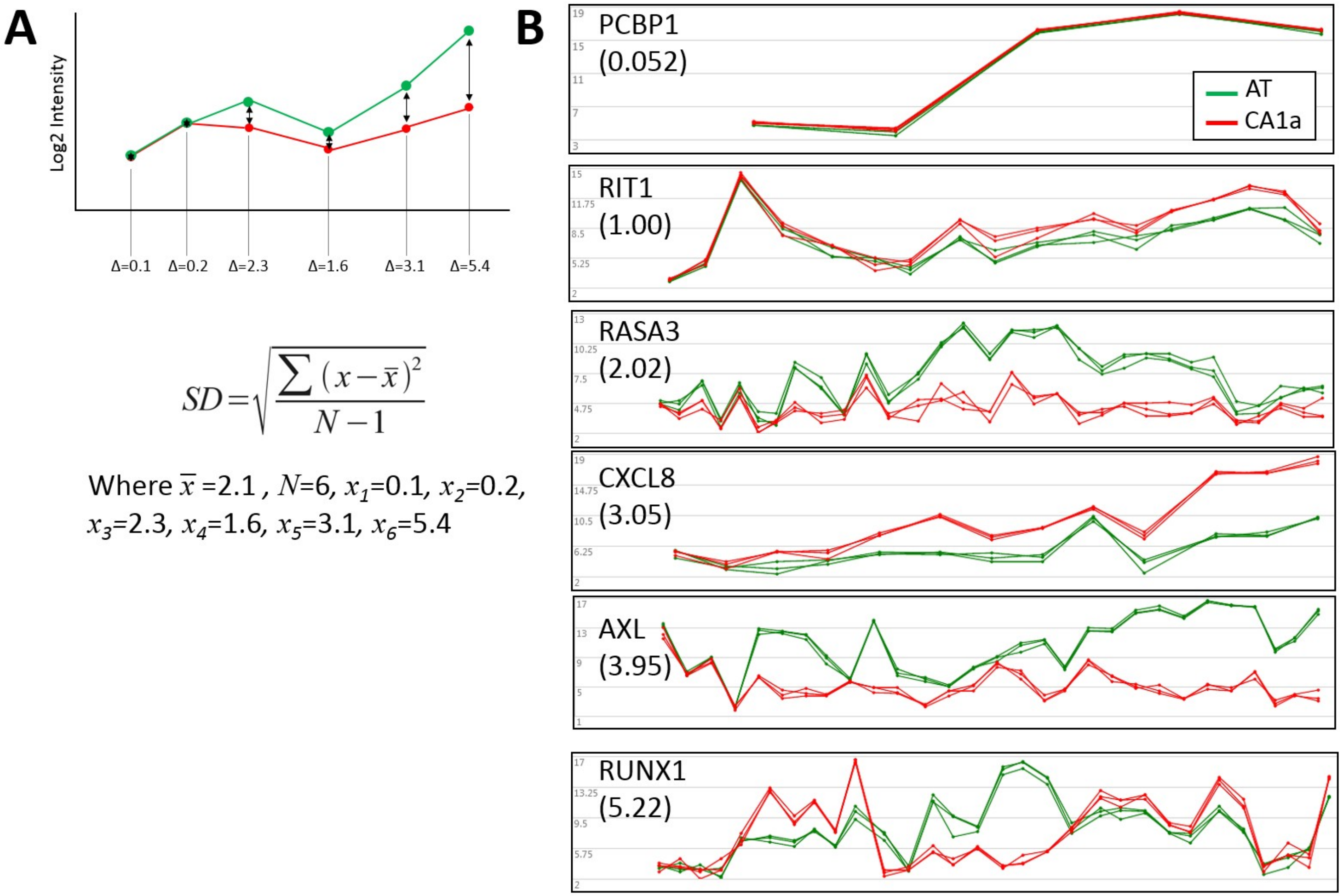

Figure S1. Calculation of exon probe standard deviation (SD) per gene as an estimate of alternative splicing. (**A**) To calculate SD, log2 exon probe intensities were averaged for 3 independent biological replicates per cell line. Differences in probe intensities in Cell line A versus Cell line B were used for SD calculations. Coding genes had to have a minimum of 3 exon probes to be included. (**B**) Representative exon probe traces for 3 replicates of AT (green) and CA1a (red). Genes are represented from the 5′ on the left to 3′ on the right. Gene names are shown along with calculated SD values in brackets.

Table S1. Phenotypic summary of the MCF10A series of human mammary epithelial cell lines with increasing tumorigenic progression.

**Cell Line:**	**MCF10A**	**MCF10AT**	**MCF10TG3B**	**MCF10CA1a**
**Description:**	quasi-normal epithelial cell line derived from a woman with fibrocystic breast disease	MCF10A transfected with T24 HRAS oncogene vector	MCF10AT serially xenografted 3X in nude mice	MCF10AT serially xenografted 4X in nude mice
**Highly Proliferative Tumors:**	not detected	about 25% of animals	>50% of animals	fully metastatic in 100% of animals
**Tumor Morphology:**	not detected	forms simple ducts; mild to moderate hyperplasia	atypical hyperplasia and ductal carcinoma in situ (DCIS) phenotypes	undifferentiated to well-differentiated adenocarcinoma phenotypes
**Degree of Tumorigenicity:**	none	low	middle	high

Table S2. Phosphosite identification and relative quantitation. Phosphopeptides were enriched from 3 biological replicates from 10A, AT, TG3B and CA1a cell lines and analyzed by LC/MS. Data shows summed spectral counts from 3 technical replicates per sample.

#	Uniprot Protein Accession	Protein Name	Site	Best Ascore	Localization Probability	Spectral Counts per Site	10A-1	10A-2	10A-3	AT-1	AT-2	AT-3	TG3B-1	TG3B-2	TG3B-3	CA1a-1	CA1a-2	CA1a-3	Total Counts
1	U5S1_HUMAN	116 kDa U5 small nuclear ribonucleoprotein component	S19	100%	1.00	131	3	2	3	4	4	5	3	3	3	3	2	1	36
2	1433S_HUMAN	14-3-3 protein sigma	T217	48%	0.48	9	0	0	0	0	0	1	0	0	0	2	0	0	3
3	1433S_HUMAN	14-3-3 protein sigma	T228	99%	0.99	119	0	4	1	1	1	2	0	3	1	2	4	4	23
4	1433S_HUMAN	14-3-3 protein sigma	T231	18%	0.18	1	0	0	0	0	0	0	0	0	0	0	1	0	1
5	1433T_HUMAN	14-3-3 protein theta	T215	8%	0.08	1	0	0	0	0	0	0	0	1	0	0	0	0	1
6	1433Z_HUMAN	14-3-3 protein zeta/delta	T194	9%	0.09	1	0	0	0	0	1	0	0	0	0	0	0	0	1
7	1433Z_HUMAN	14-3-3 protein zeta/delta	T215	17%	0.17	22	0	0	0	0	1	0	1	1	2	0	0	0	5
8	1433Z_HUMAN	14-3-3 protein zeta/delta	T226	99%	0.99	606	11	10	10	6	7	13	9	9	10	12	12	9	118
9	TB182_HUMAN	182 kDa tankyrase-1-binding protein	S1024	100%	1.00	87	2	2	2	4	4	4	3	1	2	2	4	3	33
10	TB182_HUMAN	182 kDa tankyrase-1-binding protein	S1029	67%	0.67	4	0	0	0	0	0	0	0	0	0	0	2	0	2
11	TB182_HUMAN	182 kDa tankyrase-1-binding protein	S1473	99%	0.99	97	2	2	3	2	2	3	2	2	3	3	3	2	29
12	TB182_HUMAN	182 kDa tankyrase-1-binding protein	S1476	67%	0.67	22	0	0	0	1	1	0	1	1	0	0	0	1	5
13	TB182_HUMAN	182 kDa tankyrase-1-binding protein	S1552	17%	0.17	5	0	0	0	0	1	1	0	0	0	1	0	0	3

14	TB182_HUMAN	182 kDa tankyrase-1-binding protein	S1554	17%	0.17	33	5	2	1	3	2	1	0	1	0	0	0	1	16
15	TB182_HUMAN	182 kDa tankyrase-1-binding protein	S1558	17%	0.17	1	0	0	0	0	1	0	0	0	0	0	0	0	1
16	TB182_HUMAN	182 kDa tankyrase-1-binding protein	S1652	100%	1.00	2	0	0	0	0	0	0	0	0	0	1	0	0	1
17	TB182_HUMAN	182 kDa tankyrase-1-binding protein	S429	100%	1.00	61	4	10	1	0	0	1	6	2	1	3	1	1	30
18	TB182_HUMAN	182 kDa tankyrase-1-binding protein	S494	99%	0.99	419	13	6	7	11	7	3	6	3	1	12	9	2	80
19	TB182_HUMAN	182 kDa tankyrase-1-binding protein	S498	99%	0.99	273	9	2	1	7	5	0	6	3	1	7	6	1	48
20	TB182_HUMAN	182 kDa tankyrase-1-binding protein	S504	75%	0.75	189	5	3	6	7	8	6	5	5	3	6	6	3	63
21	TB182_HUMAN	182 kDa tankyrase-1-binding protein	S601	100%	1.00	51	0	0	0	0	2	0	0	0	0	2	4	1	9
22	TB182_HUMAN	182 kDa tankyrase-1-binding protein	S672	100%	1.00	19	1	0	0	0	1	4	0	0	0	0	1	0	7
23	TB182_HUMAN	182 kDa tankyrase-1-binding protein	S691	100%	1.00	331	1	1	1	3	7	6	6	8	6	13	16	15	83
24	TB182_HUMAN	182 kDa tankyrase-1-binding protein	S893	99%	0.99	2	0	0	0	0	0	0	0	0	0	0	1	0	1
25	TB182_HUMAN	182 kDa tankyrase-1-binding protein	S983	20%	0.20	8	2	0	0	2	1	1	0	0	1	0	0	1	8
26	TB182_HUMAN	182 kDa tankyrase-1-binding protein	S984	86%	0.86	15	2	0	0	4	3	0	0	0	1	4	0	1	15
27	TB182_HUMAN	182 kDa tankyrase-1-binding protein	S987	20%	0.20	2	0	0	0	0	0	0	2	0	0	0	0	0	2
28	TB182_HUMAN	182 kDa tankyrase-1-binding protein	T501	77%	0.77	214	6	5	4	6	9	8	7	4	6	2	7	2	66
29	TB182_HUMAN	182 kDa tankyrase-1-binding protein	T607	61%	0.61	1	0	0	0	0	0	0	0	0	0	0	0	1	1
30	TB182_HUMAN	182 kDa tankyrase-1-binding protein	T663	12%	0.12	1	1	0	0	0	0	0	0	0	0	0	0	0	1

31	OAS3_HUMAN	2'-5'-oligoadenylate synthase 3	S381	100%	1.00	1	1	0	0	0	0	0	0	0	0	0	0	0	1
32	OAS3_HUMAN	2'-5'-oligoadenylate synthase 3	S409	24%	0.24	1	1	0	0	0	0	0	0	0	0	0	0	0	1
33	OAS3_HUMAN	2'-5'-oligoadenylate synthase 3	T413	41%	0.41	1	1	0	0	0	0	0	0	0	0	0	0	0	1
34	PRS6A_HUMAN	26S protease regulatory subunit 6A	S9	100%	1.00	3	0	0	0	0	0	0	0	0	1	0	0	0	1
35	PSMD2_HUMAN	26S proteasome non-ATPase regulatory subunit 2	S16	100%	1.00	12	0	1	0	0	0	0	3	0	1	2	1	4	12
36	ODBA_HUMAN	2-oxoisovalerate dehydrogenase subunit alpha, mitochondrial	S295	97%	0.97	5	0	1	0	0	0	0	0	2	0	0	0	0	3
37	RM04_HUMAN	39S ribosomal protein L4, mitochondrial	S160	36%	0.36	3	0	0	0	0	0	0	0	0	0	1	1	0	2
38	RM04_HUMAN	39S ribosomal protein L4, mitochondrial	Y162	96%	0.96	3	0	0	0	0	0	0	0	0	0	1	1	0	2
39	RM04_HUMAN	39S ribosomal protein L4, mitochondrial	Y163	100%	1.00	3	0	0	0	0	0	0	0	0	0	1	1	0	2
40	HACD3_HUMAN	3-hydroxyacyl-CoA dehydratase 3	S114	100%	1.00	101	2	10	4	4	4	6	4	6	4	4	6	2	56
41	PDPK1_HUMAN	3-phosphoinositide-dependent protein kinase 1	S241	100%	1.00	104	2	3	2	1	0	0	3	1	0	4	2	0	18
42	RS12_HUMAN	40S ribosomal protein S12	T24	100%	1.00	363	7	9	7	9	11	8	9	10	5	7	5	7	94
43	RS3_HUMAN	40S ribosomal protein S3	T220	93%	0.93	30	1	1	0	0	0	0	0	0	1	4	1	2	10
44	RS3_HUMAN	40S ribosomal protein S3	T221	100%	1.00	868	14	21	17	16	17	13	15	16	14	26	27	22	218
45	RS8_HUMAN	40S ribosomal protein S8	T130	100%	1.00	12	0	0	0	0	0	0	0	0	0	2	0	0	2
46	XRN2_HUMAN	5'-3' exoribonuclease 2	S499	100%	1.00	4	1	0	1	1	0	1	0	0	0	0	0	0	4
47	XRN2_HUMAN	5'-3' exoribonuclease 2	S501	100%	1.00	4	1	0	1	1	0	1	0	0	0	0	0	0	4
48	XRN2_HUMAN	5'-3' exoribonuclease 2	S678	100%	1.00	25	0	0	0	4	0	0	2	2	0	0	0	0	8
49	AAKB2_HUMAN	5'-AMP-activated protein kinase subunit beta-2	S39	75%	0.75	2	0	0	0	0	0	0	0	0	0	0	2	0	2
50	AZI1_HUMAN	5-azacytidine-induced protein 1	T96	11%	0.11	1	0	1	0	0	0	0	0	0	0	0	0	0	1

51	5HT1D_HUMAN	5-hydroxytryptamine receptor 1D	S26	97%	0.97	1	0	0	0	0	0	0	0	0	0	1	0	0	1
52	5HT1D_HUMAN	5-hydroxytryptamine receptor 1D	T23	99%	0.99	1	0	0	0	0	0	0	0	0	0	1	0	0	1
53	5HT1D_HUMAN	5-hydroxytryptamine receptor 1D	T25	97%	0.97	1	0	0	0	0	0	0	0	0	0	1	0	0	1
54	68MP_HUMAN	6.8 kDa mitochondrial proteolipid	T18	59%	0.59	1	1	0	0	0	0	0	0	0	0	0	0	0	1
55	68MP_HUMAN	6.8 kDa mitochondrial proteolipid	Y17	75%	0.75	1	1	0	0	0	0	0	0	0	0	0	0	0	1
56	RLA0_HUMAN	60S acidic ribosomal protein P0	S304	100%	1.00	####	42	37	48	53	44	46	47	37	40	42	38	34	508
57	RLA0_HUMAN	60S acidic ribosomal protein P0	S307	100%	1.00	####	49	38	52	61	47	48	51	44	44	44	42	34	554
58	RLA0_HUMAN	60S acidic ribosomal protein P0	S46	100%	1.00	1	0	0	0	1	0	0	0	0	0	0	0	0	1
59	RLA0L_HUMAN	60S acidic ribosomal protein P0-like	S304	100%	1.00	####	42	37	48	53	44	46	47	37	40	42	38	34	508
60	RLA0L_HUMAN	60S acidic ribosomal protein P0-like	S307	100%	1.00	####	49	38	52	61	47	48	51	44	44	44	42	34	554
61	RLA0L_HUMAN	60S acidic ribosomal protein P0-like	S46	100%	1.00	1	0	0	0	1	0	0	0	0	0	0	0	0	1
62	RLA1_HUMAN	60S acidic ribosomal protein P1	S101	100%	1.00	####	69	44	60	79	49	58	66	44	47	45	45	42	648
63	RLA1_HUMAN	60S acidic ribosomal protein P1	S104	100%	1.00	####	86	53	69	89	66	67	71	62	57	56	47	50	773
64	RLA2_HUMAN	60S acidic ribosomal protein P2	S102	100%	1.00	####	69	44	60	79	49	58	66	44	47	45	45	42	648
65	RLA2_HUMAN	60S acidic ribosomal protein P2	S105	100%	1.00	####	86	53	69	89	66	67	71	62	57	56	47	50	773
66	RLA2_HUMAN	60S acidic ribosomal protein P2	S17	100%	1.00	5	0	0	0	0	0	0	0	0	0	0	1	0	1
67	RLA2_HUMAN	60S acidic ribosomal protein P2	S64	0%	0.00	2	0	0	0	1	0	0	0	0	0	0	0	0	1
68	RLA2_HUMAN	60S acidic ribosomal protein P2	S74	10%	0.10	5	0	0	0	0	0	0	0	0	0	1	0	0	1
69	RLA2_HUMAN	60S acidic ribosomal protein P2	S79	100%	1.00	131	0	0	0	1	2	1	2	0	0	8	5	7	26

70	RLA2_HUMAN	60S acidic ribosomal protein P2	S86	100%	1.00	32	0	0	0	0	0	0	0	1	0	0	0	0	1
71	NMD3_HUMAN	60S ribosomal export protein NMD3	S468	100%	1.00	130	1	1	2	3	5	3	4	3	3	2	0	2	29
72	RL14_HUMAN	60S ribosomal protein L14	S139	100%	1.00	26	0	0	0	0	0	0	0	0	0	6	5	10	21
73	RL18_HUMAN	60S ribosomal protein L18	S130	100%	1.00	2	0	1	0	0	0	0	0	0	0	0	0	1	2
74	RL22L_HUMAN	60S ribosomal protein L22-like 1	S118	100%	1.00	54	2	0	2	0	2	2	0	0	2	0	0	0	10
75	F262_HUMAN	6-phosphofructo-2-kinase/fructose-2,6-biphosphatase 2	S466	100%	1.00	3	0	0	0	1	0	0	0	0	0	1	0	0	2
76	F263_HUMAN	6-phosphofructo-2-kinase/fructose-2,6-biphosphatase 3	S34	100%	1.00	1	0	0	0	1	0	0	0	0	0	0	0	0	1
77	F263_HUMAN	6-phosphofructo-2-kinase/fructose-2,6-biphosphatase 3	T32	100%	1.00	1	0	0	0	1	0	0	0	0	0	0	0	0	1
78	F263_HUMAN	6-phosphofructo-2-kinase/fructose-2,6-biphosphatase 3	T36	100%	1.00	1	0	0	0	1	0	0	0	0	0	0	0	0	1
79	MEPCE_HUMAN	7SK snRNA methylphosphate capping enzyme	S254	100%	1.00	70	0	0	0	0	0	0	0	0	1	1	2	0	4
80	MEPCE_HUMAN	7SK snRNA methylphosphate capping enzyme	S57	43%	0.43	1	1	0	0	0	0	0	0	0	0	0	0	0	1
81	MEPCE_HUMAN	7SK snRNA methylphosphate capping enzyme	S69	88%	0.88	7	2	0	0	0	1	1	1	0	0	0	0	0	5
82	MCM3A_HUMAN	80 kDa MCM3-associated protein	S527	44%	0.44	1	0	0	0	0	0	0	0	0	0	0	0	1	1
83	MCM3A_HUMAN	80 kDa MCM3-associated protein	S529	75%	0.75	10	0	0	0	0	0	0	0	0	1	1	1	0	3
84	MCM3A_HUMAN	80 kDa MCM3-associated protein	T530	64%	0.64	6	0	1	0	0	1	0	0	0	0	1	1	0	4
85	MUTYH_HUMAN	A/G-specific adenine DNA glycosylase	S222	68%	0.68	1	0	0	0	0	0	0	0	1	0	0	0	0	1
86	MUTYH_HUMAN	A/G-specific adenine DNA glycosylase	T216	77%	0.77	1	0	0	0	0	0	0	0	1	0	0	0	0	1
87	MUTYH_HUMAN	A/G-specific adenine DNA glycosylase	T229	58%	0.58	1	0	0	0	0	0	0	0	1	0	0	0	0	1

88	MUTYH_HUMAN	A/G-specific adenine DNA glycosylase	Y215	80%	0.80	1	0	0	0	0	0	0	0	1	0	0	0	0	1
89	ABL2_HUMAN	Abelson tyrosine-protein kinase 2	S936	100%	1.00	2	0	0	0	0	0	0	0	0	0	0	2	0	2
90	AIM1_HUMAN	Absent in melanoma 1 protein	S19	25%	0.25	3	0	0	0	1	0	0	0	0	0	0	0	0	1
91	AIM1_HUMAN	Absent in melanoma 1 protein	S329	100%	1.00	26	0	0	0	0	0	0	0	0	0	5	4	3	12
92	AIM1_HUMAN	Absent in melanoma 1 protein	S533	100%	1.00	214	3	5	4	6	5	6	5	4	5	6	3	7	59
93	AIM1_HUMAN	Absent in melanoma 1 protein	S539	86%	0.86	129	0	2	3	2	4	2	4	4	4	4	6	3	38
94	AIM1_HUMAN	Absent in melanoma 1 protein	S543	91%	0.91	20	0	1	2	0	1	0	0	0	0	2	1	0	7
95	AIM1_HUMAN	Absent in melanoma 1 protein	S725	100%	1.00	11	0	0	0	0	0	1	0	0	0	0	1	2	4
96	AIM1_HUMAN	Absent in melanoma 1 protein	S75	100%	1.00	131	2	3	2	3	3	3	3	3	3	3	3	3	34
97	AIM1_HUMAN	Absent in melanoma 1 protein	S78	45%	0.45	4	0	0	0	0	0	0	0	0	0	0	0	1	1
98	AIM1_HUMAN	Absent in melanoma 1 protein	S81	79%	0.79	22	0	0	0	0	0	0	0	0	0	3	1	2	6
99	AIM1_HUMAN	Absent in melanoma 1 protein	T20	25%	0.25	150	0	0	0	6	6	5	6	0	4	4	5	5	41
100	AIM1_HUMAN	Absent in melanoma 1 protein	T524	1%	0.01	2	0	0	0	0	0	0	0	0	0	0	0	1	1
101	AIM1_HUMAN	Absent in melanoma 1 protein	T525	100%	1.00	76	0	2	1	5	2	1	0	2	1	3	3	3	23
102	AIM1_HUMAN	Absent in melanoma 1 protein	T530	12%	0.12	10	0	1	0	0	0	0	0	1	0	0	0	0	2
103	THIL_HUMAN	Acetyl-CoA acetyltransferase, mitochondrial	S155	38%	0.38	1	0	0	1	0	0	0	0	0	0	0	0	0	1
104	THIL_HUMAN	Acetyl-CoA acetyltransferase, mitochondrial	S157	38%	0.38	1	0	0	1	0	0	0	0	0	0	0	0	0	1
105	THIL_HUMAN	Acetyl-CoA acetyltransferase, mitochondrial	Y161	53%	0.53	1	0	0	1	0	0	0	0	0	0	0	0	0	1
106	ACACA_HUMAN	Acetyl-CoA carboxylase 1	S1259	79%	0.79	5	0	0	0	0	0	0	1	0	0	0	1	0	2

107	ACACA_HUMAN	Acetyl-CoA carboxylase 1	S1263	75%	0.75	4	0	0	0	0	0	0	1	0	0	0	1	0	2
108	ACACA_HUMAN	Acetyl-CoA carboxylase 1	S17	100%	1.00	43	1	0	0	0	0	1	1	0	0	0	0	0	3
109	ACACA_HUMAN	Acetyl-CoA carboxylase 1	S23	100%	1.00	93	1	0	0	0	2	2	1	0	0	6	3	2	17
110	ACACA_HUMAN	Acetyl-CoA carboxylase 1	S25	100%	1.00	67	2	0	0	0	2	2	1	0	0	0	1	0	8
111	ACACA_HUMAN	Acetyl-CoA carboxylase 1	S29	100%	1.00	588	11	8	15	10	12	9	13	8	6	8	7	6	113
112	ACACA_HUMAN	Acetyl-CoA carboxylase 1	S5	100%	1.00	43	1	0	0	0	0	1	1	0	0	0	0	0	3
113	ACSA_HUMAN	Acetyl-coenzyme A synthetase, cytoplasmic	S265	12%	0.12	10	0	0	0	0	1	0	0	0	0	0	0	2	3
114	ACSA_HUMAN	Acetyl-coenzyme A synthetase, cytoplasmic	S267	100%	1.00	206	0	1	1	1	1	2	0	2	0	26	20	20	74
115	ACSA_HUMAN	Acetyl-coenzyme A synthetase, cytoplasmic	S28	49%	0.49	10	0	0	0	0	0	0	0	0	0	0	1	2	3
116	ACSA_HUMAN	Acetyl-coenzyme A synthetase, cytoplasmic	S30	100%	1.00	66	0	1	0	0	1	0	0	0	0	5	8	5	20
117	ACSA_HUMAN	Acetyl-coenzyme A synthetase, cytoplasmic	T264	68%	0.68	6	0	0	0	0	0	0	0	0	0	0	0	1	1
118	AFAP1_HUMAN	Actin filament-associated protein 1	S264	75%	0.75	18	0	0	0	0	0	0	0	0	0	1	3	0	4
119	AFAP1_HUMAN	Actin filament-associated protein 1	S265	69%	0.69	40	0	0	0	0	0	0	0	1	0	2	2	2	7
120	AFAP1_HUMAN	Actin filament-associated protein 1	S282	69%	0.69	6	0	0	0	0	0	0	0	0	1	0	0	0	1
121	ABLM3_HUMAN	Actin-binding LIM protein 3	S503	100%	1.00	57	0	0	2	6	3	4	6	7	6	0	0	0	34
122	ABLM3_HUMAN	Actin-binding LIM protein 3	S504	100%	1.00	6	0	0	0	0	0	1	1	1	0	0	0	0	3
123	ANLN_HUMAN	Actin-binding protein anillin	S927	61%	0.61	1	0	0	0	0	0	0	0	0	0	0	1	0	1
124	MCAF1_HUMAN	Activating transcription factor 7-interacting protein 1	S113	100%	1.00	4	0	0	0	0	0	0	0	0	2	0	0	0	2

125	ADNP_HUMAN	Activity-dependent neuroprotector homeobox protein	S953	100%	1.00	3	0	1	0	0	0	0	0	0	0	0	0	0	1
126	ARFG3_HUMAN	ADP-ribosylation factor GTPase-activating protein 3	S453	69%	0.69	1	0	0	0	0	0	0	0	0	0	0	0	1	1
127	ARFG3_HUMAN	ADP-ribosylation factor GTPase-activating protein 3	S454	4%	0.04	1	0	0	0	0	0	0	0	0	0	0	1	0	1
128	AR6P4_HUMAN	ADP-ribosylation factor-like protein 6-interacting protein 4	S271	100%	1.00	27	1	7	4	0	2	0	3	0	2	0	0	0	19
129	AKA11_HUMAN	A-kinase anchor protein 11	S1242	25%	0.25	3	0	0	0	0	0	0	1	0	1	0	0	0	2
130	AKP13_HUMAN	A-kinase anchor protein 13	S1635	79%	0.79	1	0	0	1	0	0	0	0	0	0	0	0	0	1
131	AKP13_HUMAN	A-kinase anchor protein 13	S1645	51%	0.51	1	0	0	1	0	0	0	0	0	0	0	0	0	1
132	AKP13_HUMAN	A-kinase anchor protein 13	S1653	99%	0.99	1	0	0	1	0	0	0	0	0	0	0	0	0	1
133	AKP13_HUMAN	A-kinase anchor protein 13	S1876	100%	1.00	17	0	0	0	0	0	0	0	0	1	1	1	0	3
134	AKP13_HUMAN	A-kinase anchor protein 13	S2709	100%	1.00	43	0	0	1	0	0	0	0	0	0	5	4	2	12
135	AKP13_HUMAN	A-kinase anchor protein 13	S790	77%	0.77	1	0	0	0	0	0	0	0	0	0	1	0	0	1
136	AKP13_HUMAN	A-kinase anchor protein 13	S983	88%	0.88	1	0	0	0	0	0	0	0	0	0	1	0	0	1
137	AKAP2_HUMAN	A-kinase anchor protein 2	S152	100%	1.00	69	0	0	0	3	5	1	1	0	1	2	0	0	13
138	AKAP2_HUMAN	A-kinase anchor protein 2	S154	49%	0.49	7	0	0	0	0	0	0	1	0	1	0	0	0	2
139	AKAP2_HUMAN	A-kinase anchor protein 2	S155	35%	0.35	4	0	0	0	1	0	0	0	0	0	0	0	0	1
140	AKAP2_HUMAN	A-kinase anchor protein 2	S158	12%	0.13	2	0	0	0	0	1	0	0	0	0	0	0	0	1
141	AKAP2_HUMAN	A-kinase anchor protein 2	S630	51%	0.51	9	0	0	0	2	0	0	0	0	0	0	0	0	2
142	AKAP2_HUMAN	A-kinase anchor protein 2	S720	100%	1.00	18	0	0	0	4	6	4	0	0	0	0	0	0	14
143	AKAP2_HUMAN	A-kinase anchor protein 2	S778	99%	0.99	23	0	0	0	0	0	0	1	1	0	0	0	0	2

144	AKAP2_HUMAN	A-kinase anchor protein 2	T157	11%	0.11	1	0	0	0	1	0	0	0	0	0	0	0	0	1
145	AKAP2_HUMAN	A-kinase anchor protein 2	T780	55%	0.55	26	0	0	0	0	0	0	0	1	0	0	0	0	1
146	AKAP3_HUMAN	A-kinase anchor protein 3	S364	55%	0.55	1	0	0	0	1	0	0	0	0	0	0	0	0	1
147	AKAP3_HUMAN	A-kinase anchor protein 3	S651	91%	0.91	1	0	0	0	0	0	0	0	0	0	0	1	0	1
148	AKAP3_HUMAN	A-kinase anchor protein 3	T371	45%	0.45	1	0	0	0	1	0	0	0	0	0	0	0	0	1
149	AKAP3_HUMAN	A-kinase anchor protein 3	T646	75%	0.75	1	0	0	0	0	0	0	0	0	0	0	1	0	1
150	AKAP3_HUMAN	A-kinase anchor protein 3	Y641	95%	0.95	1	0	0	0	0	0	0	0	0	0	0	1	0	1
151	AAAS_HUMAN	Aladin	S29	72%	0.72	18	0	0	0	0	0	1	0	1	1	2	2	0	7
152	AAAS_HUMAN	Aladin	S3	75%	0.75	18	0	0	0	0	0	1	0	1	1	2	2	0	7
153	AAAS_HUMAN	Aladin	S30	77%	0.77	18	0	0	0	0	0	1	0	1	1	2	2	0	7
154	AAAS_HUMAN	Aladin	S33	89%	0.89	9	0	0	0	0	0	0	0	1	1	1	0	0	3
155	AAAS_HUMAN	Aladin	T17	40%	0.40	1	0	0	0	0	0	1	0	0	0	0	0	0	1
156	AAAS_HUMAN	Aladin	T27	55%	0.55	8	0	0	0	0	0	0	0	0	0	1	2	0	3
157	AK1A1_HUMAN	Alcohol dehydrogenase [NADP+]	S4	100%	1.00	1	0	0	1	0	0	0	0	0	0	0	0	0	1
158	ADO_HUMAN	Aldehyde oxidase	S1304	75%	0.75	3	0	0	0	1	0	0	0	1	1	0	0	0	3
159	ADO_HUMAN	Aldehyde oxidase	T1301	94%	0.94	3	0	0	0	1	0	0	0	1	1	0	0	0	3
160	ADA2C_HUMAN	Alpha-2C adrenergic receptor	S21	100%	1.00	3	0	0	0	0	0	0	1	0	0	0	1	0	2
161	ADA2C_HUMAN	Alpha-2C adrenergic receptor	S3	100%	1.00	3	0	0	0	0	0	0	1	0	0	0	1	0	2
162	FETUA_HUMAN	Alpha-2-HS-glycoprotein	S325	54%	0.54	1	0	0	0	0	0	0	0	1	0	0	0	0	1
163	FETUA_HUMAN	Alpha-2-HS-glycoprotein	S334	73%	0.73	1	0	0	0	0	0	0	0	1	0	0	0	0	1
164	FETUA_HUMAN	Alpha-2-HS-glycoprotein	T319	65%	0.65	1	0	0	0	0	0	0	0	1	0	0	0	0	1

165	SIA7F_HUMAN	Alpha-N-acetylgalactosaminide alpha-2,6-sialyltransferase 6	S271	95%	0.95	1	0	0	0	1	0	0	0	0	0	0	0	0	1
166	SIA7F_HUMAN	Alpha-N-acetylgalactosaminide alpha-2,6-sialyltransferase 6	T244	23%	0.23	1	0	0	0	1	0	0	0	0	0	0	0	0	1
167	SIA7F_HUMAN	Alpha-N-acetylgalactosaminide alpha-2,6-sialyltransferase 6	Y262	96%	0.96	1	0	0	0	1	0	0	0	0	0	0	0	0	1
168	SIA7F_HUMAN	Alpha-N-acetylgalactosaminide alpha-2,6-sialyltransferase 6	Y269	95%	0.95	1	0	0	0	1	0	0	0	0	0	0	0	0	1
169	AIMP2_HUMAN	Aminoacyl tRNA synthase complex-interacting multifunctional protein 2	T82	97%	0.97	9	0	0	0	0	0	0	0	0	0	1	0	1	2
170	AIMP2_HUMAN	Aminoacyl tRNA synthase complex-interacting multifunctional protein 2	T90	28%	0.28	2	0	0	0	0	0	0	0	0	0	0	1	0	1
171	AREG_HUMAN	Amphiregulin	S14	99%	0.99	1	0	0	1	0	0	0	0	0	0	0	0	0	1
172	AREG_HUMAN	Amphiregulin	S20	76%	0.76	1	0	0	1	0	0	0	0	0	0	0	0	0	1
173	AREG_HUMAN	Amphiregulin	S34	89%	0.89	1	0	0	1	0	0	0	0	0	0	0	0	0	1
174	APBA3_HUMAN	Amyloid beta A4 precursor protein-binding family A member 3	S11	50%	0.50	2	0	0	0	0	0	0	0	0	0	1	0	0	1
175	ZFAN3_HUMAN	AN1-type zinc finger protein 3	S117	86%	0.86	1	0	0	0	0	0	0	0	0	0	1	0	0	1
176	ZFAN3_HUMAN	AN1-type zinc finger protein 3	S122	91%	0.91	1	0	0	0	0	0	0	0	0	0	1	0	0	1
177	ZFAN3_HUMAN	AN1-type zinc finger protein 3	S129	89%	0.89	1	0	0	0	0	0	0	0	0	0	1	0	0	1
178	ZFAN3_HUMAN	AN1-type zinc finger protein 3	T120	46%	0.46	1	0	0	0	0	0	0	0	0	0	1	0	0	1
179	ZFAN5_HUMAN	AN1-type zinc finger protein 5	S54	23%	0.23	1	0	0	0	0	1	0	0	0	0	0	0	0	1
180	APC1_HUMAN	Anaphase-promoting complex subunit 1	S686	99%	0.99	21	0	0	0	0	0	0	1	0	0	0	0	0	1

181	APC1_HUMAN	Anaphase-promoting complex subunit 1	S688	100%	1.00	197	0	2	2	0	6	1	4	4	2	3	2	0	26
182	APC4_HUMAN	Anaphase-promoting complex subunit 4	S777	96%	0.96	42	0	1	1	1	2	0	1	1	2	0	1	1	11
183	APC4_HUMAN	Anaphase-promoting complex subunit 4	S779	96%	0.96	89	3	3	0	2	0	4	4	0	0	0	0	1	17
184	AUP1_HUMAN	Ancient ubiquitous protein 1	S350	15%	0.15	1	0	0	0	0	0	0	0	0	0	0	0	1	1
185	RAI14_HUMAN	Ankycorbin	S327	49%	0.49	1	0	0	0	0	0	0	1	0	0	0	0	0	1
186	BTBDB_HUMAN	Ankyrin repeat and BTB/POZ domain-containing protein BTBD11	S971	99%	0.99	19	0	0	0	0	1	0	0	0	0	0	0	0	1
187	BTBDB_HUMAN	Ankyrin repeat and BTB/POZ domain-containing protein BTBD11	S987	80%	0.80	4	0	0	0	0	1	0	0	0	0	0	0	0	1
188	BTBDB_HUMAN	Ankyrin repeat and BTB/POZ domain-containing protein BTBD11	T12	53%	0.53	1	0	1	0	0	0	0	0	0	0	0	0	0	1
189	BTBDB_HUMAN	Ankyrin repeat and BTB/POZ domain-containing protein BTBD11	Y970	99%	0.99	19	0	0	0	0	1	0	0	0	0	0	0	0	1
190	BTBDB_HUMAN	Ankyrin repeat and BTB/POZ domain-containing protein BTBD11	Y979	99%	0.99	19	0	0	0	0	1	0	0	0	0	0	0	0	1
191	ANKH1_HUMAN	Ankyrin repeat and KH domain-containing protein 1	S64	34%	0.34	1	0	0	0	0	1	0	0	0	0	0	0	0	1
192	ANKH1_HUMAN	Ankyrin repeat and KH domain-containing protein 1	S66	66%	0.66	1	0	0	0	0	1	0	0	0	0	0	0	0	1
193	ANKH1_HUMAN	Ankyrin repeat and KH domain-containing protein 1	S93	66%	0.66	13	1	1	1	0	1	0	1	0	0	0	0	0	5
194	ANKH1_HUMAN	Ankyrin repeat and KH domain-containing protein 1	S95	80%	0.80	11	1	0	1	0	1	0	0	0	0	0	0	0	3
195	ANKH1_HUMAN	Ankyrin repeat and KH domain-containing protein 1	T68	34%	0.34	1	0	0	0	0	1	0	0	0	0	0	0	0	1
196	ANKH1_HUMAN	Ankyrin repeat and KH domain-containing protein 1	T86	47%	0.47	5	0	1	0	0	0	0	1	0	0	0	0	0	2

197	ANR11_HUMAN	Ankyrin repeat domain-containing protein 11	S276	100%	1.00	2	0	0	1	0	0	0	0	0	0	0	1	0	2
198	AN13B_HUMAN	Ankyrin repeat domain-containing protein 13B	T208	67%	0.67	1	1	0	0	0	0	0	0	0	0	0	0	0	1
199	AN13B_HUMAN	Ankyrin repeat domain-containing protein 13B	Y205	67%	0.67	1	1	0	0	0	0	0	0	0	0	0	0	0	1
200	AN36C_HUMAN	Ankyrin repeat domain-containing protein 36C	S1154	25%	0.25	1	0	0	0	0	0	0	0	0	1	0	0	0	1
201	ANK3_HUMAN	Ankyrin-3	S1459	99%	0.99	1	0	0	0	0	0	0	0	0	0	0	0	1	1
202	ANK3_HUMAN	Ankyrin-3	S4298	100%	1.00	3	0	0	0	0	0	0	0	0	0	0	0	2	2
203	ANK3_HUMAN	Ankyrin-3	S4306	34%	0.34	1	0	0	0	0	0	0	0	0	1	0	0	0	1
204	ANK3_HUMAN	Ankyrin-3	T4310	34%	0.34	1	0	0	0	0	0	0	0	0	1	0	0	0	1
205	ANXA1_HUMAN	Annexin A1	S37	93%	0.93	26	0	0	0	0	0	0	0	0	0	4	6	6	16
206	ANXA1_HUMAN	Annexin A1	S5	100%	1.00	43	1	2	0	1	1	2	3	2	3	1	0	2	18
207	ANXA2_HUMAN	Annexin A2	S12	100%	1.00	16	0	0	0	0	0	1	0	0	1	0	0	0	2
208	ANXA2_HUMAN	Annexin A2	S127	100%	1.00	48	0	1	0	0	0	0	0	0	0	1	2	5	9
209	ANXA2_HUMAN	Annexin A2	T123	1%	0.01	3	0	0	0	0	0	0	0	0	0	1	0	1	2
210	ANXA2_HUMAN	Annexin A2	T19	96%	0.96	27	0	0	0	0	0	0	0	0	0	5	5	1	11
211	AP2A1_HUMAN	AP-2 complex subunit alpha-1	S655	78%	0.78	2	0	0	0	0	0	0	2	0	0	0	0	0	2
212	AP2B1_HUMAN	AP-2 complex subunit beta	S767	34%	0.34	5	0	0	0	0	0	0	1	0	0	0	1	1	3
213	AP2B1_HUMAN	AP-2 complex subunit beta	T768	22%	0.22	5	0	0	0	0	0	0	1	0	0	0	1	1	3
214	AAK1_HUMAN	AP2-associated protein kinase 1	S637	100%	1.00	259	5	6	6	6	6	5	6	6	7	2	0	1	56
215	AP3D1_HUMAN	AP-3 complex subunit delta-1	S658	100%	1.00	2	0	0	0	0	0	0	0	0	0	2	0	0	2

216	ACINU_HUMAN	Apoptotic chromatin condensation inducer in the nucleus	S478	100%	1.00	103	0	2	2	5	3	0	3	1	3	0	0	1	20
217	ACINU_HUMAN	Apoptotic chromatin condensation inducer in the nucleus	S481	61%	0.61	15	0	0	1	0	0	2	1	2	1	0	0	0	7
218	ACINU_HUMAN	Apoptotic chromatin condensation inducer in the nucleus	S482	49%	0.49	7	0	0	0	1	0	1	0	0	1	0	0	0	3
219	ACINU_HUMAN	Apoptotic chromatin condensation inducer in the nucleus	S490	100%	1.00	24	1	1	1	1	1	0	0	0	3	0	0	0	8
220	ACINU_HUMAN	Apoptotic chromatin condensation inducer in the nucleus	S710	100%	1.00	4	0	0	0	0	0	1	0	0	0	0	0	3	4
221	ACINU_HUMAN	Apoptotic chromatin condensation inducer in the nucleus	T414	100%	1.00	2	0	0	1	0	0	0	0	0	0	0	0	0	1
222	GIT1_HUMAN	ARF GTPase-activating protein GIT1	S362	51%	0.51	12	0	0	0	0	0	0	0	0	0	1	0	0	1
223	AGAP2_HUMAN	Arf-GAP with GTPase, ANK repeat and PH domain-containing protein 2	S248	79%	0.79	1	0	0	0	0	0	0	0	0	0	1	0	0	1
224	AGAP2_HUMAN	Arf-GAP with GTPase, ANK repeat and PH domain-containing protein 2	S251	27%	0.27	2	0	0	0	0	0	0	0	0	0	1	0	0	1
225	AGAP2_HUMAN	Arf-GAP with GTPase, ANK repeat and PH domain-containing protein 2	T239	40%	0.40	1	0	0	0	0	0	0	0	0	0	1	0	0	1
226	AGAP2_HUMAN	Arf-GAP with GTPase, ANK repeat and PH domain-containing protein 2	T249	98%	0.98	1	0	0	0	0	0	0	0	0	0	1	0	0	1
227	ASAP2_HUMAN	Arf-GAP with SH3 domain, ANK repeat and PH domain-containing protein 2	S701	100%	1.00	34	1	1	3	2	0	1	1	1	0	1	0	2	13
228	ARGL1_HUMAN	Arginine and glutamate-rich protein 1	S77	100%	1.00	9	0	0	0	0	0	0	0	0	0	4	1	0	5
229	RSRC2_HUMAN	Arginine/serine-rich coiled-coil protein 2	T6	100%	1.00	1	0	0	0	0	0	0	1	0	0	0	0	0	1

230	ARMC1_HUMAN	Armadillo repeat-containing protein 1	S112	73%	0.73	1	0	0	0	0	0	0	0	0	0	0	0	1	1
231	ARMC1_HUMAN	Armadillo repeat-containing protein 1	S246	37%	0.37	1	0	0	0	0	0	0	0	1	0	0	0	0	1
232	ARMC1_HUMAN	Armadillo repeat-containing protein 1	T235	55%	0.55	1	0	0	0	0	0	0	0	1	0	0	0	0	1
233	ARMC1_HUMAN	Armadillo repeat-containing protein 1	T248	37%	0.37	1	0	0	0	0	0	0	0	1	0	0	0	0	1
234	ARMC1_HUMAN	Armadillo repeat-containing protein 1	Y107	71%	0.71	1	0	0	0	0	0	0	0	0	0	0	0	1	1
235	ARSB_HUMAN	Arylsulfatase B	S417	61%	0.61	1	0	0	0	0	0	0	1	0	0	0	0	0	1
236	ARSB_HUMAN	Arylsulfatase B	S428	22%	0.22	1	0	0	0	0	0	0	1	0	0	0	0	0	1
237	ARSG_HUMAN	Arylsulfatase G	T371	86%	0.86	1	0	1	0	0	0	0	0	0	0	0	0	0	1
238	PEA15_HUMAN	Astrocytic phosphoprotein PEA-15	S116	100%	1.00	17	0	2	1	0	0	1	0	1	2	2	0	1	10
239	ASTN2_HUMAN	Astrotactin-2	S956	29%	0.29	1	0	0	1	0	0	0	0	0	0	0	0	0	1
240	ASTN2_HUMAN	Astrotactin-2	T960	32%	0.32	1	0	0	1	0	0	0	0	0	0	0	0	0	1
241	ATX1L_HUMAN	Ataxin-1-like	T333	0%	0.00	1	0	0	1	0	0	0	0	0	0	0	0	0	1
242	ATX2_HUMAN	Ataxin-2	S728	52%	0.52	19	1	2	1	0	0	0	1	0	1	1	0	0	7
243	ATX2_HUMAN	Ataxin-2	S736	7%	0.07	1	0	1	0	0	0	0	0	0	0	0	0	0	1
244	ATX2_HUMAN	Ataxin-2	T730	60%	0.60	14	0	0	0	1	0	1	0	0	1	1	3	0	7
245	ATX2L_HUMAN	Ataxin-2-like protein	S111	100%	1.00	47	3	0	0	2	0	3	0	2	2	4	4	0	20
246	ATX2L_HUMAN	Ataxin-2-like protein	S32	90%	0.90	350	10	10	9	9	0	6	9	8	7	9	10	4	91
247	ATX2L_HUMAN	Ataxin-2-like protein	S45	33%	0.33	11	1	0	0	0	0	0	0	0	0	0	0	1	2
248	ATX2L_HUMAN	Ataxin-2-like protein	S56	17%	0.17	1	0	0	0	1	0	0	0	0	0	0	0	0	1
249	ATX2L_HUMAN	Ataxin-2-like protein	S594	100%	1.00	97	3	2	3	4	4	2	3	3	3	8	9	10	54

250	ATX2L_HUMAN	Ataxin-2-like protein	T31	75%	0.75	52	2	5	0	3	0	0	2	3	3	1	0	3	22
251	ATX2L_HUMAN	Ataxin-2-like protein	T44	44%	0.44	3	0	0	0	0	0	0	0	0	0	0	1	0	1
252	SAMD1_HUMAN	Atherin	S161	100%	1.00	43	3	2	3	2	3	1	3	3	4	1	3	3	31
253	AHDC1_HUMAN	AT-hook DNA-binding motif-containing protein 1	S12	30%	0.30	2	0	0	0	0	0	0	0	0	0	1	0	0	1
254	AHDC1_HUMAN	AT-hook DNA-binding motif-containing protein 1	S17	37%	0.37	2	0	0	0	0	0	0	0	0	0	1	0	0	1
255	ATD2B_HUMAN	ATPase family AAA domain-containing protein 2B	S744	26%	0.26	1	0	0	0	0	0	0	1	0	0	0	0	0	1
256	ATD2B_HUMAN	ATPase family AAA domain-containing protein 2B	Y751	26%	0.26	1	0	0	0	0	0	0	1	0	0	0	0	0	1
257	ATD2B_HUMAN	ATPase family AAA domain-containing protein 2B	Y765	26%	0.26	1	0	0	0	0	0	0	1	0	0	0	0	0	1
258	WRIP1_HUMAN	ATPase WRNIP1	S75	95%	0.95	1	0	0	0	0	0	0	0	0	0	0	0	1	1
259	ABCAC_HUMAN	ATP-binding cassette sub-family A member 12	T1878	74%	0.74	1	0	1	0	0	0	0	0	0	0	0	0	0	1
260	ABCF1_HUMAN	ATP-binding cassette sub-family F member 1	S109	100%	1.00	725	14	17	19	15	13	12	13	15	13	4	7	6	148
261	ABCF1_HUMAN	ATP-binding cassette sub-family F member 1	T108	100%	1.00	78	1	2	0	3	1	2	2	0	2	0	0	1	14
262	ACLY_HUMAN	ATP-citrate synthase	S455	99%	0.99	12	0	0	0	0	0	0	0	0	0	5	3	4	12
263	ACLY_HUMAN	ATP-citrate synthase	S478	98%	0.98	9	0	0	0	0	0	0	0	0	0	4	2	0	6
264	ACLY_HUMAN	ATP-citrate synthase	S481	100%	1.00	102	0	0	0	0	0	0	0	0	0	12	13	12	37
265	DHX9_HUMAN	ATP-dependent RNA helicase A	S1077	100%	1.00	1	0	0	0	0	1	0	0	0	0	0	0	0	1
266	DHX9_HUMAN	ATP-dependent RNA helicase A	S77	8%	0.08	7	1	0	0	0	0	0	0	0	0	0	1	0	2
267	DHX9_HUMAN	ATP-dependent RNA helicase A	S87	100%	1.00	628	15	11	7	16	12	2	18	9	8	27	18	8	151
268	DHX9_HUMAN	ATP-dependent RNA helicase A	T92	38%	0.38	19	1	2	1	0	1	0	0	0	0	1	0	0	6

269	DHX9_HUMAN	ATP-dependent RNA helicase A	T94	10%	0.10	7	1	0	0	0	0	0	0	0	0	0	0	0	1
270	DHX9_HUMAN	ATP-dependent RNA helicase A	T97	0%	0.00	1	0	0	0	0	0	0	0	0	0	1	0	0	1
271	DDX24_HUMAN	ATP-dependent RNA helicase DDX24	S287	100%	1.00	53	1	1	0	2	1	1	0	0	1	0	0	1	8
272	DDX24_HUMAN	ATP-dependent RNA helicase DDX24	S295	73%	0.73	33	0	0	0	1	0	0	0	1	0	0	0	1	3
273	DDX24_HUMAN	ATP-dependent RNA helicase DDX24	T302	89%	0.89	20	1	0	0	0	0	0	0	0	0	1	0	0	2
274	DDX42_HUMAN	ATP-dependent RNA helicase DDX42	S185	99%	0.99	30	2	0	0	0	3	1	0	4	5	2	0	0	17
275	DDX54_HUMAN	ATP-dependent RNA helicase DDX54	S71	9%	0.09	2	0	0	0	0	0	0	0	0	1	0	0	0	1
276	DDX54_HUMAN	ATP-dependent RNA helicase DDX54	S75	100%	1.00	185	4	8	4	6	3	8	6	2	4	3	3	3	54
277	DDX54_HUMAN	ATP-dependent RNA helicase DDX54	T74	9%	0.09	9	0	0	0	0	2	0	0	0	0	0	0	0	2
278	DDX54_HUMAN	ATP-dependent RNA helicase DDX54	T81	4%	0.04	1	0	0	0	0	1	0	0	0	0	0	0	0	1
279	ARI1A_HUMAN	AT-rich interactive domain-containing protein 1A	S1755	50%	0.50	9	0	1	0	0	0	0	0	0	0	1	0	0	2
280	ARI1B_HUMAN	AT-rich interactive domain-containing protein 1B	S1555	99%	0.99	1	0	1	0	0	0	0	0	0	0	0	0	0	1
281	ARI1B_HUMAN	AT-rich interactive domain-containing protein 1B	S1629	84%	0.84	3	0	0	2	0	1	0	0	0	0	0	0	0	3
282	A16L1_HUMAN	Autophagy-related protein 16-1	S289	80%	0.80	5	0	0	0	0	0	0	1	0	0	1	1	1	4
283	A16L1_HUMAN	Autophagy-related protein 16-1	S290	38%	0.38	1	1	0	0	0	0	0	0	0	0	0	0	0	1
284	ATG2A_HUMAN	Autophagy-related protein 2 homolog A	S442	79%	0.79	1	0	1	0	0	0	0	0	0	0	0	0	0	1
285	ATG2A_HUMAN	Autophagy-related protein 2 homolog A	S445	88%	0.88	1	0	1	0	0	0	0	0	0	0	0	0	0	1
286	ATG2A_HUMAN	Autophagy-related protein 2 homolog A	S469	38%	0.38	1	0	1	0	0	0	0	0	0	0	0	0	0	1
287	ATG2A_HUMAN	Autophagy-related protein 2 homolog A	T430	80%	0.80	1	0	1	0	0	0	0	0	0	0	0	0	0	1

288	ATG2A_HUMAN	Autophagy-related protein 2 homolog A	T435	83%	0.83	1	0	1	0	0	0	0	0	0	0	0	0	0	1
289	ATG2A_HUMAN	Autophagy-related protein 2 homolog A	T437	66%	0.66	1	0	1	0	0	0	0	0	0	0	0	0	0	1
290	ATG2A_HUMAN	Autophagy-related protein 2 homolog A	T441	64%	0.64	1	0	1	0	0	0	0	0	0	0	0	0	0	1
291	ATG9A_HUMAN	Autophagy-related protein 9A	S828	100%	1.00	281	10	11	5	10	5	5	13	9	9	9	5	2	93
292	BAG3_HUMAN	BAG family molecular chaperone regulator 3	S377	100%	1.00	####	16	17	13	35	33	28	20	18	21	23	27	21	272
293	BAG3_HUMAN	BAG family molecular chaperone regulator 3	S381	95%	0.95	95	1	1	0	3	4	0	2	1	1	2	1	3	19
294	BAG3_HUMAN	BAG family molecular chaperone regulator 3	S385	94%	0.94	69	1	2	0	4	1	3	0	0	0	4	1	0	16
295	BAG3_HUMAN	BAG family molecular chaperone regulator 3	S386	99%	0.99	622	3	2	6	18	11	14	5	9	6	17	20	13	124
296	BAG3_HUMAN	BAG family molecular chaperone regulator 3	T406	100%	1.00	2	0	0	0	0	1	0	0	0	0	0	0	0	1
297	E41LA_HUMAN	Band 4.1-like protein 4A	S484	29%	0.29	1	0	0	0	0	0	0	0	1	0	0	0	0	1
298	E41LA_HUMAN	Band 4.1-like protein 4A	S486	29%	0.29	1	0	0	0	0	0	0	0	1	0	0	0	0	1
299	BARH2_HUMAN	BarH-like 2 homeobox protein	S10	15%	0.15	1	0	0	0	0	0	0	1	0	0	0	0	0	1
300	BARH2_HUMAN	BarH-like 2 homeobox protein	T15	46%	0.46	1	0	0	0	0	0	0	1	0	0	0	0	0	1
301	BASI_HUMAN	Basigin	S362	100%	1.00	60	4	6	0	0	4	4	0	6	4	2	0	0	30
302	BCL7C_HUMAN	B-cell CLL/lymphoma 7 protein family member C	S126	100%	1.00	36	1	0	1	1	2	0	4	0	2	0	0	0	11
303	BCL9_HUMAN	B-cell CLL/lymphoma 9 protein	S687	69%	0.69	1	0	0	0	0	0	0	0	0	0	1	0	0	1
304	BAD_HUMAN	Bcl2 antagonist of cell death	S118	100%	1.00	36	2	0	0	3	0	2	3	2	2	0	0	0	14
305	BCLF1_HUMAN	Bcl-2-associated transcription factor 1	S177	100%	1.00	2	0	0	1	0	0	0	1	0	0	0	0	0	2
306	BCLF1_HUMAN	Bcl-2-associated transcription factor 1	S183	81%	0.81	26	1	0	1	1	0	1	1	0	2	1	0	0	8

307	BCLF1_HUMAN	Bcl-2-associated transcription factor 1	S196	41%	0.41	1	0	0	0	0	0	0	1	0	0	0	0	0	1
308	BCLF1_HUMAN	Bcl-2-associated transcription factor 1	S285	51%	0.51	14	1	0	0	1	1	2	0	0	0	0	0	0	5
309	BCLF1_HUMAN	Bcl-2-associated transcription factor 1	S287	91%	0.91	4	0	1	0	0	0	0	0	0	0	0	0	0	1
310	BCLF1_HUMAN	Bcl-2-associated transcription factor 1	S389	30%	0.30	2	0	0	0	0	0	0	1	0	0	0	0	0	1
311	BCLF1_HUMAN	Bcl-2-associated transcription factor 1	S397	100%	1.00	264	6	6	6	6	6	6	8	6	6	0	6	6	68
312	BCLF1_HUMAN	Bcl-2-associated transcription factor 1	S512	100%	1.00	866	14	23	24	18	18	18	18	18	18	23	23	18	233
313	BCLF1_HUMAN	Bcl-2-associated transcription factor 1	S658	100%	1.00	492	14	17	17	12	12	12	12	12	10	18	15	17	168
314	BCLF1_HUMAN	Bcl-2-associated transcription factor 1	S660	33%	0.33	8	0	0	0	0	0	0	0	0	0	0	3	0	3
315	BCLF1_HUMAN	Bcl-2-associated transcription factor 1	T190	71%	0.71	10	0	0	0	1	0	0	1	0	0	0	0	0	2
316	BCLF1_HUMAN	Bcl-2-associated transcription factor 1	T661	99%	0.99	36	0	0	0	0	0	0	0	0	0	0	2	4	6
317	BCN1L_HUMAN	Beclin-1-like protein 1	S10	100%	1.00	1	0	0	0	0	0	0	0	0	0	0	0	1	1
318	BCN1L_HUMAN	Beclin-1-like protein 1	S12	100%	1.00	1	0	0	0	0	0	0	0	0	0	0	0	1	1
319	BCN1L_HUMAN	Beclin-1-like protein 1	S13	100%	1.00	1	0	0	0	0	0	0	0	0	0	0	0	1	1
320	BCN1L_HUMAN	Beclin-1-like protein 1	S4	100%	1.00	1	0	0	0	0	0	0	0	0	0	0	0	1	1
321	BCN1L_HUMAN	Beclin-1-like protein 1	T6	100%	1.00	1	0	0	0	0	0	0	0	0	0	0	0	1	1
322	BEND2_HUMAN	BEN domain-containing protein 2	S337	75%	0.75	1	1	0	0	0	0	0	0	0	0	0	0	0	1
323	BEND2_HUMAN	BEN domain-containing protein 2	Y321	25%	0.25	1	1	0	0	0	0	0	0	0	0	0	0	0	1
324	BEND2_HUMAN	BEN domain-containing protein 2	Y328	25%	0.25	1	1	0	0	0	0	0	0	0	0	0	0	0	1

325	GCNT1_HUMAN	Beta-1,3-galactosyl-O-glycosyl-glycoprotein beta-1,6-N-acetylglucosaminyltransferase	S30	100%	1.00	1	0	0	0	0	1	0	0	0	0	0	0	0	1
326	GCNT1_HUMAN	Beta-1,3-galactosyl-O-glycosyl-glycoprotein beta-1,6-N-acetylglucosaminyltransferase	Y18	25%	0.25	1	0	0	0	0	1	0	0	0	0	0	0	0	1
327	B4GT1_HUMAN	Beta-1,4-galactosyltransferase 1	S74	100%	1.00	33	1	0	0	2	1	1	3	2	2	0	0	0	12
328	SNTB2_HUMAN	Beta-2-syntrophin	S95	100%	1.00	101	0	0	0	0	0	0	0	0	0	10	9	6	25
329	SYEP_HUMAN	Bifunctional aminoacyl-tRNA synthetase	S886	100%	1.00	173	7	3	2	4	5	3	4	3	3	3	3	2	42
330	SYEP_HUMAN	Bifunctional aminoacyl-tRNA synthetase	T888	14%	0.14	8	0	0	1	0	0	0	0	0	0	0	0	1	2
331	COASY_HUMAN	Bifunctional coenzyme A synthase	S178	99%	0.99	27	4	0	0	0	0	1	3	1	0	0	0	0	9
332	COASY_HUMAN	Bifunctional coenzyme A synthase	S183	12%	0.13	4	0	0	0	0	0	1	0	0	0	0	0	0	1
333	COASY_HUMAN	Bifunctional coenzyme A synthase	T173	12%	0.13	2	0	1	0	0	0	0	0	0	0	0	0	0	1
334	PNKP_HUMAN	Bifunctional polynucleotide phosphatase/kinase	S469	28%	0.28	1	0	0	0	0	0	0	0	0	0	1	0	0	1
335	PNKP_HUMAN	Bifunctional polynucleotide phosphatase/kinase	Y482	45%	0.45	1	0	0	0	0	0	0	0	0	0	1	0	0	1
336	NCOAT_HUMAN	Bifunctional protein NCOAT	T709	82%	0.82	3	0	0	0	0	0	0	0	0	0	0	2	1	3
337	NCOAT_HUMAN	Bifunctional protein NCOAT	T711	9%	0.09	1	0	0	0	0	0	0	0	0	0	0	0	1	1
338	BCD1_HUMAN	Box C/D snoRNA protein 1	S25	100%	1.00	50	1	2	3	2	2	0	0	2	1	1	1	0	15
339	BAI1_HUMAN	Brain-specific angiogenesis inhibitor 1	S250	88%	0.88	2	0	0	0	0	0	0	0	0	1	0	0	0	1
340	BAI1_HUMAN	Brain-specific angiogenesis inhibitor 1	T249	99%	0.99	2	0	0	0	0	0	0	0	0	0	1	0	0	1
341	BAI1_HUMAN	Brain-specific angiogenesis inhibitor 1	T252	90%	0.90	4	0	0	0	0	0	0	0	0	1	1	0	0	2

342	BI2L1_HUMAN	Brain-specific angiogenesis inhibitor 1-associated protein 2-like protein 1	S261	88%	0.88	1	0	0	0	0	0	0	0	0	0	0	1	0	1
343	BI2L1_HUMAN	Brain-specific angiogenesis inhibitor 1-associated protein 2-like protein 1	S414	100%	1.00	122	2	1	0	2	4	2	3	3	2	4	3	2	28
344	BI2L2_HUMAN	Brain-specific angiogenesis inhibitor 1-associated protein 2-like protein 2	S465	100%	1.00	137	1	2	3	3	3	6	4	3	3	0	0	0	28
345	BSH_HUMAN	Brain-specific homeobox protein homolog	S14	30%	0.30	1	0	0	0	0	0	0	0	0	1	0	0	0	1
346	BSH_HUMAN	Brain-specific homeobox protein homolog	S7	31%	0.31	1	0	0	0	0	0	0	0	0	1	0	0	0	1
347	BCR_HUMAN	Breakpoint cluster region protein	S122	100%	1.00	362	11	7	5	10	7	8	9	7	4	11	11	11	101
348	BCAS1_HUMAN	Breast carcinoma-amplified sequence 1	T572	100%	1.00	1	0	0	0	0	0	0	0	0	0	0	0	1	1
349	BIG2_HUMAN	Brefeldin A-inhibited guanine nucleotide-exchange protein 2	S277	75%	0.75	15	2	0	0	0	2	2	0	1	1	1	2	1	12
350	BIG2_HUMAN	Brefeldin A-inhibited guanine nucleotide-exchange protein 2	S614	44%	0.44	1	1	0	0	0	0	0	0	0	0	0	0	0	1
351	BIG2_HUMAN	Brefeldin A-inhibited guanine nucleotide-exchange protein 2	T955	38%	0.38	1	0	0	0	0	0	0	1	0	0	0	0	0	1
352	BIG2_HUMAN	Brefeldin A-inhibited guanine nucleotide-exchange protein 2	T961	73%	0.73	1	0	0	0	0	0	0	1	0	0	0	0	0	1
353	BIG2_HUMAN	Brefeldin A-inhibited guanine nucleotide-exchange protein 2	Y944	82%	0.82	1	0	0	0	0	0	0	1	0	0	0	0	0	1
354	BABA1_HUMAN	BRISC and BRCA1-A complex member 1	S8	90%	0.90	51	5	2	2	1	2	3	1	1	2	0	2	3	24
355	BRPF3_HUMAN	Bromodomain and PHD finger-containing protein 3	S1036	100%	1.00	1	0	0	0	0	0	0	1	0	0	0	0	0	1
356	BRPF3_HUMAN	Bromodomain and PHD finger-containing protein 3	S1043	100%	1.00	1	0	0	0	0	0	0	1	0	0	0	0	0	1

357	BRWD1_HUMAN	Bromodomain and WD repeat-containing protein 1	S1139	56%	0.56	1	0	0	0	0	0	0	0	0	0	1	0	0	1
358	BRWD1_HUMAN	Bromodomain and WD repeat-containing protein 1	T1142	86%	0.86	1	0	0	0	0	0	0	0	0	0	1	0	0	1
359	BRD2_HUMAN	Bromodomain-containing protein 2	S301	61%	0.61	1	0	0	0	1	0	0	0	0	0	0	0	0	1
360	BRD8_HUMAN	Bromodomain-containing protein 8	S387	100%	1.00	13	1	0	0	0	0	0	0	0	0	0	0	0	1
361	BRD9_HUMAN	Bromodomain-containing protein 9	S588	100%	1.00	7	1	2	1	1	0	0	0	0	0	0	0	0	5
362	BTBD1_HUMAN	BTB/POZ domain-containing protein 1	S30	44%	0.44	6	0	0	0	0	0	0	0	0	0	1	0	0	1
363	BTBD1_HUMAN	BTB/POZ domain-containing protein 1	S32	55%	0.55	4	0	0	0	0	1	0	0	0	0	0	0	0	1
364	BTBD1_HUMAN	BTB/POZ domain-containing protein 1	S33	42%	0.42	8	0	0	0	0	0	0	0	0	1	0	0	0	1
365	KCD12_HUMAN	BTB/POZ domain-containing protein KCTD12	S176	87%	0.87	4	0	0	0	1	1	0	1	0	0	0	1	0	4
366	KCD12_HUMAN	BTB/POZ domain-containing protein KCTD12	T178	64%	0.64	3	0	0	0	0	0	0	0	0	0	1	0	0	1
367	KCTD5_HUMAN	BTB/POZ domain-containing protein KCTD5	S68	100%	1.00	1	0	0	0	0	0	0	0	0	0	0	0	1	1
368	KCTD5_HUMAN	BTB/POZ domain-containing protein KCTD5	Y71	100%	1.00	1	0	0	0	0	0	0	0	0	0	0	0	1	1
369	BUD13_HUMAN	BUD13 homolog	S271	100%	1.00	10	0	1	0	0	0	0	0	0	0	2	5	1	9
370	BYST_HUMAN	Bystin	S98	100%	1.00	48	3	8	1	1	6	1	1	7	1	1	0	0	30
371	C1QRF_HUMAN	C1q-related factor	S120	99%	0.99	2	0	0	1	0	0	0	0	0	0	0	0	0	1
372	C1QRF_HUMAN	C1q-related factor	T125	32%	0.32	2	0	0	1	0	0	0	0	0	0	0	0	0	1
373	C1QRF_HUMAN	C1q-related factor	Y124	54%	0.54	2	0	0	1	0	0	0	0	0	0	0	0	0	1
374	PYR1_HUMAN	CAD protein	S1859	100%	1.00	24	1	2	3	1	2	2	2	4	2	2	1	0	22

375	PDE1C_HUMAN	Calcium/calmodulin-dependent 3',5'-cyclic nucleotide phosphodiesterase 1C	S3	100%	1.00	29	0	0	0	0	0	0	0	0	0	1	0	0	1
376	SCMC2_HUMAN	Calcium-binding mitochondrial carrier protein SCaMC-2	S221	90%	0.90	1	0	0	0	0	0	1	0	0	0	0	0	0	1
377	SCMC2_HUMAN	Calcium-binding mitochondrial carrier protein SCaMC-2	T231	69%	0.69	1	0	0	0	0	0	1	0	0	0	0	0	0	1
378	CHSP1_HUMAN	Calcium-regulated heat stable protein 1	S17	71%	0.71	1	0	0	0	0	0	0	0	0	0	1	0	0	1
379	CHSP1_HUMAN	Calcium-regulated heat stable protein 1	S26	32%	0.32	2	0	2	0	0	0	0	0	0	0	0	0	0	2
380	CALM_HUMAN	Calmodulin	S39	21%	0.21	1	1	0	0	0	0	0	0	0	0	0	0	0	1
381	CALM_HUMAN	Calmodulin	T71	65%	0.65	1	1	0	0	0	0	0	0	0	0	0	0	0	1
382	CAMP1_HUMAN	Calmodulin-regulated spectrin-associated protein 1	S1080	100%	1.00	226	0	0	1	5	5	6	5	4	6	7	15	9	63
383	CAMP1_HUMAN	Calmodulin-regulated spectrin-associated protein 1	S563	100%	1.00	1	0	0	0	0	0	0	0	0	0	0	1	0	1
384	CAMP1_HUMAN	Calmodulin-regulated spectrin-associated protein 1	S575	84%	0.84	4	0	0	0	0	0	0	0	0	0	0	1	0	1
385	CAMP1_HUMAN	Calmodulin-regulated spectrin-associated protein 1	T1082	3%	0.03	1	0	0	0	0	0	0	0	0	0	0	1	0	1
386	CALX_HUMAN	Calnexin	S583	100%	1.00	110	5	3	4	4	4	4	5	5	3	3	6	4	50
387	CPNS1_HUMAN	Calpain small subunit 1	S6	94%	0.94	13	0	0	0	0	0	0	0	0	1	0	0	0	1
388	CPNS1_HUMAN	Calpain small subunit 1	T57	100%	1.00	18	0	1	0	0	0	1	0	0	1	0	1	0	4
389	CAN3_HUMAN	Calpain-3	S40	28%	0.28	1	0	0	0	0	0	0	0	0	1	0	0	0	1
390	CAN3_HUMAN	Calpain-3	S44	88%	0.88	1	0	0	0	0	0	0	0	0	1	0	0	0	1
391	CAN3_HUMAN	Calpain-3	S48	69%	0.69	1	0	0	0	0	0	0	0	0	1	0	0	0	1

392	ICAL_HUMAN	Calpastatin	S133	17%	0.17	1	0	0	0	0	0	0	0	0	0	0	1	0	1
393	ICAL_HUMAN	Calpastatin	S236	12%	0.13	5	0	0	0	0	0	0	0	0	1	0	0	1	2
394	ICAL_HUMAN	Calpastatin	S237	44%	0.44	11	0	0	0	0	0	1	0	1	1	1	0	0	4
395	ICAL_HUMAN	Calpastatin	S243	99%	0.99	169	4	1	0	4	2	0	4	1	4	15	12	10	57
396	ICAL_HUMAN	Calpastatin	S364	100%	1.00	62	0	0	0	0	1	1	0	0	0	6	5	2	15
397	ICAL_HUMAN	Calpastatin	S366	48%	0.48	83	0	0	0	2	1	1	0	0	0	7	6	8	25
398	ICAL_HUMAN	Calpastatin	T135	77%	0.77	4	0	0	0	0	1	0	0	0	0	0	1	0	2
399	ICAL_HUMAN	Calpastatin	T240	22%	0.22	30	0	1	0	0	1	1	1	0	0	1	1	1	7
400	ICAL_HUMAN	Calpastatin	T245	56%	0.56	89	0	1	1	3	1	4	2	3	1	5	1	2	24
401	KAP0_HUMAN	cAMP-dependent protein kinase type I-alpha regulatory subunit	S77	7%	0.07	10	2	0	0	0	0	0	2	1	0	0	0	0	5
402	KAP0_HUMAN	cAMP-dependent protein kinase type I-alpha regulatory subunit	S83	100%	1.00	155	8	6	6	0	0	1	11	12	14	0	0	0	58
403	KAP2_HUMAN	cAMP-dependent protein kinase type II-alpha regulatory subunit	S78	100%	1.00	103	10	8	4	0	5	4	7	1	4	2	3	1	49
404	KAP2_HUMAN	cAMP-dependent protein kinase type II-alpha regulatory subunit	S80	100%	1.00	103	10	8	4	0	5	4	7	1	4	2	3	1	49
405	ARP19_HUMAN	cAMP-regulated phosphoprotein 19	S62	100%	1.00	6	0	4	0	0	0	0	0	0	0	0	0	2	6
406	CT47A_HUMAN	Cancer/testis antigen 47A	T244	14%	0.14	1	0	0	0	0	0	0	0	0	1	0	0	0	1
407	CLIP2_HUMAN	CAP-Gly domain-containing linker protein 2	S352	100%	1.00	157	5	10	8	3	8	4	3	3	3	6	6	6	65
408	CLIP2_HUMAN	CAP-Gly domain-containing linker protein 2	T354	94%	0.94	2	1	0	0	0	0	0	0	0	0	0	0	0	1
409	CAPR1_HUMAN	Caprin-1	S335	55%	0.55	7	1	1	0	0	1	0	0	0	0	1	2	1	7
410	CAPSD_HASV8	Capsid polyprotein	T593	11%	0.11	1	0	0	0	0	0	0	0	0	0	0	1	0	1
411	CAH6_HUMAN	Carbonic anhydrase 6	T69	42%	0.42	1	0	0	0	0	0	0	0	0	1	0	0	0	1
412	CAH6_HUMAN	Carbonic anhydrase 6	T73	42%	0.42	1	0	0	0	0	0	0	0	0	1	0	0	0	1

413	CAH6_HUMAN	Carbonic anhydrase 6	Y71	42%	0.42	1	0	0	0	0	0	0	0	0	1	0	0	0	1
414	CEA16_HUMAN	Carcinoembryonic antigen-related cell adhesion molecule 16	S2	83%	0.83	1	0	0	0	0	0	0	0	1	0	0	0	0	1
415	CEA16_HUMAN	Carcinoembryonic antigen-related cell adhesion molecule 16	S6	83%	0.83	1	0	0	0	0	0	0	0	1	0	0	0	0	1
416	CEA16_HUMAN	Carcinoembryonic antigen-related cell adhesion molecule 16	S9	83%	0.83	1	0	0	0	0	0	0	0	1	0	0	0	0	1
417	CEA16_HUMAN	Carcinoembryonic antigen-related cell adhesion molecule 16	T16	83%	0.83	1	0	0	0	0	0	0	0	1	0	0	0	0	1
418	CEA16_HUMAN	Carcinoembryonic antigen-related cell adhesion molecule 16	Y8	83%	0.83	1	0	0	0	0	0	0	0	1	0	0	0	0	1
419	CPT1A_HUMAN	Carnitine O-palmitoyltransferase 1, liver isoform	S106	92%	0.92	1	0	0	0	0	0	0	0	0	0	0	1	0	1
420	CPT1A_HUMAN	Carnitine O-palmitoyltransferase 1, liver isoform	S99	79%	0.79	1	0	0	0	0	0	0	0	0	0	0	1	0	1
421	CPT1A_HUMAN	Carnitine O-palmitoyltransferase 1, liver isoform	T101	77%	0.77	1	0	0	0	0	0	0	0	0	0	0	1	0	1
422	CPT1A_HUMAN	Carnitine O-palmitoyltransferase 1, liver isoform	T112	100%	1.00	1	0	0	0	0	0	0	0	0	0	0	1	0	1
423	CPT1A_HUMAN	Carnitine O-palmitoyltransferase 1, liver isoform	T121	100%	1.00	1	0	0	0	0	0	0	0	0	0	0	1	0	1
424	CPT1A_HUMAN	Carnitine O-palmitoyltransferase 1, liver isoform	T90	51%	0.51	1	0	0	0	0	0	0	0	0	0	0	1	0	1
425	CPT1B_HUMAN	Carnitine O-palmitoyltransferase 1, muscle isoform	S623	6%	0.06	1	0	0	0	0	0	0	0	0	0	0	1	0	1
426	CSKI2_HUMAN	Caskin-2	S700	8%	0.08	1	0	0	0	0	0	0	0	0	0	1	0	0	1

427	CASP4_HUMAN	Caspase-4	S83	100%	1.00	2	0	0	0	0	0	0	0	0	0	1	0	1	2
428	CTNA1_HUMAN	Catenin alpha-1	S641	100%	1.00	134	7	5	2	2	4	4	0	0	0	2	6	0	32
429	CTNA1_HUMAN	Catenin alpha-1	T634	100%	1.00	2	0	0	0	0	0	1	0	0	0	0	0	0	1
430	CTNB1_HUMAN	Catenin beta-1	S552	100%	1.00	33	3	9	0	0	3	1	1	2	1	1	1	0	22
431	CTNB1_HUMAN	Catenin beta-1	T551	99%	0.99	4	0	1	0	0	0	0	0	0	0	2	0	0	3
432	CTNB1_HUMAN	Catenin beta-1	T556	33%	0.33	5	1	1	0	0	0	1	0	0	0	0	1	1	5
433	CTND1_HUMAN	Catenin delta-1	S252	100%	1.00	23	2	4	2	0	3	2	0	2	1	1	2	0	19
434	CTND1_HUMAN	Catenin delta-1	S346	100%	1.00	4	0	0	0	0	1	0	0	0	1	0	0	0	2
435	CTND1_HUMAN	Catenin delta-1	S349	100%	1.00	21	0	0	0	0	1	0	0	0	1	0	0	0	2
436	CTSR3_HUMAN	Cation channel sperm-associated protein 3	Y103	100%	1.00	1	0	0	0	0	0	0	0	0	1	0	0	0	1
437	MPRI_HUMAN	Cation-independent mannose-6-phosphate receptor	S2401	13%	0.13	4	1	0	0	0	0	0	0	0	0	0	0	0	1
438	MPRI_HUMAN	Cation-independent mannose-6-phosphate receptor	S2409	100%	1.00	126	4	6	2	2	7	0	9	9	2	4	6	3	54
439	MPRI_HUMAN	Cation-independent mannose-6-phosphate receptor	S2484	100%	1.00	491	10	11	13	12	10	12	14	17	13	13	13	12	150
440	CATR1_HUMAN	CATR tumorigenic conversion 1 protein	T14	100%	1.00	5	1	0	1	0	0	0	0	0	0	0	1	0	3
441	CTIF_HUMAN	CBP80/20-dependent translation initiation factor	S299	100%	1.00	19	0	0	0	2	0	0	0	0	0	0	0	1	3
442	CTIF_HUMAN	CBP80/20-dependent translation initiation factor	T302	1%	0.01	1	0	0	0	0	0	0	0	0	0	0	0	1	1
443	CCL26_HUMAN	C-C motif chemokine 26	S14	32%	0.32	1	0	0	0	0	0	0	1	0	0	0	0	0	1
444	CCL26_HUMAN	C-C motif chemokine 26	S8	32%	0.32	1	0	0	0	0	0	0	1	0	0	0	0	0	1
445	CNOT3_HUMAN	CCR4-NOT transcription complex subunit 3	S506	39%	0.39	4	1	0	0	0	0	0	0	1	0	0	0	0	2

446	CNOT3_HUMAN	CCR4-NOT transcription complex subunit 3	S507	25%	0.25	18	0	0	0	1	0	0	0	0	1	1	2	0	5
447	CNOT3_HUMAN	CCR4-NOT transcription complex subunit 3	S511	29%	0.29	20	0	0	0	2	1	0	0	0	0	1	0	0	4
448	CNOT3_HUMAN	CCR4-NOT transcription complex subunit 3	S513	54%	0.54	34	0	1	0	0	1	0	3	1	1	1	0	1	9
449	CNOT3_HUMAN	CCR4-NOT transcription complex subunit 3	T509	15%	0.15	12	1	0	0	0	0	0	0	0	0	0	0	1	2
450	CNOT4_HUMAN	CCR4-NOT transcription complex subunit 4	S324	100%	1.00	10	0	0	0	1	0	0	0	0	0	0	0	0	1
451	CD2AP_HUMAN	CD2-associated protein	S193	19%	0.19	1	0	0	0	0	0	0	0	0	0	1	0	0	1
452	CD2AP_HUMAN	CD2-associated protein	S458	100%	1.00	138	4	0	6	6	10	6	6	6	6	10	10	8	78
453	CD2AP_HUMAN	CD2-associated protein	T168	12%	0.12	1	0	0	0	0	0	0	0	0	0	0	1	0	1
454	CD44_HUMAN	CD44 antigen	S697	100%	1.00	4	1	2	0	0	0	0	0	1	0	0	0	0	4
455	CD44_HUMAN	CD44 antigen	S704	62%	0.62	20	1	1	0	0	0	0	0	3	1	1	1	0	8
456	CD44_HUMAN	CD44 antigen	S706	100%	1.00	206	6	8	1	5	9	7	10	8	7	8	4	4	77
457	BORG5_HUMAN	Cdc42 effector protein 1	S192	100%	1.00	6	0	0	0	0	0	0	0	0	0	0	2	0	2
458	BORG5_HUMAN	Cdc42 effector protein 1	T367	25%	0.25	2	0	0	0	0	0	0	0	0	0	2	0	0	2
459	BORG2_HUMAN	Cdc42 effector protein 3	S100	100%	1.00	5	0	0	0	1	0	0	0	0	0	2	2	0	5
460	BORG2_HUMAN	Cdc42 effector protein 3	T111	94%	0.94	1	0	1	0	0	0	0	0	0	0	0	0	0	1
461	BORG4_HUMAN	Cdc42 effector protein 4	S174	100%	1.00	61	0	0	0	0	2	0	1	0	0	2	4	3	12
462	BORG4_HUMAN	Cdc42 effector protein 4	S18	100%	1.00	3	0	0	0	1	0	0	0	0	0	0	0	1	2
463	BORG4_HUMAN	Cdc42 effector protein 4	T23	85%	0.85	1	0	0	0	0	0	1	0	0	0	0	0	0	1
464	CIP4_HUMAN	Cdc42-interacting protein 4	S296	100%	1.00	583	13	9	14	15	13	10	13	14	12	22	21	15	171
465	CIP4_HUMAN	Cdc42-interacting protein 4	S299	66%	0.66	17	0	0	0	1	0	0	1	1	0	0	1	1	5
466	CIP4_HUMAN	Cdc42-interacting protein 4	S304	27%	0.27	2	1	0	0	0	0	0	0	0	0	0	0	0	1

467	CDCA7_HUMAN	Cell division cycle-associated protein 7	S33	26%	0.26	1	0	0	0	0	0	0	0	1	0	0	0	0	1
468	CDCA7_HUMAN	Cell division cycle-associated protein 7	S34	43%	0.43	1	0	0	0	0	0	0	0	1	0	0	0	0	1
469	CDCA7_HUMAN	Cell division cycle-associated protein 7	S37	33%	0.33	1	0	0	0	0	0	0	0	1	0	0	0	0	1
470	CDCA7_HUMAN	Cell division cycle-associated protein 7	Y22	35%	0.35	1	0	0	0	0	0	0	0	1	0	0	0	0	1
471	CE164_HUMAN	Centrosomal protein of 164 kDa	T765	100%	1.00	1	0	0	0	0	0	0	0	0	0	0	0	1	1
472	CE170_HUMAN	Centrosomal protein of 170 kDa	S1112	100%	1.00	7	0	0	0	1	0	0	0	1	0	0	0	0	2
473	CE170_HUMAN	Centrosomal protein of 170 kDa	S1515	100%	1.00	25	0	0	0	0	1	0	0	0	1	1	3	0	6
474	CE192_HUMAN	Centrosomal protein of 192 kDa	S99	61%	0.61	1	0	0	0	0	0	0	0	0	0	0	0	1	1
475	CE192_HUMAN	Centrosomal protein of 192 kDa	T92	46%	0.46	1	0	0	0	0	0	0	0	0	0	0	0	1	1
476	CEP55_HUMAN	Centrosomal protein of 55 kDa	S428	96%	0.96	21	0	0	0	0	0	0	0	0	0	0	1	0	1
477	CEP55_HUMAN	Centrosomal protein of 55 kDa	T430	97%	0.97	14	0	0	0	0	0	0	0	0	0	0	0	1	1
478	CHMP3_HUMAN	Charged multivesicular body protein 3	S200	100%	1.00	64	3	3	2	3	2	3	2	1	3	2	3	1	28
479	CHMP7_HUMAN	Charged multivesicular body protein 7	S417	100%	1.00	70	0	0	0	3	2	2	0	0	1	3	5	1	17
480	CCKN_HUMAN	Cholecystokinin	S31	28%	0.28	1	0	0	0	0	0	0	0	0	0	1	0	0	1
481	CAF1A_HUMAN	Chromatin assembly factor 1 subunit A	S65	100%	1.00	7	0	0	0	0	0	1	0	0	0	0	0	0	1
482	CAF1A_HUMAN	Chromatin assembly factor 1 subunit A	S775	76%	0.76	9	0	1	0	0	0	2	0	0	0	0	1	0	4
483	CAF1A_HUMAN	Chromatin assembly factor 1 subunit A	S777	37%	0.37	11	0	0	0	1	1	0	1	1	1	0	0	0	5
484	CAF1A_HUMAN	Chromatin assembly factor 1 subunit A	T779	10%	0.10	5	0	1	0	0	0	0	0	1	1	0	0	0	3

485	BAP18_HUMAN	Chromatin complexes subunit BAP18	S96	100%	1.00	136	2	1	3	2	3	3	2	0	2	3	3	3	27
486	CHD3_HUMAN	Chromodomain-helicase-DNA-binding protein 3	S1601	100%	1.00	21	4	4	4	0	1	0	4	4	0	0	0	0	21
487	CHD3_HUMAN	Chromodomain-helicase-DNA-binding protein 3	T1595	7%	0.07	2	0	0	1	0	1	0	0	0	0	0	0	0	2
488	CHD8_HUMAN	Chromodomain-helicase-DNA-binding protein 8	S1995	50%	0.50	1	0	0	1	0	0	0	0	0	0	0	0	0	1
489	CHD8_HUMAN	Chromodomain-helicase-DNA-binding protein 8	S2046	86%	0.86	3	0	0	1	0	0	0	0	0	0	0	0	0	1
490	CHD9_HUMAN	Chromodomain-helicase-DNA-binding protein 9	S550	100%	1.00	1	0	0	1	0	0	0	0	0	0	0	0	0	1
491	CTF18_HUMAN	Chromosome transmission fidelity protein 18 homolog	S871	100%	1.00	41	1	0	0	0	0	0	1	0	0	1	0	0	3
492	KIF4A_HUMAN	Chromosome-associated kinesin KIF4A	T1181	100%	1.00	2	0	0	0	0	0	0	0	0	1	0	0	0	1
493	CTRO_HUMAN	Citron Rho-interacting kinase	S480	100%	1.00	6	0	0	0	0	0	0	0	0	0	0	2	0	2
494	JIP3_HUMAN	C-Jun-amino-terminal kinase-interacting protein 3	S26	75%	0.75	1	0	0	0	0	0	0	0	0	0	0	0	1	1
495	JIP3_HUMAN	C-Jun-amino-terminal kinase-interacting protein 3	S30	79%	0.79	1	0	0	0	0	0	0	0	0	0	0	0	1	1
496	JIP3_HUMAN	C-Jun-amino-terminal kinase-interacting protein 3	S35	80%	0.80	1	0	0	0	0	0	0	0	0	0	0	0	1	1
497	JIP3_HUMAN	C-Jun-amino-terminal kinase-interacting protein 3	Y15	96%	0.96	1	0	0	0	0	0	0	0	0	0	0	0	1	1
498	JIP3_HUMAN	C-Jun-amino-terminal kinase-interacting protein 3	Y37	74%	0.74	1	0	0	0	0	0	0	0	0	0	0	0	1	1
499	JIP4_HUMAN	C-Jun-amino-terminal kinase-interacting protein 4	S203	100%	1.00	9	0	0	0	0	1	0	0	0	0	2	3	1	7
500	JIP4_HUMAN	C-Jun-amino-terminal kinase-interacting protein 4	S265	92%	0.92	1	0	0	0	0	0	0	0	0	0	1	0	0	1
501	JIP4_HUMAN	C-Jun-amino-terminal kinase-interacting protein 4	S268	84%	0.84	1	0	0	0	0	0	0	0	0	0	1	0	0	1

502	JIP4_HUMAN	C-Jun-amino-terminal kinase-interacting protein 4	S283	64%	0.64	1	0	0	0	0	0	0	0	0	0	1	0	0	1
503	JIP4_HUMAN	C-Jun-amino-terminal kinase-interacting protein 4	T217	100%	1.00	49	0	0	0	0	0	1	0	0	0	3	2	3	9
504	JIP4_HUMAN	C-Jun-amino-terminal kinase-interacting protein 4	T280	78%	0.78	1	0	0	0	0	0	0	0	0	0	1	0	0	1
505	JIP4_HUMAN	C-Jun-amino-terminal kinase-interacting protein 4	T287	74%	0.74	1	0	0	0	0	0	0	0	0	0	1	0	0	1
506	JIP4_HUMAN	C-Jun-amino-terminal kinase-interacting protein 4	T290	77%	0.77	1	0	0	0	0	0	0	0	0	0	1	0	0	1
507	JIP4_HUMAN	C-Jun-amino-terminal kinase-interacting protein 4	T292	69%	0.69	1	0	0	0	0	0	0	0	0	0	1	0	0	1
508	CLSPN_HUMAN	Claspin	S1289	100%	1.00	2	0	0	0	0	0	0	0	0	0	0	0	1	1
509	CSTF2_HUMAN	Cleavage stimulation factor subunit 2	S524	56%	0.56	1	1	0	0	0	0	0	0	0	0	0	0	0	1
510	CSTF3_HUMAN	Cleavage stimulation factor subunit 3	S691	100%	1.00	135	7	2	2	3	3	0	2	3	2	2	1	4	31
511	CLAP1_HUMAN	CLIP-associating protein 1	S797	91%	0.91	96	0	2	2	2	1	2	1	2	1	0	1	1	15
512	CLAP1_HUMAN	CLIP-associating protein 1	T796	25%	0.25	2	0	0	0	0	0	0	1	0	0	0	0	0	1
513	CLAP1_HUMAN	CLIP-associating protein 1	T798	85%	0.85	72	1	1	1	2	1	0	2	1	3	3	2	1	18
514	CLAP2_HUMAN	CLIP-associating protein 2	S295	90%	0.90	2	0	0	0	0	0	0	1	0	0	0	0	0	1
515	CLAP2_HUMAN	CLIP-associating protein 2	S299	75%	0.75	2	0	0	0	0	0	0	1	0	0	0	0	0	1
516	CLAP2_HUMAN	CLIP-associating protein 2	S596	100%	1.00	195	3	4	0	6	6	4	4	0	0	0	2	1	30
517	CLAP2_HUMAN	CLIP-associating protein 2	T277	84%	0.84	2	0	0	0	0	0	0	1	0	0	0	0	0	1
518	CLAP2_HUMAN	CLIP-associating protein 2	Y278	86%	0.86	2	0	0	0	0	0	0	1	0	0	0	0	0	1
519	CLAP2_HUMAN	CLIP-associating protein 2	Y300	72%	0.72	2	0	0	0	0	0	0	1	0	0	0	0	0	1

520	CLASR_HUMAN	CLK4-associating serine/arginine rich protein	S331	39%	0.39	5	0	0	0	1	0	1	0	0	0	1	0	1	4
521	CLASR_HUMAN	CLK4-associating serine/arginine rich protein	S335	100%	1.00	371	7	10	6	10	6	8	8	6	6	7	8	5	87
522	CLASR_HUMAN	CLK4-associating serine/arginine rich protein	T327	78%	0.78	47	3	3	2	2	3	4	3	3	1	2	1	3	30
523	CLASR_HUMAN	CLK4-associating serine/arginine rich protein	T330	74%	0.74	77	2	2	3	1	3	1	2	0	4	1	1	0	20
524	CLASR_HUMAN	CLK4-associating serine/arginine rich protein	T353	8%	0.08	1	0	0	0	0	0	0	0	0	0	0	0	1	1
525	SIA4C_HUMAN	CMP-N-acetylneuraminate-beta-galactosamide-alpha-2,3-sialyltransferase 4	S312	26%	0.26	1	0	0	0	0	0	0	0	0	0	0	0	1	1
526	FA5_HUMAN	Coagulation factor V	S1914	100%	1.00	1	0	0	0	0	0	0	1	0	0	0	0	0	1
527	FA5_HUMAN	Coagulation factor V	T1915	100%	1.00	1	0	0	0	0	0	0	1	0	0	0	0	0	1
528	FA12_HUMAN	Coagulation factor XII	S541	69%	0.69	1	0	0	0	0	0	0	0	0	0	0	0	1	1
529	FA12_HUMAN	Coagulation factor XII	S563	46%	0.46	1	0	0	0	0	0	0	0	0	0	0	0	1	1
530	FA12_HUMAN	Coagulation factor XII	T556	46%	0.46	1	0	0	0	0	0	0	0	0	0	0	0	1	1
531	COPA_HUMAN	Coatomer subunit alpha	S173	100%	1.00	7	0	0	0	0	0	0	0	0	0	0	2	2	4
532	COPB2_HUMAN	Coatomer subunit beta'	T861	78%	0.78	8	0	0	0	0	0	0	0	0	0	0	0	1	1
533	COPD_HUMAN	Coatomer subunit delta	S259	76%	0.76	1	0	0	0	0	0	0	0	0	0	0	0	1	1
534	COPD_HUMAN	Coatomer subunit delta	T258	79%	0.79	1	0	0	0	0	0	0	0	0	0	0	0	1	1
535	COPD_HUMAN	Coatomer subunit delta	T262	93%	0.93	1	0	0	0	0	0	0	0	0	0	0	0	1	1
536	COPE_HUMAN	Coatomer subunit epsilon	S99	100%	1.00	49	0	2	0	2	2	4	4	4	6	4	0	6	34
537	COF1_HUMAN	Cofilin-1	S3	100%	1.00	614	17	22	18	17	19	14	14	13	17	9	10	9	179
538	STAG1_HUMAN	Cohesin subunit SA-1	S20	99%	0.99	2	0	0	0	0	0	0	0	0	0	0	1	0	1
539	STAG1_HUMAN	Cohesin subunit SA-1	S24	99%	0.99	2	0	0	0	0	0	0	0	0	0	0	1	0	1
540	STAG2_HUMAN	Cohesin subunit SA-2	S17	39%	0.39	2	0	0	0	0	1	0	0	0	0	0	0	0	1

541	STAG2_HUMAN	Cohesin subunit SA-2	S22	60%	0.60	3	0	0	1	0	0	0	0	0	0	0	0	0	1
542	STAG2_HUMAN	Cohesin subunit SA-2	S23	41%	0.41	7	1	0	1	0	1	0	0	0	0	0	0	0	3
543	STAG2_HUMAN	Cohesin subunit SA-2	T19	39%	0.39	1	0	0	0	0	1	0	0	0	0	0	0	0	1
544	STAG2_HUMAN	Cohesin subunit SA-2	T25	28%	0.28	6	0	0	2	0	0	0	0	0	0	0	0	0	2
545	C2D1A_HUMAN	Coiled-coil and C2 domain-containing protein 1A	S118	25%	0.25	2	0	0	0	1	0	0	0	0	0	0	0	0	1
546	CC135_HUMAN	Coiled-coil domain-containing protein 135	S381	50%	0.50	1	0	0	0	0	0	1	0	0	0	0	0	0	1
547	CC135_HUMAN	Coiled-coil domain-containing protein 135	T378	50%	0.50	1	0	0	0	0	0	1	0	0	0	0	0	0	1
548	CC135_HUMAN	Coiled-coil domain-containing protein 135	T386	50%	0.50	1	0	0	0	0	0	1	0	0	0	0	0	0	1
549	CC141_HUMAN	Coiled-coil domain-containing protein 141	S120	61%	0.61	5	0	0	0	1	1	0	0	0	0	1	0	0	3
550	CC141_HUMAN	Coiled-coil domain-containing protein 141	S134	60%	0.60	5	0	0	0	1	1	0	0	0	0	1	0	0	3
551	CC165_HUMAN	Coiled-coil domain-containing protein 165	S767	100%	1.00	4	0	1	2	0	0	1	0	0	0	0	0	0	4
552	CCD43_HUMAN	Coiled-coil domain-containing protein 43	T139	96%	0.96	1	0	0	1	0	0	0	0	0	0	0	0	0	1
553	CCDC6_HUMAN	Coiled-coil domain-containing protein 6	S240	100%	1.00	4	0	0	0	0	1	0	1	0	0	0	1	1	4
554	CCDC6_HUMAN	Coiled-coil domain-containing protein 6	S244	100%	1.00	23	0	0	0	0	1	0	1	0	0	2	2	4	10
555	CCD61_HUMAN	Coiled-coil domain-containing protein 61	S335	70%	0.70	1	0	1	0	0	0	0	0	0	0	0	0	0	1
556	CCD80_HUMAN	Coiled-coil domain-containing protein 80	T708	60%	0.60	1	0	0	0	0	0	0	0	0	1	0	0	0	1
557	CCD80_HUMAN	Coiled-coil domain-containing protein 80	T717	89%	0.89	1	0	0	0	0	0	0	0	0	1	0	0	0	1

558	CCD80_HUMAN	Coiled-coil domain-containing protein 80	Y705	90%	0.90	1	0	0	0	0	0	0	0	0	1	0	0	0	1
559	CCD86_HUMAN	Coiled-coil domain-containing protein 86	S18	100%	1.00	545	22	20	22	10	19	11	15	4	4	18	21	18	184
560	CCD86_HUMAN	Coiled-coil domain-containing protein 86	S21	7%	0.07	16	3	1	1	0	1	1	0	0	0	3	2	0	12
561	CCD86_HUMAN	Coiled-coil domain-containing protein 86	S47	100%	1.00	566	11	9	7	15	10	9	9	7	7	7	6	10	107
562	CCD86_HUMAN	Coiled-coil domain-containing protein 86	S80	100%	1.00	65	0	0	0	0	3	0	0	0	0	0	0	0	3
563	CCD86_HUMAN	Coiled-coil domain-containing protein 86	S91	100%	1.00	750	13	13	11	10	17	13	15	8	12	8	9	3	132
564	CCD86_HUMAN	Coiled-coil domain-containing protein 86	T23	4%	0.04	6	0	1	0	0	0	1	0	0	0	0	0	0	2
565	CC88B_HUMAN	Coiled-coil domain-containing protein 88B	S1063	100%	1.00	1	0	0	0	0	0	0	0	0	0	0	0	1	1
566	CC88B_HUMAN	Coiled-coil domain-containing protein 88B	T1067	100%	1.00	1	0	0	0	0	0	0	0	0	0	0	0	1	1
567	CCDC9_HUMAN	Coiled-coil domain-containing protein 9	S386	14%	0.14	4	0	0	0	0	0	1	0	0	0	0	0	0	1
568	CCDC9_HUMAN	Coiled-coil domain-containing protein 9	S390	90%	0.90	10	0	0	0	0	2	1	0	1	0	0	0	0	4
569	CC90B_HUMAN	Coiled-coil domain-containing protein 90B, mitochondrial	S91	42%	0.42	1	0	0	0	0	0	0	0	0	1	0	0	0	1
570	CC90B_HUMAN	Coiled-coil domain-containing protein 90B, mitochondrial	Y99	15%	0.15	1	0	0	0	0	0	0	0	0	1	0	0	0	1
571	CCD94_HUMAN	Coiled-coil domain-containing protein 94	S211	100%	1.00	16	0	1	0	0	0	0	1	0	0	0	0	0	2
572	CCD94_HUMAN	Coiled-coil domain-containing protein 94	S213	100%	1.00	14	0	1	0	0	0	0	1	0	0	0	0	0	2
573	CHCH3_HUMAN	Coiled-coil-helix-coiled-coil-helix domain-containing protein 3, mitochondrial	S50	20%	0.20	3	0	0	0	0	0	2	0	0	0	0	0	0	2

574	COCA1_HUMAN	Collagen alpha-1(XII) chain	S2009	20%	0.20	1	0	1	0	0	0	0	0	0	0	0	0	0	1
575	COCA1_HUMAN	Collagen alpha-1(XII) chain	S2016	17%	0.17	1	0	1	0	0	0	0	0	0	0	0	0	0	1
576	COHA1_HUMAN	Collagen alpha-1(XVII) chain	S85	100%	1.00	35	2	3	0	0	1	1	0	0	0	1	2	0	10
577	COHA1_HUMAN	Collagen alpha-1(XVII) chain	S88	51%	0.51	18	0	3	0	0	1	0	0	0	0	1	1	0	6
578	COHA1_HUMAN	Collagen alpha-1(XVII) chain	S93	97%	0.97	21	0	0	0	0	1	0	0	0	0	3	1	1	6
579	COHA1_HUMAN	Collagen alpha-1(XVII) chain	T97	0%	0.00	2	0	0	0	0	0	0	0	0	0	0	1	0	1
580	CO4A3_HUMAN	Collagen alpha-3(IV) chain	S2	100%	1.00	1	0	0	0	0	0	0	1	0	0	0	0	0	1
581	CO4A3_HUMAN	Collagen alpha-3(IV) chain	S28	100%	1.00	2	0	0	0	0	0	0	1	0	0	0	0	0	1
582	CO6A3_HUMAN	Collagen alpha-3(VI) chain	S2672	97%	0.97	4	0	0	0	0	0	0	1	0	0	0	0	0	1
583	CO6A3_HUMAN	Collagen alpha-3(VI) chain	S2679	61%	0.61	4	0	0	0	0	0	0	1	0	0	0	0	0	1
584	CRX_HUMAN	Cone-rod homeobox protein	S12	65%	0.65	1	0	1	0	0	0	0	0	0	0	0	0	0	1
585	CRX_HUMAN	Cone-rod homeobox protein	S19	75%	0.75	1	0	1	0	0	0	0	0	0	0	0	0	0	1
586	CRX_HUMAN	Cone-rod homeobox protein	Y11	64%	0.64	1	0	1	0	0	0	0	0	0	0	0	0	0	1
587	CRX_HUMAN	Cone-rod homeobox protein	Y4	43%	0.43	1	0	1	0	0	0	0	0	0	0	0	0	0	1
588	COG1_HUMAN	Conserved oligomeric Golgi complex subunit 1	S7	98%	0.98	2	0	0	0	0	1	0	0	1	0	0	0	0	2
589	COG1_HUMAN	Conserved oligomeric Golgi complex subunit 1	T6	99%	0.99	2	0	0	0	0	1	0	0	1	0	0	0	0	2
590	COG2_HUMAN	Conserved oligomeric Golgi complex subunit 2	S718	100%	1.00	1	1	0	0	0	0	0	0	0	0	0	0	0	1
591	COG2_HUMAN	Conserved oligomeric Golgi complex subunit 2	S720	100%	1.00	1	1	0	0	0	0	0	0	0	0	0	0	0	1
592	COG4_HUMAN	Conserved oligomeric Golgi complex subunit 4	S11	51%	0.51	1	0	0	0	0	0	0	1	0	0	0	0	0	1
593	CSN3_HUMAN	COP9 signalosome complex subunit 3	S300	98%	0.98	1	0	0	0	0	1	0	0	0	0	0	0	0	1
594	CIR1_HUMAN	Corepressor interacting with RBPJ 1	S202	100%	1.00	4	2	0	0	0	0	1	0	0	0	0	0	0	3

595	CXXC1_HUMAN	CpG-binding protein	S511	68%	0.68	1	0	0	0	0	0	0	0	1	0	0	0	0	1
596	CXXC1_HUMAN	CpG-binding protein	S517	55%	0.55	2	0	0	0	0	0	0	0	1	0	0	0	0	1
597	CXXC1_HUMAN	CpG-binding protein	T513	66%	0.66	2	0	0	0	0	0	0	0	1	0	0	0	0	1
598	CXXC1_HUMAN	CpG-binding protein	T527	31%	0.31	1	0	0	0	0	0	0	0	1	0	0	0	0	1
599	CRTC2_HUMAN	CREB-regulated transcription coactivator 2	S433	100%	1.00	7	0	0	0	2	1	0	0	0	0	0	0	0	3
600	CTBP2_HUMAN	C-terminal-binding protein 2	S424	40%	0.40	11	1	0	0	0	0	0	0	0	0	1	0	0	2
601	CTBP2_HUMAN	C-terminal-binding protein 2	S428	77%	0.77	24	1	2	0	0	0	1	1	2	0	1	1	0	9
602	CTBP2_HUMAN	C-terminal-binding protein 2	T414	18%	0.18	11	1	1	0	0	0	0	0	0	1	0	1	0	4
603	CTBP2_HUMAN	C-terminal-binding protein 2	T433	17%	0.17	21	1	3	0	0	0	0	1	1	0	1	0	2	9
604	CT2NL_HUMAN	CTTNBP2 N-terminal-like protein	S488	100%	1.00	8	0	0	0	0	0	0	0	0	0	0	1	1	2
605	CUZD1_HUMAN	CUB and zona pellucida-like domain-containing protein 1	S313	55%	0.55	1	0	0	0	0	0	1	0	0	0	0	0	0	1
606	CTGE5_HUMAN	Cutaneous T-cell lymphoma-associated antigen 5	S536	100%	1.00	3	0	1	0	0	0	0	0	0	0	0	0	0	1
607	CTGE5_HUMAN	Cutaneous T-cell lymphoma-associated antigen 5	S548	100%	1.00	3	0	1	0	0	0	0	0	0	0	0	0	0	1
608	ATF2_HUMAN	Cyclic AMP-dependent transcription factor ATF-2	S112	100%	1.00	35	0	1	0	0	0	0	0	0	0	0	0	0	1
609	CNGB1_HUMAN	Cyclic nucleotide-gated cation channel beta-1	S561	40%	0.40	9	0	1	1	0	0	0	0	0	0	0	1	0	3
610	CNGB1_HUMAN	Cyclic nucleotide-gated cation channel beta-1	T556	40%	0.40	5	0	1	0	0	0	0	0	0	0	0	1	0	2
611	CNGB1_HUMAN	Cyclic nucleotide-gated cation channel beta-1	T559	40%	0.40	4	0	0	1	0	0	0	0	0	0	0	0	0	1
612	CDK1_HUMAN	Cyclin-dependent kinase 1	Y15	100%	1.00	159	2	4	4	4	4	3	0	6	3	1	4	5	40
613	CD11A_HUMAN	Cyclin-dependent kinase 11A	S271	100%	1.00	5	0	0	0	2	0	0	0	0	2	0	0	0	4

614	CD11B_HUMAN	Cyclin-dependent kinase 11B	S283	100%	1.00	5	0	0	0	2	0	0	0	0	2	0	0	0	4
615	CDK12_HUMAN	Cyclin-dependent kinase 12	S383	100%	1.00	9	1	0	0	0	0	0	0	0	1	0	0	0	2
616	CDK12_HUMAN	Cyclin-dependent kinase 12	S385	100%	1.00	9	1	0	0	0	0	0	0	0	1	0	0	0	2
617	CDK12_HUMAN	Cyclin-dependent kinase 12	S681	100%	1.00	91	3	3	3	4	4	2	3	2	0	3	1	1	29
618	CDK12_HUMAN	Cyclin-dependent kinase 12	S685	100%	1.00	61	2	1	3	3	2	1	2	1	0	2	0	0	17
619	CDK13_HUMAN	Cyclin-dependent kinase 13	T1246	100%	1.00	19	2	1	1	0	1	0	1	0	1	2	1	1	11
620	CDK16_HUMAN	Cyclin-dependent kinase 16	S119	100%	1.00	67	3	4	4	1	4	4	5	4	2	4	3	3	41
621	CDK16_HUMAN	Cyclin-dependent kinase 16	S138	85%	0.85	4	0	0	0	0	0	0	0	0	0	1	0	1	2
622	CDK16_HUMAN	Cyclin-dependent kinase 16	S153	100%	1.00	51	4	6	2	0	2	0	0	0	0	6	6	6	32
623	CDK17_HUMAN	Cyclin-dependent kinase 17	S146	100%	1.00	27	0	4	4	0	0	0	0	0	0	4	3	0	15
624	CDK17_HUMAN	Cyclin-dependent kinase 17	S180	100%	1.00	23	0	5	3	0	0	0	0	0	0	2	2	0	12
625	CDK17_HUMAN	Cyclin-dependent kinase 17	S51	23%	0.23	1	0	0	1	0	0	0	0	0	0	0	0	0	1
626	CDK17_HUMAN	Cyclin-dependent kinase 17	T62	64%	0.64	1	0	0	1	0	0	0	0	0	0	0	0	0	1
627	CCNL2_HUMAN	Cyclin-L2	S330	100%	1.00	11	0	0	1	1	0	0	0	1	1	0	0	0	4
628	CCNT2_HUMAN	Cyclin-T2	S637	64%	0.64	1	0	0	0	0	1	0	0	0	0	0	0	0	1
629	CSRP1_HUMAN	Cysteine and glycine-rich protein 1	S192	100%	1.00	16	0	0	2	0	2	0	2	0	4	2	0	0	12
630	ATG4B_HUMAN	Cysteine protease ATG4B	S383	100%	1.00	220	5	6	5	6	5	4	7	5	3	5	3	2	56
631	SYCC_HUMAN	Cysteinyl-tRNA synthetase, cytoplasmic	S305	23%	0.23	1	0	0	0	0	0	0	0	0	1	0	0	0	1
632	COX5B_HUMAN	Cytochrome c oxidase subunit 5B, mitochondrial	S71	100%	1.00	1	0	0	0	0	0	0	0	0	0	0	0	1	1

633	COX5B_HUMAN	Cytochrome c oxidase subunit 5B, mitochondrial	T73	100%	1.00	1	0	0	0	0	0	0	0	0	0	0	0	1	1
634	COX5B_HUMAN	Cytochrome c oxidase subunit 5B, mitochondrial	Y62	100%	1.00	1	0	0	0	0	0	0	0	0	0	0	0	1	1
635	CP2S1_HUMAN	Cytochrome P450 2S1	S343	100%	1.00	1	0	0	0	0	0	1	0	0	0	0	0	0	1
636	CP2S1_HUMAN	Cytochrome P450 2S1	T348	100%	1.00	1	0	0	0	0	0	1	0	0	0	0	0	0	1
637	CP4F8_HUMAN	Cytochrome P450 4F8	S17	83%	0.83	1	0	0	0	0	0	0	0	0	1	0	0	0	1
638	CP4F8_HUMAN	Cytochrome P450 4F8	S2	43%	0.43	1	0	0	0	0	0	0	0	0	1	0	0	0	1
639	CP4F8_HUMAN	Cytochrome P450 4F8	S28	83%	0.83	1	0	0	0	0	0	0	0	0	1	0	0	0	1
640	CYTIP_HUMAN	Cytohesin-interacting protein	S314	20%	0.20	1	0	0	0	0	0	0	0	0	0	1	0	0	1
641	CYTIP_HUMAN	Cytohesin-interacting protein	S322	48%	0.48	1	0	0	0	0	0	0	0	0	0	1	0	0	1
642	CYTIP_HUMAN	Cytohesin-interacting protein	S323	20%	0.20	1	0	0	0	0	0	0	0	0	0	1	0	0	1
643	DYHC1_HUMAN	Cytoplasmic dynein 1 heavy chain 1	S3331	95%	0.95	1	0	0	0	0	0	1	0	0	0	0	0	0	1
644	DC1I2_HUMAN	Cytoplasmic dynein 1 intermediate chain 2	S78	97%	0.97	1	0	0	0	1	0	0	0	0	0	0	0	0	1
645	DC1I2_HUMAN	Cytoplasmic dynein 1 intermediate chain 2	S87	55%	0.55	1	0	0	0	1	0	0	0	0	0	0	0	0	1
646	DC1I2_HUMAN	Cytoplasmic dynein 1 intermediate chain 2	Y80	99%	0.99	1	0	0	0	1	0	0	0	0	0	0	0	0	1
647	DC1L1_HUMAN	Cytoplasmic dynein 1 light intermediate chain 1	S207	100%	1.00	73	3	2	0	0	2	1	0	1	2	4	6	3	24
648	DC1L1_HUMAN	Cytoplasmic dynein 1 light intermediate chain 1	S510	100%	1.00	153	1	4	4	6	6	6	5	4	6	4	3	4	53
649	DC1L1_HUMAN	Cytoplasmic dynein 1 light intermediate chain 1	S516	96%	0.96	229	4	3	3	6	4	5	6	3	4	7	8	9	62
650	DC1L1_HUMAN	Cytoplasmic dynein 1 light intermediate chain 1	T508	30%	0.30	7	0	0	1	0	0	0	0	0	0	1	1	1	4

651	DC1L1_HUMAN	Cytoplasmic dynein 1 light intermediate chain 1	T512	67%	0.67	19	0	1	0	1	2	0	0	1	0	1	2	1	9
652	DC1L1_HUMAN	Cytoplasmic dynein 1 light intermediate chain 1	T515	74%	0.74	16	0	0	0	0	0	0	0	0	0	2	0	0	2
653	DC1L2_HUMAN	Cytoplasmic dynein 1 light intermediate chain 2	S203	80%	0.80	21	0	0	0	0	0	0	0	0	0	1	2	0	3
654	DC1L2_HUMAN	Cytoplasmic dynein 1 light intermediate chain 2	S205	80%	0.80	32	0	0	0	0	0	0	0	0	0	0	2	0	2
655	DYHC2_HUMAN	Cytoplasmic dynein 2 heavy chain 1	S2947	84%	0.84	1	0	0	0	0	0	0	0	0	0	0	1	0	1
656	DYHC2_HUMAN	Cytoplasmic dynein 2 heavy chain 1	T2940	99%	0.99	1	0	0	0	0	0	0	0	0	0	0	1	0	1
657	NCK1_HUMAN	Cytoplasmic protein NCK1	S85	100%	1.00	380	6	8	10	9	8	8	10	8	11	10	10	8	106
658	NCK1_HUMAN	Cytoplasmic protein NCK1	S89	93%	0.93	57	3	2	1	0	1	1	0	2	1	2	3	0	16
659	NCK1_HUMAN	Cytoplasmic protein NCK1	S91	48%	0.48	12	0	0	0	1	1	0	0	0	0	0	0	3	5
660	CKAP2_HUMAN	Cytoskeleton-associated protein 2	S202	100%	1.00	1	0	0	0	0	0	0	0	0	0	1	0	0	1
661	CKAP2_HUMAN	Cytoskeleton-associated protein 2	T205	100%	1.00	1	0	0	0	0	0	0	0	0	0	1	0	0	1
662	CKAP4_HUMAN	Cytoskeleton-associated protein 4	S515	65%	0.65	1	0	0	0	0	0	0	0	0	0	1	0	0	1
663	CKAP4_HUMAN	Cytoskeleton-associated protein 4	T512	18%	0.18	1	0	0	0	0	0	0	0	0	0	1	0	0	1
664	CKAP5_HUMAN	Cytoskeleton-associated protein 5	S1134	50%	0.50	1	1	0	0	0	0	0	0	0	0	0	0	0	1
665	CKAP5_HUMAN	Cytoskeleton-associated protein 5	T773	88%	0.88	1	0	0	0	0	0	1	0	0	0	0	0	0	1
666	CKAP5_HUMAN	Cytoskeleton-associated protein 5	T777	91%	0.91	1	0	0	0	0	0	1	0	0	0	0	0	0	1
667	CKAP5_HUMAN	Cytoskeleton-associated protein 5	Y783	92%	0.92	1	0	0	0	0	0	1	0	0	0	0	0	0	1
668	CKAP5_HUMAN	Cytoskeleton-associated protein 5	Y785	61%	0.61	1	0	0	0	0	0	1	0	0	0	0	0	0	1
669	CRTAM_HUMAN	Cytotoxic and regulatory T-cell molecule	S7	50%	0.50	1	1	0	0	0	0	0	0	0	0	0	0	0	1

670	CRTAM_HUMAN	Cytotoxic and regulatory T-cell molecule	T27	50%	0.50	1	1	0	0	0	0	0	0	0	0	0	0	0	1
671	CRTAM_HUMAN	Cytotoxic and regulatory T-cell molecule	T33	50%	0.50	1	1	0	0	0	0	0	0	0	0	0	0	0	1
672	CRTAM_HUMAN	Cytotoxic and regulatory T-cell molecule	T35	50%	0.50	1	1	0	0	0	0	0	0	0	0	0	0	0	1
673	DACH2_HUMAN	Dachshund homolog 2	T246	12%	0.12	1	0	0	0	0	1	0	0	0	0	0	0	0	1
674	DCNL5_HUMAN	DCN1-like protein 5	S9	100%	1.00	35	3	0	1	1	1	1	2	1	2	0	0	0	12
675	DAXX_HUMAN	Death domain-associated protein 6	S668	85%	0.85	9	0	0	1	0	0	0	0	0	1	0	0	0	2
676	DAXX_HUMAN	Death domain-associated protein 6	S671	100%	1.00	68	1	1	2	0	0	0	3	0	0	0	0	0	7
677	DIDO1_HUMAN	Death-inducer obliterator 1	S1456	100%	1.00	230	4	4	6	2	8	2	2	2	0	3	3	0	36
678	DOCK5_HUMAN	Dedicator of cytokinesis protein 5	S1756	100%	1.00	5	0	0	0	0	0	0	0	0	1	0	0	1	2
679	DOCK6_HUMAN	Dedicator of cytokinesis protein 6	S42	15%	0.15	1	0	0	0	0	0	0	0	0	0	1	0	0	1
680	DOCK7_HUMAN	Dedicator of cytokinesis protein 7	S900	74%	0.74	7	2	0	0	0	0	0	0	0	0	0	0	0	2
681	DBC1_HUMAN	Deleted in bladder cancer protein 1	T134	48%	0.48	1	0	0	0	0	0	0	1	0	0	0	0	0	1
682	DBC1_HUMAN	Deleted in bladder cancer protein 1	Y125	89%	0.89	1	0	0	0	0	0	0	1	0	0	0	0	0	1
683	DBC1_HUMAN	Deleted in bladder cancer protein 1	Y144	99%	0.99	1	0	0	0	0	0	0	1	0	0	0	0	0	1
684	DEN4C_HUMAN	DENN domain-containing protein 4C	S1041	14%	0.14	9	0	0	0	0	1	0	0	0	0	0	0	0	1
685	DEN4C_HUMAN	DENN domain-containing protein 4C	S1042	100%	1.00	177	3	5	1	5	3	6	8	5	6	6	6	6	60
686	DEN4C_HUMAN	DENN domain-containing protein 4C	S890	33%	0.33	23	0	0	1	1	1	0	1	0	0	3	2	0	9
687	DENR_HUMAN	Density-regulated protein	S73	89%	0.89	97	3	1	0	4	0	1	2	0	0	0	0	1	12
688	DENR_HUMAN	Density-regulated protein	S81	23%	0.23	3	0	0	0	0	0	0	0	0	1	0	0	0	1

689	DENR_HUMAN	Density-regulated protein	T69	75%	0.75	29	2	0	0	0	1	0	1	0	0	0	0	0	4
690	TDIF2_HUMAN	Deoxynucleotidyltransferase terminal-interacting protein 2	S117	100%	1.00	10	0	0	0	0	0	0	0	0	0	1	1	1	3
691	DEPD5_HUMAN	DEP domain-containing protein 5	S1102	14%	0.14	1	0	0	0	1	0	0	0	0	0	0	0	0	1
692	DSG2_HUMAN	Desmoglein-2	S680	100%	1.00	11	0	0	0	0	0	0	0	0	0	4	4	0	8
693	DSG2_HUMAN	Desmoglein-2	T922	100%	1.00	2	0	1	0	0	1	0	0	0	0	0	0	0	2
694	DESP_HUMAN	Desmoplakin	S22	100%	1.00	2	0	0	0	0	0	0	0	0	0	0	1	1	2
695	DESP_HUMAN	Desmoplakin	S2549	80%	0.80	3	0	0	0	0	0	0	0	0	0	1	2	0	3
696	DESP_HUMAN	Desmoplakin	S2551	74%	0.74	15	0	0	0	0	0	0	0	0	0	3	2	6	11
697	DESP_HUMAN	Desmoplakin	S2606	74%	0.74	32	0	0	1	0	1	1	0	0	1	1	1	1	7
698	DESP_HUMAN	Desmoplakin	S2607	25%	0.25	124	0	1	0	4	4	2	0	2	0	13	10	6	42
699	DESP_HUMAN	Desmoplakin	S2608	78%	0.78	406	5	4	2	15	12	7	3	3	2	24	20	10	107
700	DESP_HUMAN	Desmoplakin	S2610	83%	0.83	218	2	3	2	7	4	7	4	3	2	8	8	6	56
701	DESP_HUMAN	Desmoplakin	S2616	31%	0.31	27	0	0	2	0	0	0	1	0	1	0	1	0	5
702	DESP_HUMAN	Desmoplakin	S2820	96%	0.96	46	2	1	4	1	2	0	1	0	1	7	5	4	28
703	DESP_HUMAN	Desmoplakin	S2821	37%	0.37	26	3	3	2	1	4	1	2	0	0	1	2	2	21
704	DESP_HUMAN	Desmoplakin	S2825	100%	1.00	72	3	4	5	2	6	7	0	1	3	5	4	5	45
705	DESP_HUMAN	Desmoplakin	T2612	64%	0.64	225	4	0	0	10	4	5	2	1	1	14	13	7	61
706	DEST_HUMAN	Destrin	S3	100%	1.00	121	9	13	6	5	6	4	0	1	2	0	0	0	46
707	DBLOH_HUMAN	Diablo homolog, mitochondrial	S67	26%	0.26	1	1	0	0	0	0	0	0	0	0	0	0	0	1
708	DBLOH_HUMAN	Diablo homolog, mitochondrial	S69	26%	0.26	1	1	0	0	0	0	0	0	0	0	0	0	0	1
709	DGKI_HUMAN	Diacylglycerol kinase iota	S57	14%	0.14	1	0	0	0	0	0	0	1	0	0	0	0	0	1
710	DYR_HUMAN	Dihydrofolate reductase	S60	100%	1.00	1	0	0	0	0	0	0	0	0	0	0	1	0	1
711	DPYL2_HUMAN	Dihydropyrimidinase-related protein 2	T509	100%	1.00	5	0	1	0	0	0	0	0	0	0	0	0	0	1
712	FMO4_HUMAN	Dimethylaniline monooxygenase [N-oxide-forming] 4	S376	66%	0.66	1	1	0	0	0	0	0	0	0	0	0	0	0	1

713	FMO4_HUMAN	Dimethylaniline monooxygenase [N-oxide-forming] 4	T381	87%	0.87	1	1	0	0	0	0	0	0	0	0	0	0	0	1
714	MVD1_HUMAN	Diphosphomevalonate decarboxylase	S96	78%	0.78	22	0	0	0	0	0	0	0	0	0	0	1	0	1
715	DPH5_HUMAN	Diphthine synthase	T39	32%	0.32	4	0	0	1	0	0	1	0	1	0	0	0	0	3
716	DPH5_HUMAN	Diphthine synthase	Y49	35%	0.35	4	0	0	1	0	0	1	0	1	0	0	0	0	3
717	DI3L2_HUMAN	DIS3-like exonuclease 2	S875	86%	0.86	3	0	1	0	0	0	0	1	0	0	0	0	0	2
718	DAB2P_HUMAN	Disabled homolog 2-interacting protein	S745	92%	0.92	1	0	0	0	0	0	0	1	0	0	0	0	0	1
719	DAB2P_HUMAN	Disabled homolog 2-interacting protein	S747	92%	0.92	1	0	0	0	0	0	0	1	0	0	0	0	0	1
720	ADA11_HUMAN	Disintegrin and metalloproteinase domain-containing protein 11	S126	11%	0.11	1	0	0	0	0	0	0	0	0	0	0	0	1	1
721	ADA15_HUMAN	Disintegrin and metalloproteinase domain-containing protein 15	S16	77%	0.77	1	0	0	0	1	0	0	0	0	0	0	0	0	1
722	ADA15_HUMAN	Disintegrin and metalloproteinase domain-containing protein 15	S20	68%	0.68	1	0	0	0	1	0	0	0	0	0	0	0	0	1
723	ADA15_HUMAN	Disintegrin and metalloproteinase domain-containing protein 15	T29	84%	0.84	1	0	0	0	1	0	0	0	0	0	0	0	0	1
724	ADA17_HUMAN	Disintegrin and metalloproteinase domain-containing protein 17	S791	100%	1.00	47	0	2	2	2	1	1	2	3	1	1	1	1	17
725	ADAM8_HUMAN	Disintegrin and metalloproteinase domain-containing protein 8	T730	14%	0.14	2	0	0	0	0	0	0	0	0	0	1	0	0	1
726	DNMT1_HUMAN	DNA (cytosine-5)-methyltransferase 1	S714	100%	1.00	23	0	1	0	1	1	0	0	0	0	0	0	0	3
727	DDB2_HUMAN	DNA damage-binding protein 2	S24	54%	0.54	1	0	0	1	0	0	0	0	0	0	0	0	0	1
728	DDB2_HUMAN	DNA damage-binding protein 2	S26	99%	0.99	1	0	0	0	0	0	1	0	0	0	0	0	0	1

729	COM1_HUMAN	DNA endonuclease RBBP8	S327	96%	0.96	2	0	1	0	0	0	0	0	0	0	0	0	0	1
730	COM1_HUMAN	DNA endonuclease RBBP8	T315	47%	0.47	2	0	0	0	2	0	0	0	0	0	0	0	0	2
731	ERC6L_HUMAN	DNA excision repair protein ERCC-6-like	S1028	80%	0.80	1	0	0	0	0	0	0	0	0	0	1	0	0	1
732	ERC6L_HUMAN	DNA excision repair protein ERCC-6-like	S820	100%	1.00	1	0	0	0	0	1	0	0	0	0	0	0	0	1
733	DNLI1_HUMAN	DNA ligase 1	S201	2%	0.02	1	0	0	1	0	0	0	0	0	0	0	0	0	1
734	DNLI1_HUMAN	DNA ligase 1	S66	100%	1.00	140	7	0	4	0	0	0	0	2	0	0	0	0	13
735	DNLI1_HUMAN	DNA ligase 1	S76	100%	1.00	352	7	5	7	6	6	6	3	6	6	6	6	4	68
736	DNLI1_HUMAN	DNA ligase 1	T195	100%	1.00	59	0	0	2	1	0	0	1	0	0	0	0	0	4
737	DNLI3_HUMAN	DNA ligase 3	S242	75%	0.75	6	2	0	0	0	0	0	0	0	0	0	1	0	3
738	DMAP1_HUMAN	DNA methyltransferase 1-associated protein 1	T445	100%	1.00	22	0	0	0	0	0	0	0	0	1	1	0	0	2
739	DPOLB_HUMAN	DNA polymerase beta	S94	14%	0.14	1	0	0	0	0	0	0	0	0	0	0	0	1	1
740	DPOD3_HUMAN	DNA polymerase delta subunit 3	S307	100%	1.00	25	0	2	3	1	0	0	0	0	0	0	0	0	6
741	POLH_HUMAN	DNA polymerase eta	S379	94%	0.94	2	0	1	0	0	0	0	0	0	0	0	0	1	2
742	POLH_HUMAN	DNA polymerase eta	S380	97%	0.97	1	0	0	0	0	0	0	0	0	0	1	0	0	1
743	POLH_HUMAN	DNA polymerase eta	S497	29%	0.29	3	0	1	0	0	0	0	0	0	1	0	0	0	2
744	POLH_HUMAN	DNA polymerase eta	S499	26%	0.26	6	0	1	0	0	0	1	0	0	1	0	0	0	3
745	POLH_HUMAN	DNA polymerase eta	S500	26%	0.26	10	0	1	0	0	0	1	0	1	1	0	1	0	5
746	POLH_HUMAN	DNA polymerase eta	S510	40%	0.40	2	0	0	0	0	0	0	0	0	0	0	0	1	1
747	POLH_HUMAN	DNA polymerase eta	T502	20%	0.20	8	0	0	0	0	0	1	0	1	0	0	1	1	4

748	POLH_HUMAN	DNA polymerase eta	T505	57%	0.57	6	0	0	0	0	0	0	0	1	0	0	1	1	3
749	DPOLN_HUMAN	DNA polymerase nu	T178	33%	0.33	1	0	0	0	0	0	1	0	0	0	0	0	0	1
750	DPOLN_HUMAN	DNA polymerase nu	Y184	33%	0.33	1	0	0	0	0	0	1	0	0	0	0	0	0	1
751	DPOLZ_HUMAN	DNA polymerase zeta catalytic subunit	S1272	25%	0.25	1	0	0	0	0	0	0	1	0	0	0	0	0	1
752	DPOLZ_HUMAN	DNA polymerase zeta catalytic subunit	S1302	25%	0.25	1	0	0	0	0	0	0	1	0	0	0	0	0	1
753	CDT1_HUMAN	DNA replication factor Cdt1	S491	33%	0.33	1	0	0	0	0	1	0	0	0	0	0	0	0	1
754	MCM2_HUMAN	DNA replication licensing factor MCM2	S108	100%	1.00	14	2	0	0	0	0	1	0	0	0	0	0	0	3
755	MCM2_HUMAN	DNA replication licensing factor MCM2	S13	100%	1.00	90	3	2	4	2	1	3	3	3	3	0	0	0	24
756	MCM2_HUMAN	DNA replication licensing factor MCM2	S139	100%	1.00	623	16	20	17	11	16	12	10	13	13	0	1	1	130
757	TOP2A_HUMAN	DNA topoisomerase 2-alpha	S1247	100%	1.00	23	1	1	0	0	0	0	0	0	0	4	1	0	7
758	TOP2A_HUMAN	DNA topoisomerase 2-alpha	S1392	4%	0.04	1	0	0	1	0	0	0	0	0	0	0	0	0	1
759	TOP2B_HUMAN	DNA topoisomerase 2-beta	S1581	79%	0.79	9	2	0	0	0	0	0	0	0	0	0	0	0	2
760	DBPA_HUMAN	DNA-binding protein A	S134	100%	1.00	57	0	0	0	2	0	0	1	0	0	6	6	6	21
761	DBPA_HUMAN	DNA-binding protein A	S34	93%	0.93	484	6	5	7	14	7	8	7	5	9	9	9	12	98
762	DBPA_HUMAN	DNA-binding protein A	S38	80%	0.80	761	9	7	13	15	10	11	9	4	9	12	10	12	121
763	DBPA_HUMAN	DNA-binding protein A	S72	18%	0.18	2	0	0	0	0	1	0	0	0	0	0	0	0	1
764	PRKDC_HUMAN	DNA-dependent protein kinase catalytic subunit	S3481	25%	0.25	1	0	0	0	0	0	0	0	0	1	0	0	0	1
765	PRKDC_HUMAN	DNA-dependent protein kinase catalytic subunit	S893	100%	1.00	2	0	2	0	0	0	0	0	0	0	0	0	0	2
766	PRKDC_HUMAN	DNA-dependent protein kinase catalytic subunit	T3484	25%	0.25	1	0	0	0	0	0	0	0	0	1	0	0	0	1

767	RPA34_HUMAN	DNA-directed RNA polymerase I subunit RPA34	S126	69%	0.69	1	1	0	0	0	0	0	0	0	0	0	0	0	1
768	RPA34_HUMAN	DNA-directed RNA polymerase I subunit RPA34	S128	97%	0.97	22	1	2	2	2	0	3	0	0	0	0	0	0	10
769	RPA34_HUMAN	DNA-directed RNA polymerase I subunit RPA34	S136	100%	1.00	291	4	8	1	9	4	7	2	4	4	6	3	2	54
770	DNJA1_HUMAN	DnaJ homolog subfamily A member 1	S335	100%	1.00	43	0	0	0	2	0	0	1	0	1	1	0	0	5
771	DNJA1_HUMAN	DnaJ homolog subfamily A member 1	T252	100%	1.00	59	0	0	0	0	0	1	1	0	0	0	0	0	2
772	DNJB6_HUMAN	DnaJ homolog subfamily B member 6	S277	100%	1.00	4	0	1	0	0	0	0	0	0	1	0	0	0	2
773	DJC28_HUMAN	DnaJ homolog subfamily C member 28	S234	60%	0.60	11	0	0	0	0	0	0	1	0	0	0	0	0	1
774	DJC28_HUMAN	DnaJ homolog subfamily C member 28	T240	33%	0.33	1	0	0	0	0	0	0	1	0	0	0	0	0	1
775	DNJC5_HUMAN	DnaJ homolog subfamily C member 5	S10	98%	0.98	260	11	7	3	4	7	4	4	3	4	5	4	1	57
776	DNJC5_HUMAN	DnaJ homolog subfamily C member 5	S8	95%	0.95	11	0	1	0	0	0	1	0	1	0	0	0	0	3
777	DNJC5_HUMAN	DnaJ homolog subfamily C member 5	T11	17%	0.17	5	0	0	0	1	0	0	0	0	0	0	0	0	1
778	RPN1_HUMAN	Dolichyl-diphosphooligosaccharide--protein glycosyltransferase subunit 1	S275	52%	0.52	1	0	0	0	0	0	0	0	0	0	0	0	1	1
779	RPN1_HUMAN	Dolichyl-diphosphooligosaccharide--protein glycosyltransferase subunit 1	S276	52%	0.52	1	0	0	0	0	0	0	0	0	0	0	0	1	1
780	DREB_HUMAN	Drebrin	S141	3%	0.03	1	0	0	0	0	0	0	0	0	0	0	0	1	1
781	DREB_HUMAN	Drebrin	S142	100%	1.00	6	0	0	0	0	0	0	0	0	0	2	2	2	6
782	DREB_HUMAN	Drebrin	S337	97%	0.97	254	6	5	5	3	7	2	7	2	7	8	7	6	65

783	DREB_HUMAN	Drebrin	S339	90%	0.90	134	0	4	3	5	2	4	5	3	0	7	7	7	47
784	DREB_HUMAN	Drebrin	S342	15%	0.15	7	1	0	0	1	0	0	0	0	1	2	0	0	5
785	DREB_HUMAN	Drebrin	S345	2%	0.02	1	0	0	0	0	0	0	0	0	0	1	0	0	1
786	DREB_HUMAN	Drebrin	T331	11%	0.11	5	0	0	0	0	0	0	0	0	0	1	0	0	1
787	DREB_HUMAN	Drebrin	T335	65%	0.65	65	2	0	2	1	0	3	0	4	2	3	2	2	21
788	DREB_HUMAN	Drebrin	T343	35%	0.35	7	0	1	1	0	1	0	0	0	0	1	0	0	4
789	DBNL_HUMAN	Drebrin-like protein	T45	16%	0.16	1	0	0	1	0	0	0	0	0	0	0	0	0	1
790	CLK4_HUMAN	Dual specificity protein kinase CLK4	S136	100%	1.00	4	0	0	0	0	0	0	0	1	0	0	0	0	1
791	CLK4_HUMAN	Dual specificity protein kinase CLK4	S138	100%	1.00	5	0	0	0	0	0	0	1	1	0	0	0	0	2
792	DUS7_HUMAN	Dual specificity protein phosphatase 7	S369	100%	1.00	11	0	0	0	0	0	0	0	0	0	2	2	0	4
793	DYN2_HUMAN	Dynamin-2	S764	8%	0.08	2	0	1	0	0	0	0	0	0	0	0	0	0	1
794	DYN2_HUMAN	Dynamin-2	T766	8%	0.08	1	0	1	0	0	0	0	0	0	0	0	0	0	1
795	DYH10_HUMAN	Dynein heavy chain 10, axonemal	S1578	93%	0.93	1	0	0	0	0	0	1	0	0	0	0	0	0	1
796	DYH10_HUMAN	Dynein heavy chain 10, axonemal	S1583	79%	0.79	1	0	0	0	0	0	1	0	0	0	0	0	0	1
797	DYH14_HUMAN	Dynein heavy chain 14, axonemal	T3364	45%	0.45	5	0	1	0	0	0	0	0	0	1	0	0	0	2
798	DYH14_HUMAN	Dynein heavy chain 14, axonemal	T3365	40%	0.40	5	0	1	0	0	0	0	0	0	1	0	0	0	2
799	DAG1_HUMAN	Dystroglycan	S14	100%	1.00	1	0	0	0	0	0	0	0	0	0	0	1	0	1
800	DAG1_HUMAN	Dystroglycan	S4	100%	1.00	1	0	0	0	0	0	0	0	0	0	0	1	0	1
801	DAG1_HUMAN	Dystroglycan	S8	100%	1.00	1	0	0	0	0	0	0	0	0	0	0	1	0	1
802	RBP2_HUMAN	E3 SUMO-protein ligase RanBP2	S1160	100%	1.00	25	1	2	0	0	0	0	0	1	0	0	0	0	4

803	RBP2_HUMAN	E3 SUMO-protein ligase RanBP2	S1509	100%	1.00	50	2	2	0	0	0	0	0	0	0	0	1	0	5
804	RBP2_HUMAN	E3 SUMO-protein ligase RanBP2	S2510	100%	1.00	49	3	3	2	1	2	1	2	0	0	3	1	2	20
805	RBP2_HUMAN	E3 SUMO-protein ligase RanBP2	S2741	100%	1.00	32	2	3	2	1	1	2	1	1	3	0	0	0	16
806	RBP2_HUMAN	E3 SUMO-protein ligase RanBP2	S2900	100%	1.00	599	10	8	6	10	9	8	8	3	8	9	9	7	95
807	RBP2_HUMAN	E3 SUMO-protein ligase RanBP2	S955	100%	1.00	7	0	0	0	0	0	0	0	0	0	2	0	0	2
808	RBP2_HUMAN	E3 SUMO-protein ligase RanBP2	T1396	100%	1.00	28	0	0	1	1	0	0	0	0	0	0	0	0	2
809	RBP2_HUMAN	E3 SUMO-protein ligase RanBP2	T1513	1%	0.01	1	1	0	0	0	0	0	0	0	0	0	0	0	1
810	RNF4_HUMAN	E3 ubiquitin ligase RNF4	S94	100%	1.00	10	0	1	2	0	0	0	0	0	0	0	0	0	3
811	RNF4_HUMAN	E3 ubiquitin ligase RNF4	S95	100%	1.00	10	0	1	2	0	0	0	0	0	0	0	0	0	3
812	BRE1A_HUMAN	E3 ubiquitin-protein ligase BRE1A	S138	100%	1.00	55	3	3	3	3	2	1	3	4	3	2	0	1	28
813	CBL_HUMAN	E3 ubiquitin-protein ligase CBL	S486	86%	0.86	1	0	1	0	0	0	0	0	0	0	0	0	0	1
814	CHIP_HUMAN	E3 ubiquitin-protein ligase CHIP	S19	97%	0.97	9	0	0	0	0	1	0	0	0	0	0	0	0	1
815	CHIP_HUMAN	E3 ubiquitin-protein ligase CHIP	S23	99%	0.99	5	0	0	0	0	0	0	1	1	0	0	0	0	2
816	DTX3L_HUMAN	E3 ubiquitin-protein ligase DTX3L	S9	100%	1.00	183	4	3	2	5	4	3	5	4	6	6	4	4	50
817	HERC2_HUMAN	E3 ubiquitin-protein ligase HERC2	S2454	100%	1.00	28	0	5	0	0	0	0	1	1	0	0	0	0	7
818	HUWE1_HUMAN	E3 ubiquitin-protein ligase HUWE1	S1907	100%	1.00	142	4	5	4	6	4	5	6	6	6	4	2	3	55
819	HUWE1_HUMAN	E3 ubiquitin-protein ligase HUWE1	S2953	10%	0.10	1	0	0	0	0	0	0	0	0	0	0	1	0	1
820	HUWE1_HUMAN	E3 ubiquitin-protein ligase HUWE1	S3816	65%	0.65	6	1	1	0	0	1	0	0	0	0	0	0	0	3
821	HUWE1_HUMAN	E3 ubiquitin-protein ligase HUWE1	S3818	23%	0.23	2	0	0	0	0	1	0	0	0	0	0	0	0	1
822	HUWE1_HUMAN	E3 ubiquitin-protein ligase HUWE1	S3919	100%	1.00	58	1	1	0	1	1	0	2	0	1	4	1	1	13
823	HUWE1_HUMAN	E3 ubiquitin-protein ligase HUWE1	S649	20%	0.20	71	2	1	2	0	1	0	1	1	1	1	1	0	11

824	LTN1_HUMAN	E3 ubiquitin-protein ligase listerin	T1543	100%	1.00	1	0	0	1	0	0	0	0	0	0	0	0	0	1
825	NEDD4_HUMAN	E3 ubiquitin-protein ligase NEDD4	S747	78%	0.78	2	0	1	0	0	0	0	0	0	0	0	0	0	1
826	NED4L_HUMAN	E3 ubiquitin-protein ligase NEDD4-like	S446	86%	0.86	57	2	3	0	1	1	1	2	0	1	1	1	4	17
827	NED4L_HUMAN	E3 ubiquitin-protein ligase NEDD4-like	S448	90%	0.90	434	8	10	14	7	7	5	5	7	5	6	8	4	86
828	RBBP6_HUMAN	E3 ubiquitin-protein ligase RBBP6	S1179	100%	1.00	115	5	5	6	4	2	4	2	1	2	6	6	4	47
829	RBBP6_HUMAN	E3 ubiquitin-protein ligase RBBP6	S1328	100%	1.00	4	1	0	0	1	0	0	0	0	0	0	0	0	2
830	RBBP6_HUMAN	E3 ubiquitin-protein ligase RBBP6	T1173	0%	0.00	1	0	0	0	0	0	0	0	0	0	0	0	1	1
831	RFFL_HUMAN	E3 ubiquitin-protein ligase rififylin	S240	100%	1.00	115	0	0	2	0	2	0	0	0	1	4	7	2	18
832	RFFL_HUMAN	E3 ubiquitin-protein ligase rififylin	S242	87%	0.87	15	0	0	0	1	0	0	0	0	0	1	2	2	6
833	RFFL_HUMAN	E3 ubiquitin-protein ligase rififylin	T245	93%	0.93	13	0	0	0	0	0	0	0	0	1	1	2	0	4
834	RN167_HUMAN	E3 ubiquitin-protein ligase RNF167	T316	16%	0.16	1	0	1	0	0	0	0	0	0	0	0	0	0	1
835	TRI33_HUMAN	E3 ubiquitin-protein ligase TRIM33	S1105	100%	1.00	4	0	0	0	0	0	0	0	0	1	0	0	0	1
836	TRI33_HUMAN	E3 ubiquitin-protein ligase TRIM33	T1085	33%	0.33	1	0	0	0	0	0	0	0	0	1	0	0	0	1
837	UBR4_HUMAN	E3 ubiquitin-protein ligase UBR4	S619	100%	1.00	6	0	1	1	0	0	0	1	2	0	1	0	0	6
838	UBR4_HUMAN	E3 ubiquitin-protein ligase UBR4	S620	100%	1.00	37	3	1	2	2	3	3	3	2	2	5	5	5	36
839	UBR5_HUMAN	E3 ubiquitin-protein ligase UBR5	S2026	100%	1.00	7	1	1	0	0	0	0	0	0	1	0	0	0	3
840	UBR5_HUMAN	E3 ubiquitin-protein ligase UBR5	S2028	100%	1.00	10	1	3	0	0	0	0	1	1	0	0	0	0	6
841	UBR5_HUMAN	E3 ubiquitin-protein ligase UBR5	T2030	100%	1.00	7	2	2	0	0	0	0	1	0	0	0	0	0	5
842	UHRF1_HUMAN	E3 ubiquitin-protein ligase UHRF1	S287	100%	1.00	2	0	0	2	0	0	0	0	0	0	0	0	0	2

843	EMAL4_HUMAN	Echinoderm microtubule-associated protein-like 4	S144	67%	0.67	2	0	0	0	0	0	0	1	0	0	0	1	0	2
844	EMAL4_HUMAN	Echinoderm microtubule-associated protein-like 4	S146	27%	0.27	1	0	0	0	0	0	1	0	0	0	0	0	0	1
845	ENPP2_HUMAN	Ectonucleotide pyrophosphatase/phosphodiesterase family member 2	S687	29%	0.29	1	0	0	0	0	0	0	0	0	0	0	0	1	1
846	ENPP2_HUMAN	Ectonucleotide pyrophosphatase/phosphodiesterase family member 2	S689	29%	0.29	1	0	0	0	0	0	0	0	0	0	0	0	1	1
847	ENPP2_HUMAN	Ectonucleotide pyrophosphatase/phosphodiesterase family member 2	T700	69%	0.69	1	0	0	0	0	0	0	0	0	0	0	0	1	1
848	EHBP1_HUMAN	EH domain-binding protein 1	S295	75%	0.75	3	0	0	0	1	0	0	0	0	1	0	0	0	2
849	EHBP1_HUMAN	EH domain-binding protein 1	S335	24%	0.24	2	1	0	0	1	0	0	0	0	0	0	0	0	2
850	EH1L1_HUMAN	EH domain-binding protein 1-like protein 1	S1257	100%	1.00	43	0	0	0	0	0	0	0	0	0	9	4	5	18
851	EH1L1_HUMAN	EH domain-binding protein 1-like protein 1	S285	50%	0.50	13	0	0	0	0	0	0	1	2	0	0	3	1	7
852	EH1L1_HUMAN	EH domain-binding protein 1-like protein 1	S310	100%	1.00	12	0	0	0	0	0	0	0	0	0	2	0	1	3
853	EH1L1_HUMAN	EH domain-binding protein 1-like protein 1	T284	100%	1.00	14	0	0	0	0	0	1	0	0	0	3	0	2	6
854	EHD2_HUMAN	EH domain-containing protein 2	S438	100%	1.00	27	2	0	0	0	2	0	0	0	2	2	0	0	8
855	ELAV1_HUMAN	ELAV-like protein 1	S202	100%	1.00	13	0	0	0	0	0	0	0	0	0	1	2	2	5
856	EF1B_HUMAN	Elongation factor 1-beta	S106	100%	1.00	278	5	4	6	4	7	5	4	4	5	0	4	0	48
857	EF1B_HUMAN	Elongation factor 1-beta	S112	54%	0.54	20	1	2	0	2	0	1	0	1	0	0	0	0	7
858	EF1D_HUMAN	Elongation factor 1-delta	S133	99%	0.99	4	0	0	0	0	0	0	0	0	0	2	1	1	4
859	EF1D_HUMAN	Elongation factor 1-delta	S162	100%	1.00	####	26	25	31	26	27	31	20	21	20	27	22	19	295
860	EF1D_HUMAN	Elongation factor 1-delta	T125	0%	0.00	1	0	0	0	0	0	0	0	0	0	0	0	1	1
861	EF1D_HUMAN	Elongation factor 1-delta	T129	7%	0.07	1	0	0	0	0	0	0	0	0	0	0	1	0	1

862	EF1D_HUMAN	Elongation factor 1-delta	T147	100%	1.00	30	1	0	0	0	0	0	0	0	0	1	0	0	2
863	EFTS_HUMAN	Elongation factor Ts, mitochondrial	S6	100%	1.00	1	0	0	0	0	0	0	0	0	0	0	0	1	1
864	ELOV2_HUMAN	Elongation of very long chain fatty acids protein 2	S24	100%	1.00	1	0	0	1	0	0	0	0	0	0	0	0	0	1
865	ELP2_HUMAN	Elongator complex protein 2	S122	13%	0.13	1	0	0	0	0	0	0	0	0	0	0	1	0	1
866	EVL_HUMAN	Ena/VASP-like protein	S102	14%	0.14	1	0	0	0	0	0	0	0	0	0	1	0	0	1
867	EVL_HUMAN	Ena/VASP-like protein	S296	61%	0.61	1	0	0	0	0	0	0	1	0	0	0	0	0	1
868	EVL_HUMAN	Ena/VASP-like protein	S319	16%	0.16	1	0	0	0	0	0	0	1	0	0	0	0	0	1
869	EDC4_HUMAN	Enhancer of mRNA-decapping protein 4	S723	98%	0.98	371	3	4	5	1	4	2	3	4	2	12	7	5	52
870	EDC4_HUMAN	Enhancer of mRNA-decapping protein 4	S725	99%	0.99	244	3	4	5	4	0	1	2	6	2	5	2	3	37
871	EDC4_HUMAN	Enhancer of mRNA-decapping protein 4	S729	100%	1.00	308	8	7	7	7	8	4	5	3	6	8	7	6	76
872	EDC4_HUMAN	Enhancer of mRNA-decapping protein 4	S735	12%	0.13	8	2	0	0	0	0	1	0	0	0	0	0	0	3
873	EDC4_HUMAN	Enhancer of mRNA-decapping protein 4	S737	22%	0.22	8	0	0	0	1	0	0	0	0	0	0	1	0	2
874	EDC4_HUMAN	Enhancer of mRNA-decapping protein 4	S875	22%	0.22	2	1	0	0	0	0	0	0	0	0	0	0	0	1
875	EDC4_HUMAN	Enhancer of mRNA-decapping protein 4	S879	34%	0.34	4	2	0	0	0	0	1	0	0	0	0	1	0	4
876	EDC4_HUMAN	Enhancer of mRNA-decapping protein 4	T727	57%	0.57	11	0	0	1	0	0	0	1	0	0	0	0	0	2
877	EDC4_HUMAN	Enhancer of mRNA-decapping protein 4	T738	13%	0.13	28	2	0	0	0	1	1	0	0	0	0	1	0	5
878	MAP7_HUMAN	Ensconsin	S209	100%	1.00	4	0	0	0	0	0	0	0	0	0	0	0	2	2
879	EPHA2_HUMAN	Ephrin type-A receptor 2	S897	100%	1.00	108	0	0	0	0	1	0	0	0	0	11	9	8	29
880	EPHA2_HUMAN	Ephrin type-A receptor 2	S901	38%	0.38	29	0	0	0	0	0	0	0	1	0	1	2	2	6
881	EPHA5_HUMAN	Ephrin type-A receptor 5	S534	24%	0.24	1	0	0	0	0	0	0	1	0	0	0	0	0	1

882	EPHA5_HUMAN	Ephrin type-A receptor 5	Y536	77%	0.77	1	0	0	0	0	0	0	1	0	0	0	0	0	1
883	EGFR_HUMAN	Epidermal growth factor receptor	T693	100%	1.00	134	0	0	0	4	3	5	0	0	1	5	5	3	26
884	ES8L1_HUMAN	Epidermal growth factor receptor kinase substrate 8-like protein 1	S631	92%	0.92	2	0	0	0	0	0	0	0	0	0	0	0	2	2
885	ES8L2_HUMAN	Epidermal growth factor receptor kinase substrate 8-like protein 2	S449	99%	0.99	6	0	0	0	0	0	0	0	0	0	0	0	1	1
886	ES8L2_HUMAN	Epidermal growth factor receptor kinase substrate 8-like protein 2	S452	12%	0.12	7	0	0	0	0	0	0	0	0	0	1	4	0	5
887	ES8L2_HUMAN	Epidermal growth factor receptor kinase substrate 8-like protein 2	S459	57%	0.57	5	0	0	0	0	0	0	0	0	0	0	1	1	2
888	ES8L2_HUMAN	Epidermal growth factor receptor kinase substrate 8-like protein 2	S479	97%	0.97	28	0	1	0	1	0	1	1	0	0	1	5	1	11
889	ES8L2_HUMAN	Epidermal growth factor receptor kinase substrate 8-like protein 2	S480	68%	0.68	34	0	1	1	2	1	1	1	1	0	1	1	1	11
890	ES8L2_HUMAN	Epidermal growth factor receptor kinase substrate 8-like protein 2	S570	96%	0.96	1	0	0	0	0	0	0	0	0	0	0	1	0	1
891	ES8L2_HUMAN	Epidermal growth factor receptor kinase substrate 8-like protein 2	T465	15%	0.15	9	2	1	0	0	0	0	0	0	0	1	0	0	4
892	ES8L2_HUMAN	Epidermal growth factor receptor kinase substrate 8-like protein 2	T483	40%	0.40	5	0	0	0	0	0	0	0	0	0	0	1	2	3
893	EPS15_HUMAN	Epidermal growth factor receptor substrate 15	S323	97%	0.97	2	0	0	0	0	0	0	0	0	0	1	0	1	2
894	EPS15_HUMAN	Epidermal growth factor receptor substrate 15	S324	98%	0.98	4	0	0	0	0	0	0	0	0	0	2	1	1	4
895	EPIPL_HUMAN	Epiplakin	S2716	100%	1.00	118	0	0	1	1	2	2	3	1	2	6	10	4	32
896	EPIPL_HUMAN	Epiplakin	S2718	82%	0.82	21	0	0	0	0	0	0	0	0	0	4	3	0	7
897	EPN1_HUMAN	Epsin-1	S435	100%	1.00	21	1	0	0	1	2	1	0	0	0	1	0	1	7

898	EPN1_HUMAN	Epsin-1	S473	99%	0.99	113	4	4	5	4	2	3	6	6	4	0	0	1	39
899	EPN1_HUMAN	Epsin-1	T470	100%	1.00	453	6	9	11	11	9	9	13	12	12	8	8	7	115
900	EPN3_HUMAN	Epsin-3	T495	87%	0.87	1	0	0	1	0	0	0	0	0	0	0	0	0	1
901	ERRFI_HUMAN	ERBB receptor feedback inhibitor 1	S273	98%	0.98	1	0	0	0	0	0	0	0	0	0	1	0	0	1
902	ELK1_HUMAN	ETS domain-containing protein Elk-1	S324	100%	1.00	31	1	1	2	4	0	2	2	1	0	2	5	1	21
903	ELK1_HUMAN	ETS domain-containing protein Elk-1	S326	100%	1.00	1	0	0	0	0	0	0	0	0	0	0	1	0	1
904	ERF_HUMAN	ETS domain-containing transcription factor ERF	T526	100%	1.00	1	0	0	0	0	1	0	0	0	0	0	0	0	1
905	IF4A2_HUMAN	Eukaryotic initiation factor 4A-II	S2	75%	0.75	1	0	0	0	1	0	0	0	0	0	0	0	0	1
906	IF4A2_HUMAN	Eukaryotic initiation factor 4A-II	S5	83%	0.83	1	0	0	0	1	0	0	0	0	0	0	0	0	1
907	IF4A2_HUMAN	Eukaryotic initiation factor 4A-II	Y8	60%	0.60	1	0	0	0	1	0	0	0	0	0	0	0	0	1
908	IF2B_HUMAN	Eukaryotic translation initiation factor 2 subunit 2	S2	100%	1.00	####	37	47	36	40	36	41	31	34	25	28	24	21	400
909	IF2B_HUMAN	Eukaryotic translation initiation factor 2 subunit 2	T111	100%	1.00	5	1	2	0	0	0	0	0	0	0	0	0	0	3
910	IF2BL_HUMAN	Eukaryotic translation initiation factor 2 subunit 2-like protein	S2	100%	1.00	####	37	47	36	40	36	41	31	34	25	28	24	21	400
911	IF2BL_HUMAN	Eukaryotic translation initiation factor 2 subunit 2-like protein	T111	100%	1.00	5	1	2	0	0	0	0	0	0	0	0	0	0	3
912	EIF2A_HUMAN	Eukaryotic translation initiation factor 2A	S503	20%	0.20	2	0	0	0	0	0	0	0	0	1	0	0	0	1
913	EIF2A_HUMAN	Eukaryotic translation initiation factor 2A	S506	71%	0.71	20	0	0	0	0	1	1	0	0	0	3	1	2	8
914	E2AK4_HUMAN	Eukaryotic translation initiation factor 2-alpha kinase 4	T667	100%	1.00	115	4	6	6	6	6	6	8	5	6	3	3	4	63
915	EIF3B_HUMAN	Eukaryotic translation initiation factor 3 subunit B	S152	100%	1.00	484	11	15	14	8	15	14	10	9	10	9	9	4	128

916	EIF3B_HUMAN	Eukaryotic translation initiation factor 3 subunit B	S154	100%	1.00	####	26	24	27	27	29	26	24	19	22	19	19	9	271
917	EIF3B_HUMAN	Eukaryotic translation initiation factor 3 subunit B	S164	100%	1.00	380	10	10	13	9	12	12	7	4	5	5	5	1	93
918	EIF3B_HUMAN	Eukaryotic translation initiation factor 3 subunit B	S83	99%	0.99	35	0	0	0	0	2	1	0	0	1	0	0	0	4
919	EIF3B_HUMAN	Eukaryotic translation initiation factor 3 subunit B	S85	85%	0.85	18	0	0	0	0	0	2	0	0	1	0	0	0	3
920	EIF3C_HUMAN	Eukaryotic translation initiation factor 3 subunit C	S39	100%	1.00	39	1	2	2	1	1	2	2	0	3	0	1	1	16
921	EIF3G_HUMAN	Eukaryotic translation initiation factor 3 subunit G	S42	100%	1.00	939	24	13	17	18	17	16	16	18	14	23	18	19	213
922	EIF3G_HUMAN	Eukaryotic translation initiation factor 3 subunit G	T38	62%	0.62	16	0	2	0	0	0	0	0	0	0	0	0	0	2
923	EIF3G_HUMAN	Eukaryotic translation initiation factor 3 subunit G	T41	100%	1.00	304	8	9	10	10	7	6	7	6	9	7	8	8	95
924	EIF3J_HUMAN	Eukaryotic translation initiation factor 3 subunit J	S11	100%	1.00	223	11	13	11	9	10	3	4	7	9	3	5	2	87
925	EIF3J_HUMAN	Eukaryotic translation initiation factor 3 subunit J	S13	100%	1.00	105	8	2	1	1	3	1	2	2	0	2	1	1	24
926	EIF3K_HUMAN	Eukaryotic translation initiation factor 3 subunit K	S217	100%	1.00	2	0	0	1	0	0	1	0	0	0	0	0	0	2
927	IF4G1_HUMAN	Eukaryotic translation initiation factor 4 gamma 1	S1185	100%	1.00	9	1	0	0	0	1	0	0	0	0	0	2	0	4
928	IF4G1_HUMAN	Eukaryotic translation initiation factor 4 gamma 1	S1187	100%	1.00	38	0	4	3	0	1	1	1	1	1	2	1	2	17
929	IF4G1_HUMAN	Eukaryotic translation initiation factor 4 gamma 1	S1209	90%	0.90	1	0	0	1	0	0	0	0	0	0	0	0	0	1
930	IF4G1_HUMAN	Eukaryotic translation initiation factor 4 gamma 1	S1231	100%	1.00	237	2	4	4	7	8	6	6	3	4	10	11	11	76
931	IF4G2_HUMAN	Eukaryotic translation initiation factor 4 gamma 2	S395	92%	0.92	13	0	1	0	0	0	0	0	0	1	1	3	2	8
932	IF4G2_HUMAN	Eukaryotic translation initiation factor 4 gamma 2	T397	19%	0.19	4	0	1	0	0	0	0	0	0	0	0	1	1	3

933	IF4G2_HUMAN	Eukaryotic translation initiation factor 4 gamma 2	T508	97%	0.97	4	1	0	0	0	1	0	0	0	0	0	1	0	3
934	IF4G3_HUMAN	Eukaryotic translation initiation factor 4 gamma 3	S495	100%	1.00	237	1	3	4	2	1	5	6	5	3	2	0	1	33
935	4ET_HUMAN	Eukaryotic translation initiation factor 4E transporter	S564	100%	1.00	16	0	1	2	1	1	0	1	0	0	0	0	0	6
936	4ET_HUMAN	Eukaryotic translation initiation factor 4E transporter	S587	100%	1.00	79	5	11	5	2	2	2	2	6	4	4	5	3	51
937	4EBP1_HUMAN	Eukaryotic translation initiation factor 4E-binding protein 1	S35	71%	0.71	66	10	3	3	3	4	5	6	2	5	3	2	1	47
938	4EBP1_HUMAN	Eukaryotic translation initiation factor 4E-binding protein 1	S44	63%	0.63	152	9	10	8	9	5	7	8	12	9	4	10	1	92
939	4EBP1_HUMAN	Eukaryotic translation initiation factor 4E-binding protein 1	S65	100%	1.00	3	0	0	0	0	0	0	0	0	0	2	0	1	3
940	4EBP1_HUMAN	Eukaryotic translation initiation factor 4E-binding protein 1	S86	9%	0.09	1	0	0	0	0	0	0	0	0	0	1	0	0	1
941	4EBP1_HUMAN	Eukaryotic translation initiation factor 4E-binding protein 1	T37	99%	0.99	150	2	0	2	1	4	1	3	0	3	4	2	3	25
942	4EBP1_HUMAN	Eukaryotic translation initiation factor 4E-binding protein 1	T41	97%	0.97	123	4	5	5	7	6	5	8	6	7	8	4	3	68
943	4EBP1_HUMAN	Eukaryotic translation initiation factor 4E-binding protein 1	T45	77%	0.77	149	10	8	10	7	8	7	5	10	8	3	10	4	90
944	4EBP1_HUMAN	Eukaryotic translation initiation factor 4E-binding protein 1	T46	98%	0.98	119	7	5	6	5	9	7	5	9	5	3	4	6	71
945	4EBP1_HUMAN	Eukaryotic translation initiation factor 4E-binding protein 1	T50	22%	0.22	66	11	3	3	3	4	5	7	2	5	3	3	3	52
946	4EBP1_HUMAN	Eukaryotic translation initiation factor 4E-binding protein 1	T70	98%	0.98	150	10	6	9	11	9	9	10	10	10	4	5	2	95

947	4EBP1_HUMAN	Eukaryotic translation initiation factor 4E-binding protein 1	T77	82%	0.82	16	0	0	0	2	1	2	2	2	1	0	0	0	10
948	4EBP1_HUMAN	Eukaryotic translation initiation factor 4E-binding protein 1	Y34	17%	0.17	17	1	0	1	1	3	1	1	3	0	0	1	1	13
949	IF2P_HUMAN	Eukaryotic translation initiation factor 5B	S135	100%	1.00	33	1	1	0	0	0	1	2	2	1	0	2	0	10
950	IF2P_HUMAN	Eukaryotic translation initiation factor 5B	S137	100%	1.00	44	4	1	0	0	1	1	2	3	1	0	3	0	16
951	IF2P_HUMAN	Eukaryotic translation initiation factor 5B	S214	100%	1.00	####	35	40	31	41	30	31	36	33	25	32	30	26	390
952	IF2P_HUMAN	Eukaryotic translation initiation factor 5B	S222	0%	0.00	11	1	0	2	0	0	1	0	0	1	0	0	0	5
953	IF2P_HUMAN	Eukaryotic translation initiation factor 5B	Y134	67%	0.67	10	3	0	0	0	1	0	0	0	0	0	0	0	4
954	IF6_HUMAN	Eukaryotic translation initiation factor 6	S235	61%	0.61	6	0	0	0	0	0	0	0	0	0	0	1	0	1
955	IF6_HUMAN	Eukaryotic translation initiation factor 6	S239	70%	0.70	37	0	0	0	0	2	0	0	0	0	0	1	0	3
956	EAA2_HUMAN	Excitatory amino acid transporter 2	S106	100%	1.00	18	1	1	0	0	0	1	0	0	0	1	0	0	4
957	EAA2_HUMAN	Excitatory amino acid transporter 2	T103	100%	1.00	18	1	1	0	0	0	1	0	0	0	1	0	0	4
958	XPO1_HUMAN	Exportin-1	S391	80%	0.80	2	0	0	0	0	0	0	0	0	0	1	1	0	2
959	XPO6_HUMAN	Exportin-6	S208	39%	0.39	3	0	0	0	0	0	1	0	0	0	0	0	0	1
960	XPO6_HUMAN	Exportin-6	T204	42%	0.42	3	0	0	0	0	0	1	0	0	0	0	0	0	1
961	ESYT2_HUMAN	Extended synaptotagmin-2	S758	100%	1.00	17	0	0	0	0	0	0	0	1	0	2	0	0	3
962	FRAS1_HUMAN	Extracellular matrix protein FRAS1	S1970	89%	0.89	12	0	1	0	0	0	0	0	1	0	2	0	0	4
963	FRAS1_HUMAN	Extracellular matrix protein FRAS1	S1971	62%	0.62	15	1	1	0	0	0	0	0	1	0	2	0	0	5
964	FRAS1_HUMAN	Extracellular matrix protein FRAS1	S1976	79%	0.79	5	1	0	0	0	0	1	0	0	0	0	0	0	2
965	FRAS1_HUMAN	Extracellular matrix protein FRAS1	S1978	18%	0.18	2	0	0	0	0	0	1	0	0	0	0	0	0	1

966	CAZA2_HUMAN	F-actin-capping protein subunit alpha-2	S9	100%	1.00	30	3	0	1	2	1	2	2	0	1	1	1	3	17
967	FUBP2_HUMAN	Far upstream element-binding protein 2	S181	100%	1.00	7	0	0	0	0	0	0	0	0	0	0	1	4	5
968	FPPS_HUMAN	Farnesyl pyrophosphate synthase	S64	69%	0.69	3	0	0	0	0	0	0	0	0	0	0	1	0	1
969	FAF1_HUMAN	FAS-associated factor 1	S320	100%	1.00	5	1	0	1	0	0	1	1	0	0	0	0	0	4
970	FAF1_HUMAN	FAS-associated factor 1	S582	100%	1.00	19	0	0	0	0	0	0	0	0	0	3	2	2	7
971	FAS_HUMAN	Fatty acid synthase	S1411	100%	1.00	18	0	0	0	0	0	0	0	0	0	2	1	1	4
972	FAS_HUMAN	Fatty acid synthase	S207	100%	1.00	34	0	0	0	0	0	0	0	0	0	4	4	3	11
973	FAS_HUMAN	Fatty acid synthase	S2236	84%	0.84	7	0	0	0	0	0	0	0	0	0	0	0	1	1
974	FAS_HUMAN	Fatty acid synthase	S974	82%	0.82	46	0	0	0	1	0	1	3	1	0	4	1	2	13
975	FAS_HUMAN	Fatty acid synthase	T1407	59%	0.59	3	0	0	0	0	0	0	0	0	0	0	1	1	2
976	FAS_HUMAN	Fatty acid synthase	T2204	100%	1.00	155	0	0	0	0	0	1	0	0	0	14	14	8	37
977	FAS_HUMAN	Fatty acid synthase	T976	99%	0.99	101	2	4	0	1	0	2	0	0	1	5	6	4	25
978	FAS_HUMAN	Fatty acid synthase	T980	25%	0.25	9	1	0	0	0	0	0	0	0	0	0	0	0	1
979	FXL19_HUMAN	F-box/LRR-repeat protein 19	S408	96%	0.96	3	0	1	0	0	0	0	0	1	0	0	0	0	2
980	FXL19_HUMAN	F-box/LRR-repeat protein 19	T413	3%	0.03	2	1	0	0	1	0	0	0	0	0	0	0	0	2
981	FCHO1_HUMAN	FCH domain only protein 1	S131	32%	0.32	1	0	0	0	1	0	0	0	0	0	0	0	0	1
982	FCHO2_HUMAN	FCH domain only protein 2	S387	100%	1.00	2	0	0	0	0	0	0	0	0	0	0	0	2	2
983	FCHO2_HUMAN	FCH domain only protein 2	S488	100%	1.00	27	1	2	0	0	1	0	0	2	2	1	2	1	12
984	FCHO2_HUMAN	FCH domain only protein 2	S496	90%	0.90	17	0	0	0	0	0	2	0	0	0	1	3	1	7
985	FCHO2_HUMAN	FCH domain only protein 2	S533	31%	0.31	3	0	0	0	0	0	0	0	1	0	0	0	0	1
986	FCHO2_HUMAN	FCH domain only protein 2	S536	46%	0.46	9	2	0	1	1	0	0	1	2	0	0	0	1	8
987	FCHO2_HUMAN	FCH domain only protein 2	T492	18%	0.18	4	0	0	1	0	0	1	0	0	0	0	0	0	2
988	FCHO2_HUMAN	FCH domain only protein 2	T495	9%	0.09	4	0	0	0	0	0	0	1	0	0	0	1	0	2
989	FCHO2_HUMAN	FCH domain only protein 2	T542	30%	0.30	5	0	1	0	0	0	0	2	0	0	1	0	0	4

990	FRIH_HUMAN	Ferritin heavy chain	S179	61%	0.61	1	0	0	0	0	0	0	1	0	0	0	0	0	1
991	FHOD1_HUMAN	FH1/FH2 domain-containing protein 1	S573	97%	0.97	1	0	0	0	0	0	0	0	0	0	0	0	1	1
992	FHOD3_HUMAN	FH1/FH2 domain-containing protein 3	S761	16%	0.16	1	1	0	0	0	0	0	0	0	0	0	0	0	1
993	FGFR2_HUMAN	Fibroblast growth factor receptor 2	S529	33%	0.33	3	0	0	0	0	0	0	0	0	0	1	0	0	1
994	FGL2_HUMAN	Fibroleukin	Y316	42%	0.42	1	1	0	0	0	0	0	0	0	0	0	0	0	1
995	FGL2_HUMAN	Fibroleukin	Y320	88%	0.88	1	1	0	0	0	0	0	0	0	0	0	0	0	1
996	FNDC1_HUMAN	Fibronectin type III domain-containing protein 1	T1117	93%	0.93	1	0	0	0	0	0	0	0	0	0	0	1	0	1
997	FBSL_HUMAN	Fibrosin-1-like protein	T1010	100%	1.00	1	0	1	0	0	0	0	0	0	0	0	0	0	1
998	FBSL_HUMAN	Fibrosin-1-like protein	T989	85%	0.85	1	0	1	0	0	0	0	0	0	0	0	0	0	1
999	FSIP2_HUMAN	Fibrous sheath-interacting protein 2	S3981	65%	0.65	2	0	0	0	0	0	1	0	0	0	0	0	0	1
1000	FSIP2_HUMAN	Fibrous sheath-interacting protein 2	S464	97%	0.97	1	0	0	1	0	0	0	0	0	0	0	0	0	1
1001	FSIP2_HUMAN	Fibrous sheath-interacting protein 2	S465	97%	0.97	1	0	0	1	0	0	0	0	0	0	0	0	0	1
1002	FSIP2_HUMAN	Fibrous sheath-interacting protein 2	S470	99%	0.99	1	0	0	1	0	0	0	0	0	0	0	0	0	1
1003	FSIP2_HUMAN	Fibrous sheath-interacting protein 2	S483	96%	0.96	1	0	0	1	0	0	0	0	0	0	0	0	0	1
1004	FSIP2_HUMAN	Fibrous sheath-interacting protein 2	S484	80%	0.80	1	0	0	1	0	0	0	0	0	0	0	0	0	1
1005	FSIP2_HUMAN	Fibrous sheath-interacting protein 2	T3974	84%	0.84	12	0	0	0	0	0	1	0	0	0	0	0	0	1
1006	FSIP2_HUMAN	Fibrous sheath-interacting protein 2	T477	97%	0.97	1	0	0	1	0	0	0	0	0	0	0	0	0	1
1007	FSIP2_HUMAN	Fibrous sheath-interacting protein 2	Y3967	44%	0.44	11	0	0	0	0	0	1	0	0	0	0	0	0	1
1008	FSIP2_HUMAN	Fibrous sheath-interacting protein 2	Y3971	77%	0.77	12	0	0	0	0	0	1	0	0	0	0	0	0	1
1009	FSIP2_HUMAN	Fibrous sheath-interacting protein 2	Y466	98%	0.98	1	0	0	1	0	0	0	0	0	0	0	0	0	1
1010	FLNA_HUMAN	Filamin-A	S1459	100%	1.00	12	0	2	5	0	0	0	0	1	0	0	0	0	8

1011	FLNA_HUMAN	Filamin-A	S2152	100%	1.00	452	8	13	11	14	13	15	15	12	18	0	0	0	119
1012	FLNA_HUMAN	Filamin-A	S2163	97%	0.97	1	0	0	1	0	0	0	0	0	0	0	0	0	1
1013	FLNA_HUMAN	Filamin-A	S2180	97%	0.97	16	0	0	0	0	0	0	0	0	2	0	0	0	2
1014	FLNA_HUMAN	Filamin-A	S2327	96%	0.96	11	0	1	0	0	0	0	0	0	0	0	0	0	1
1015	FLNA_HUMAN	Filamin-A	T1594	100%	1.00	1	0	0	0	0	1	0	0	0	0	0	0	0	1
1016	FLNA_HUMAN	Filamin-A	T1613	11%	0.11	1	0	0	0	0	1	0	0	0	0	0	0	0	1
1017	FLNB_HUMAN	Filamin-B	S1505	100%	1.00	1	0	0	0	0	0	0	0	0	0	0	1	0	1
1018	FLNB_HUMAN	Filamin-B	S2107	100%	1.00	167	7	6	4	8	10	5	5	3	3	12	9	7	79
1019	FLNB_HUMAN	Filamin-B	S2478	100%	1.00	485	6	7	6	12	8	7	8	12	7	18	18	16	125
1020	FLNB_HUMAN	Filamin-B	S2481	43%	0.43	70	1	0	1	1	1	2	2	1	1	2	1	3	16
1021	FLNB_HUMAN	Filamin-B	S983	99%	0.99	6	0	0	0	0	0	0	0	0	0	1	1	0	2
1022	FLNB_HUMAN	Filamin-B	S985	34%	0.34	3	0	0	0	0	0	0	0	0	0	0	0	1	1
1023	FLNB_HUMAN	Filamin-B	T2485	22%	0.22	7	0	0	0	0	1	0	1	0	1	0	1	1	5
1024	FKB15_HUMAN	FK506-binding protein 15	S1162	51%	0.51	7	0	0	0	0	1	0	0	0	0	0	0	0	1
1025	FKB15_HUMAN	FK506-binding protein 15	S1164	100%	1.00	7	0	0	0	0	1	0	0	0	0	0	0	0	1
1026	FKB15_HUMAN	FK506-binding protein 15	S311	90%	0.90	8	0	0	0	0	0	0	0	0	0	2	1	0	3
1027	FKB15_HUMAN	FK506-binding protein 15	S956	20%	0.20	1	0	0	0	0	0	0	0	1	0	0	0	0	1
1028	FKB15_HUMAN	FK506-binding protein 15	S960	77%	0.77	3	0	1	1	0	0	0	0	0	0	1	0	0	3
1029	FKB15_HUMAN	FK506-binding protein 15	S979	54%	0.54	3	0	0	1	0	0	1	0	0	0	0	1	0	3
1030	FWCH2_HUMAN	FLYWCH family member 2	S21	77%	0.77	1	0	0	0	0	0	0	0	0	0	0	1	0	1

1031	FAK1_HUMAN	Focal adhesion kinase 1	S910	100%	1.00	35	0	0	0	0	0	0	0	0	0	1	1	2	4
1032	FAK1_HUMAN	Focal adhesion kinase 1	T914	4%	0.04	2	0	0	0	0	0	0	0	0	0	0	1	0	1
1033	FOXF1_HUMAN	Forkhead box protein F1	S2	100%	1.00	1	0	0	1	0	0	0	0	0	0	0	0	0	1
1034	FOXF1_HUMAN	Forkhead box protein F1	S3	100%	1.00	2	0	0	1	0	0	0	0	0	0	0	0	0	1
1035	FOXF1_HUMAN	Forkhead box protein F1	S30	96%	0.96	2	1	0	1	0	0	0	0	0	0	0	0	0	2
1036	FOXF1_HUMAN	Forkhead box protein F1	S31	96%	0.96	3	1	0	1	0	0	0	0	0	0	0	0	0	2
1037	FOXF1_HUMAN	Forkhead box protein F1	S34	94%	0.94	1	1	0	0	0	0	0	0	0	0	0	0	0	1
1038	FOXK1_HUMAN	Forkhead box protein K1	S416	100%	1.00	8	2	1	0	0	0	0	1	0	1	0	0	0	5
1039	FOXK1_HUMAN	Forkhead box protein K1	S420	100%	1.00	12	2	1	0	0	0	0	1	0	1	0	0	0	5
1040	FOXK1_HUMAN	Forkhead box protein K1	S445	100%	1.00	13	0	0	1	1	1	1	0	2	0	0	0	0	6
1041	FOXK2_HUMAN	Forkhead box protein K2	S398	100%	1.00	57	4	0	3	2	1	0	1	1	3	0	0	0	15
1042	FOXO3_HUMAN	Forkhead box protein O3	S12	100%	1.00	4	0	0	0	0	0	0	0	0	0	2	0	0	2
1043	FOXO3_HUMAN	Forkhead box protein O3	S7	100%	1.00	5	0	0	0	0	0	0	0	0	1	2	0	0	3
1044	FNBP4_HUMAN	Formin-binding protein 4	S18	100%	1.00	3	0	0	0	0	0	0	0	0	0	1	0	1	2
1045	FMNL2_HUMAN	Formin-like protein 2	S171	100%	1.00	1	0	0	0	0	0	1	0	0	0	0	0	0	1
1046	FOSL2_HUMAN	Fos-related antigen 2	S200	100%	1.00	69	4	6	8	8	4	4	1	1	4	4	3	4	51
1047	FGD3_HUMAN	FYVE, RhoGEF and PH domain-containing protein 3	S327	70%	0.70	4	0	0	1	0	0	0	0	0	0	0	0	0	1
1048	FGD3_HUMAN	FYVE, RhoGEF and PH domain-containing protein 3	T333	67%	0.67	3	0	0	1	0	0	0	0	0	0	0	0	0	1
1049	GPTC8_HUMAN	G patch domain-containing protein 8	S740	100%	1.00	16	1	3	3	0	0	1	2	1	1	2	1	1	16

1050	GTSE1_HUMAN	G2 and S phase-expressed protein 1	S168	55%	0.55	1	1	0	0	0	0	0	0	0	0	0	0	0	1
1051	GTSE1_HUMAN	G2 and S phase-expressed protein 1	S173	71%	0.71	1	1	0	0	0	0	0	0	0	0	0	0	0	1
1052	GTSE1_HUMAN	G2 and S phase-expressed protein 1	T181	62%	0.62	1	1	0	0	0	0	0	0	0	0	0	0	0	1
1053	CCNB3_HUMAN	G2/mitotic-specific cyclin-B3	S1093	33%	0.33	1	0	0	0	0	1	0	0	0	0	0	0	0	1
1054	CCNB3_HUMAN	G2/mitotic-specific cyclin-B3	S1095	33%	0.33	1	0	0	0	0	1	0	0	0	0	0	0	0	1
1055	CCNB3_HUMAN	G2/mitotic-specific cyclin-B3	S1100	33%	0.33	1	0	0	0	0	1	0	0	0	0	0	0	0	1
1056	POL_HTL32	Gag-Pro-Pol polyprotein	S1426	50%	0.50	2	0	0	0	0	0	0	0	0	1	0	0	0	1
1057	G3ST3_HUMAN	Galactose-3-O-sulfotransferase 3	T74	34%	0.34	1	0	0	0	0	0	0	1	0	0	0	0	0	1
1058	G3ST3_HUMAN	Galactose-3-O-sulfotransferase 3	T78	8%	0.08	1	0	0	0	0	0	0	1	0	0	0	0	0	1
1059	ADDG_HUMAN	Gamma-adducin	S681	20%	0.20	1	0	0	0	0	0	0	1	0	0	0	0	0	1
1060	GBRR3_HUMAN	Gamma-aminobutyric acid receptor subunit rho-3	S301	51%	0.51	1	0	0	0	1	0	0	0	0	0	0	0	0	1
1061	GBRR3_HUMAN	Gamma-aminobutyric acid receptor subunit rho-3	S325	43%	0.43	1	0	0	0	1	0	0	0	0	0	0	0	0	1
1062	GBRR3_HUMAN	Gamma-aminobutyric acid receptor subunit rho-3	T305	68%	0.68	1	0	0	0	1	0	0	0	0	0	0	0	0	1
1063	GBRR3_HUMAN	Gamma-aminobutyric acid receptor subunit rho-3	T306	28%	0.28	1	0	0	0	1	0	0	0	0	0	0	0	0	1
1064	IF16_HUMAN	Gamma-interferon-inducible protein 16	S153	100%	1.00	102	8	6	5	7	8	7	7	4	3	0	0	0	55
1065	IF16_HUMAN	Gamma-interferon-inducible protein 16	T149	0%	0.00	1	0	0	0	0	0	0	0	0	1	0	0	0	1
1066	SAP3_HUMAN	Ganglioside GM2 activator	S50	68%	0.68	3	0	0	0	1	0	0	0	0	0	0	0	0	1
1067	SAP3_HUMAN	Ganglioside GM2 activator	S73	86%	0.86	3	0	0	0	1	0	0	0	0	0	0	0	0	1
1068	SAP3_HUMAN	Ganglioside GM2 activator	S77	97%	0.97	3	0	0	0	1	0	0	0	0	0	0	0	0	1

1069	SAP3_HUMAN	Ganglioside GM2 activator	S78	97%	0.97	3	0	0	0	1	0	0	0	0	0	0	0	0	1
1070	SAP3_HUMAN	Ganglioside GM2 activator	T52	68%	0.68	3	0	0	0	1	0	0	0	0	0	0	0	0	1
1071	SAP3_HUMAN	Ganglioside GM2 activator	T65	85%	0.85	3	0	0	0	1	0	0	0	0	0	0	0	0	1
1072	SAP3_HUMAN	Ganglioside GM2 activator	T72	88%	0.88	3	0	0	0	1	0	0	0	0	0	0	0	0	1
1073	GA2L1_HUMAN	GAS2-like protein 1	S316	97%	0.97	1	0	0	0	0	0	0	0	0	1	0	0	0	1
1074	GCFC1_HUMAN	GC-rich sequence DNA-binding factor 1	S62	95%	0.95	25	0	0	1	1	1	0	0	0	0	1	0	0	4
1075	TF3C1_HUMAN	General transcription factor 3C polypeptide 1	S1845	12%	0.12	1	0	0	0	0	0	0	1	0	0	0	0	0	1
1076	TF3C1_HUMAN	General transcription factor 3C polypeptide 1	S1854	58%	0.58	7	1	1	0	0	0	1	1	0	1	0	0	0	5
1077	TF3C1_HUMAN	General transcription factor 3C polypeptide 1	S1856	51%	0.51	13	2	0	0	2	2	0	2	1	0	0	0	0	9
1078	TF3C2_HUMAN	General transcription factor 3C polypeptide 2	S893	7%	0.07	4	0	0	0	0	0	0	0	0	0	0	0	1	1
1079	TF3C4_HUMAN	General transcription factor 3C polypeptide 4	S611	100%	1.00	2	0	2	0	0	0	0	0	0	0	0	0	0	2
1080	GFPT1_HUMAN	Glucosamine--fructose-6-phosphate aminotransferase [isomerizing] 1	S261	100%	1.00	169	6	6	2	0	1	0	5	2	5	4	4	2	37
1081	GFPT1_HUMAN	Glucosamine--fructose-6-phosphate aminotransferase [isomerizing] 1	T262	33%	0.33	15	1	0	0	1	0	0	0	0	0	2	1	0	5
1082	GFPT1_HUMAN	Glucosamine--fructose-6-phosphate aminotransferase [isomerizing] 1	T263	89%	0.89	8	0	0	0	1	0	0	0	0	0	0	0	0	1
1083	QRIC1_HUMAN	Glutamine-rich protein 1	S345	100%	1.00	20	0	0	0	0	0	0	0	0	1	0	0	0	1
1084	G3P_HUMAN	Glyceraldehyde-3-phosphate dehydrogenase	T177	4%	0.04	1	0	0	0	0	0	0	0	0	0	0	0	1	1
1085	G3P_HUMAN	Glyceraldehyde-3-phosphate dehydrogenase	T184	100%	1.00	82	0	0	0	0	0	0	0	0	0	0	0	10	10

1086	GSK3A_HUMAN	Glycogen synthase kinase-3 alpha	S21	54%	0.54	4	0	0	0	0	1	0	0	0	0	0	1	0	2
1087	GSK3A_HUMAN	Glycogen synthase kinase-3 alpha	T19	17%	0.17	2	0	0	0	0	1	0	1	0	0	0	0	0	2
1088	GSK3A_HUMAN	Glycogen synthase kinase-3 alpha	Y279	100%	1.00	16	0	0	0	0	0	0	1	0	0	0	0	0	1
1089	GOLI4_HUMAN	Golgi integral membrane protein 4	T640	95%	0.95	51	2	3	2	3	2	1	3	3	1	3	3	3	29
1090	GORS1_HUMAN	Golgi reassembly-stacking protein 1	T216	100%	1.00	4	1	0	0	0	0	0	1	0	0	0	0	0	2
1091	GORS2_HUMAN	Golgi reassembly-stacking protein 2	S436	82%	0.82	5	0	0	0	1	0	0	0	0	0	0	0	0	1
1092	GORS2_HUMAN	Golgi reassembly-stacking protein 2	S451	100%	1.00	68	2	2	2	1	0	2	0	0	1	1	2	2	15
1093	GCP60_HUMAN	Golgi resident protein GCP60	S43	100%	1.00	193	0	0	0	2	2	0	0	2	3	2	6	7	24
1094	GCP60_HUMAN	Golgi resident protein GCP60	S47	96%	0.96	243	0	2	3	1	0	2	3	1	2	5	3	4	26
1095	GOGA4_HUMAN	Golgin subfamily A member 4	S71	100%	1.00	106	4	6	6	6	4	4	6	4	4	6	6	6	62
1096	GBF1_HUMAN	Golgi-specific brefeldin A-resistance guanine nucleotide exchange factor 1	S1298	100%	1.00	150	1	0	2	3	2	1	1	3	4	0	2	2	21
1097	GBF1_HUMAN	Golgi-specific brefeldin A-resistance guanine nucleotide exchange factor 1	S1300	94%	0.94	32	0	0	0	0	0	0	0	0	1	0	0	0	1
1098	GON4L_HUMAN	GON-4-like protein	S1258	100%	1.00	2	0	0	1	0	0	0	0	0	0	0	0	1	2
1099	GON4L_HUMAN	GON-4-like protein	T1260	25%	0.25	1	0	0	0	0	0	0	0	0	0	0	1	0	1
1100	PIGT_HUMAN	GPI transamidase component PIG-T	S329	100%	1.00	3	0	0	0	1	1	0	0	1	0	0	0	0	3
1101	PIGT_HUMAN	GPI transamidase component PIG-T	T323	100%	1.00	3	0	0	0	1	1	0	0	1	0	0	0	0	3
1102	NAR1_HUMAN	GPI-linked NAD(P)(+)--arginine ADP-ribosyltransferase 1	S24	22%	0.22	51	2	0	1	1	0	0	1	1	1	2	1	2	12
1103	GPN1_HUMAN	GPN-loop GTPase 1	S312	64%	0.64	18	1	0	0	0	0	1	1	0	0	1	0	1	5

1104	GPN1_HUMAN	GPN-loop GTPase 1	S314	100%	1.00	137	1	0	2	0	1	2	2	1	1	7	8	5	30
1105	GPN1_HUMAN	GPN-loop GTPase 1	S338	100%	1.00	96	4	0	1	3	2	3	3	1	1	1	2	2	23
1106	GP126_HUMAN	G-protein coupled receptor 126	S54	48%	0.48	1	0	0	0	0	0	0	0	0	0	0	1	0	1
1107	GP126_HUMAN	G-protein coupled receptor 126	S64	23%	0.23	1	0	0	0	0	0	0	0	0	0	0	1	0	1
1108	GP126_HUMAN	G-protein coupled receptor 126	Y61	42%	0.42	1	0	0	0	0	0	0	0	0	0	0	1	0	1
1109	GPSM2_HUMAN	G-protein-signaling modulator 2	S483	100%	1.00	11	0	1	0	2	0	0	0	0	1	0	0	0	4
1110	GRM1C_HUMAN	GRAM domain-containing protein 1C	S279	92%	0.92	1	0	0	0	0	0	1	0	0	0	0	0	0	1
1111	GRB1L_HUMAN	GREB1-like protein	S1172	97%	0.97	2	0	0	0	0	0	1	0	0	0	0	0	0	1
1112	GRB1L_HUMAN	GREB1-like protein	S1173	93%	0.93	2	0	0	0	0	0	1	0	0	0	0	0	0	1
1113	GCC1_HUMAN	GRIP and coiled-coil domain-containing protein 1	S620	93%	0.93	9	1	0	0	0	0	0	1	1	0	0	0	0	3
1114	PG12B_HUMAN	Group XIIB secretory phospholipase A2-like protein	S24	69%	0.69	1	0	1	0	0	0	0	0	0	0	0	0	0	1
1115	PG12B_HUMAN	Group XIIB secretory phospholipase A2-like protein	S30	37%	0.37	1	0	1	0	0	0	0	0	0	0	0	0	0	1
1116	PG12B_HUMAN	Group XIIB secretory phospholipase A2-like protein	S32	37%	0.37	1	0	1	0	0	0	0	0	0	0	0	0	0	1
1117	PG12B_HUMAN	Group XIIB secretory phospholipase A2-like protein	T27	37%	0.37	1	0	1	0	0	0	0	0	0	0	0	0	0	1
1118	GRB7_HUMAN	Growth factor receptor-bound protein 7	S361	100%	1.00	2	0	0	0	0	0	0	0	0	0	0	0	2	2
1119	GRB7_HUMAN	Growth factor receptor-bound protein 7	S79	39%	0.39	1	0	0	0	0	0	0	0	0	0	0	1	0	1
1120	GRB7_HUMAN	Growth factor receptor-bound protein 7	T23	98%	0.98	9	0	0	0	0	0	0	0	0	0	2	0	0	2
1121	GAPD1_HUMAN	GTPase-activating protein and VPS9 domain-containing protein 1	S757	22%	0.22	3	0	0	0	0	0	0	0	0	0	0	2	0	2

1122	GAPD1_HUMAN	GTPase-activating protein and VPS9 domain-containing protein 1	S758	22%	0.22	7	0	0	1	0	0	0	0	0	0	1	2	0	4
1123	GAPD1_HUMAN	GTPase-activating protein and VPS9 domain-containing protein 1	S761	72%	0.72	5	0	0	0	0	0	0	0	0	0	1	0	0	1
1124	GAPD1_HUMAN	GTPase-activating protein and VPS9 domain-containing protein 1	Y155	100%	1.00	2	0	0	0	0	0	0	1	1	0	0	0	0	2
1125	GTPB1_HUMAN	GTP-binding protein 1	S24	6%	0.06	2	0	0	0	0	1	0	0	0	0	0	0	1	2
1126	GTPB1_HUMAN	GTP-binding protein 1	S25	99%	0.99	29	2	3	2	0	1	0	0	2	0	1	0	2	13
1127	DKC1_HUMAN	H/ACA ribonucleoprotein complex subunit 4	S21	100%	1.00	116	1	0	1	2	1	2	1	1	0	2	0	2	13
1128	DKC1_HUMAN	H/ACA ribonucleoprotein complex subunit 4	S494	100%	1.00	22	0	2	3	1	3	4	1	2	2	0	2	1	21
1129	DKC1_HUMAN	H/ACA ribonucleoprotein complex subunit 4	T496	25%	0.25	1	0	0	0	0	0	0	0	0	1	0	0	0	1
1130	DKC1_HUMAN	H/ACA ribonucleoprotein complex subunit 4	T497	42%	0.42	5	1	0	1	1	0	0	0	2	0	0	0	0	5
1131	HAUS8_HUMAN	HAUS augmin-like complex subunit 8	S160	33%	0.33	1	0	0	1	0	0	0	0	0	0	0	0	0	1
1132	HAUS8_HUMAN	HAUS augmin-like complex subunit 8	T157	33%	0.33	1	0	0	1	0	0	0	0	0	0	0	0	0	1
1133	H1BP3_HUMAN	HCLS1-binding protein 3	S280	100%	1.00	1	0	0	0	0	0	0	0	0	0	1	0	0	1
1134	H1BP3_HUMAN	HCLS1-binding protein 3	S289	90%	0.90	1	0	0	0	0	0	0	0	0	0	1	0	0	1
1135	H1BP3_HUMAN	HCLS1-binding protein 3	S295	40%	0.40	1	0	0	0	0	0	0	0	0	0	1	0	0	1
1136	H1BP3_HUMAN	HCLS1-binding protein 3	S297	95%	0.95	1	0	0	0	0	0	0	0	0	0	1	0	0	1
1137	H1BP3_HUMAN	HCLS1-binding protein 3	S302	85%	0.85	1	0	0	0	0	0	0	0	0	0	1	0	0	1
1138	HSP74_HUMAN	Heat shock 70 kDa protein 4	S647	94%	0.94	6	0	1	0	0	0	0	0	0	0	0	0	0	1

1139	HSP74_HUMAN	Heat shock 70 kDa protein 4	S76	100%	1.00	44	2	0	4	0	0	3	1	0	0	4	7	5	26
1140	HSP7C_HUMAN	Heat shock cognate 71 kDa protein	S613	25%	0.25	3	0	0	0	0	1	0	0	1	0	0	0	0	2
1141	HSP7C_HUMAN	Heat shock cognate 71 kDa protein	T608	28%	0.28	2	0	0	0	0	1	0	0	0	0	0	1	0	2
1142	HSP7C_HUMAN	Heat shock cognate 71 kDa protein	Y611	58%	0.58	3	0	0	0	0	0	0	0	1	0	0	1	0	2
1143	HSF1_HUMAN	Heat shock factor protein 1	S314	100%	1.00	126	2	3	3	3	4	4	3	2	2	3	3	2	34
1144	HSF1_HUMAN	Heat shock factor protein 1	S319	25%	0.25	7	0	0	0	0	0	0	1	0	0	0	0	0	1
1145	HSF1_HUMAN	Heat shock factor protein 1	S320	47%	0.47	43	0	1	3	1	1	0	2	2	0	0	0	1	11
1146	HSF1_HUMAN	Heat shock factor protein 1	S326	100%	1.00	43	0	0	0	0	0	0	0	0	1	1	3	0	5
1147	HSF1_HUMAN	Heat shock factor protein 1	S333	5%	0.05	2	0	0	0	0	0	0	0	0	0	0	0	1	1
1148	HSF1_HUMAN	Heat shock factor protein 1	S363	100%	1.00	16	1	0	2	4	1	1	2	1	1	0	0	1	14
1149	HSF1_HUMAN	Heat shock factor protein 1	T323	100%	1.00	122	4	2	0	1	1	1	2	2	3	2	2	5	25
1150	HSF1_HUMAN	Heat shock factor protein 1	T369	8%	0.08	2	0	0	0	0	1	0	0	1	0	0	0	0	2
1151	HS105_HUMAN	Heat shock protein 105 kDa	S809	100%	1.00	12	1	3	0	0	2	0	1	0	1	1	2	1	12
1152	HSPB1_HUMAN	Heat shock protein beta-1	S82	99%	0.99	1	0	0	0	0	0	0	0	0	0	1	0	0	1
1153	HS90A_HUMAN	Heat shock protein HSP 90-alpha	S252	100%	1.00	6	0	0	0	0	1	0	0	1	0	0	0	1	3
1154	HS90A_HUMAN	Heat shock protein HSP 90-alpha	S263	100%	1.00	223	2	6	6	3	6	6	6	4	5	3	3	6	56
1155	HS90B_HUMAN	Heat shock protein HSP 90-beta	S255	100%	1.00	804	25	18	25	29	24	25	23	22	28	23	18	30	290
1156	HS90B_HUMAN	Heat shock protein HSP 90-beta	S261	2%	0.02	6	0	0	1	0	1	0	0	0	0	0	0	0	2
1157	SKIV2_HUMAN	Helicase SKI2W	S256	100%	1.00	102	3	3	3	2	3	2	3	2	4	4	3	3	35

1158	HN1_HUMAN	Hematological and neurological expressed 1 protein	S115	11%	0.11	1	0	0	0	0	0	0	0	0	0	0	0	1	1
1159	HN1_HUMAN	Hematological and neurological expressed 1 protein	S119	94%	0.94	20	0	0	0	0	0	0	0	0	0	2	0	0	2
1160	HN1_HUMAN	Hematological and neurological expressed 1 protein	S131	100%	1.00	132	0	0	1	3	1	0	0	0	3	6	3	6	23
1161	HN1_HUMAN	Hematological and neurological expressed 1 protein	S87	100%	1.00	####	17	18	22	20	19	19	20	20	21	19	15	17	227
1162	HN1_HUMAN	Hematological and neurological expressed 1 protein	S88	98%	0.98	314	6	6	8	10	7	8	10	8	10	5	5	4	87
1163	HN1_HUMAN	Hematological and neurological expressed 1 protein	S91	36%	0.36	4	0	0	0	2	0	0	0	0	0	0	0	0	2
1164	HN1_HUMAN	Hematological and neurological expressed 1 protein	S92	100%	1.00	19	0	0	0	0	0	0	0	0	0	1	1	0	2
1165	HMCN1_HUMAN	Hemicentin-1	S2262	70%	0.70	1	0	0	0	0	0	0	1	0	0	0	0	0	1
1166	HMCN1_HUMAN	Hemicentin-1	S2268	88%	0.88	1	0	0	0	0	0	0	1	0	0	0	0	0	1
1167	HMCN1_HUMAN	Hemicentin-1	S2272	74%	0.74	1	0	0	0	0	0	0	1	0	0	0	0	0	1
1168	HMCN1_HUMAN	Hemicentin-1	T2277	89%	0.89	1	0	0	0	0	0	0	1	0	0	0	0	0	1
1169	HPSE2_HUMAN	Heparanase-2	S194	86%	0.86	4	0	0	1	0	0	0	0	0	0	0	0	0	1
1170	HDGF_HUMAN	Hepatoma-derived growth factor	S132	100%	1.00	443	1	1	4	8	4	4	3	2	5	5	3	7	47
1171	HDGF_HUMAN	Hepatoma-derived growth factor	S133	100%	1.00	523	1	1	4	8	4	5	6	2	6	8	4	9	58
1172	HDGF_HUMAN	Hepatoma-derived growth factor	S165	100%	1.00	87	4	5	5	3	2	4	3	3	3	3	3	4	42
1173	GAK8_HUMAN	HERV-K_3q12.3 provirus ancestral Gag polyprotein	S129	28%	0.28	1	0	0	0	0	0	0	0	0	0	0	1	0	1

1174	GAK8_HUMAN	HERV-K_3q12.3 provirus ancestral Gag polyprotein	T136	86%	0.86	1	0	0	0	0	0	0	0	0	0	0	1	0	1
1175	GAK8_HUMAN	HERV-K_3q12.3 provirus ancestral Gag polyprotein	Y134	62%	0.62	1	0	0	0	0	0	0	0	0	0	0	1	0	1
1176	GAK8_HUMAN	HERV-K_3q12.3 provirus ancestral Gag polyprotein	Y157	28%	0.28	1	0	0	0	0	0	0	0	0	0	0	1	0	1
1177	ROAA_HUMAN	Heterogeneous nuclear ribonucleoprotein A/B	S27	84%	0.84	1	0	0	0	0	0	0	0	0	0	1	0	0	1
1178	ROAA_HUMAN	Heterogeneous nuclear ribonucleoprotein A/B	T16	29%	0.29	1	0	0	0	0	0	0	0	0	0	1	0	0	1
1179	ROA3_HUMAN	Heterogeneous nuclear ribonucleoprotein A3	S14	100%	1.00	1	0	0	0	0	0	0	0	0	1	0	0	0	1
1180	ROA3_HUMAN	Heterogeneous nuclear ribonucleoprotein A3	S355	65%	0.65	5	0	0	0	1	0	0	0	0	1	0	0	0	2
1181	ROA3_HUMAN	Heterogeneous nuclear ribonucleoprotein A3	S356	9%	0.09	1	1	0	0	0	0	0	0	0	0	0	0	0	1
1182	ROA3_HUMAN	Heterogeneous nuclear ribonucleoprotein A3	S370	84%	0.84	5	0	0	0	1	0	0	0	0	1	0	0	0	2
1183	ROA3_HUMAN	Heterogeneous nuclear ribonucleoprotein A3	S375	40%	0.40	5	0	0	0	1	0	0	0	0	1	0	0	0	2
1184	ROA3_HUMAN	Heterogeneous nuclear ribonucleoprotein A3	Y364	53%	0.53	5	0	0	0	1	0	0	0	0	1	0	0	0	2
1185	HNRPD_HUMAN	Heterogeneous nuclear ribonucleoprotein D0	S82	85%	0.85	5	1	1	0	0	0	0	0	1	2	0	0	0	5
1186	HNRPF_HUMAN	Heterogeneous nuclear ribonucleoprotein F	S310	100%	1.00	4	1	0	0	0	0	0	0	0	0	2	0	1	4
1187	HNRH1_HUMAN	Heterogeneous nuclear ribonucleoprotein H	S104	100%	1.00	313	5	12	12	10	12	9	12	10	10	16	16	10	134
1188	HNRH1_HUMAN	Heterogeneous nuclear ribonucleoprotein H	S310	100%	1.00	4	1	0	0	0	0	0	0	0	0	2	0	1	4
1189	HNRH1_HUMAN	Heterogeneous nuclear ribonucleoprotein H	T100	93%	0.93	22	0	1	3	1	0	1	1	0	0	1	0	2	10
1190	HNRH1_HUMAN	Heterogeneous nuclear ribonucleoprotein H	T107	27%	0.27	14	0	0	1	0	1	1	0	1	1	1	0	2	8

1191	HNRH2_HUMAN	Heterogeneous nuclear ribonucleoprotein H2	S104	100%	1.00	12	0	0	0	0	0	0	12	0	0	0	0	0	12
1192	HNRH2_HUMAN	Heterogeneous nuclear ribonucleoprotein H2	T100	0%	0.00	1	0	0	0	0	0	0	1	0	0	0	0	0	1
1193	HNRH2_HUMAN	Heterogeneous nuclear ribonucleoprotein H2	T286	50%	0.50	1	0	0	0	0	0	0	1	0	0	0	0	0	1
1194	HNRH2_HUMAN	Heterogeneous nuclear ribonucleoprotein H2	T287	75%	0.75	1	0	0	0	0	0	0	1	0	0	0	0	0	1
1195	HNRH2_HUMAN	Heterogeneous nuclear ribonucleoprotein H2	Y266	24%	0.24	1	0	0	0	0	0	0	1	0	0	0	0	0	1
1196	HNRPK_HUMAN	Heterogeneous nuclear ribonucleoprotein K	S116	100%	1.00	343	7	6	4	5	3	5	3	4	3	6	8	5	59
1197	HNRPK_HUMAN	Heterogeneous nuclear ribonucleoprotein K	S214	90%	0.90	22	0	0	0	0	0	0	0	0	0	1	2	3	6
1198	HNRPK_HUMAN	Heterogeneous nuclear ribonucleoprotein K	S216	100%	1.00	25	0	0	0	0	0	0	0	0	0	1	4	4	9
1199	HNRPK_HUMAN	Heterogeneous nuclear ribonucleoprotein K	S284	100%	1.00	1	0	0	0	0	0	0	0	0	0	0	0	1	1
1200	HNRPK_HUMAN	Heterogeneous nuclear ribonucleoprotein K	T118	84%	0.84	37	0	0	0	0	0	0	0	1	1	2	2	0	6
1201	HNRPU_HUMAN	Heterogeneous nuclear ribonucleoprotein U	S271	100%	1.00	97	4	3	0	0	0	2	3	0	0	1	2	2	17
1202	HNRPU_HUMAN	Heterogeneous nuclear ribonucleoprotein U	S4	94%	0.94	4	0	0	0	0	0	0	0	0	3	0	0	1	4
1203	HNRPU_HUMAN	Heterogeneous nuclear ribonucleoprotein U	S59	100%	1.00	81	1	0	2	0	0	0	1	0	0	0	0	0	4
1204	HNRL1_HUMAN	Heterogeneous nuclear ribonucleoprotein U-like protein 1	S194	100%	1.00	1	0	0	0	0	0	0	0	1	0	0	0	0	1
1205	HNRL1_HUMAN	Heterogeneous nuclear ribonucleoprotein U-like protein 1	S716	23%	0.23	2	0	0	0	0	0	0	1	0	0	0	0	0	1
1206	HNRL1_HUMAN	Heterogeneous nuclear ribonucleoprotein U-like protein 1	S718	99%	0.99	102	5	3	2	2	2	1	2	1	1	0	0	0	19

1207	HNRL2_HUMAN	Heterogeneous nuclear ribonucleoprotein U-like protein 2	S161	100%	1.00	37	0	0	1	0	1	1	1	0	0	1	0	0	5
1208	HNRPC_HUMAN	Heterogeneous nuclear ribonucleoproteins C1/C2	S260	100%	1.00	225	2	2	6	2	0	2	6	1	0	0	0	0	21
1209	HIG1B_HUMAN	HIG1 domain family member 1B	S58	62%	0.62	2	0	0	0	0	1	0	0	0	0	0	0	0	1
1210	HIG1B_HUMAN	HIG1 domain family member 1B	S86	79%	0.79	2	0	0	0	0	1	0	0	0	0	0	0	0	1
1211	HIG1B_HUMAN	HIG1 domain family member 1B	Y85	84%	0.84	2	0	0	0	0	1	0	0	0	0	0	0	0	1
1212	HIG1B_HUMAN	HIG1 domain family member 1B	Y88	78%	0.78	2	0	0	0	0	1	0	0	0	0	0	0	0	1
1213	HDX_HUMAN	Highly divergent homeobox	S378	16%	0.16	1	0	0	0	0	0	0	0	0	0	0	1	0	1
1214	HDX_HUMAN	Highly divergent homeobox	T381	76%	0.76	1	0	0	0	0	0	0	0	0	0	0	1	0	1
1215	SYHC_HUMAN	Histidyl-tRNA synthetase, cytoplasmic	S27	100%	1.00	1	0	1	0	0	0	0	0	0	0	0	0	0	1
1216	HDAC1_HUMAN	Histone deacetylase 1	S393	100%	1.00	292	14	11	10	6	9	9	11	8	9	5	6	1	99
1217	HDAC1_HUMAN	Histone deacetylase 1	S421	100%	1.00	10	0	0	0	0	0	0	0	0	1	0	0	0	1
1218	HDAC1_HUMAN	Histone deacetylase 1	S423	100%	1.00	10	0	0	0	0	0	0	0	0	1	0	0	0	1
1219	HDAC2_HUMAN	Histone deacetylase 2	S394	100%	1.00	469	15	11	11	12	9	13	10	10	13	9	10	9	132
1220	HDAC2_HUMAN	Histone deacetylase 2	S422	100%	1.00	112	2	3	2	1	4	4	1	4	4	0	1	2	28
1221	HDAC2_HUMAN	Histone deacetylase 2	S424	100%	1.00	104	2	3	2	1	4	4	1	4	4	0	1	2	28
1222	H11_HUMAN	Histone H1.1	S42	92%	0.92	49	2	4	1	2	2	2	2	4	3	0	0	0	22
1223	H11_HUMAN	Histone H1.1	S44	96%	0.96	49	2	4	1	2	2	2	2	4	3	0	0	0	22
1224	H11_HUMAN	Histone H1.1	S52	99%	0.99	9	0	1	0	1	0	0	0	2	0	0	0	0	4
1225	H11_HUMAN	Histone H1.1	S53	89%	0.89	40	2	3	1	1	2	2	2	2	3	0	0	0	18

1226	H11_HUMAN	Histone H1.1	S54	93%	0.93	49	2	4	1	2	2	2	2	4	3	0	0	0	22
1227	H12_HUMAN	Histone H1.2	S2	50%	0.50	7	0	0	0	1	0	0	0	0	0	1	0	1	3
1228	H12_HUMAN	Histone H1.2	T4	99%	0.99	37	0	0	1	1	0	0	2	0	1	1	0	1	7
1229	H13_HUMAN	Histone H1.3	T10	99%	0.99	12	0	1	0	0	0	0	0	0	0	0	0	0	1
1230	H13_HUMAN	Histone H1.3	T18	100%	1.00	208	6	5	6	3	3	2	3	3	3	0	0	0	34
1231	H14_HUMAN	Histone H1.4	S2	99%	0.99	10	0	0	1	0	0	0	0	0	0	0	0	1	2
1232	H14_HUMAN	Histone H1.4	T18	100%	1.00	647	15	14	14	12	15	13	12	12	10	14	14	13	158
1233	H14_HUMAN	Histone H1.4	T4	50%	0.50	6	0	0	0	0	0	0	0	1	1	0	0	1	3
1234	H15_HUMAN	Histone H1.5	S18	100%	1.00	614	16	14	14	17	17	15	16	15	14	0	0	0	138
1235	H15_HUMAN	Histone H1.5	S2	90%	0.90	7	0	0	0	2	0	0	2	0	0	0	0	0	4
1236	H15_HUMAN	Histone H1.5	T11	55%	0.55	4	0	0	0	0	0	1	0	0	0	0	0	0	1
1237	H15_HUMAN	Histone H1.5	T4	25%	0.25	7	1	0	0	1	0	1	1	0	0	0	0	0	4
1238	H15_HUMAN	Histone H1.5	T9	39%	0.39	17	0	0	1	0	1	0	1	0	0	0	0	0	3
1239	H1X_HUMAN	Histone H1x	S31	98%	0.98	3	0	0	0	0	0	0	0	0	0	0	0	3	3
1240	EZH2_HUMAN	Histone-lysine N-methyltransferase EZH2	T487	100%	1.00	32	3	3	0	2	0	1	3	3	1	0	1	0	17
1241	MLL2_HUMAN	Histone-lysine N-methyltransferase MLL2	S4359	100%	1.00	3	0	0	0	0	0	0	1	0	0	1	0	0	2
1242	MLL2_HUMAN	Histone-lysine N-methyltransferase MLL2	S4738	100%	1.00	22	0	0	0	0	0	0	0	0	0	1	4	2	7
1243	MLL2_HUMAN	Histone-lysine N-methyltransferase MLL2	S48	15%	0.15	1	0	0	0	0	0	0	1	0	0	0	0	0	1
1244	MLL3_HUMAN	Histone-lysine N-methyltransferase MLL3	S3758	95%	0.95	2	0	0	0	1	0	0	0	0	0	0	0	0	1

1245	SETD2_HUMAN	Histone-lysine N-methyltransferase SETD2	S131	99%	0.99	1	0	0	0	0	0	0	0	1	0	0	0	0	1
1246	SETD7_HUMAN	Histone-lysine N-methyltransferase SETD7	S3	100%	1.00	4	2	0	0	0	2	0	0	0	0	0	0	0	4
1247	DOT1L_HUMAN	Histone-lysine N-methyltransferase, H3 lysine-79 specific	T385	100%	1.00	1	0	0	1	0	0	0	0	0	0	0	0	0	1
1248	HTSF1_HUMAN	HIV Tat-specific factor 1	S579	100%	1.00	12	0	0	0	0	0	0	2	0	0	0	0	0	2
1249	DOB_HUMAN	HLA class II histocompatibility antigen, DO beta chain	S29	41%	0.41	1	0	0	0	0	0	0	0	1	0	0	0	0	1
1250	DOB_HUMAN	HLA class II histocompatibility antigen, DO beta chain	T27	40%	0.40	1	0	0	0	0	0	0	0	1	0	0	0	0	1
1251	HJURP_HUMAN	Holliday junction recognition protein	S473	100%	1.00	24	0	0	2	0	0	0	0	0	0	2	3	2	9
1252	HJURP_HUMAN	Holliday junction recognition protein	S686	77%	0.77	5	0	0	0	0	0	1	0	0	0	0	0	0	1
1253	HJURP_HUMAN	Holliday junction recognition protein	S691	77%	0.77	5	0	0	0	0	0	1	0	0	0	0	0	0	1
1254	HJURP_HUMAN	Holliday junction recognition protein	S697	80%	0.80	5	0	0	0	0	0	1	0	0	0	0	0	0	1
1255	HJURP_HUMAN	Holliday junction recognition protein	S701	18%	0.18	1	0	0	0	0	0	0	1	0	0	0	0	0	1
1256	HJURP_HUMAN	Holliday junction recognition protein	S715	93%	0.93	5	0	0	0	0	0	1	0	0	0	0	0	0	1
1257	HJURP_HUMAN	Holliday junction recognition protein	S716	92%	0.92	5	0	0	0	0	0	1	0	0	0	0	0	0	1
1258	HJURP_HUMAN	Holliday junction recognition protein	S717	91%	0.91	5	0	0	0	0	0	1	0	0	0	0	0	0	1
1259	HJURP_HUMAN	Holliday junction recognition protein	S721	88%	0.88	5	0	0	0	0	0	1	0	0	0	0	0	0	1
1260	HJURP_HUMAN	Holliday junction recognition protein	T707	22%	0.22	1	0	0	0	0	0	0	1	0	0	0	0	0	1

1261	CUX1_HUMAN	Homeobox protein cut-like 1	S188	20%	0.20	1	0	0	0	0	0	1	0	0	0	0	0	0	1
1262	HXD13_HUMAN	Homeobox protein Hox-D13	S26	39%	0.39	10	0	0	1	0	0	0	0	1	1	0	0	0	3
1263	HXD13_HUMAN	Homeobox protein Hox-D13	S27	26%	0.26	8	0	0	0	0	0	1	1	0	1	0	1	0	4
1264	HXD13_HUMAN	Homeobox protein Hox-D13	S28	26%	0.26	51	4	3	4	3	3	1	2	2	1	0	2	2	27
1265	NKX61_HUMAN	Homeobox protein Nkx-6.1	S13	83%	0.83	2	0	0	1	0	0	0	0	0	0	0	0	0	1
1266	NKX61_HUMAN	Homeobox protein Nkx-6.1	S18	89%	0.89	1	0	0	1	0	0	0	0	0	0	0	0	0	1
1267	NKX61_HUMAN	Homeobox protein Nkx-6.1	S19	85%	0.85	1	0	0	1	0	0	0	0	0	0	0	0	0	1
1268	PKNX2_HUMAN	Homeobox protein PKNOX2	S38	75%	0.75	1	0	0	1	0	0	0	0	0	0	0	0	0	1
1269	PKNX2_HUMAN	Homeobox protein PKNOX2	S6	75%	0.75	1	0	0	1	0	0	0	0	0	0	0	0	0	1
1270	PKNX2_HUMAN	Homeobox protein PKNOX2	T12	75%	0.75	1	0	0	1	0	0	0	0	0	0	0	0	0	1
1271	PKNX2_HUMAN	Homeobox protein PKNOX2	T16	75%	0.75	1	0	0	1	0	0	0	0	0	0	0	0	0	1
1272	PKNX2_HUMAN	Homeobox protein PKNOX2	T33	75%	0.75	1	0	0	1	0	0	0	0	0	0	0	0	0	1
1273	PKNX2_HUMAN	Homeobox protein PKNOX2	Y24	75%	0.75	1	0	0	1	0	0	0	0	0	0	0	0	0	1
1274	HOME3_HUMAN	Homer protein homolog 3	S256	100%	1.00	8	0	0	0	1	1	2	0	0	0	0	0	0	4
1275	HORN_HUMAN	Hornerin	S936	100%	1.00	1	0	0	0	0	0	0	0	0	0	1	0	0	1
1276	HORN_HUMAN	Hornerin	S937	100%	1.00	1	0	0	0	0	0	0	0	0	0	1	0	0	1
1277	HORN_HUMAN	Hornerin	S938	100%	1.00	1	0	0	0	0	0	0	0	0	0	1	0	0	1
1278	HORN_HUMAN	Hornerin	S941	100%	1.00	1	0	0	0	0	0	0	0	0	0	1	0	0	1
1279	HORN_HUMAN	Hornerin	S942	95%	0.95	1	0	0	0	0	0	0	0	0	0	1	0	0	1
1280	HORN_HUMAN	Hornerin	S955	45%	0.45	1	0	0	0	0	0	0	0	0	0	1	0	0	1

1281	HORN_HUMAN	Hornerin	S956	62%	0.62	1	0	0	0	0	0	0	0	0	0	1	0	0	1
1282	HORN_HUMAN	Hornerin	S962	45%	0.45	1	0	0	0	0	0	0	0	0	0	1	0	0	1
1283	HORN_HUMAN	Hornerin	S964	45%	0.45	1	0	0	0	0	0	0	0	0	0	1	0	0	1
1284	HORN_HUMAN	Hornerin	S967	45%	0.45	1	0	0	0	0	0	0	0	0	0	1	0	0	1
1285	HORN_HUMAN	Hornerin	S968	45%	0.45	1	0	0	0	0	0	0	0	0	0	1	0	0	1
1286	HCFC1_HUMAN	Host cell factor 1	S1172	79%	0.79	69	5	2	3	0	3	1	1	0	0	0	0	3	18
1287	HCFC1_HUMAN	Host cell factor 1	S1196	38%	0.38	68	5	2	3	0	3	1	1	0	0	0	0	3	18
1288	HCFC1_HUMAN	Host cell factor 1	S1507	100%	1.00	103	1	1	1	2	2	3	2	1	2	2	2	3	22
1289	HCFC1_HUMAN	Host cell factor 1	S6	100%	1.00	35	2	0	1	0	0	0	0	0	0	1	0	0	4
1290	HCFC1_HUMAN	Host cell factor 1	T1171	58%	0.58	69	5	2	3	0	3	1	1	0	0	0	0	3	18
1291	CDC37_HUMAN	Hsp90 co-chaperone Cdc37	S13	99%	0.99	6	5	1	0	0	0	0	0	0	0	0	0	0	6
1292	CD37L_HUMAN	Hsp90 co-chaperone Cdc37-like 1	S32	100%	1.00	2	0	0	0	0	0	0	0	0	0	2	0	0	2
1293	HYAL1_HUMAN	Hyaluronidase-1	S245	63%	0.63	1	0	0	0	0	1	0	0	0	0	0	0	0	1
1294	HYAL1_HUMAN	Hyaluronidase-1	T255	63%	0.63	1	0	0	0	0	1	0	0	0	0	0	0	0	1
1295	HYAL1_HUMAN	Hyaluronidase-1	Y243	67%	0.67	1	0	0	0	0	1	0	0	0	0	0	0	0	1
1296	HYAL1_HUMAN	Hyaluronidase-1	Y247	63%	0.63	1	0	0	0	0	1	0	0	0	0	0	0	0	1
1297	HMCS1_HUMAN	Hydroxymethylglutaryl-CoA synthase, cytoplasmic	S4	100%	1.00	37	0	0	0	0	0	0	0	0	0	2	6	4	12
1298	HMCS1_HUMAN	Hydroxymethylglutaryl-CoA synthase, cytoplasmic	S486	92%	0.92	21	0	0	0	0	1	0	0	0	0	2	2	0	5
1299	HMCS1_HUMAN	Hydroxymethylglutaryl-CoA synthase, cytoplasmic	S495	100%	1.00	####	9	17	10	16	10	13	8	8	10	58	63	68	290

1300	HMCS1_HUMAN	Hydroxymethylglutaryl-CoA synthase, cytoplasmic	S516	100%	1.00	325	0	0	0	3	2	0	0	0	1	6	13	9	34
1301	HMCS1_HUMAN	Hydroxymethylglutaryl-CoA synthase, cytoplasmic	T490	83%	0.83	129	1	0	1	1	3	0	0	1	2	6	5	5	25
1302	HMCS1_HUMAN	Hydroxymethylglutaryl-CoA synthase, cytoplasmic	T506	0%	0.00	5	0	0	0	0	0	0	0	0	0	0	0	1	1
1303	HV106_HUMAN	Ig heavy chain V-I region SIE	S17	100%	1.00	1	0	0	0	0	0	0	0	0	0	0	0	1	1
1304	HV105_HUMAN	Ig heavy chain V-I region WOL	S17	100%	1.00	1	0	0	0	0	0	0	0	0	0	0	0	1	1
1305	KV303_HUMAN	Ig kappa chain V-III region NG9 (Fragment)	S58	75%	0.75	1	0	0	0	0	0	0	0	0	0	0	0	1	1
1306	KV303_HUMAN	Ig kappa chain V-III region NG9 (Fragment)	T61	83%	0.83	1	0	0	0	0	0	0	0	0	0	0	0	1	1
1307	KV303_HUMAN	Ig kappa chain V-III region NG9 (Fragment)	Y54	100%	1.00	1	0	0	0	0	0	0	0	0	0	0	0	1	1
1308	IGHM_HUMAN	Ig mu chain C region	S157	98%	0.98	1	0	0	0	0	0	0	0	0	0	0	0	1	1
1309	IGHM_HUMAN	Ig mu chain C region	T160	88%	0.88	1	0	0	0	0	0	0	0	0	0	0	0	1	1
1310	FCGBP_HUMAN	IgGFc-binding protein	S4181	100%	1.00	1	0	1	0	0	0	0	0	0	0	0	0	0	1
1311	IMUP_HUMAN	Immortalization up-regulated protein	S13	44%	0.44	13	1	0	0	0	0	0	0	1	1	0	0	0	3
1312	IMUP_HUMAN	Immortalization up-regulated protein	S29	100%	1.00	426	4	2	2	5	3	5	6	2	2	5	1	2	39
1313	IGBP1_HUMAN	Immunoglobulin-binding protein 1	S147	31%	0.31	1	0	0	0	0	1	0	0	0	0	0	0	0	1
1314	IGBP1_HUMAN	Immunoglobulin-binding protein 1	S268	99%	0.99	1	0	0	0	1	0	0	0	0	0	0	0	0	1
1315	IGBP1_HUMAN	Immunoglobulin-binding protein 1	T264	96%	0.96	1	0	0	0	1	0	0	0	0	0	0	0	0	1
1316	IGBP1_HUMAN	Immunoglobulin-binding protein 1	T266	58%	0.58	1	0	0	0	1	0	0	0	0	0	0	0	0	1
1317	IGBP1_HUMAN	Immunoglobulin-binding protein 1	Y145	59%	0.59	1	0	0	0	0	1	0	0	0	0	0	0	0	1
1318	IGBP1_HUMAN	Immunoglobulin-binding protein 1	Y259	58%	0.58	1	0	0	0	1	0	0	0	0	0	0	0	0	1

1319	IGBP1_HUMAN	Immunoglobulin-binding protein 1	Y271	99%	0.99	1	0	0	0	1	0	0	0	0	0	0	0	0	1
1320	IMA2_HUMAN	Importin subunit alpha-2	S62	99%	0.99	24	1	0	0	0	1	0	0	0	0	0	0	0	2
1321	IMA3_HUMAN	Importin subunit alpha-3	S60	100%	1.00	339	13	8	9	11	7	7	9	7	10	4	3	2	90
1322	IMA7_HUMAN	Importin subunit alpha-7	S6	100%	1.00	28	0	0	0	0	1	0	0	0	0	0	2	0	3
1323	INCE_HUMAN	Inner centromere protein	T292	97%	0.97	2	0	0	0	0	0	0	0	0	0	1	1	0	2
1324	IMDH1_HUMAN	Inosine-5'-monophosphate dehydrogenase 1	S280	16%	0.16	1	0	0	0	0	0	0	0	0	0	1	0	0	1
1325	IMDH1_HUMAN	Inosine-5'-monophosphate dehydrogenase 1	Y258	93%	0.93	1	0	0	0	0	0	0	0	0	0	1	0	0	1
1326	IMDH1_HUMAN	Inosine-5'-monophosphate dehydrogenase 1	Y282	16%	0.16	1	0	0	0	0	0	0	0	0	0	1	0	0	1
1327	ITPR3_HUMAN	Inositol 1,4,5-trisphosphate receptor type 3	S1855	86%	0.86	1	0	1	0	0	0	0	0	0	0	0	0	0	1
1328	ITPR3_HUMAN	Inositol 1,4,5-trisphosphate receptor type 3	S1860	84%	0.84	1	0	1	0	0	0	0	0	0	0	0	0	0	1
1329	ITPR3_HUMAN	Inositol 1,4,5-trisphosphate receptor type 3	S1861	84%	0.84	1	0	1	0	0	0	0	0	0	0	0	0	0	1
1330	ITPR3_HUMAN	Inositol 1,4,5-trisphosphate receptor type 3	S916	100%	1.00	1	0	0	0	0	0	0	0	0	0	0	0	1	1
1331	ITPR3_HUMAN	Inositol 1,4,5-trisphosphate receptor type 3	S934	100%	1.00	6	2	2	0	0	0	0	0	0	0	0	0	0	4
1332	VIP2_HUMAN	Inositol hexakisphosphate and diphosphoinositol-pentakisphosphate kinase 2	S38	100%	1.00	5	2	0	0	0	0	0	0	0	0	0	1	2	5
1333	IRS1_HUMAN	Insulin receptor substrate 1	S3	100%	1.00	25	0	0	0	1	2	1	5	3	4	0	0	0	16
1334	IRS2_HUMAN	Insulin receptor substrate 2	S672	98%	0.98	1	0	0	0	0	1	0	0	0	0	0	0	0	1
1335	IRS2_HUMAN	Insulin receptor substrate 2	Y675	96%	0.96	1	0	0	0	0	1	0	0	0	0	0	0	0	1
1336	INSL3_HUMAN	Insulin-like 3	T26	100%	1.00	1	0	0	0	0	0	0	0	0	0	0	1	0	1

1337	IF2B2_HUMAN	Insulin-like growth factor 2 mRNA-binding protein 2	S162	99%	0.99	17	0	0	0	0	0	0	0	0	0	2	3	2	7
1338	INT1_HUMAN	Integrator complex subunit 1	S1105	70%	0.70	1	0	0	0	0	0	0	0	0	0	0	1	0	1
1339	INT1_HUMAN	Integrator complex subunit 1	S1108	36%	0.36	1	0	0	0	0	0	0	0	0	0	0	1	0	1
1340	INT1_HUMAN	Integrator complex subunit 1	S1110	70%	0.70	1	0	0	0	0	0	0	0	0	0	0	1	0	1
1341	INT1_HUMAN	Integrator complex subunit 1	S1113	25%	0.25	1	0	0	0	0	0	0	0	0	0	0	1	0	1
1342	ITA5_HUMAN	Integrin alpha-5	S127	96%	0.96	18	0	0	0	0	0	0	0	2	0	0	0	0	2
1343	ITB4_HUMAN	Integrin beta-4	S1457	96%	0.96	13	0	0	0	0	0	0	0	0	0	8	4	1	13
1344	ITB4_HUMAN	Integrin beta-4	S1474	20%	0.20	5	0	0	0	0	0	0	0	0	0	2	2	0	4
1345	ITB4_HUMAN	Integrin beta-4	T1455	14%	0.14	1	0	0	0	0	0	0	0	0	0	1	0	0	1
1346	ILKAP_HUMAN	Integrin-linked kinase-associated serine/threonine phosphatase 2C	S13	100%	1.00	531	12	11	12	12	12	10	8	12	12	7	12	9	129
1347	I2BP1_HUMAN	Interferon regulatory factor 2-binding protein 1	S436	100%	1.00	133	2	6	2	6	6	6	5	5	6	2	0	2	48
1348	I2BP2_HUMAN	Interferon regulatory factor 2-binding protein 2	S175	100%	1.00	79	0	0	2	3	1	2	2	1	2	0	0	0	13
1349	I2BP2_HUMAN	Interferon regulatory factor 2-binding protein 2	S360	100%	1.00	52	0	0	2	1	4	3	11	9	7	1	0	3	41
1350	I2BP2_HUMAN	Interferon regulatory factor 2-binding protein 2	S406	94%	0.94	77	0	0	0	3	2	1	2	2	1	0	1	1	13
1351	I2BP2_HUMAN	Interferon regulatory factor 2-binding protein 2	S409	51%	0.51	50	0	0	0	0	3	1	2	2	1	1	0	1	11
1352	I2BP2_HUMAN	Interferon regulatory factor 2-binding protein 2	S71	100%	1.00	117	2	0	5	6	5	4	4	3	3	1	2	4	39
1353	I2BP2_HUMAN	Interferon regulatory factor 2-binding protein 2	T404	53%	0.53	10	1	0	0	1	0	0	0	0	0	0	0	0	2
1354	I2BPL_HUMAN	Interferon regulatory factor 2-binding protein-like	S547	100%	1.00	12	0	0	0	1	0	3	3	1	3	0	0	0	11

1355	IRF3_HUMAN	Interferon regulatory factor 3	S175	50%	0.50	2	0	0	0	0	0	1	0	0	0	0	0	0	1
1356	E2AK2_HUMAN	Interferon-induced, double-stranded RNA-activated protein kinase	S83	96%	0.96	1	0	0	0	0	0	0	0	0	0	1	0	0	1
1357	IFRD1_HUMAN	Interferon-related developmental regulator 1	S383	100%	1.00	1	0	0	0	1	0	0	0	0	0	0	0	0	1
1358	IFRD1_HUMAN	Interferon-related developmental regulator 1	S391	100%	1.00	1	0	0	0	1	0	0	0	0	0	0	0	0	1
1359	IFRD1_HUMAN	Interferon-related developmental regulator 1	T411	100%	1.00	1	0	0	0	1	0	0	0	0	0	0	0	0	1
1360	IFRD1_HUMAN	Interferon-related developmental regulator 1	Y387	100%	1.00	1	0	0	0	1	0	0	0	0	0	0	0	0	1
1361	ILF3_HUMAN	Interleukin enhancer-binding factor 3	S382	100%	1.00	10	0	1	2	1	0	1	0	1	1	2	0	1	10
1362	ILF3_HUMAN	Interleukin enhancer-binding factor 3	S384	91%	0.91	6	0	0	0	0	0	2	0	1	0	0	0	3	6
1363	ILF3_HUMAN	Interleukin enhancer-binding factor 3	S477	10%	0.10	58	1	0	0	0	0	2	1	1	0	1	2	0	8
1364	ILF3_HUMAN	Interleukin enhancer-binding factor 3	S482	97%	0.97	472	10	10	7	11	9	6	9	6	5	5	2	3	83
1365	ILF3_HUMAN	Interleukin enhancer-binding factor 3	T486	63%	0.63	128	4	2	3	4	2	2	3	2	3	1	2	1	29
1366	ILF3_HUMAN	Interleukin enhancer-binding factor 3	T592	100%	1.00	9	0	0	0	0	0	0	0	0	0	3	2	1	6
1367	IL1A_HUMAN	Interleukin-1 alpha	S104	100%	1.00	13	0	0	0	0	1	0	0	0	0	1	0	0	2
1368	IL1A_HUMAN	Interleukin-1 alpha	S87	100%	1.00	78	0	0	0	1	0	0	0	0	0	6	3	3	13
1369	IL1A_HUMAN	Interleukin-1 alpha	S89	17%	0.17	6	0	0	0	0	0	0	0	0	0	0	1	0	1
1370	IL1A_HUMAN	Interleukin-1 alpha	T93	72%	0.72	2	0	0	0	0	1	0	0	0	0	0	0	0	1
1371	I18RA_HUMAN	Interleukin-18 receptor accessory protein	S482	100%	1.00	1	0	0	0	0	0	0	0	0	0	1	0	0	1

1372	I18RA_HUMAN	Interleukin-18 receptor accessory protein	T506	99%	0.99	1	0	0	0	0	0	0	0	0	0	1	0	0	1
1373	I18RA_HUMAN	Interleukin-18 receptor accessory protein	Y485	99%	0.99	1	0	0	0	0	0	0	0	0	0	1	0	0	1
1374	IL5_HUMAN	Interleukin-5	S27	60%	0.60	1	0	0	0	0	0	0	0	1	0	0	0	0	1
1375	IL5_HUMAN	Interleukin-5	T22	79%	0.79	1	0	0	0	0	0	0	0	1	0	0	0	0	1
1376	IL5_HUMAN	Interleukin-5	Y16	60%	0.60	1	0	0	0	0	0	0	0	1	0	0	0	0	1
1377	IL5_HUMAN	Interleukin-5	Y18	69%	0.69	1	0	0	0	0	0	0	0	1	0	0	0	0	1
1378	IFT81_HUMAN	Intraflagellar transport protein 81 homolog	T61	100%	1.00	11	0	0	0	1	0	0	0	0	0	0	0	0	1
1379	INF2_HUMAN	Inverted formin-2	S1147	98%	0.98	41	1	0	0	2	0	1	0	0	2	0	0	0	6
1380	INF2_HUMAN	Inverted formin-2	S1149	100%	1.00	30	0	0	0	0	0	0	1	0	1	0	1	1	4
1381	INF2_HUMAN	Inverted formin-2	T1148	40%	0.40	18	0	1	0	0	0	0	0	1	1	1	0	0	4
1382	INF2_HUMAN	Inverted formin-2	T420	89%	0.89	1	0	0	0	0	0	0	0	0	0	1	0	0	1
1383	RUSC2_HUMAN	Iporin	S525	15%	0.15	1	1	0	0	0	0	0	0	0	0	0	0	0	1
1384	IQUB_HUMAN	IQ and ubiquitin-like domain-containing protein	S305	73%	0.73	1	0	0	0	0	1	0	0	0	0	0	0	0	1
1385	IQUB_HUMAN	IQ and ubiquitin-like domain-containing protein	S316	98%	0.98	1	0	0	0	0	1	0	0	0	0	0	0	0	1
1386	IQUB_HUMAN	IQ and ubiquitin-like domain-containing protein	T301	73%	0.73	1	0	0	0	0	1	0	0	0	0	0	0	0	1
1387	IQUB_HUMAN	IQ and ubiquitin-like domain-containing protein	T303	73%	0.73	1	0	0	0	0	1	0	0	0	0	0	0	0	1
1388	IQUB_HUMAN	IQ and ubiquitin-like domain-containing protein	T304	73%	0.73	1	0	0	0	0	1	0	0	0	0	0	0	0	1
1389	IQUB_HUMAN	IQ and ubiquitin-like domain-containing protein	T306	73%	0.73	1	0	0	0	0	1	0	0	0	0	0	0	0	1

1390	IQUB_HUMAN	IQ and ubiquitin-like domain-containing protein	T309	93%	0.93	1	0	0	0	0	1	0	0	0	0	0	0	0	1
1391	IQUB_HUMAN	IQ and ubiquitin-like domain-containing protein	T319	100%	1.00	1	0	0	0	0	1	0	0	0	0	0	0	0	1
1392	IQCG_HUMAN	IQ domain-containing protein G	S120	91%	0.91	1	0	0	0	0	0	0	0	1	0	0	0	0	1
1393	IQCG_HUMAN	IQ domain-containing protein G	T114	39%	0.39	1	0	0	0	0	0	0	0	1	0	0	0	0	1
1394	IQCG_HUMAN	IQ domain-containing protein G	T124	91%	0.91	1	0	0	0	0	0	0	0	1	0	0	0	0	1
1395	IQEC1_HUMAN	IQ motif and SEC7 domain-containing protein 1	S512	100%	1.00	2	1	0	0	0	1	0	0	0	0	0	0	0	2
1396	IREB2_HUMAN	Iron-responsive element-binding protein 2	S383	55%	0.55	1	0	0	0	0	0	0	0	0	0	0	1	0	1
1397	IREB2_HUMAN	Iron-responsive element-binding protein 2	S401	55%	0.55	1	0	0	0	0	0	0	0	0	0	0	1	0	1
1398	IREB2_HUMAN	Iron-responsive element-binding protein 2	T391	55%	0.55	1	0	0	0	0	0	0	0	0	0	0	1	0	1
1399	IREB2_HUMAN	Iron-responsive element-binding protein 2	T398	55%	0.55	1	0	0	0	0	0	0	0	0	0	0	1	0	1
1400	SYIC_HUMAN	Isoleucyl-tRNA synthetase, cytoplasmic	S1047	89%	0.89	2	0	0	0	0	0	0	0	0	0	1	0	0	1
1401	JKIP3_HUMAN	Janus kinase and microtubule-interacting protein 3	S383	89%	0.89	1	0	0	0	0	0	0	0	1	0	0	0	0	1
1402	KBTBC_HUMAN	Kelch repeat and BTB domain-containing protein 12	T325	62%	0.62	1	0	0	0	0	0	0	1	0	0	0	0	0	1
1403	KBTBC_HUMAN	Kelch repeat and BTB domain-containing protein 12	Y316	58%	0.58	1	0	0	0	0	0	0	1	0	0	0	0	0	1
1404	KLH26_HUMAN	Kelch-like protein 26	S264	99%	0.99	1	0	0	0	0	0	1	0	0	0	0	0	0	1
1405	KLH26_HUMAN	Kelch-like protein 26	T267	97%	0.97	1	0	0	0	0	0	1	0	0	0	0	0	0	1
1406	K1C13_HUMAN	Keratin, type I cytoskeletal 13	S340	100%	1.00	32	1	0	0	0	0	0	0	1	0	0	0	0	2
1407	K1C13_HUMAN	Keratin, type I cytoskeletal 13	T327	100%	1.00	32	1	0	0	0	0	0	0	1	0	0	0	0	2

1408	K1C14_HUMAN	Keratin, type I cytoskeletal 14	S433	12%	0.13	11	0	0	0	0	0	1	0	0	0	0	0	0	1
1409	K1C14_HUMAN	Keratin, type I cytoskeletal 14	S435	100%	1.00	230	1	4	0	12	14	14	1	0	2	5	4	6	63
1410	K1C14_HUMAN	Keratin, type I cytoskeletal 14	S437	12%	0.12	4	0	0	0	1	0	1	0	0	0	0	0	0	2
1411	K1C14_HUMAN	Keratin, type I cytoskeletal 14	S438	75%	0.75	13	0	0	0	2	1	0	0	0	0	0	0	0	3
1412	K1C15_HUMAN	Keratin, type I cytoskeletal 15	S17	52%	0.52	6	0	0	0	0	0	0	0	0	0	0	2	1	3
1413	K1C15_HUMAN	Keratin, type I cytoskeletal 15	S22	100%	1.00	202	0	0	0	0	0	0	2	3	1	9	11	10	36
1414	K1C15_HUMAN	Keratin, type I cytoskeletal 15	S34	100%	1.00	303	0	0	0	0	0	0	6	5	3	15	20	6	55
1415	K1C15_HUMAN	Keratin, type I cytoskeletal 15	S36	100%	1.00	67	0	0	0	0	0	0	1	0	0	3	6	4	14
1416	K1C15_HUMAN	Keratin, type I cytoskeletal 15	S41	37%	0.37	7	0	0	0	0	0	0	0	0	0	0	1	2	3
1417	K1C15_HUMAN	Keratin, type I cytoskeletal 15	S54	79%	0.79	5	0	0	0	0	0	0	0	0	0	1	0	0	1
1418	K1C15_HUMAN	Keratin, type I cytoskeletal 15	S56	100%	1.00	74	0	0	0	0	0	0	0	0	0	10	7	9	26
1419	K1C15_HUMAN	Keratin, type I cytoskeletal 15	T18	33%	0.33	10	0	0	0	0	0	0	0	0	0	0	1	2	3
1420	K1C15_HUMAN	Keratin, type I cytoskeletal 15	T2	13%	0.13	5	0	0	0	0	0	0	0	0	0	0	1	0	1
1421	K1C17_HUMAN	Keratin, type I cytoskeletal 17	S32	100%	1.00	3	0	0	0	0	0	0	0	0	0	1	1	1	3
1422	K1C18_HUMAN	Keratin, type I cytoskeletal 18	S60	100%	1.00	9	0	0	0	0	0	0	0	0	0	2	0	0	2
1423	K1C24_HUMAN	Keratin, type I cytoskeletal 24	S121	46%	0.46	1	0	0	0	0	0	0	0	0	0	1	0	0	1
1424	K1C24_HUMAN	Keratin, type I cytoskeletal 24	S340	79%	0.79	1	0	0	0	0	0	0	0	0	0	0	0	1	1
1425	K1C24_HUMAN	Keratin, type I cytoskeletal 24	S342	79%	0.79	1	0	0	0	0	0	0	0	0	0	0	0	1	1
1426	K1C24_HUMAN	Keratin, type I cytoskeletal 24	T143	80%	0.80	1	0	0	0	0	0	0	0	0	0	1	0	0	1
1427	K1C24_HUMAN	Keratin, type I cytoskeletal 24	Y120	46%	0.46	1	0	0	0	0	0	0	0	0	0	1	0	0	1

1428	K1C24_HUMAN	Keratin, type I cytoskeletal 24	Y122	46%	0.46	1	0	0	0	0	0	0	0	0	0	1	0	0	1
1429	K2C1_HUMAN	Keratin, type II cytoskeletal 1	S604	49%	0.49	1	0	0	0	0	0	1	0	0	0	0	0	0	1
1430	K22O_HUMAN	Keratin, type II cytoskeletal 2 oral	S592	28%	0.28	1	0	0	0	0	0	0	0	1	0	0	0	0	1
1431	K22O_HUMAN	Keratin, type II cytoskeletal 2 oral	S594	29%	0.29	1	0	0	0	0	0	0	0	1	0	0	0	0	1
1432	K22O_HUMAN	Keratin, type II cytoskeletal 2 oral	S596	29%	0.29	1	0	0	0	0	0	0	0	1	0	0	0	0	1
1433	K22O_HUMAN	Keratin, type II cytoskeletal 2 oral	S600	28%	0.28	1	0	0	0	0	0	0	0	1	0	0	0	0	1
1434	K22O_HUMAN	Keratin, type II cytoskeletal 2 oral	S601	29%	0.29	1	0	0	0	0	0	0	0	1	0	0	0	0	1
1435	K22O_HUMAN	Keratin, type II cytoskeletal 2 oral	S602	28%	0.28	1	0	0	0	0	0	0	0	1	0	0	0	0	1
1436	K22O_HUMAN	Keratin, type II cytoskeletal 2 oral	S604	28%	0.28	1	0	0	0	0	0	0	0	1	0	0	0	0	1
1437	K22O_HUMAN	Keratin, type II cytoskeletal 2 oral	S608	28%	0.28	1	0	0	0	0	0	0	0	1	0	0	0	0	1
1438	K22O_HUMAN	Keratin, type II cytoskeletal 2 oral	S611	28%	0.28	1	0	0	0	0	0	0	0	1	0	0	0	0	1
1439	K22O_HUMAN	Keratin, type II cytoskeletal 2 oral	T607	28%	0.28	1	0	0	0	0	0	0	0	1	0	0	0	0	1
1440	K22O_HUMAN	Keratin, type II cytoskeletal 2 oral	Y576	100%	1.00	2	0	0	0	0	0	0	0	1	0	0	0	0	1
1441	K2C5_HUMAN	Keratin, type II cytoskeletal 5	S16	100%	1.00	101	4	3	2	3	4	2	0	1	0	4	3	6	32
1442	K2C5_HUMAN	Keratin, type II cytoskeletal 5	S18	83%	0.83	28	1	2	1	1	2	1	0	0	0	2	3	2	15
1443	K2C5_HUMAN	Keratin, type II cytoskeletal 5	S567	51%	0.51	58	1	3	1	2	0	3	0	0	0	1	4	3	18
1444	K2C5_HUMAN	Keratin, type II cytoskeletal 5	S571	99%	0.99	116	3	1	2	0	5	4	0	0	0	6	3	8	32
1445	K2C5_HUMAN	Keratin, type II cytoskeletal 5	S572	83%	0.83	75	2	2	3	4	1	0	0	0	0	5	4	4	25
1446	K2C5_HUMAN	Keratin, type II cytoskeletal 5	S574	99%	0.99	80	1	5	0	2	3	1	0	0	0	4	5	4	25
1447	K2C5_HUMAN	Keratin, type II cytoskeletal 5	S75	100%	1.00	16	1	4	0	1	6	2	0	0	0	0	0	1	15

1448	K2C5_HUMAN	Keratin, type II cytoskeletal 5	S79	26%	0.26	6	0	0	0	0	0	0	0	0	0	1	2	3	6
1449	K2C5_HUMAN	Keratin, type II cytoskeletal 5	S82	98%	0.98	18	0	0	0	0	0	0	0	0	0	5	4	3	12
1450	K2C5_HUMAN	Keratin, type II cytoskeletal 5	T19	60%	0.60	4	1	1	1	0	0	0	0	0	0	0	0	1	4
1451	K2C5_HUMAN	Keratin, type II cytoskeletal 5	T78	11%	0.11	2	0	0	0	0	0	0	0	0	0	0	2	0	2
1452	K2C6A_HUMAN	Keratin, type II cytoskeletal 6A	S19	100%	1.00	11	0	0	0	0	1	0	0	0	0	3	2	5	11
1453	K2C6A_HUMAN	Keratin, type II cytoskeletal 6A	S22	100%	1.00	4	0	0	0	0	0	0	0	0	0	1	2	1	4
1454	K2C6A_HUMAN	Keratin, type II cytoskeletal 6A	S44	100%	1.00	72	0	0	0	3	0	2	0	0	0	2	0	1	8
1455	K2C6A_HUMAN	Keratin, type II cytoskeletal 6A	S540	27%	0.27	1	0	0	0	0	0	0	0	0	0	0	1	0	1
1456	K2C6A_HUMAN	Keratin, type II cytoskeletal 6A	S541	37%	0.37	1	0	0	0	0	0	0	0	0	0	1	0	0	1
1457	K2C6A_HUMAN	Keratin, type II cytoskeletal 6A	S546	60%	0.60	6	0	0	0	0	0	0	0	0	0	1	2	3	6
1458	K2C6A_HUMAN	Keratin, type II cytoskeletal 6A	S547	22%	0.22	6	0	0	0	0	0	0	0	0	0	0	1	2	3
1459	K2C6A_HUMAN	Keratin, type II cytoskeletal 6A	S71	100%	1.00	114	0	0	0	0	1	0	0	0	0	0	0	0	1
1460	K2C6A_HUMAN	Keratin, type II cytoskeletal 6A	S76	56%	0.56	2	0	0	0	0	0	0	0	0	0	1	0	0	1
1461	K2C6A_HUMAN	Keratin, type II cytoskeletal 6A	T548	60%	0.60	3	0	0	0	0	0	0	0	0	0	0	0	1	1
1462	K2C7_HUMAN	Keratin, type II cytoskeletal 7	S12	8%	0.08	5	0	0	0	1	1	1	0	0	0	0	0	0	3
1463	K2C7_HUMAN	Keratin, type II cytoskeletal 7	S2	100%	1.00	73	0	4	0	0	1	0	0	0	0	7	5	3	20
1464	K2C7_HUMAN	Keratin, type II cytoskeletal 7	S6	20%	0.20	43	1	0	0	0	0	0	1	0	0	2	0	2	6
1465	K2C7_HUMAN	Keratin, type II cytoskeletal 7	S7	99%	0.99	389	7	8	9	6	5	5	5	6	4	7	10	8	80
1466	K2C7_HUMAN	Keratin, type II cytoskeletal 7	T11	31%	0.31	4	0	0	0	0	0	0	1	0	0	0	0	0	1
1467	K2C79_HUMAN	Keratin, type II cytoskeletal 79	T368	100%	1.00	29	1	3	1	1	0	0	0	0	0	0	0	0	6

1468	K2C8_HUMAN	Keratin, type II cytoskeletal 8	S330	100%	1.00	15	0	0	0	0	0	0	0	0	0	0	1	0	1
1469	K2C8_HUMAN	Keratin, type II cytoskeletal 8	S475	100%	1.00	2	0	0	0	0	0	0	0	0	0	2	0	0	2
1470	K2C8_HUMAN	Keratin, type II cytoskeletal 8	S74	100%	1.00	120	0	0	0	0	0	0	0	0	0	11	13	5	29
1471	KHDR1_HUMAN	KH domain-containing, RNA-binding, signal transduction-associated protein 1	S58	82%	0.82	21	1	1	1	1	0	1	1	0	0	0	0	1	7
1472	KHDR1_HUMAN	KH domain-containing, RNA-binding, signal transduction-associated protein 1	T61	11%	0.11	9	1	1	0	0	0	0	0	0	1	0	0	1	4
1473	KLC1_HUMAN	Kinesin light chain 1	S101	81%	0.81	1	0	0	1	0	0	0	0	0	0	0	0	0	1
1474	KLC1_HUMAN	Kinesin light chain 1	S93	93%	0.93	1	0	0	1	0	0	0	0	0	0	0	0	0	1
1475	KLC2_HUMAN	Kinesin light chain 2	S582	100%	1.00	7	0	0	0	0	0	0	0	0	0	2	3	2	7
1476	KLC3_HUMAN	Kinesin light chain 3	S173	100%	1.00	27	0	0	0	1	0	0	0	0	0	2	0	0	3
1477	KLC3_HUMAN	Kinesin light chain 3	S466	100%	1.00	66	6	9	6	0	0	2	0	0	0	5	4	0	32
1478	KLC4_HUMAN	Kinesin light chain 4	S590	97%	0.97	4	0	0	0	0	0	0	1	0	0	0	0	0	1
1479	KIF1B_HUMAN	Kinesin-like protein KIF1B	S1487	100%	1.00	3	0	1	1	0	0	0	0	0	0	0	0	0	2
1480	KI21A_HUMAN	Kinesin-like protein KIF21A	S1382	78%	0.78	1	0	0	0	0	1	0	0	0	0	0	0	0	1
1481	KI21A_HUMAN	Kinesin-like protein KIF21A	S853	17%	0.17	4	0	0	0	0	0	0	0	0	0	1	0	0	1
1482	KI21A_HUMAN	Kinesin-like protein KIF21A	S854	15%	0.15	2	0	0	0	0	1	0	0	0	0	0	0	1	2
1483	KI21A_HUMAN	Kinesin-like protein KIF21A	S855	41%	0.41	19	1	0	1	2	1	1	0	1	0	1	2	1	11
1484	KI21A_HUMAN	Kinesin-like protein KIF21A	T1376	83%	0.83	1	0	0	0	0	1	0	0	0	0	0	0	0	1
1485	KI21A_HUMAN	Kinesin-like protein KIF21A	T862	95%	0.95	19	0	2	1	0	0	2	0	0	0	0	0	0	5

1486	KI21A_HUMAN	Kinesin-like protein KIF21A	Y1395	54%	0.54	1	0	0	0	0	1	0	0	0	0	0	0	0	1
1487	KI21A_HUMAN	Kinesin-like protein KIF21A	Y1409	21%	0.21	1	0	0	0	0	1	0	0	0	0	0	0	0	1
1488	KIF24_HUMAN	Kinesin-like protein KIF24	S170	62%	0.62	1	0	0	0	0	0	0	0	0	1	0	0	0	1
1489	KIF24_HUMAN	Kinesin-like protein KIF24	S173	62%	0.62	1	0	0	0	0	0	0	0	0	1	0	0	0	1
1490	KIF24_HUMAN	Kinesin-like protein KIF24	S178	62%	0.62	1	0	0	0	0	0	0	0	0	1	0	0	0	1
1491	KIF24_HUMAN	Kinesin-like protein KIF24	T151	62%	0.62	1	0	0	0	0	0	0	0	0	1	0	0	0	1
1492	KIF24_HUMAN	Kinesin-like protein KIF24	T157	62%	0.62	1	0	0	0	0	0	0	0	0	1	0	0	0	1
1493	KIF24_HUMAN	Kinesin-like protein KIF24	Y162	71%	0.71	1	0	0	0	0	0	0	0	0	1	0	0	0	1
1494	KIF24_HUMAN	Kinesin-like protein KIF24	Y176	62%	0.62	1	0	0	0	0	0	0	0	0	1	0	0	0	1
1495	KI26B_HUMAN	Kinesin-like protein KIF26B	T881	100%	1.00	1	0	0	0	0	0	0	0	0	0	0	1	0	1
1496	KIF2A_HUMAN	Kinesin-like protein KIF2A	T78	31%	0.31	2	0	0	0	0	0	0	0	0	0	0	1	0	1
1497	KIF2B_HUMAN	Kinesin-like protein KIF2B	S497	100%	1.00	8	0	3	1	2	0	0	0	0	0	0	0	2	8
1498	KIFC1_HUMAN	Kinesin-like protein KIFC1	S6	100%	1.00	6	0	0	0	0	0	0	0	0	0	0	0	1	1
1499	KANK1_HUMAN	KN motif and ankyrin repeat domain-containing protein 1	S186	100%	1.00	57	0	0	0	1	3	0	2	0	1	6	7	6	26
1500	KANK1_HUMAN	KN motif and ankyrin repeat domain-containing protein 1	S325	73%	0.73	16	0	0	0	0	0	0	0	0	0	3	2	0	5
1501	KLF16_HUMAN	Krueppel-like factor 16	S52	91%	0.91	15	2	0	0	1	1	0	0	0	0	2	0	0	6
1502	KLF16_HUMAN	Krueppel-like factor 16	T55	92%	0.92	3	0	0	0	0	1	0	0	0	0	0	0	0	1
1503	LPH_HUMAN	Lactase-phlorizin hydrolase	S329	35%	0.35	1	0	0	0	1	0	0	0	0	0	0	0	0	1
1504	LPH_HUMAN	Lactase-phlorizin hydrolase	S331	35%	0.35	1	0	0	0	1	0	0	0	0	0	0	0	0	1
1505	LPH_HUMAN	Lactase-phlorizin hydrolase	S353	100%	1.00	1	0	0	0	1	0	0	0	0	0	0	0	0	1

1506	LPH_HUMAN	Lactase-phlorizin hydrolase	S354	100%	1.00	1	0	0	0	1	0	0	0	0	0	0	0	0	1
1507	LPH_HUMAN	Lactase-phlorizin hydrolase	S357	100%	1.00	1	0	0	0	1	0	0	0	0	0	0	0	0	1
1508	LPH_HUMAN	Lactase-phlorizin hydrolase	T350	100%	1.00	1	0	0	0	1	0	0	0	0	0	0	0	0	1
1509	LPH_HUMAN	Lactase-phlorizin hydrolase	T351	100%	1.00	1	0	0	0	1	0	0	0	0	0	0	0	0	1
1510	LPH_HUMAN	Lactase-phlorizin hydrolase	Y359	100%	1.00	1	0	0	0	1	0	0	0	0	0	0	0	0	1
1511	LAD1_HUMAN	Ladinin-1	S177	100%	1.00	1	0	0	0	0	0	0	0	0	0	0	0	1	1
1512	LAD1_HUMAN	Ladinin-1	S269	8%	0.08	1	0	0	0	0	0	0	0	0	0	1	0	0	1
1513	LAD1_HUMAN	Ladinin-1	S272	99%	0.99	8	0	0	0	0	0	0	0	0	0	2	1	5	8
1514	LAD1_HUMAN	Ladinin-1	S301	92%	0.92	29	0	0	0	0	0	0	0	0	0	3	6	4	13
1515	LAD1_HUMAN	Ladinin-1	S356	100%	1.00	8	0	1	0	0	0	1	0	0	0	2	2	1	7
1516	LAD1_HUMAN	Ladinin-1	S38	100%	1.00	19	0	0	0	0	0	0	0	0	0	6	4	7	17
1517	LAD1_HUMAN	Ladinin-1	S64	100%	1.00	270	0	0	1	7	7	4	1	0	0	11	13	16	60
1518	LAD1_HUMAN	Ladinin-1	S78	100%	1.00	27	0	0	0	0	0	1	0	0	0	1	1	2	5
1519	LAD1_HUMAN	Ladinin-1	T274	53%	0.53	2	0	0	0	0	0	0	0	0	0	1	1	0	2
1520	LAD1_HUMAN	Ladinin-1	T305	94%	0.94	10	0	0	0	0	0	0	0	0	0	0	0	1	1
1521	LAP2A_HUMAN	Lamina-associated polypeptide 2, isoform alpha	S424	100%	1.00	2	0	0	0	0	0	0	0	0	0	0	1	1	2
1522	LAP2A_HUMAN	Lamina-associated polypeptide 2, isoform alpha	S66	100%	1.00	174	6	7	1	3	6	5	7	5	8	3	3	2	56
1523	LAP2A_HUMAN	Lamina-associated polypeptide 2, isoform alpha	S67	100%	1.00	280	10	9	8	6	4	12	8	7	11	4	5	4	88
1524	LAP2A_HUMAN	Lamina-associated polypeptide 2, isoform alpha	T74	100%	1.00	227	10	11	2	1	5	6	5	8	7	10	6	6	77

1525	LAP2B_HUMAN	Lamina-associated polypeptide 2, isoforms beta/gamma	S306	100%	1.00	1	0	0	0	0	0	0	0	0	0	0	1	0	1
1526	LAP2B_HUMAN	Lamina-associated polypeptide 2, isoforms beta/gamma	S66	25%	0.25	3	0	0	0	0	0	0	0	0	0	0	3	0	3
1527	LAP2B_HUMAN	Lamina-associated polypeptide 2, isoforms beta/gamma	S67	60%	0.60	5	0	0	0	0	0	0	0	0	0	0	5	0	5
1528	LAP2B_HUMAN	Lamina-associated polypeptide 2, isoforms beta/gamma	T74	100%	1.00	6	0	0	0	0	0	0	0	0	0	0	6	0	6
1529	LMNB2_HUMAN	Lamin-B2	S268	73%	0.73	1	0	0	0	0	0	0	0	0	0	0	1	0	1
1530	LAMB3_HUMAN	Laminin subunit beta-3	S79	7%	0.07	1	0	1	0	0	0	0	0	0	0	0	0	0	1
1531	LAMB3_HUMAN	Laminin subunit beta-3	Y77	33%	0.33	1	0	0	0	0	0	0	1	0	0	0	0	0	1
1532	LAMB3_HUMAN	Laminin subunit beta-3	Y78	75%	0.75	2	0	1	0	0	0	0	1	0	0	0	0	0	2
1533	LARP1_HUMAN	La-related protein 1	S143	100%	1.00	49	3	0	1	1	3	1	0	1	1	0	0	0	11
1534	LARP1_HUMAN	La-related protein 1	S546	89%	0.89	23	0	0	0	1	0	1	1	1	0	0	2	3	9
1535	LARP1_HUMAN	La-related protein 1	S548	96%	0.96	407	8	8	8	5	6	3	9	5	9	8	8	3	80
1536	LARP1_HUMAN	La-related protein 1	S75	100%	1.00	38	2	1	1	1	0	0	0	0	1	1	1	2	10
1537	LARP1_HUMAN	La-related protein 1	S824	48%	0.48	3	0	0	0	0	0	0	0	0	0	0	0	3	3
1538	LARP1_HUMAN	La-related protein 1	S90	100%	1.00	848	25	27	21	18	22	21	21	25	18	13	12	14	237
1539	LARP1_HUMAN	La-related protein 1	T128	100%	1.00	2	1	0	0	0	0	0	0	0	0	0	0	0	1
1540	LARP1_HUMAN	La-related protein 1	T526	100%	1.00	61	4	2	0	6	2	4	3	3	5	5	3	1	38
1541	LARP4_HUMAN	La-related protein 4	S583	100%	1.00	40	0	0	0	0	0	0	0	0	1	1	4	2	8
1542	LARP4_HUMAN	La-related protein 4	S597	66%	0.66	23	1	0	0	0	1	0	0	0	0	2	0	0	4

1543	LARP4_HUMAN	La-related protein 4	T587	7%	0.07	1	0	1	0	0	0	0	0	0	0	0	0	0	1
1544	LARP4_HUMAN	La-related protein 4	T595	25%	0.25	1	0	1	0	0	0	0	0	0	0	0	0	0	1
1545	LAR4B_HUMAN	La-related protein 4B	S487	11%	0.11	1	0	0	0	0	0	0	0	0	0	1	0	0	1
1546	LAR4B_HUMAN	La-related protein 4B	S524	94%	0.94	49	2	2	0	1	0	1	2	1	1	5	2	0	17
1547	BAG6_HUMAN	Large proline-rich protein BAG6	S100	41%	0.41	1	0	0	0	0	1	0	0	0	0	0	0	0	1
1548	BAG6_HUMAN	Large proline-rich protein BAG6	S104	14%	0.14	5	0	0	0	0	1	0	0	0	1	1	0	0	3
1549	BAG6_HUMAN	Large proline-rich protein BAG6	S106	21%	0.21	3	0	0	0	0	0	0	0	1	0	0	1	0	2
1550	BAG6_HUMAN	Large proline-rich protein BAG6	S113	100%	1.00	293	14	10	9	8	10	7	8	6	5	5	7	8	97
1551	BAG6_HUMAN	Large proline-rich protein BAG6	S964	100%	1.00	18	1	1	0	1	0	0	2	1	1	0	0	0	7
1552	BAG6_HUMAN	Large proline-rich protein BAG6	S973	100%	1.00	6	0	3	0	0	0	0	0	0	0	1	0	0	4
1553	BAG6_HUMAN	Large proline-rich protein BAG6	S99	47%	0.47	1	0	0	0	0	1	0	0	0	0	0	0	0	1
1554	BAG6_HUMAN	Large proline-rich protein BAG6	T108	40%	0.40	15	0	0	0	0	0	0	0	1	0	0	0	1	2
1555	BAG6_HUMAN	Large proline-rich protein BAG6	T117	92%	0.92	21	0	0	1	0	1	1	0	1	0	0	0	0	4
1556	AVL9_HUMAN	Late secretory pathway protein AVL9 homolog	S246	11%	0.11	2	0	0	0	0	0	0	0	0	0	2	0	0	2
1557	LPHN1_HUMAN	Latrophilin-1	S546	69%	0.69	2	0	0	0	0	0	0	0	0	0	0	1	1	2
1558	LPHN1_HUMAN	Latrophilin-1	T561	72%	0.72	2	0	0	0	0	0	0	0	0	0	0	1	1	2
1559	LMIP_HUMAN	Lens fiber membrane intrinsic protein	S3	82%	0.82	1	0	0	0	0	0	0	0	0	0	0	1	0	1
1560	LMIP_HUMAN	Lens fiber membrane intrinsic protein	T16	88%	0.88	1	0	0	0	0	0	0	0	0	0	0	1	0	1
1561	LMIP_HUMAN	Lens fiber membrane intrinsic protein	T25	74%	0.74	1	0	0	0	0	0	0	0	0	0	0	1	0	1

1562	LMIP_HUMAN	Lens fiber membrane intrinsic protein	Y2	82%	0.82	1	0	0	0	0	0	0	0	0	0	0	1	0	1
1563	LMBL3_HUMAN	Lethal(3)malignant brain tumor-like protein 3	S17	92%	0.92	3	0	1	0	0	0	0	0	0	0	0	0	0	1
1564	LMBL3_HUMAN	Lethal(3)malignant brain tumor-like protein 3	S31	78%	0.78	3	0	1	0	0	0	0	0	0	0	0	0	0	1
1565	LMBL3_HUMAN	Lethal(3)malignant brain tumor-like protein 3	S6	95%	0.95	3	0	1	0	0	0	0	0	0	0	0	0	0	1
1566	LMBL3_HUMAN	Lethal(3)malignant brain tumor-like protein 3	S7	93%	0.93	3	0	1	0	0	0	0	0	0	0	0	0	0	1
1567	LMBL3_HUMAN	Lethal(3)malignant brain tumor-like protein 3	S9	94%	0.94	3	0	1	0	0	0	0	0	0	0	0	0	0	1
1568	LMBL3_HUMAN	Lethal(3)malignant brain tumor-like protein 3	T2	94%	0.94	3	0	1	0	0	0	0	0	0	0	0	0	0	1
1569	LMBL3_HUMAN	Lethal(3)malignant brain tumor-like protein 3	T27	78%	0.78	3	0	1	0	0	0	0	0	0	0	0	0	0	1
1570	LMBL3_HUMAN	Lethal(3)malignant brain tumor-like protein 3	T8	93%	0.93	3	0	1	0	0	0	0	0	0	0	0	0	0	1
1571	LUZP1_HUMAN	Leucine zipper protein 1	S659	100%	1.00	89	5	3	3	4	2	2	4	2	3	2	1	1	32
1572	LRCH4_HUMAN	Leucine-rich repeat and calponin homology domain-containing protein 4	S25	97%	0.97	11	0	0	0	0	0	0	0	0	0	0	1	0	1
1573	LRFN6_HUMAN	Leucine-rich repeat and fibronectin type-III domain-containing protein 6	S741	100%	1.00	8	0	0	0	0	0	0	0	0	0	2	1	1	4
1574	LRFN6_HUMAN	Leucine-rich repeat and fibronectin type-III domain-containing protein 6	S744	40%	0.40	9	1	0	0	0	1	0	0	0	0	0	0	0	2
1575	LRFN6_HUMAN	Leucine-rich repeat and fibronectin type-III domain-containing protein 6	S747	100%	1.00	8	0	0	0	0	0	0	0	0	0	2	1	1	4
1576	LRFN6_HUMAN	Leucine-rich repeat and fibronectin type-III domain-containing protein 6	Y743	82%	0.82	9	1	0	0	0	1	0	0	0	0	0	0	0	2

1577	LRWD1_HUMAN	Leucine-rich repeat and WD repeat-containing protein 1	S243	100%	1.00	206	2	6	6	5	6	5	3	2	3	5	7	5	55
1578	LRWD1_HUMAN	Leucine-rich repeat and WD repeat-containing protein 1	S245	97%	0.97	23	0	0	0	0	0	1	1	0	1	1	1	1	6
1579	LRWD1_HUMAN	Leucine-rich repeat and WD repeat-containing protein 1	S251	89%	0.89	22	2	0	0	1	1	0	3	0	1	0	1	0	9
1580	LRWD1_HUMAN	Leucine-rich repeat and WD repeat-containing protein 1	S253	42%	0.42	8	1	1	0	0	0	0	0	0	0	0	0	1	3
1581	LRRF1_HUMAN	Leucine-rich repeat flightless-interacting protein 1	S115	90%	0.90	12	1	0	0	1	0	0	0	0	0	1	0	0	3
1582	LRRF1_HUMAN	Leucine-rich repeat flightless-interacting protein 1	S116	99%	0.99	147	4	3	5	2	4	3	3	4	3	3	4	4	42
1583	LRRF1_HUMAN	Leucine-rich repeat flightless-interacting protein 1	S120	100%	1.00	151	1	2	2	1	6	3	15	4	1	12	14	7	68
1584	LRRF1_HUMAN	Leucine-rich repeat flightless-interacting protein 1	S124	100%	1.00	36	0	0	1	0	1	1	2	1	2	5	5	10	28
1585	LRRF1_HUMAN	Leucine-rich repeat flightless-interacting protein 1	S126	95%	0.95	14	0	0	1	0	1	0	1	1	2	2	1	1	10
1586	LRRF1_HUMAN	Leucine-rich repeat flightless-interacting protein 1	S714	99%	0.99	1	0	0	0	0	0	0	0	0	0	0	0	1	1
1587	LRRF1_HUMAN	Leucine-rich repeat flightless-interacting protein 1	S735	98%	0.98	39	3	2	0	1	2	1	0	0	0	3	3	2	17
1588	LRRF1_HUMAN	Leucine-rich repeat flightless-interacting protein 1	T123	80%	0.80	20	0	2	1	0	0	0	1	0	0	2	2	2	10
1589	LRRF1_HUMAN	Leucine-rich repeat flightless-interacting protein 1	T129	5%	0.05	2	0	0	0	0	0	0	0	0	0	1	0	0	1
1590	LRRF1_HUMAN	Leucine-rich repeat flightless-interacting protein 1	T727	9%	0.09	1	0	0	1	0	0	0	0	0	0	0	0	0	1
1591	LRRF2_HUMAN	Leucine-rich repeat flightless-interacting protein 2	S328	100%	1.00	221	2	2	2	3	1	4	2	0	1	6	4	6	33
1592	LRRF2_HUMAN	Leucine-rich repeat flightless-interacting protein 2	S332	85%	0.85	41	1	2	0	1	1	0	2	1	3	2	1	2	16
1593	LRRF2_HUMAN	Leucine-rich repeat flightless-interacting protein 2	S333	84%	0.84	82	0	1	3	2	2	2	2	3	2	4	4	2	27

1594	LRRF2_HUMAN	Leucine-rich repeat flightless-interacting protein 2	T331	50%	0.50	37	0	0	1	1	1	0	0	0	0	0	0	0	3
1595	LR16A_HUMAN	Leucine-rich repeat-containing protein 16A	S640	95%	0.95	1	0	0	0	0	0	0	0	0	0	0	1	0	1
1596	LR16A_HUMAN	Leucine-rich repeat-containing protein 16A	S967	1%	0.01	1	0	0	0	1	0	0	0	0	0	0	0	0	1
1597	LR16A_HUMAN	Leucine-rich repeat-containing protein 16A	S968	100%	1.00	14	0	0	0	0	1	0	0	1	1	0	1	0	4
1598	LR37B_HUMAN	Leucine-rich repeat-containing protein 37B	S19	100%	1.00	1	0	0	0	0	0	0	0	0	0	0	0	1	1
1599	LR37B_HUMAN	Leucine-rich repeat-containing protein 37B	S2	100%	1.00	1	0	0	0	0	0	0	0	0	0	0	0	1	1
1600	LR37B_HUMAN	Leucine-rich repeat-containing protein 37B	T14	100%	1.00	1	0	0	0	0	0	0	0	0	0	0	0	1	1
1601	LRC45_HUMAN	Leucine-rich repeat-containing protein 45	S227	93%	0.93	1	1	0	0	0	0	0	0	0	0	0	0	0	1
1602	LRC47_HUMAN	Leucine-rich repeat-containing protein 47	S518	27%	0.27	7	0	0	0	1	0	1	0	0	0	0	0	0	2
1603	LRC47_HUMAN	Leucine-rich repeat-containing protein 47	S520	70%	0.70	45	3	0	2	1	2	1	2	1	2	1	0	0	15
1604	LRC47_HUMAN	Leucine-rich repeat-containing protein 47	T510	10%	0.10	5	1	0	0	0	0	0	0	1	0	0	0	0	2
1605	LRC47_HUMAN	Leucine-rich repeat-containing protein 47	T522	52%	0.52	7	0	0	0	0	0	0	2	0	0	1	0	0	3
1606	LRC67_HUMAN	Leucine-rich repeat-containing protein 67	S265	79%	0.79	1	0	0	0	0	0	1	0	0	0	0	0	0	1
1607	LRC67_HUMAN	Leucine-rich repeat-containing protein 67	S268	41%	0.41	1	0	0	0	0	0	1	0	0	0	0	0	0	1
1608	LRIG2_HUMAN	Leucine-rich repeats and immunoglobulin-like domains protein 2	S523	13%	0.13	1	0	0	0	0	0	0	0	0	0	0	1	0	1
1609	LRIG2_HUMAN	Leucine-rich repeats and immunoglobulin-like domains protein 2	S524	26%	0.26	1	0	0	0	0	0	0	0	0	0	0	1	0	1

1610	LRIG2_HUMAN	Leucine-rich repeats and immunoglobulin-like domains protein 2	T518	94%	0.94	1	0	0	0	0	0	0	0	0	0	0	1	0	1
1611	LRIG2_HUMAN	Leucine-rich repeats and immunoglobulin-like domains protein 2	T520	46%	0.46	1	0	0	0	0	0	0	0	0	0	0	1	0	1
1612	LEUK_HUMAN	Leukosialin	S37	84%	0.84	1	0	0	0	0	0	0	0	1	0	0	0	0	1
1613	LEUK_HUMAN	Leukosialin	S41	87%	0.87	1	0	0	0	0	0	0	0	1	0	0	0	0	1
1614	LEUK_HUMAN	Leukosialin	S42	78%	0.78	1	0	0	0	0	0	0	0	1	0	0	0	0	1
1615	LEUK_HUMAN	Leukosialin	T3	11%	0.11	1	0	0	0	0	0	0	0	1	0	0	0	0	1
1616	LIMC1_HUMAN	LIM and calponin homology domains-containing protein 1	S718	100%	1.00	245	0	2	2	2	0	0	20	20	18	2	2	4	72
1617	LIMC1_HUMAN	LIM and calponin homology domains-containing protein 1	T724	99%	0.99	2	0	0	0	0	0	0	0	1	0	0	0	0	1
1618	LASP1_HUMAN	LIM and SH3 domain protein 1	T104	100%	1.00	2	0	1	0	0	0	0	0	0	0	0	0	0	1
1619	LIMA1_HUMAN	LIM domain and actin-binding protein 1	S132	100%	1.00	57	0	0	0	0	0	0	0	0	0	7	9	9	25
1620	LIMA1_HUMAN	LIM domain and actin-binding protein 1	S225	36%	0.36	3	0	0	0	0	0	0	0	0	0	1	1	1	3
1621	LIMA1_HUMAN	LIM domain and actin-binding protein 1	S228	74%	0.74	1	0	0	0	0	0	0	0	0	0	1	0	0	1
1622	LIMA1_HUMAN	LIM domain and actin-binding protein 1	S490	100%	1.00	190	3	5	1	6	6	5	5	2	1	9	7	7	57
1623	LIMA1_HUMAN	LIM domain and actin-binding protein 1	S686	100%	1.00	184	3	2	5	5	5	5	0	2	1	6	7	6	47
1624	LIMA1_HUMAN	LIM domain and actin-binding protein 1	S692	100%	1.00	18	0	1	1	1	0	2	0	0	0	0	0	0	5
1625	LIMA1_HUMAN	LIM domain and actin-binding protein 1	T487	100%	1.00	6	0	1	0	1	0	0	0	0	1	1	0	0	4
1626	LMO7_HUMAN	LIM domain only protein 7	S1026	100%	1.00	7	0	0	0	0	0	0	0	0	0	3	2	0	5

1627	LMO7_HUMAN	LIM domain only protein 7	S1493	33%	0.33	2	0	0	0	0	0	0	0	0	0	0	1	0	1
1628	LMO7_HUMAN	LIM domain only protein 7	S246	99%	0.99	8	0	0	1	0	0	0	0	0	0	0	0	0	1
1629	LMO7_HUMAN	LIM domain only protein 7	S805	100%	1.00	3	0	0	0	0	0	0	0	0	0	2	0	1	3
1630	LMO7_HUMAN	LIM domain only protein 7	S867	100%	1.00	23	0	0	0	0	0	0	0	0	0	0	2	1	3
1631	LMO7_HUMAN	LIM domain only protein 7	S988	96%	0.96	19	0	0	0	0	0	0	0	0	0	3	1	0	4
1632	LMO7_HUMAN	LIM domain only protein 7	S991	93%	0.93	2	0	0	0	0	0	0	0	0	0	1	1	0	2
1633	LMO7_HUMAN	LIM domain only protein 7	T990	31%	0.31	7	0	0	0	0	0	0	0	0	0	0	3	0	3
1634	LIMD1_HUMAN	LIM domain-containing protein 1	S272	100%	1.00	1	0	1	0	0	0	0	0	0	0	0	0	0	1
1635	LIMD1_HUMAN	LIM domain-containing protein 1	S384	98%	0.98	68	0	0	0	0	0	1	0	0	0	2	1	1	5
1636	LIMD1_HUMAN	LIM domain-containing protein 1	S387	82%	0.82	26	0	0	0	0	0	0	0	0	1	2	1	1	5
1637	LIMD1_HUMAN	LIM domain-containing protein 1	T386	24%	0.24	6	0	0	0	0	0	0	0	0	0	0	1	0	1
1638	LMBR1_HUMAN	Limb region 1 protein homolog	S8	33%	0.33	1	0	1	0	0	0	0	0	0	0	0	0	0	1
1639	LCNL1_HUMAN	Lipocalin-like 1 protein	S6	98%	0.98	1	1	0	0	0	0	0	0	0	0	0	0	0	1
1640	LSR_HUMAN	Lipolysis-stimulated lipoprotein receptor	S493	100%	1.00	92	7	5	4	3	4	5	5	2	1	3	1	1	41
1641	LSR_HUMAN	Lipolysis-stimulated lipoprotein receptor	T501	0%	0.00	2	0	0	0	0	0	1	0	1	0	0	0	0	2
1642	LRBA_HUMAN	Lipopolysaccharide-responsive and beige-like anchor protein	S10	100%	1.00	3	0	0	0	0	0	0	3	0	0	0	0	0	3
1643	LRBA_HUMAN	Lipopolysaccharide-responsive and beige-like anchor protein	S1051	39%	0.39	8	1	0	0	0	0	1	0	0	0	1	0	0	3
1644	LRBA_HUMAN	Lipopolysaccharide-responsive and beige-like anchor protein	S1052	55%	0.55	37	2	3	3	1	3	1	0	0	0	1	4	0	18

1645	LIPT_HUMAN	Lipoyltransferase 1, mitochondrial	S101	89%	0.89	1	0	0	0	0	0	1	0	0	0	0	0	0	1
1646	LIPT_HUMAN	Lipoyltransferase 1, mitochondrial	T105	91%	0.91	1	0	0	0	0	0	1	0	0	0	0	0	0	1
1647	LIPT_HUMAN	Lipoyltransferase 1, mitochondrial	T119	66%	0.66	1	0	0	0	0	0	1	0	0	0	0	0	0	1
1648	LIPT_HUMAN	Lipoyltransferase 1, mitochondrial	T120	66%	0.66	1	0	0	0	0	0	1	0	0	0	0	0	0	1
1649	LIPT_HUMAN	Lipoyltransferase 1, mitochondrial	Y107	93%	0.93	1	0	0	0	0	0	1	0	0	0	0	0	0	1
1650	LIPA1_HUMAN	Liprin-alpha-1	S275	60%	0.60	2	0	0	0	1	0	0	0	0	0	0	0	0	1
1651	LIPA1_HUMAN	Liprin-alpha-1	S42	10%	0.10	2	0	0	0	0	0	0	0	0	0	1	0	0	1
1652	K1468_HUMAN	LisH domain and HEAT repeat-containing protein KIAA1468	S180	100%	1.00	4	0	0	0	0	0	0	0	0	2	0	0	2	4
1653	LA_HUMAN	Lupus La protein	S366	100%	1.00	246	19	15	15	12	15	14	16	15	17	9	10	13	170
1654	LY65C_HUMAN	Lymphocyte antigen 6 complex locus protein G5c	S21	13%	0.13	1	0	0	0	1	0	0	0	0	0	0	0	0	1
1655	LY65C_HUMAN	Lymphocyte antigen 6 complex locus protein G5c	T27	5%	0.05	1	0	0	0	0	0	1	0	0	0	0	0	0	1
1656	KDM5C_HUMAN	Lysine-specific demethylase 5C	S1359	100%	1.00	3	0	0	0	1	0	0	0	0	0	0	0	0	1
1657	KDM6A_HUMAN	Lysine-specific demethylase 6A	S1400	50%	0.50	1	0	0	0	0	0	0	0	0	0	1	0	0	1
1658	KDM6A_HUMAN	Lysine-specific demethylase 6A	S1401	50%	0.50	1	0	0	0	0	0	0	0	0	0	1	0	0	1
1659	KDM6A_HUMAN	Lysine-specific demethylase 6A	T1391	100%	1.00	1	0	0	0	0	0	0	0	0	0	1	0	0	1
1660	KDM1A_HUMAN	Lysine-specific histone demethylase 1A	S166	100%	1.00	30	3	0	0	2	0	0	2	0	0	0	0	0	7
1661	KDM1A_HUMAN	Lysine-specific histone demethylase 1A	S93	40%	0.40	1	0	0	1	0	0	0	0	0	0	0	0	0	1
1662	KDM1A_HUMAN	Lysine-specific histone demethylase 1A	T104	96%	0.96	1	0	0	1	0	0	0	0	0	0	0	0	0	1

1663	KDM1A_HUMAN	Lysine-specific histone demethylase 1A	T95	56%	0.56	1	0	0	1	0	0	0	0	0	0	0	0	0	1
1664	KDM1A_HUMAN	Lysine-specific histone demethylase 1A	T99	40%	0.40	1	0	0	1	0	0	0	0	0	0	0	0	0	1
1665	PCAT1_HUMAN	Lysophosphatidylcholine acyltransferase 1	S76	100%	1.00	1	0	0	0	0	0	0	1	0	0	0	0	0	1
1666	PCAT1_HUMAN	Lysophosphatidylcholine acyltransferase 1	S79	100%	1.00	1	0	0	0	0	0	0	1	0	0	0	0	0	1
1667	PCAT1_HUMAN	Lysophosphatidylcholine acyltransferase 1	T50	92%	0.92	1	0	0	0	0	0	0	1	0	0	0	0	0	1
1668	LOXL4_HUMAN	Lysyl oxidase homolog 4	T196	26%	0.26	1	0	0	0	0	0	0	0	0	0	0	1	0	1
1669	MRC1_HUMAN	Macrophage mannose receptor 1	S1260	55%	0.55	1	0	0	0	1	0	0	0	0	0	0	0	0	1
1670	MRC1_HUMAN	Macrophage mannose receptor 1	S1268	94%	0.94	1	0	0	0	1	0	0	0	0	0	0	0	0	1
1671	MRC1_HUMAN	Macrophage mannose receptor 1	S1269	85%	0.85	1	0	0	0	1	0	0	0	0	0	0	0	0	1
1672	MRC1_HUMAN	Macrophage mannose receptor 1	S1279	86%	0.86	1	0	0	0	1	0	0	0	0	0	0	0	0	1
1673	MRC1_HUMAN	Macrophage mannose receptor 1	S1280	84%	0.84	1	0	0	0	1	0	0	0	0	0	0	0	0	1
1674	MRC1_HUMAN	Macrophage mannose receptor 1	S1283	94%	0.94	1	0	0	0	1	0	0	0	0	0	0	0	0	1
1675	MRC1_HUMAN	Macrophage mannose receptor 1	Y1284	93%	0.93	1	0	0	0	1	0	0	0	0	0	0	0	0	1
1676	MPP7_HUMAN	MAGUK p55 subfamily member 7	T271	96%	0.96	1	0	0	0	0	0	0	0	0	0	0	1	0	1
1677	MFSD6_HUMAN	Major facilitator superfamily domain-containing protein 6	S643	90%	0.90	4	0	0	0	0	0	0	0	0	0	0	1	0	1
1678	MFSD6_HUMAN	Major facilitator superfamily domain-containing protein 6	S644	25%	0.25	5	0	0	0	0	0	0	0	0	0	0	1	0	1
1679	MFSD8_HUMAN	Major facilitator superfamily domain-containing protein 8	S473	14%	0.14	1	0	1	0	0	0	0	0	0	0	0	0	0	1
1680	MFSD8_HUMAN	Major facilitator superfamily domain-containing protein 8	T497	14%	0.14	1	0	1	0	0	0	0	0	0	0	0	0	0	1

1681	MVP_HUMAN	Major vault protein	S864	50%	0.50	6	0	0	0	0	0	0	0	0	0	2	1	2	5
1682	MVP_HUMAN	Major vault protein	S867	94%	0.94	4	0	0	0	0	0	0	0	0	0	1	0	0	1
1683	MVP_HUMAN	Major vault protein	S873	100%	1.00	84	0	0	0	0	0	0	0	2	0	9	10	9	30
1684	MKNK2_HUMAN	MAP kinase-interacting serine/threonine-protein kinase 2	S452	93%	0.93	2	0	0	0	0	0	1	0	0	0	0	0	0	1
1685	MA7D1_HUMAN	MAP7 domain-containing protein 1	S112	34%	0.34	3	0	0	0	0	0	0	0	1	0	1	0	0	2
1686	MA7D1_HUMAN	MAP7 domain-containing protein 1	S113	81%	0.81	12	0	1	0	2	1	0	0	0	1	1	0	1	7
1687	MA7D1_HUMAN	MAP7 domain-containing protein 1	S116	31%	0.31	6	1	1	0	1	0	0	1	1	0	1	0	0	6
1688	MA7D1_HUMAN	MAP7 domain-containing protein 1	S539	2%	0.02	1	0	0	0	0	0	0	0	0	0	1	0	0	1
1689	MA7D1_HUMAN	MAP7 domain-containing protein 1	S544	100%	1.00	57	2	0	2	5	0	1	0	0	0	0	1	5	16
1690	MA7D1_HUMAN	MAP7 domain-containing protein 1	S811	43%	0.43	1	0	0	0	0	0	0	0	0	0	1	0	0	1
1691	MA7D1_HUMAN	MAP7 domain-containing protein 1	T118	82%	0.82	8	0	1	1	0	0	1	1	0	1	0	1	0	6
1692	MA7D1_HUMAN	MAP7 domain-containing protein 1	T813	100%	1.00	63	2	0	0	3	2	1	1	1	0	3	1	3	17
1693	MISSL_HUMAN	MAPK-interacting and spindle-stabilizing protein-like	S15	100%	1.00	502	6	7	4	11	8	7	6	6	6	9	13	16	99
1694	MISSL_HUMAN	MAPK-interacting and spindle-stabilizing protein-like	S2	10%	0.10	85	2	5	6	2	3	3	4	4	2	1	0	0	32
1695	MISSL_HUMAN	MAPK-interacting and spindle-stabilizing protein-like	S6	100%	1.00	25	0	0	0	1	1	1	2	0	4	0	0	0	9
1696	MRGX1_HUMAN	Mas-related G-protein coupled receptor member X1	T13	18%	0.18	1	0	0	0	0	0	0	0	0	1	0	0	0	1
1697	MRGX1_HUMAN	Mas-related G-protein coupled receptor member X1	T18	18%	0.18	1	0	0	0	0	0	0	0	0	1	0	0	0	1
1698	MXRA8_HUMAN	Matrix-remodeling-associated protein 8	S17	100%	1.00	1	0	0	0	0	0	0	0	0	0	1	0	0	1

1699	MXRA8_HUMAN	Matrix-remodeling-associated protein 8	S18	100%	1.00	1	0	0	0	0	0	0	0	0	0	1	0	0	1
1700	MXRA8_HUMAN	Matrix-remodeling-associated protein 8	S35	45%	0.45	1	0	0	0	0	0	0	0	0	0	1	0	0	1
1701	MXRA8_HUMAN	Matrix-remodeling-associated protein 8	S38	95%	0.95	1	0	0	0	0	0	0	0	0	0	1	0	0	1
1702	MXRA8_HUMAN	Matrix-remodeling-associated protein 8	S40	95%	0.95	1	0	0	0	0	0	0	0	0	0	1	0	0	1
1703	MXRA8_HUMAN	Matrix-remodeling-associated protein 8	S43	95%	0.95	1	0	0	0	0	0	0	0	0	0	1	0	0	1
1704	MGAP_HUMAN	MAX gene-associated protein	S2546	20%	0.20	1	0	0	0	0	0	0	0	0	1	0	0	0	1
1705	MGAP_HUMAN	MAX gene-associated protein	T2550	70%	0.70	1	0	0	0	0	0	0	0	0	1	0	0	0	1
1706	EVI1_HUMAN	MDS1 and EVI1 complex locus protein EVI1	S823	56%	0.56	1	0	0	1	0	0	0	0	0	0	0	0	0	1
1707	EVI1_HUMAN	MDS1 and EVI1 complex locus protein EVI1	S824	56%	0.56	1	0	0	1	0	0	0	0	0	0	0	0	0	1
1708	EVI1_HUMAN	MDS1 and EVI1 complex locus protein EVI1	S827	70%	0.70	1	0	0	1	0	0	0	0	0	0	0	0	0	1
1709	EVI1_HUMAN	MDS1 and EVI1 complex locus protein EVI1	S831	56%	0.56	1	0	0	1	0	0	0	0	0	0	0	0	0	1
1710	EVI1_HUMAN	MDS1 and EVI1 complex locus protein EVI1	T822	56%	0.56	1	0	0	1	0	0	0	0	0	0	0	0	0	1
1711	MDC1_HUMAN	Mediator of DNA damage checkpoint protein 1	S168	100%	1.00	203	8	8	5	4	6	6	4	4	4	5	5	3	62
1712	MDC1_HUMAN	Mediator of DNA damage checkpoint protein 1	S329	98%	0.98	73	3	2	3	2	3	3	2	3	2	2	3	2	30
1713	MDC1_HUMAN	Mediator of DNA damage checkpoint protein 1	T331	86%	0.86	24	0	1	0	1	0	0	0	0	0	0	0	0	2
1714	MED1_HUMAN	Mediator of RNA polymerase II transcription subunit 1	S772	26%	0.26	19	0	0	0	1	0	0	0	0	0	0	0	0	1
1715	MED1_HUMAN	Mediator of RNA polymerase II transcription subunit 1	T805	100%	1.00	8	0	0	0	0	0	1	0	0	0	0	0	0	1

1716	MED13_HUMAN	Mediator of RNA polymerase II transcription subunit 13	S350	41%	0.41	1	0	0	0	0	0	0	0	0	0	1	0	0	1
1717	MED13_HUMAN	Mediator of RNA polymerase II transcription subunit 13	S352	98%	0.98	1	0	0	0	0	0	0	0	0	0	1	0	0	1
1718	MED13_HUMAN	Mediator of RNA polymerase II transcription subunit 13	S357	96%	0.96	1	0	0	0	0	0	0	0	0	0	1	0	0	1
1719	MED13_HUMAN	Mediator of RNA polymerase II transcription subunit 13	S359	41%	0.41	1	0	0	0	0	0	0	0	0	0	1	0	0	1
1720	MED14_HUMAN	Mediator of RNA polymerase II transcription subunit 14	S26	40%	0.40	1	0	0	0	0	1	0	0	0	0	0	0	0	1
1721	MED15_HUMAN	Mediator of RNA polymerase II transcription subunit 15	T603	100%	1.00	1	0	0	0	0	0	0	1	0	0	0	0	0	1
1722	MED27_HUMAN	Mediator of RNA polymerase II transcription subunit 27	S177	28%	0.28	1	0	0	0	0	0	0	0	1	0	0	0	0	1
1723	MEI1_HUMAN	Meiosis inhibitor protein 1	S760	91%	0.91	1	0	0	0	0	0	0	0	0	0	0	1	0	1
1724	MAGBH_HUMAN	Melanoma-associated antigen B17	S187	98%	0.98	1	0	0	0	0	0	0	0	0	0	0	1	0	1
1725	MAGBH_HUMAN	Melanoma-associated antigen B17	S190	98%	0.98	1	0	0	0	0	0	0	0	0	0	0	1	0	1
1726	MAGBH_HUMAN	Melanoma-associated antigen B17	S191	69%	0.69	1	0	0	0	0	0	0	0	0	0	0	1	0	1
1727	MAGBH_HUMAN	Melanoma-associated antigen B17	S194	90%	0.90	1	0	0	0	0	0	0	0	0	0	0	1	0	1
1728	MAGBH_HUMAN	Melanoma-associated antigen B17	S200	86%	0.86	1	0	0	0	0	0	0	0	0	0	0	1	0	1
1729	MAGBH_HUMAN	Melanoma-associated antigen B17	S201	85%	0.85	1	0	0	0	0	0	0	0	0	0	0	1	0	1
1730	MAGBH_HUMAN	Melanoma-associated antigen B17	T199	87%	0.87	1	0	0	0	0	0	0	0	0	0	0	1	0	1
1731	MAGBH_HUMAN	Melanoma-associated antigen B17	Y188	98%	0.98	1	0	0	0	0	0	0	0	0	0	0	1	0	1
1732	PITM3_HUMAN	Membrane-associated phosphatidylinositol transfer protein 3	S321	100%	1.00	3	0	0	0	0	0	0	1	1	0	0	0	0	2

1733	PGRC1_HUMAN	Membrane-associated progesterone receptor component 1	S181	99%	0.99	74	1	2	1	3	2	2	2	3	1	3	1	2	23
1734	PGRC1_HUMAN	Membrane-associated progesterone receptor component 1	S57	100%	1.00	17	0	8	0	0	0	0	0	1	0	3	0	0	12
1735	CNNM1_HUMAN	Metal transporter CNNM1	S801	99%	0.99	1	0	1	0	0	0	0	0	0	0	0	0	0	1
1736	CNNM1_HUMAN	Metal transporter CNNM1	S812	92%	0.92	1	0	1	0	0	0	0	0	0	0	0	0	0	1
1737	CNNM1_HUMAN	Metal transporter CNNM1	T808	28%	0.28	1	0	1	0	0	0	0	0	0	0	0	0	0	1
1738	CNNM1_HUMAN	Metal transporter CNNM1	T813	83%	0.83	1	0	1	0	0	0	0	0	0	0	0	0	0	1
1739	CNNM1_HUMAN	Metal transporter CNNM1	T817	89%	0.89	1	0	1	0	0	0	0	0	0	0	0	0	0	1
1740	CNNM1_HUMAN	Metal transporter CNNM1	Y786	28%	0.28	1	0	1	0	0	0	0	0	0	0	0	0	0	1
1741	MTA1_HUMAN	Metastasis-associated protein MTA1	S522	100%	1.00	1	1	0	0	0	0	0	0	0	0	0	0	0	1
1742	MTA1_HUMAN	Metastasis-associated protein MTA1	T578	100%	1.00	6	0	0	0	0	0	0	0	0	0	0	0	1	1
1743	AMPM2_HUMAN	Methionine aminopeptidase 2	S29	90%	0.90	11	0	0	1	0	0	0	0	0	0	0	0	0	1
1744	AMPM2_HUMAN	Methionine aminopeptidase 2	T30	60%	0.60	4	1	1	0	0	0	0	0	0	0	0	0	0	2
1745	MSRB2_HUMAN	Methionine-R-sulfoxide reductase B2, mitochondrial	S76	99%	0.99	1	0	0	0	0	0	0	0	0	0	0	0	1	1
1746	MBD6_HUMAN	Methyl-CpG-binding domain protein 6	S163	96%	0.96	1	0	0	0	0	0	0	0	0	0	1	0	0	1
1747	MBD6_HUMAN	Methyl-CpG-binding domain protein 6	S170	100%	1.00	1	0	0	0	0	0	0	0	0	0	1	0	0	1
1748	MBD6_HUMAN	Methyl-CpG-binding domain protein 6	S172	100%	1.00	1	0	0	0	0	0	0	0	0	0	1	0	0	1
1749	MBD6_HUMAN	Methyl-CpG-binding domain protein 6	T155	19%	0.19	1	0	0	0	0	0	0	0	0	0	1	0	0	1
1750	MUTA_HUMAN	Methylmalonyl-CoA mutase, mitochondrial	S185	38%	0.38	1	0	0	0	0	0	0	0	0	1	0	0	0	1

1751	MUTA_HUMAN	Methylmalonyl-CoA mutase, mitochondrial	T187	85%	0.85	1	0	0	0	0	0	0	0	0	1	0	0	0	1
1752	MEP50_HUMAN	Methylosome protein 50	T5	100%	1.00	13	0	0	0	0	1	0	0	0	0	1	1	1	4
1753	ICLN_HUMAN	Methylosome subunit pICln	S102	100%	1.00	719	28	20	16	20	17	18	18	17	18	19	14	12	217
1754	ICLN_HUMAN	Methylosome subunit pICln	S90	0%	0.00	3	0	0	1	0	0	1	0	0	0	0	0	0	2
1755	CF192_HUMAN	MFS-type transporter C6orf192	S151	86%	0.86	11	0	0	0	0	1	0	0	0	0	0	0	0	1
1756	MILK1_HUMAN	MICAL-like protein 1	S295	1%	0.01	2	0	0	0	0	0	0	0	0	0	1	0	0	1
1757	MILK1_HUMAN	MICAL-like protein 1	S578	100%	1.00	177	4	6	8	3	4	6	4	4	4	9	9	9	70
1758	MFAP1_HUMAN	Microfibrillar-associated protein 1	S116	100%	1.00	31	0	4	0	0	4	0	0	6	0	0	0	0	14
1759	MFAP1_HUMAN	Microfibrillar-associated protein 1	S118	100%	1.00	33	2	4	0	0	4	0	0	6	0	0	0	0	16
1760	MGST1_HUMAN	Microsomal glutathione S-transferase 1	T5	22%	0.22	1	0	0	0	0	1	0	0	0	0	0	0	0	1
1761	MGST1_HUMAN	Microsomal glutathione S-transferase 1	Y37	22%	0.22	1	0	0	0	0	1	0	0	0	0	0	0	0	1
1762	MCRS1_HUMAN	Microspherule protein 1	S282	100%	1.00	17	0	0	0	0	0	0	0	0	0	0	1	2	3
1763	MACF1_HUMAN	Microtubule-actin cross-linking factor 1, isoforms 1/2/3/5	S1376	100%	1.00	63	0	0	0	0	2	0	0	4	0	10	7	8	31
1764	MACF1_HUMAN	Microtubule-actin cross-linking factor 1, isoforms 1/2/3/5	S1378	10%	0.10	2	0	0	0	0	0	0	0	0	0	1	0	0	1
1765	MACF1_HUMAN	Microtubule-actin cross-linking factor 1, isoforms 1/2/3/5	S4521	100%	1.00	9	0	0	0	0	0	0	0	0	0	4	2	3	9
1766	MACF1_HUMAN	Microtubule-actin cross-linking factor 1, isoforms 1/2/3/5	S5808	100%	1.00	1	0	0	0	0	0	0	0	0	0	0	1	0	1
1767	MACF1_HUMAN	Microtubule-actin cross-linking factor 1, isoforms 1/2/3/5	T6148	2%	0.02	2	0	0	1	0	0	0	0	0	0	0	0	0	1

1768	MAP1S_HUMAN	Microtubule-associated protein 1S	S759	100%	1.00	56	2	1	0	1	3	0	4	4	5	2	4	4	30
1769	MAP1S_HUMAN	Microtubule-associated protein 1S	S762	88%	0.88	33	1	1	2	0	1	1	1	2	0	2	2	0	13
1770	MAP1S_HUMAN	Microtubule-associated protein 1S	S809	53%	0.53	15	1	0	1	0	0	1	1	1	1	1	0	1	8
1771	MAP1S_HUMAN	Microtubule-associated protein 1S	T813	32%	0.32	17	2	0	0	1	1	0	1	1	0	0	1	1	8
1772	MAP4_HUMAN	Microtubule-associated protein 4	S1073	100%	1.00	650	14	13	12	14	18	13	12	12	12	12	14	13	159
1773	MAP4_HUMAN	Microtubule-associated protein 4	S280	100%	1.00	####	18	24	26	21	21	21	18	19	22	21	24	19	254
1774	MAP4_HUMAN	Microtubule-associated protein 4	S358	100%	1.00	5	0	0	1	0	0	0	0	0	0	0	0	0	1
1775	MAP4_HUMAN	Microtubule-associated protein 4	S507	100%	1.00	143	2	0	0	4	0	2	0	2	4	7	8	8	37
1776	MAP4_HUMAN	Microtubule-associated protein 4	S624	71%	0.71	49	1	2	1	4	1	2	0	1	2	4	3	1	22
1777	MAP4_HUMAN	Microtubule-associated protein 4	S636	100%	1.00	206	7	5	5	9	5	7	5	5	6	7	2	3	66
1778	MAP4_HUMAN	Microtubule-associated protein 4	T282	98%	0.98	202	4	8	2	4	4	2	4	5	3	4	2	3	45
1779	MAP4_HUMAN	Microtubule-associated protein 4	T521	100%	1.00	####	18	16	21	18	15	11	22	19	19	17	17	14	207
1780	MAP4_HUMAN	Microtubule-associated protein 4	T526	15%	0.15	16	0	1	0	0	0	1	1	1	0	0	0	0	4
1781	MAP4_HUMAN	Microtubule-associated protein 4	T620	66%	0.66	10	1	0	0	0	1	0	0	2	0	0	1	0	5
1782	MAP4_HUMAN	Microtubule-associated protein 4	T627	34%	0.34	7	0	0	0	0	1	1	0	0	0	0	0	0	2
1783	MAP4_HUMAN	Microtubule-associated protein 4	T629	6%	0.06	1	0	0	0	0	1	0	0	0	0	0	0	0	1
1784	MAST2_HUMAN	Microtubule-associated serine/threonine-protein kinase 2	S911	29%	0.29	1	0	0	1	0	0	0	0	0	0	0	0	0	1
1785	MAST4_HUMAN	Microtubule-associated serine/threonine-protein kinase 4	S1373	100%	1.00	1	1	0	0	0	0	0	0	0	0	0	0	0	1

1786	MAST4_HUMAN	Microtubule-associated serine/threonine-protein kinase 4	S1782	100%	1.00	108	0	0	0	6	6	4	2	0	1	6	6	4	35
1787	M1IP1_HUMAN	Mid1-interacting protein 1	T67	100%	1.00	39	0	0	0	2	2	1	1	0	1	0	0	0	7
1788	M18BP_HUMAN	Mis18-binding protein 1	S1008	93%	0.93	1	0	1	0	0	0	0	0	0	0	0	0	0	1
1789	MINK1_HUMAN	Misshapen-like kinase 1	S732	69%	0.69	2	0	0	0	0	0	0	0	0	0	1	0	0	1
1790	MINK1_HUMAN	Misshapen-like kinase 1	S763	100%	1.00	38	3	3	2	1	2	2	1	3	2	2	3	1	25
1791	MAVS_HUMAN	Mitochondrial antiviral-signaling protein	S222	100%	1.00	16	0	0	0	1	0	1	0	0	0	0	0	0	2
1792	TOM22_HUMAN	Mitochondrial import receptor subunit TOM22 homolog	S15	100%	1.00	159	1	3	0	3	2	0	2	4	1	9	6	3	34
1793	TOM70_HUMAN	Mitochondrial import receptor subunit TOM70	S91	100%	1.00	341	8	11	7	7	6	7	7	11	4	8	8	4	88
1794	TOM70_HUMAN	Mitochondrial import receptor subunit TOM70	S96	58%	0.58	19	1	0	0	2	2	3	0	0	0	0	0	0	8
1795	NHDC2_HUMAN	Mitochondrial sodium/hydrogen exchanger NHA2	T474	48%	0.48	1	0	0	0	0	0	0	0	0	0	1	0	0	1
1796	MK01_HUMAN	Mitogen-activated protein kinase 1	T181	82%	0.82	35	0	0	0	0	0	0	0	0	0	2	0	0	2
1797	MK01_HUMAN	Mitogen-activated protein kinase 1	T185	93%	0.93	59	0	0	0	0	1	0	0	0	0	4	3	1	9
1798	MK01_HUMAN	Mitogen-activated protein kinase 1	T190	100%	1.00	30	0	0	0	0	0	0	0	0	0	0	2	1	3
1799	MK01_HUMAN	Mitogen-activated protein kinase 1	Y187	100%	1.00	294	2	4	3	3	5	5	6	5	6	6	6	8	59
1800	MK03_HUMAN	Mitogen-activated protein kinase 3	Y204	100%	1.00	54	0	0	0	0	0	0	1	1	0	3	3	2	10
1801	MK08_HUMAN	Mitogen-activated protein kinase 8	T367	100%	1.00	1	0	0	0	0	0	0	0	0	0	0	0	1	1
1802	M3K2_HUMAN	Mitogen-activated protein kinase kinase kinase 2	S153	88%	0.88	1	0	0	1	0	0	0	0	0	0	0	0	0	1
1803	M3K2_HUMAN	Mitogen-activated protein kinase kinase kinase 2	S163	80%	0.80	7	1	1	0	0	0	0	0	0	0	1	1	0	4

1804	M3K2_HUMAN	Mitogen-activated protein kinase kinase kinase 2	S164	98%	0.98	21	1	2	0	0	1	1	0	0	0	2	3	4	14
1805	M3K6_HUMAN	Mitogen-activated protein kinase kinase kinase 6	S984	40%	0.40	1	1	0	0	0	0	0	0	0	0	0	0	0	1
1806	M3K7_HUMAN	Mitogen-activated protein kinase kinase kinase 7	S389	100%	1.00	81	1	2	3	2	3	3	5	1	5	2	4	1	32
1807	M3K7_HUMAN	Mitogen-activated protein kinase kinase kinase 7	S439	100%	1.00	116	1	2	3	4	3	3	2	2	3	1	2	4	30
1808	M4K4_HUMAN	Mitogen-activated protein kinase kinase kinase kinase 4	S868	89%	0.89	4	0	0	0	1	0	0	0	0	0	0	0	0	1
1809	M4K4_HUMAN	Mitogen-activated protein kinase kinase kinase kinase 4	S900	100%	1.00	47	3	1	1	1	0	0	2	0	2	0	1	2	13
1810	M4K4_HUMAN	Mitogen-activated protein kinase kinase kinase kinase 4	T915	58%	0.58	1	0	0	0	0	0	0	0	0	0	1	0	0	1
1811	BUB1_HUMAN	Mitotic checkpoint serine/threonine-protein kinase BUB1	S381	58%	0.58	2	0	0	0	0	0	0	0	0	0	1	0	0	1
1812	MK67I_HUMAN	MKI67 FHA domain-interacting nucleolar phosphoprotein	T223	42%	0.42	5	0	0	0	0	1	0	0	0	0	0	0	0	1
1813	MOES_HUMAN	Moesin	T98	100%	1.00	1	0	0	0	0	0	0	0	0	0	0	1	0	1
1814	MOCOS_HUMAN	Molybdenum cofactor sulfurase	S528	25%	0.25	1	0	0	0	0	0	0	0	0	1	0	0	0	1
1815	MOC2B_HUMAN	Molybdopterin synthase catalytic subunit	S20	100%	1.00	38	0	0	0	0	0	0	0	0	0	0	0	1	1
1816	ABHD6_HUMAN	Monoacylglycerol lipase ABHD6	S32	98%	0.98	5	0	0	0	0	1	0	0	0	0	0	0	0	1
1817	MORC2_HUMAN	MORC family CW-type zinc finger protein 2	S615	100%	1.00	17	0	1	0	3	1	0	1	2	0	2	2	5	17
1818	MORC2_HUMAN	MORC family CW-type zinc finger protein 2	S743	100%	1.00	4	2	0	0	0	0	0	0	2	0	0	0	0	4
1819	SMAD2_HUMAN	Mothers against decapentaplegic homolog 2	T8	100%	1.00	1	0	0	0	0	0	0	0	0	1	0	0	0	1
1820	MPIP2_HUMAN	M-phase inducer phosphatase 2	S249	100%	1.00	1	0	0	0	0	0	0	0	0	0	0	0	1	1

1821	MPP8_HUMAN	M-phase phosphoprotein 8	S51	100%	1.00	5	2	0	0	0	2	0	0	0	0	0	0	0	4
1822	DCP1A_HUMAN	mRNA-decapping enzyme 1A	S315	100%	1.00	23	1	3	1	0	1	0	1	2	0	0	0	0	9
1823	DCP1A_HUMAN	mRNA-decapping enzyme 1A	S319	83%	0.83	5	1	0	0	0	0	0	0	0	0	0	0	0	1
1824	DCP1A_HUMAN	mRNA-decapping enzyme 1A	T311	32%	0.32	1	1	0	0	0	0	0	0	0	0	0	0	0	1
1825	MINT_HUMAN	Msx2-interacting protein	S1278	100%	1.00	6	0	0	0	0	0	0	5	0	0	0	0	0	5
1826	MINT_HUMAN	Msx2-interacting protein	S934	93%	0.93	1	0	0	0	0	0	0	0	0	0	0	0	1	1
1827	MTSSL_HUMAN	MTSS1-like protein	S263	69%	0.69	1	0	0	1	0	0	0	0	0	0	0	0	0	1
1828	MTSSL_HUMAN	MTSS1-like protein	S264	69%	0.69	1	0	0	1	0	0	0	0	0	0	0	0	0	1
1829	MTSSL_HUMAN	MTSS1-like protein	S266	95%	0.95	1	0	0	1	0	0	0	0	0	0	0	0	0	1
1830	MTSSL_HUMAN	MTSS1-like protein	S267	95%	0.95	1	0	0	1	0	0	0	0	0	0	0	0	0	1
1831	MTSSL_HUMAN	MTSS1-like protein	S268	99%	0.99	1	0	0	1	0	0	0	0	0	0	0	0	0	1
1832	MTSSL_HUMAN	MTSS1-like protein	S269	100%	1.00	1	0	0	1	0	0	0	0	0	0	0	0	0	1
1833	MTSSL_HUMAN	MTSS1-like protein	S270	100%	1.00	1	0	0	1	0	0	0	0	0	0	0	0	0	1
1834	MTSSL_HUMAN	MTSS1-like protein	Y254	15%	0.15	1	0	0	1	0	0	0	0	0	0	0	0	0	1
1835	MUC16_HUMAN	Mucin-16	S10165	28%	0.28	1	1	0	0	0	0	0	0	0	0	0	0	0	1
1836	MUC16_HUMAN	Mucin-16	S10169	21%	0.21	1	1	0	0	0	0	0	0	0	0	0	0	0	1
1837	MUC16_HUMAN	Mucin-16	T10168	49%	0.49	1	1	0	0	0	0	0	0	0	0	0	0	0	1
1838	MUC4_HUMAN	Mucin-4	S404	34%	0.34	1	0	0	0	0	0	0	0	0	0	0	0	1	1
1839	MUC4_HUMAN	Mucin-4	S407	62%	0.62	1	0	0	0	0	0	0	0	0	0	0	0	1	1
1840	MUC4_HUMAN	Mucin-4	S424	53%	0.53	1	0	0	0	0	0	0	0	0	0	0	0	1	1

1841	MUC4_HUMAN	Mucin-4	S428	49%	0.49	1	0	0	0	0	0	0	0	0	0	0	0	1	1
1842	MUC4_HUMAN	Mucin-4	T422	62%	0.62	1	0	0	0	0	0	0	0	0	0	0	0	1	1
1843	MUC4_HUMAN	Mucin-4	T427	52%	0.52	1	0	0	0	0	0	0	0	0	0	0	0	1	1
1844	MUC5A_HUMAN	Mucin-5AC (Fragments)	S3071	44%	0.44	1	0	0	0	0	0	0	0	0	0	0	1	0	1
1845	MUC5A_HUMAN	Mucin-5AC (Fragments)	S3073	44%	0.44	1	0	0	0	0	0	0	0	0	0	0	1	0	1
1846	MUC5A_HUMAN	Mucin-5AC (Fragments)	S3074	43%	0.43	1	0	0	0	0	0	0	0	0	0	0	1	0	1
1847	MUC5A_HUMAN	Mucin-5AC (Fragments)	T3069	38%	0.38	1	0	0	0	0	0	0	0	0	0	0	1	0	1
1848	MUC5A_HUMAN	Mucin-5AC (Fragments)	T3072	44%	0.44	1	0	0	0	0	0	0	0	0	0	0	1	0	1
1849	MPDZ_HUMAN	Multiple PDZ domain protein	S1265	17%	0.17	1	0	0	0	0	0	0	0	0	0	0	1	0	1
1850	MPDZ_HUMAN	Multiple PDZ domain protein	S1266	17%	0.17	2	0	0	0	0	0	0	0	0	0	0	1	0	1
1851	MPDZ_HUMAN	Multiple PDZ domain protein	T1267	37%	0.37	1	0	0	0	0	0	0	0	1	0	0	0	0	1
1852	MPDZ_HUMAN	Multiple PDZ domain protein	Y1262	78%	0.78	3	0	0	0	0	0	0	0	1	0	0	0	0	1
1853	F125A_HUMAN	Multivesicular body subunit 12A	S170	100%	1.00	1	0	1	0	0	0	0	0	0	0	0	0	0	1
1854	MBB1A_HUMAN	Myb-binding protein 1A	S775	100%	1.00	770	33	14	15	15	19	13	21	18	18	19	9	9	203
1855	MBB1A_HUMAN	Myb-binding protein 1A	T762	62%	0.62	104	6	4	4	3	1	2	1	6	2	5	1	1	36
1856	BIN1_HUMAN	Myc box-dependent-interacting protein 1	S296	87%	0.87	10	0	2	2	0	1	1	2	1	0	0	0	0	9
1857	BIN1_HUMAN	Myc box-dependent-interacting protein 1	S298	50%	0.50	12	1	0	0	0	3	1	0	2	0	0	0	0	7
1858	BIN1_HUMAN	Myc box-dependent-interacting protein 1	S303	6%	0.06	1	0	0	0	0	0	1	0	0	0	0	0	0	1
1859	BIN1_HUMAN	Myc box-dependent-interacting protein 1	S331	12%	0.12	2	0	0	1	0	0	0	0	0	0	0	0	0	1

1860	BIN1_HUMAN	Myc box-dependent-interacting protein 1	S333	93%	0.93	3	0	2	0	0	1	0	0	0	0	0	0	0	3
1861	MAZ_HUMAN	Myc-associated zinc finger protein	S234	67%	0.67	2	0	0	1	0	0	0	0	0	0	0	0	0	1
1862	MAZ_HUMAN	Myc-associated zinc finger protein	S237	68%	0.68	2	0	0	1	0	0	0	0	0	0	0	0	0	1
1863	MAZ_HUMAN	Myc-associated zinc finger protein	S242	53%	0.53	2	0	0	1	0	0	0	0	0	0	0	0	0	1
1864	MYEF2_HUMAN	Myelin expression factor 2	S17	100%	1.00	8	0	0	0	0	0	0	0	0	0	1	1	2	4
1865	CD33_HUMAN	Myeloid cell surface antigen CD33	T253	100%	1.00	19	0	0	0	0	1	0	1	1	0	0	1	0	4
1866	PERM_HUMAN	Myeloperoxidase	S406	63%	0.63	10	0	1	0	0	0	0	0	1	0	0	0	0	2
1867	PERM_HUMAN	Myeloperoxidase	T404	60%	0.60	10	0	1	0	0	0	0	0	1	0	0	0	0	2
1868	PERM_HUMAN	Myeloperoxidase	T417	83%	0.83	10	0	1	0	0	0	0	0	1	0	0	0	0	2
1869	MEF2A_HUMAN	Myocyte-specific enhancer factor 2A	S235	100%	1.00	30	1	1	1	0	2	2	3	1	2	0	0	2	15
1870	MEF2D_HUMAN	Myocyte-specific enhancer factor 2D	S121	100%	1.00	6	0	0	0	0	0	0	0	0	0	2	1	3	6
1871	MEF2D_HUMAN	Myocyte-specific enhancer factor 2D	S231	100%	1.00	12	1	0	2	0	0	0	1	3	0	3	1	1	12
1872	MEF2D_HUMAN	Myocyte-specific enhancer factor 2D	S251	100%	1.00	86	7	5	2	5	5	3	5	4	1	6	6	8	57
1873	MEF2D_HUMAN	Myocyte-specific enhancer factor 2D	T256	0%	0.00	1	0	0	0	0	0	0	1	0	0	0	0	0	1
1874	MYOM1_HUMAN	Myomesin-1	S886	28%	0.28	1	0	0	0	0	0	0	0	0	1	0	0	0	1
1875	MYOM1_HUMAN	Myomesin-1	S887	28%	0.28	1	0	0	0	0	0	0	0	0	1	0	0	0	1
1876	MPRIP_HUMAN	Myosin phosphatase Rho-interacting protein	S301	100%	1.00	1	0	0	0	0	1	0	0	0	0	0	0	0	1
1877	MPRIP_HUMAN	Myosin phosphatase Rho-interacting protein	S540	96%	0.96	1	0	1	0	0	0	0	0	0	0	0	0	0	1
1878	MPRIP_HUMAN	Myosin phosphatase Rho-interacting protein	S619	100%	1.00	3	0	0	0	0	1	0	0	0	0	0	1	0	2

1879	ML12A_HUMAN	Myosin regulatory light chain 12A	S19	99%	0.99	335	16	25	10	8	14	12	7	8	8	25	19	10	162
1880	ML12A_HUMAN	Myosin regulatory light chain 12A	T128	53%	0.53	1	0	0	0	0	0	0	1	0	0	0	0	0	1
1881	ML12A_HUMAN	Myosin regulatory light chain 12A	T18	33%	0.33	6	0	0	0	0	0	0	0	0	0	1	0	0	1
1882	ML12A_HUMAN	Myosin regulatory light chain 12A	Y121	30%	0.30	1	0	0	0	0	0	0	1	0	0	0	0	0	1
1883	ML12B_HUMAN	Myosin regulatory light chain 12B	S20	99%	0.99	335	16	25	10	8	14	12	7	8	8	25	19	10	162
1884	ML12B_HUMAN	Myosin regulatory light chain 12B	T129	53%	0.53	1	0	0	0	0	0	0	1	0	0	0	0	0	1
1885	ML12B_HUMAN	Myosin regulatory light chain 12B	T19	33%	0.33	6	0	0	0	0	0	0	0	0	0	1	0	0	1
1886	ML12B_HUMAN	Myosin regulatory light chain 12B	Y122	30%	0.30	1	0	0	0	0	0	0	1	0	0	0	0	0	1
1887	MYH10_HUMAN	Myosin-10	S1956	100%	1.00	13	0	0	0	0	1	0	0	1	0	0	0	0	2
1888	MYH13_HUMAN	Myosin-13	S1226	67%	0.67	1	0	1	0	0	0	0	0	0	0	0	0	0	1
1889	MYH13_HUMAN	Myosin-13	S1243	67%	0.67	1	0	1	0	0	0	0	0	0	0	0	0	0	1
1890	MYH9_HUMAN	Myosin-9	S1943	100%	1.00	76	3	2	4	4	4	10	3	5	10	5	4	9	63
1891	MYO1D_HUMAN	Myosin-Id	T686	100%	1.00	2	0	0	0	0	0	1	0	0	0	0	0	0	1
1892	MYO1D_HUMAN	Myosin-Id	T689	100%	1.00	2	0	0	0	0	0	1	0	0	0	0	0	0	1
1893	MYO9B_HUMAN	Myosin-IXb	S1992	98%	0.98	2	1	0	1	0	0	0	0	0	0	0	0	0	2
1894	MYO6_HUMAN	Myosin-VI	S28	81%	0.81	1	0	0	0	0	0	0	0	1	0	0	0	0	1
1895	MYO6_HUMAN	Myosin-VI	T30	76%	0.76	1	0	0	0	0	0	0	0	1	0	0	0	0	1
1896	MY18A_HUMAN	Myosin-XVIIIa	S102	77%	0.77	21	0	0	1	0	0	0	0	1	0	3	3	1	9
1897	MY18A_HUMAN	Myosin-XVIIIa	S103	12%	0.13	7	0	1	0	0	0	0	1	1	0	1	0	0	4
1898	MY18A_HUMAN	Myosin-XVIIIa	S1067	94%	0.94	26	0	0	0	0	0	0	0	0	0	0	1	1	2

1899	MY18A_HUMAN	Myosin-XVIIIa	S1070	20%	0.20	2	0	0	0	0	0	0	0	0	0	0	1	0	1
1900	MY18A_HUMAN	Myosin-XVIIIa	S145	13%	0.13	4	1	1	0	0	0	0	0	0	0	1	0	0	3
1901	MY18A_HUMAN	Myosin-XVIIIa	S1942	100%	1.00	17	0	0	0	0	0	1	0	3	0	1	2	0	7
1902	MY18A_HUMAN	Myosin-XVIIIa	S2020	100%	1.00	11	0	0	0	0	0	0	0	0	0	3	0	1	4
1903	MY18A_HUMAN	Myosin-XVIIIa	S72	20%	0.20	1	0	0	0	1	0	0	0	0	0	0	0	0	1
1904	MY18A_HUMAN	Myosin-XVIIIa	T99	31%	0.31	2	0	0	0	0	0	0	1	0	0	1	0	0	2
1905	MY18B_HUMAN	Myosin-XVIIIb	T2379	0%	0.00	1	0	0	0	0	0	0	0	0	0	1	0	0	1
1906	MTMR3_HUMAN	Myotubularin-related protein 3	S647	100%	1.00	15	0	1	0	0	0	0	0	0	0	0	1	0	2
1907	MTMR3_HUMAN	Myotubularin-related protein 3	S651	17%	0.17	3	0	1	0	0	0	0	1	0	0	0	0	0	2
1908	MTMR3_HUMAN	Myotubularin-related protein 3	T367	87%	0.87	3	0	0	1	0	0	0	0	0	0	0	0	0	1
1909	MTMR3_HUMAN	Myotubularin-related protein 3	T382	43%	0.43	3	0	0	1	0	0	0	0	0	0	0	0	0	1
1910	MARCS_HUMAN	Myristoylated alanine-rich C-kinase substrate	S101	100%	1.00	112	0	0	0	2	0	1	0	0	1	5	4	6	19
1911	MARCS_HUMAN	Myristoylated alanine-rich C-kinase substrate	S118	99%	0.99	43	0	0	0	0	0	0	0	0	0	6	3	0	9
1912	MARCS_HUMAN	Myristoylated alanine-rich C-kinase substrate	S147	86%	0.86	3	0	0	0	0	0	0	0	0	0	1	2	0	3
1913	MARCS_HUMAN	Myristoylated alanine-rich C-kinase substrate	S26	5%	0.05	3	0	0	0	0	0	1	0	0	0	0	0	0	1
1914	MARCS_HUMAN	Myristoylated alanine-rich C-kinase substrate	S27	100%	1.00	21	0	0	0	1	0	2	1	1	1	3	4	6	19
1915	MARCS_HUMAN	Myristoylated alanine-rich C-kinase substrate	S29	64%	0.64	4	0	0	0	0	0	0	0	0	0	1	0	2	3
1916	MARCS_HUMAN	Myristoylated alanine-rich C-kinase substrate	S77	76%	0.76	19	0	0	0	0	0	0	0	0	0	2	1	1	4
1917	MARCS_HUMAN	Myristoylated alanine-rich C-kinase substrate	S81	97%	0.97	32	0	0	0	0	0	0	0	0	0	4	7	5	16

1918	MARCS_HUMAN	Myristoylated alanine-rich C-kinase substrate	S83	84%	0.84	14	0	0	0	0	0	0	0	0	0	1	0	1	2
1919	MARCS_HUMAN	Myristoylated alanine-rich C-kinase substrate	T120	44%	0.44	21	0	0	0	0	0	0	0	0	0	3	2	2	7
1920	MARCS_HUMAN	Myristoylated alanine-rich C-kinase substrate	T150	100%	1.00	3	0	0	0	0	0	0	0	0	0	0	1	2	3
1921	N6MT2_HUMAN	N(6)-adenine-specific DNA methyltransferase 2	S13	5%	0.05	1	0	0	0	0	0	0	0	0	0	0	1	0	1
1922	N6MT2_HUMAN	N(6)-adenine-specific DNA methyltransferase 2	S2	100%	1.00	356	14	11	14	6	8	6	8	10	9	6	7	3	102
1923	N6MT2_HUMAN	N(6)-adenine-specific DNA methyltransferase 2	T9	96%	0.96	5	0	0	0	0	1	0	0	0	0	0	0	0	1
1924	NHRF1_HUMAN	Na(+)/H(+) exchange regulatory cofactor NHE-RF1	S280	100%	1.00	249	0	3	2	5	6	3	5	9	4	8	8	9	62
1925	NAGK_HUMAN	N-acetyl-D-glucosamine kinase	S74	95%	0.95	3	0	0	0	0	0	0	1	0	0	0	0	0	1
1926	ASML_HUMAN	N-acetylserotonin O-methyltransferase-like protein	S239	93%	0.93	1	0	0	1	0	0	0	0	0	0	0	0	0	1
1927	NAT6_HUMAN	N-acetyltransferase 6	S8	96%	0.96	1	0	0	0	0	0	0	0	0	0	1	0	0	1
1928	SIRT1_HUMAN	NAD-dependent deacetylase sirtuin-1	S14	100%	1.00	5	3	0	0	1	0	0	0	0	0	0	0	0	4
1929	SIRT2_HUMAN	NAD-dependent deacetylase sirtuin-2	S368	60%	0.60	1	0	0	0	0	0	0	0	0	0	0	1	0	1
1930	NAA15_HUMAN	N-alpha-acetyltransferase 15, NatA auxiliary subunit	S588	1%	0.01	1	0	0	0	1	0	0	0	0	0	0	0	0	1
1931	NAA15_HUMAN	N-alpha-acetyltransferase 15, NatA auxiliary subunit	S856	100%	1.00	21	0	0	0	1	0	0	0	0	0	0	0	0	1
1932	NRDC_HUMAN	Nardilysin	S94	100%	1.00	11	0	0	0	0	0	0	1	0	2	0	0	0	3
1933	NACA_HUMAN	Nascent polypeptide-associated complex subunit alpha	S166	100%	1.00	####	43	16	21	28	18	23	30	16	17	11	13	17	253
1934	NACA_HUMAN	Nascent polypeptide-associated complex subunit alpha	T159	99%	0.99	7	0	0	1	1	0	0	0	0	0	0	1	0	3

1935	NACA_HUMAN	Nascent polypeptide-associated complex subunit alpha	T161	100%	1.00	53	0	0	1	1	0	0	1	0	0	0	0	0	3
1936	NACA_HUMAN	Nascent polypeptide-associated complex subunit alpha	T174	30%	0.30	5	0	0	0	0	0	0	1	0	0	0	0	1	2
1937	ANFB_HUMAN	Natriuretic peptides B	S124	64%	0.64	109	4	3	5	1	3	2	1	3	2	1	2	0	27
1938	NEBU_HUMAN	Nebulin	S2700	40%	0.40	1	0	0	0	0	0	0	0	0	0	0	1	0	1
1939	NEBU_HUMAN	Nebulin	T2717	79%	0.79	1	0	0	0	0	0	0	0	0	0	0	1	0	1
1940	NRAP_HUMAN	Nebulin-related-anchoring protein	T1251	86%	0.86	1	0	0	0	0	0	0	0	0	0	0	1	0	1
1941	NELFA_HUMAN	Negative elongation factor A	S327	25%	0.25	1	0	0	0	0	1	0	0	0	0	0	0	0	1
1942	NELFA_HUMAN	Negative elongation factor A	S330	39%	0.39	1	0	0	0	0	1	0	0	0	0	0	0	0	1
1943	NELFA_HUMAN	Negative elongation factor A	S335	26%	0.26	1	0	0	0	0	1	0	0	0	0	0	0	0	1
1944	NELFA_HUMAN	Negative elongation factor A	S363	85%	0.85	1	0	0	0	0	0	0	0	0	0	0	1	0	1
1945	NELFA_HUMAN	Negative elongation factor A	T328	25%	0.25	1	0	0	0	0	1	0	0	0	0	0	0	0	1
1946	NELFA_HUMAN	Negative elongation factor A	Y324	34%	0.34	1	0	0	0	0	1	0	0	0	0	0	0	0	1
1947	NELFA_HUMAN	Negative elongation factor A	Y337	26%	0.26	1	0	0	0	0	1	0	0	0	0	0	0	0	1
1948	NELFB_HUMAN	Negative elongation factor B	S557	100%	1.00	607	13	14	14	14	13	11	9	13	12	12	12	11	148
1949	NELFB_HUMAN	Negative elongation factor B	T564	70%	0.70	24	1	0	0	2	1	1	1	0	1	1	0	0	8
1950	NELFE_HUMAN	Negative elongation factor E	T263	79%	0.79	1	0	0	0	0	0	0	1	0	0	0	0	0	1
1951	NELFE_HUMAN	Negative elongation factor E	T272	72%	0.72	1	0	0	0	0	0	0	1	0	0	0	0	0	1
1952	NELFE_HUMAN	Negative elongation factor E	T274	58%	0.58	1	0	0	0	0	0	0	1	0	0	0	0	0	1
1953	NELFE_HUMAN	Negative elongation factor E	Y265	81%	0.81	1	0	0	0	0	0	0	1	0	0	0	0	0	1

1954	NELFE_HUMAN	Negative elongation factor E	Y267	84%	0.84	1	0	0	0	0	0	0	1	0	0	0	0	0	1
1955	NEO1_HUMAN	Neogenin	S1194	100%	1.00	1	0	0	0	0	0	0	0	0	0	0	1	0	1
1956	SYNE1_HUMAN	Nesprin-1	S8098	100%	1.00	1	0	0	0	0	0	0	0	0	0	0	1	0	1
1957	NEB2_HUMAN	Neurabin-2	S190	75%	0.75	15	0	0	0	0	0	1	1	0	0	0	0	0	2
1958	NEB2_HUMAN	Neurabin-2	T191	17%	0.17	50	0	1	2	0	1	1	1	3	3	1	2	2	17
1959	NBEA_HUMAN	Neurobeachin	S3	61%	0.61	1	0	0	0	0	0	0	0	0	0	0	0	1	1
1960	NBEL2_HUMAN	Neurobeachin-like protein 2	T1867	100%	1.00	8	0	0	0	0	0	0	0	0	0	1	1	0	2
1961	AHNK_HUMAN	Neuroblast differentiation-associated protein AHNAK	S135	100%	1.00	417	0	5	2	14	12	11	12	13	11	14	13	11	118
1962	AHNK_HUMAN	Neuroblast differentiation-associated protein AHNAK	S176	0%	0.00	2	0	0	0	0	0	0	0	0	0	1	0	1	2
1963	AHNK_HUMAN	Neuroblast differentiation-associated protein AHNAK	S177	100%	1.00	105	3	2	2	3	3	4	5	2	3	4	4	4	39
1964	AHNK_HUMAN	Neuroblast differentiation-associated protein AHNAK	S210	100%	1.00	645	0	5	8	15	15	13	9	8	11	25	27	29	165
1965	AHNK_HUMAN	Neuroblast differentiation-associated protein AHNAK	S212	98%	0.98	149	3	2	3	3	3	4	6	3	6	4	3	0	40
1966	AHNK_HUMAN	Neuroblast differentiation-associated protein AHNAK	S216	100%	1.00	####	17	21	21	27	26	27	27	27	29	36	29	32	319
1967	AHNK_HUMAN	Neuroblast differentiation-associated protein AHNAK	S220	46%	0.46	87	0	0	0	1	3	2	1	2	1	2	3	2	17
1968	AHNK_HUMAN	Neuroblast differentiation-associated protein AHNAK	S2397	100%	1.00	138	0	0	0	0	0	0	0	0	0	9	12	8	29
1969	AHNK_HUMAN	Neuroblast differentiation-associated protein AHNAK	S3412	100%	1.00	8	0	0	0	0	0	0	0	0	0	2	2	3	7
1970	AHNK_HUMAN	Neuroblast differentiation-associated protein AHNAK	S3426	100%	1.00	99	0	0	0	0	1	0	0	0	0	9	11	8	29
1971	AHNK_HUMAN	Neuroblast differentiation-associated protein AHNAK	S41	100%	1.00	5	0	2	0	0	0	0	0	0	0	3	0	0	5

1972	AHNK_HUMAN	Neuroblast differentiation-associated protein AHNAK	S4850	100%	1.00	18	0	0	1	0	1	0	0	0	0	0	1	0	3
1973	AHNK_HUMAN	Neuroblast differentiation-associated protein AHNAK	S4993	84%	0.84	33	0	0	0	0	0	0	0	0	0	0	1	2	3
1974	AHNK_HUMAN	Neuroblast differentiation-associated protein AHNAK	S508	2%	0.02	3	0	0	0	0	0	0	0	0	0	1	0	0	1
1975	AHNK_HUMAN	Neuroblast differentiation-associated protein AHNAK	S5099	100%	1.00	75	0	0	0	0	0	0	0	0	0	3	6	4	13
1976	AHNK_HUMAN	Neuroblast differentiation-associated protein AHNAK	S511	100%	1.00	261	0	1	0	1	2	0	0	0	0	15	21	11	51
1977	AHNK_HUMAN	Neuroblast differentiation-associated protein AHNAK	S5110	100%	1.00	147	1	0	1	0	0	0	0	0	0	17	12	11	42
1978	AHNK_HUMAN	Neuroblast differentiation-associated protein AHNAK	S5448	100%	1.00	413	3	4	1	8	6	6	6	6	6	8	6	6	66
1979	AHNK_HUMAN	Neuroblast differentiation-associated protein AHNAK	S5552	100%	1.00	61	0	0	0	0	0	0	0	0	0	6	6	4	16
1980	AHNK_HUMAN	Neuroblast differentiation-associated protein AHNAK	S570	100%	1.00	221	0	0	0	6	2	4	3	0	0	8	8	7	38
1981	AHNK_HUMAN	Neuroblast differentiation-associated protein AHNAK	S572	41%	0.41	20	0	0	0	1	0	1	1	0	0	0	0	0	3
1982	AHNK_HUMAN	Neuroblast differentiation-associated protein AHNAK	S5731	8%	0.08	1	0	0	0	0	0	0	0	0	0	0	1	0	1
1983	AHNK_HUMAN	Neuroblast differentiation-associated protein AHNAK	S5749	100%	1.00	148	1	4	1	0	0	4	3	3	1	0	3	0	20
1984	AHNK_HUMAN	Neuroblast differentiation-associated protein AHNAK	S5752	100%	1.00	520	11	13	15	9	9	17	13	8	12	6	5	6	124
1985	AHNK_HUMAN	Neuroblast differentiation-associated protein AHNAK	S5762	82%	0.82	29	1	0	0	0	1	0	3	1	0	2	3	2	13
1986	AHNK_HUMAN	Neuroblast differentiation-associated protein AHNAK	S5763	100%	1.00	384	4	4	3	7	7	7	7	9	6	14	12	7	87
1987	AHNK_HUMAN	Neuroblast differentiation-associated protein AHNAK	S5780	72%	0.72	45	2	0	1	0	1	1	2	1	2	0	0	0	10
1988	AHNK_HUMAN	Neuroblast differentiation-associated protein AHNAK	S5782	49%	0.49	92	5	4	4	1	1	0	4	4	1	0	0	0	24

1989	AHNK_HUMAN	Neuroblast differentiation-associated protein AHNAK	S5784	12%	0.12	9	2	0	0	0	0	1	0	0	0	0	0	0	3
1990	AHNK_HUMAN	Neuroblast differentiation-associated protein AHNAK	S5841	92%	0.92	129	8	2	4	6	3	8	7	6	7	6	4	6	67
1991	AHNK_HUMAN	Neuroblast differentiation-associated protein AHNAK	T218	70%	0.70	61	1	0	0	3	2	1	4	1	3	1	4	2	22
1992	AHNK_HUMAN	Neuroblast differentiation-associated protein AHNAK	T4100	100%	1.00	74	0	0	0	0	0	0	0	0	0	2	2	0	4
1993	AHNK_HUMAN	Neuroblast differentiation-associated protein AHNAK	T5794	100%	1.00	96	0	0	0	1	1	1	0	0	1	3	2	2	11
1994	AHNK_HUMAN	Neuroblast differentiation-associated protein AHNAK	T5796	20%	0.20	19	0	0	0	2	0	0	0	0	0	1	0	0	3
1995	AHNK_HUMAN	Neuroblast differentiation-associated protein AHNAK	T5798	55%	0.55	27	0	0	0	3	0	0	0	0	0	0	1	2	6
1996	AHNK_HUMAN	Neuroblast differentiation-associated protein AHNAK	T5839	37%	0.37	10	0	1	2	0	1	0	0	1	2	0	0	1	8
1997	NOTC1_HUMAN	Neurogenic locus notch homolog protein 1	T2371	16%	0.16	1	0	0	0	0	0	1	0	0	0	0	0	0	1
1998	NAV1_HUMAN	Neuron navigator 1	S648	97%	0.97	5	0	0	0	1	0	0	0	0	0	0	0	0	1
1999	NAV1_HUMAN	Neuron navigator 1	S981	57%	0.57	1	0	0	0	1	0	0	0	0	0	0	0	0	1
2000	NAV1_HUMAN	Neuron navigator 1	T647	25%	0.25	1	0	0	0	1	0	0	0	0	0	0	0	0	1
2001	ACHA3_HUMAN	Neuronal acetylcholine receptor subunit alpha-3	S13	45%	0.45	1	0	0	0	0	0	0	0	0	0	1	0	0	1
2002	ACHA3_HUMAN	Neuronal acetylcholine receptor subunit alpha-3	S24	45%	0.45	1	0	0	0	0	0	0	0	0	0	1	0	0	1
2003	GPM6A_HUMAN	Neuronal membrane glycoprotein M6-a	T210	44%	0.44	1	0	0	0	0	0	0	1	0	0	0	0	0	1
2004	GPM6A_HUMAN	Neuronal membrane glycoprotein M6-a	Y241	31%	0.31	1	0	0	0	0	0	0	1	0	0	0	0	0	1
2005	NPY2R_HUMAN	Neuropeptide Y receptor type 2	S317	50%	0.50	1	0	0	0	0	0	0	0	1	0	0	0	0	1
2006	NPY2R_HUMAN	Neuropeptide Y receptor type 2	S330	66%	0.66	1	0	0	0	0	0	0	0	1	0	0	0	0	1

2007	NPY2R_HUMAN	Neuropeptide Y receptor type 2	T318	96%	0.96	1	0	0	0	0	0	0	0	1	0	0	0	0	1
2008	NPY2R_HUMAN	Neuropeptide Y receptor type 2	Y332	64%	0.64	1	0	0	0	0	0	0	0	1	0	0	0	0	1
2009	NKAP_HUMAN	NF-kappa-B-activating protein	S149	100%	1.00	69	4	5	3	0	1	0	4	0	2	0	2	2	23
2010	NAB2_HUMAN	NGFI-A-binding protein 2	S479	100%	1.00	2	1	0	0	0	0	0	1	0	0	0	0	0	2
2011	NAB2_HUMAN	NGFI-A-binding protein 2	T434	100%	1.00	1	0	0	0	0	0	0	0	0	0	0	0	1	1
2012	NHSL1_HUMAN	NHS-like protein 1	S328	100%	1.00	4	0	2	0	0	0	0	0	0	0	0	1	0	3
2013	NHSL1_HUMAN	NHS-like protein 1	S941	100%	1.00	12	0	0	0	0	0	0	0	0	0	1	0	1	2
2014	NIBL1_HUMAN	Niban-like protein 1	S638	55%	0.55	8	0	0	0	0	0	0	0	0	0	1	0	1	2
2015	NIBL1_HUMAN	Niban-like protein 1	S641	100%	1.00	448	2	2	3	10	10	13	12	8	13	18	22	14	127
2016	NIBL1_HUMAN	Niban-like protein 1	S646	100%	1.00	697	4	3	2	21	15	20	14	15	22	20	22	18	176
2017	NIBL1_HUMAN	Niban-like protein 1	S665	100%	1.00	####	24	22	24	49	49	44	48	38	53	63	56	47	517
2018	NIBL1_HUMAN	Niban-like protein 1	S681	100%	1.00	453	6	2	2	14	12	10	10	11	11	21	20	18	137
2019	NIBL1_HUMAN	Niban-like protein 1	S691	92%	0.92	66	0	0	0	1	4	3	0	4	1	0	2	1	16
2020	NIBL1_HUMAN	Niban-like protein 1	S692	100%	1.00	583	0	1	0	8	11	10	8	11	10	14	14	8	95
2021	NIBL1_HUMAN	Niban-like protein 1	S696	100%	1.00	254	0	0	1	3	8	4	2	4	4	7	7	7	47
2022	NIBL1_HUMAN	Niban-like protein 1	T651	88%	0.88	91	1	0	0	0	5	3	2	4	2	5	2	3	27
2023	NIBL2_HUMAN	Niban-like protein 2	S317	100%	1.00	1	0	0	0	0	0	0	0	1	0	0	0	0	1
2024	NMNA1_HUMAN	Nicotinamide mononucleotide adenylyltransferase 1	S117	89%	0.89	5	0	0	0	2	1	0	0	0	0	1	0	0	4
2025	NMNA1_HUMAN	Nicotinamide mononucleotide adenylyltransferase 1	T119	78%	0.78	2	0	0	0	0	0	0	1	0	0	0	0	1	2

2026	PNCB_HUMAN	Nicotinate phosphoribosyltransferase	S537	100%	1.00	1	0	0	0	0	0	0	0	0	0	0	0	1	1
2027	NPCL1_HUMAN	Niemann-Pick C1-like protein 1	S1237	84%	0.84	1	0	0	1	0	0	0	0	0	0	0	0	0	1
2028	NPCL1_HUMAN	Niemann-Pick C1-like protein 1	T1232	94%	0.94	1	0	0	1	0	0	0	0	0	0	0	0	0	1
2029	NPCL1_HUMAN	Niemann-Pick C1-like protein 1	T1246	61%	0.61	1	0	0	1	0	0	0	0	0	0	0	0	0	1
2030	NIPBL_HUMAN	Nipped-B-like protein	S2658	100%	1.00	2	0	0	0	0	0	0	0	0	0	2	0	0	2
2031	NIPBL_HUMAN	Nipped-B-like protein	S318	100%	1.00	6	0	0	0	1	0	0	0	1	0	0	0	0	2
2032	NKTR_HUMAN	NK-tumor recognition protein	S613	93%	0.93	5	0	0	1	1	0	0	0	0	0	0	0	0	2
2033	NOMO1_HUMAN	Nodal modulator 1	T588	18%	0.18	1	0	0	0	0	0	0	0	0	0	0	0	1	1
2034	NONO_HUMAN	Non-POU domain-containing octamer-binding protein	T428	83%	0.83	6	0	1	0	0	0	0	0	0	0	0	1	0	2
2035	NONO_HUMAN	Non-POU domain-containing octamer-binding protein	T450	100%	1.00	230	5	7	6	9	5	4	3	6	4	8	10	7	74
2036	NLTP_HUMAN	Non-specific lipid-transfer protein	S249	21%	0.21	1	0	0	0	1	0	0	0	0	0	0	0	0	1
2037	NLTP_HUMAN	Non-specific lipid-transfer protein	S265	81%	0.81	1	0	0	0	1	0	0	0	0	0	0	0	0	1
2038	NLTP_HUMAN	Non-specific lipid-transfer protein	S266	93%	0.93	1	0	0	0	1	0	0	0	0	0	0	0	0	1
2039	NSF1C_HUMAN	NSFL1 cofactor p47	S114	100%	1.00	301	6	9	9	14	9	8	10	6	6	0	0	0	77
2040	SP100_HUMAN	Nuclear autoantigen Sp-100	S157	100%	1.00	6	0	0	0	1	0	0	1	0	2	1	0	0	5
2041	NASP_HUMAN	Nuclear autoantigenic sperm protein	S28	25%	0.25	1	1	0	0	0	0	0	0	0	0	0	0	0	1
2042	NASP_HUMAN	Nuclear autoantigenic sperm protein	S503	100%	1.00	48	2	2	1	0	2	0	3	2	2	0	0	0	14
2043	NASP_HUMAN	Nuclear autoantigenic sperm protein	T29	31%	0.31	1	1	0	0	0	0	0	0	0	0	0	0	0	1
2044	NCBP1_HUMAN	Nuclear cap-binding protein subunit 1	S22	99%	0.99	63	0	0	0	0	2	0	0	0	0	0	0	0	2

2045	NEMF_HUMAN	Nuclear export mediator factor NEMF	S831	100%	1.00	4	0	0	0	0	0	0	0	0	0	0	0	2	2
2046	NFIB_HUMAN	Nuclear factor 1 B-type	S326	9%	0.09	2	0	0	0	0	0	0	0	0	0	0	1	0	1
2047	NFIB_HUMAN	Nuclear factor 1 B-type	S328	100%	1.00	28	1	0	0	0	0	0	0	1	0	4	5	3	14
2048	NFIB_HUMAN	Nuclear factor 1 B-type	T359	97%	0.97	5	1	1	0	0	0	0	0	0	0	1	1	1	5
2049	NFKB1_HUMAN	Nuclear factor NF-kappa-B p105 subunit	S855	70%	0.70	1	0	0	0	0	0	0	0	0	0	0	0	1	1
2050	NFKB1_HUMAN	Nuclear factor NF-kappa-B p105 subunit	S937	98%	0.98	15	0	0	2	2	0	0	0	0	0	2	0	0	6
2051	NFKB1_HUMAN	Nuclear factor NF-kappa-B p105 subunit	T857	57%	0.57	1	0	0	0	0	0	0	0	0	0	0	0	1	1
2052	NFKB1_HUMAN	Nuclear factor NF-kappa-B p105 subunit	Y862	64%	0.64	1	0	0	0	0	0	0	0	0	0	0	0	1	1
2053	NUFP2_HUMAN	Nuclear fragile X mental retardation-interacting protein 2	S572	100%	1.00	1	0	0	0	0	0	0	0	0	0	0	1	0	1
2054	NUFP2_HUMAN	Nuclear fragile X mental retardation-interacting protein 2	S629	100%	1.00	241	0	0	1	4	3	1	1	0	1	6	7	4	28
2055	NUFP2_HUMAN	Nuclear fragile X mental retardation-interacting protein 2	S637	57%	0.57	15	0	0	0	2	0	0	0	0	0	0	0	1	3
2056	NUFP2_HUMAN	Nuclear fragile X mental retardation-interacting protein 2	T631	54%	0.54	25	0	0	0	0	1	0	0	0	0	0	1	1	3
2057	NUFP2_HUMAN	Nuclear fragile X mental retardation-interacting protein 2	T633	39%	0.39	42	0	0	0	0	0	1	0	0	0	1	0	2	4
2058	NUMA1_HUMAN	Nuclear mitotic apparatus protein 1	S1225	100%	1.00	363	7	2	5	4	6	4	4	6	8	1	6	4	57
2059	NUMA1_HUMAN	Nuclear mitotic apparatus protein 1	S1229	75%	0.75	47	1	0	2	0	1	0	0	0	2	1	2	0	9
2060	NUMA1_HUMAN	Nuclear mitotic apparatus protein 1	S169	100%	1.00	142	1	3	2	3	3	1	2	3	0	2	0	1	21
2061	NUMA1_HUMAN	Nuclear mitotic apparatus protein 1	S172	94%	0.94	25	1	0	0	0	0	0	0	0	1	0	0	0	2

2062	NU133_HUMAN	Nuclear pore complex protein Nup133	S50	100%	1.00	4	0	0	0	0	0	0	0	0	0	0	1	0	1
2063	NU153_HUMAN	Nuclear pore complex protein Nup153	S333	91%	0.91	5	0	0	1	0	0	0	1	0	0	0	0	0	2
2064	NU153_HUMAN	Nuclear pore complex protein Nup153	S334	100%	1.00	23	0	0	0	0	0	0	0	0	1	0	0	0	1
2065	NU153_HUMAN	Nuclear pore complex protein Nup153	S518	25%	0.25	4	0	0	0	0	0	0	0	0	0	1	0	0	1
2066	NU155_HUMAN	Nuclear pore complex protein Nup155	S1005	95%	0.95	1	0	0	0	0	0	0	1	0	0	0	0	0	1
2067	NU155_HUMAN	Nuclear pore complex protein Nup155	S1006	90%	0.90	1	0	0	0	0	0	0	1	0	0	0	0	0	1
2068	NU155_HUMAN	Nuclear pore complex protein Nup155	S1012	97%	0.97	1	0	0	0	0	0	0	1	0	0	0	0	0	1
2069	NU155_HUMAN	Nuclear pore complex protein Nup155	S3	86%	0.86	4	0	1	0	1	0	0	0	0	0	0	0	0	2
2070	NU155_HUMAN	Nuclear pore complex protein Nup155	S4	70%	0.70	35	0	1	1	1	1	2	0	0	1	3	3	3	16
2071	NU155_HUMAN	Nuclear pore complex protein Nup155	S992	93%	0.93	1	0	0	0	0	0	0	1	0	0	0	0	0	1
2072	NU214_HUMAN	Nuclear pore complex protein Nup214	S1083	35%	0.35	6	0	0	0	0	0	0	0	0	0	1	2	0	3
2073	NU214_HUMAN	Nuclear pore complex protein Nup214	S450	9%	0.09	42	3	1	0	2	0	1	1	1	1	3	4	1	18
2074	NU214_HUMAN	Nuclear pore complex protein Nup214	S457	77%	0.77	58	0	5	0	2	0	2	3	4	1	4	3	0	24
2075	NU214_HUMAN	Nuclear pore complex protein Nup214	S461	41%	0.41	10	0	1	0	2	0	0	0	0	1	0	0	0	4
2076	NU214_HUMAN	Nuclear pore complex protein Nup214	S462	92%	0.92	56	0	4	0	1	0	1	1	2	0	5	4	1	19
2077	NU214_HUMAN	Nuclear pore complex protein Nup214	S471	9%	0.09	2	1	0	0	0	0	0	0	0	0	0	0	0	1
2078	NUP50_HUMAN	Nuclear pore complex protein Nup50	S255	100%	1.00	2	0	0	0	0	0	0	0	0	0	2	0	0	2

2079	NUP98_HUMAN	Nuclear pore complex protein Nup98-Nup96	S618	0%	0.00	1	0	0	0	0	0	1	0	0	0	0	0	0	1
2080	NUP98_HUMAN	Nuclear pore complex protein Nup98-Nup96	S623	100%	1.00	12	2	0	0	0	1	0	1	1	1	0	0	0	6
2081	NUP62_HUMAN	Nuclear pore glycoprotein p62	S408	100%	1.00	48	0	0	0	0	2	0	0	1	0	0	0	0	3
2082	NCOA2_HUMAN	Nuclear receptor coactivator 2	T1273	28%	0.28	1	0	0	0	0	0	0	0	1	0	0	0	0	1
2083	NCOA3_HUMAN	Nuclear receptor coactivator 3	S1360	91%	0.91	2	0	0	0	1	0	0	0	0	0	0	0	0	1
2084	NCOA3_HUMAN	Nuclear receptor coactivator 3	S857	100%	1.00	5	0	2	0	0	0	0	2	0	0	0	0	0	4
2085	NCOA5_HUMAN	Nuclear receptor coactivator 5	S9	77%	0.77	5	0	0	0	1	1	0	0	0	0	0	0	0	2
2086	NCOA5_HUMAN	Nuclear receptor coactivator 5	T11	100%	1.00	5	0	2	0	0	0	0	0	1	0	0	0	0	3
2087	NCOA5_HUMAN	Nuclear receptor coactivator 5	T3	25%	0.25	13	0	2	2	0	1	0	0	0	1	0	1	1	8
2088	NCOR1_HUMAN	Nuclear receptor corepressor 1	S2184	100%	1.00	66	0	2	4	2	4	2	3	0	3	0	2	1	23
2089	NCOR1_HUMAN	Nuclear receptor corepressor 1	T1672	84%	0.84	1	0	0	0	1	0	0	0	0	0	0	0	0	1
2090	NCOR2_HUMAN	Nuclear receptor corepressor 2	S1018	100%	1.00	29	2	0	1	1	1	0	0	2	1	1	2	2	13
2091	NCOR2_HUMAN	Nuclear receptor corepressor 2	S149	100%	1.00	3	0	0	0	0	0	0	0	1	0	0	0	0	1
2092	NCOR2_HUMAN	Nuclear receptor corepressor 2	S2016	100%	1.00	20	0	0	0	3	1	2	2	2	1	0	0	0	11
2093	NCOR2_HUMAN	Nuclear receptor corepressor 2	T156	94%	0.94	3	0	0	0	0	0	0	0	1	0	0	0	0	1
2094	NRBP_HUMAN	Nuclear receptor-binding protein	S2	86%	0.86	13	0	2	0	0	0	0	0	0	0	0	2	0	4
2095	NXF1_HUMAN	Nuclear RNA export factor 1	S556	53%	0.53	1	0	0	0	0	0	0	0	0	0	0	1	0	1
2096	NUCKS_HUMAN	Nuclear ubiquitous casein and cyclin-dependent kinases substrate	S181	100%	1.00	6	0	0	0	0	0	0	0	0	0	2	0	4	6
2097	NUCKS_HUMAN	Nuclear ubiquitous casein and cyclin-dependent kinases substrate	S19	100%	1.00	674	32	22	21	16	19	14	22	21	22	11	9	17	226

2098	NIPA_HUMAN	Nuclear-interacting partner of ALK	S321	100%	1.00	10	0	1	0	0	0	0	0	0	0	0	0	3	4
2099	NIPA_HUMAN	Nuclear-interacting partner of ALK	S395	93%	0.93	10	2	2	0	0	1	0	0	0	0	0	1	1	7
2100	YBOX1_HUMAN	Nuclease-sensitive element-binding protein 1	S102	100%	1.00	57	0	0	0	2	0	0	1	0	0	6	6	6	21
2101	NOLC1_HUMAN	Nucleolar and coiled-body phosphoprotein 1	S698	100%	1.00	221	5	5	1	1	2	2	1	0	2	1	1	0	21
2102	NOC2L_HUMAN	Nucleolar complex protein 2 homolog	S496	100%	1.00	3	0	0	0	0	0	0	0	0	0	0	1	0	1
2103	NOC2L_HUMAN	Nucleolar complex protein 2 homolog	S497	100%	1.00	3	0	0	0	0	0	0	0	0	0	0	1	0	1
2104	NOC2L_HUMAN	Nucleolar complex protein 2 homolog	S503	100%	1.00	3	0	0	0	0	0	0	0	0	0	0	1	0	1
2105	NOC2L_HUMAN	Nucleolar complex protein 2 homolog	S672	100%	1.00	239	3	5	6	5	8	8	3	2	6	3	2	1	52
2106	NOC2L_HUMAN	Nucleolar complex protein 2 homolog	S673	100%	1.00	236	3	5	6	3	8	8	3	2	6	3	2	0	49
2107	NOP56_HUMAN	Nucleolar protein 56	S519	7%	0.07	2	1	0	0	0	0	0	0	0	0	0	0	0	1
2108	NOP56_HUMAN	Nucleolar protein 56	S520	99%	0.99	72	2	2	4	3	2	3	2	1	2	2	0	0	23
2109	NOP56_HUMAN	Nucleolar protein 56	S570	97%	0.97	3	0	2	0	0	0	0	0	0	0	0	0	0	2
2110	NOP58_HUMAN	Nucleolar protein 58	S502	100%	1.00	438	13	11	11	9	8	8	8	7	8	6	3	7	99
2111	NOP58_HUMAN	Nucleolar protein 58	S514	100%	1.00	102	2	2	2	0	1	0	0	0	0	0	0	0	7
2112	NOP58_HUMAN	Nucleolar protein 58	T508	86%	0.86	2	2	0	0	0	0	0	0	0	0	0	0	0	2
2113	DDX21_HUMAN	Nucleolar RNA helicase 2	S121	100%	1.00	63	3	8	5	4	7	7	4	3	1	1	0	0	43
2114	DDX21_HUMAN	Nucleolar RNA helicase 2	S164	94%	0.94	4	0	0	0	0	0	0	0	0	0	0	1	0	1
2115	DDX21_HUMAN	Nucleolar RNA helicase 2	S168	100%	1.00	26	0	0	0	1	1	0	0	0	0	0	0	0	2
2116	DDX21_HUMAN	Nucleolar RNA helicase 2	S171	100%	1.00	26	0	0	0	1	1	0	0	0	0	0	1	0	3

2117	NUCL_HUMAN	Nucleolin	S145	100%	1.00	99	4	11	4	0	3	2	5	4	3	0	3	0	39
2118	NUCL_HUMAN	Nucleolin	S153	100%	1.00	99	4	11	4	0	3	2	5	4	3	0	3	0	39
2119	NUCL_HUMAN	Nucleolin	S67	100%	1.00	15	1	1	4	0	3	1	1	0	0	1	0	3	15
2120	NUCL_HUMAN	Nucleolin	T69	100%	1.00	11	1	0	6	0	0	0	0	0	0	2	0	2	11
2121	NPM_HUMAN	Nucleophosmin	S10	100%	1.00	818	22	18	16	16	16	14	12	15	17	19	19	11	195
2122	NPM_HUMAN	Nucleophosmin	S106	73%	0.73	75	0	0	1	1	2	2	2	4	1	1	2	0	16
2123	NPM_HUMAN	Nucleophosmin	S112	67%	0.67	96	2	0	2	3	2	0	0	2	1	0	2	0	14
2124	NPM_HUMAN	Nucleophosmin	S125	100%	1.00	####	###	###	###	###	###	###	###	###	###	###	###	###	###
2125	NPM_HUMAN	Nucleophosmin	S137	71%	0.71	9	0	0	0	0	0	1	0	0	0	0	0	0	1
2126	NPM_HUMAN	Nucleophosmin	S139	20%	0.20	8	2	1	1	0	0	0	2	0	0	0	0	0	6
2127	NPM_HUMAN	Nucleophosmin	S4	100%	1.00	90	7	5	3	1	7	1	0	1	2	7	7	0	41
2128	NPM_HUMAN	Nucleophosmin	S70	100%	1.00	955	27	21	28	21	7	16	20	21	22	21	24	31	259
2129	NPM_HUMAN	Nucleophosmin	T75	93%	0.93	96	2	3	1	1	1	0	2	1	1	1	3	1	17
2130	NPM_HUMAN	Nucleophosmin	T78	55%	0.55	26	0	0	0	0	0	0	0	1	0	1	0	2	4
2131	NU188_HUMAN	Nucleoporin NUP188 homolog	S1709	95%	0.95	2	0	0	0	0	0	0	0	0	0	0	0	1	1
2132	TPR_HUMAN	Nucleoprotein TPR	S2155	100%	1.00	249	2	3	1	2	3	0	5	2	5	7	8	4	42
2133	TPR_HUMAN	Nucleoprotein TPR	S379	100%	1.00	38	0	0	1	2	0	0	0	0	0	5	4	0	12
2134	TPR_HUMAN	Nucleoprotein TPR	T2116	100%	1.00	145	0	0	0	0	0	1	1	0	0	2	4	3	11
2135	TPR_HUMAN	Nucleoprotein TPR	T2146	100%	1.00	27	1	0	0	0	0	0	1	0	1	1	0	0	4
2136	TPR_HUMAN	Nucleoprotein TPR	T641	100%	1.00	50	0	1	0	0	0	0	1	0	0	0	1	0	3

2137	NXN_HUMAN	Nucleoredoxin	S401	85%	0.85	1	0	0	0	0	1	0	0	0	0	0	0	0	1
2138	NXN_HUMAN	Nucleoredoxin	T386	59%	0.59	1	0	0	0	0	1	0	0	0	0	0	0	0	1
2139	NXN_HUMAN	Nucleoredoxin	T396	96%	0.96	1	0	0	0	0	1	0	0	0	0	0	0	0	1
2140	NDKA_HUMAN	Nucleoside diphosphate kinase A	T94	99%	0.99	1	0	0	0	0	0	0	0	0	0	0	0	1	1
2141	NDKB_HUMAN	Nucleoside diphosphate kinase B	T94	99%	0.99	1	0	0	0	0	0	0	0	0	0	0	0	1	1
2142	NP1L4_HUMAN	Nucleosome assembly protein 1-like 4	S12	97%	0.97	19	0	0	0	1	2	0	0	0	2	1	1	1	8
2143	NP1L4_HUMAN	Nucleosome assembly protein 1-like 4	S125	97%	0.97	34	2	3	0	0	0	0	0	0	0	0	0	0	5
2144	NP1L4_HUMAN	Nucleosome assembly protein 1-like 4	S5	100%	1.00	51	0	0	1	2	1	1	1	0	1	2	2	2	13
2145	NP1L4_HUMAN	Nucleosome assembly protein 1-like 4	S7	100%	1.00	35	2	1	1	1	0	0	1	1	0	0	0	2	9
2146	BPTF_HUMAN	Nucleosome-remodeling factor subunit BPTF	S216	100%	1.00	3	0	1	1	0	0	0	0	0	0	0	0	0	2
2147	O10Z1_HUMAN	Olfactory receptor 10Z1	S291	30%	0.30	1	0	0	0	1	0	0	0	0	0	0	0	0	1
2148	O10Z1_HUMAN	Olfactory receptor 10Z1	T279	30%	0.30	1	0	0	0	1	0	0	0	0	0	0	0	0	1
2149	OR1A1_HUMAN	Olfactory receptor 1A1	S89	32%	0.32	1	0	0	0	0	0	1	0	0	0	0	0	0	1
2150	OR1A1_HUMAN	Olfactory receptor 1A1	S91	32%	0.32	1	0	0	0	0	0	1	0	0	0	0	0	0	1
2151	OR1A1_HUMAN	Olfactory receptor 1A1	Y120	32%	0.32	1	0	0	0	0	0	1	0	0	0	0	0	0	1
2152	O2AG1_HUMAN	Olfactory receptor 2AG1	S148	32%	0.32	35	0	2	1	1	2	0	0	1	0	0	0	0	7
2153	O2AG1_HUMAN	Olfactory receptor 2AG1	T147	32%	0.32	35	0	2	1	1	2	0	0	1	0	0	0	0	7
2154	O2AG1_HUMAN	Olfactory receptor 2AG1	T163	69%	0.69	35	0	2	1	1	2	0	0	1	0	0	0	0	7
2155	OR4CG_HUMAN	Olfactory receptor 4C16	S65	27%	0.27	1	0	0	0	0	0	0	0	0	0	1	0	0	1

2156	OR4CG_HUMAN	Olfactory receptor 4C16	S67	86%	0.86	1	0	0	0	0	0	0	0	0	0	1	0	0	1
2157	OR4CG_HUMAN	Olfactory receptor 4C16	T69	54%	0.54	1	0	0	0	0	0	0	0	0	0	1	0	0	1
2158	O4F15_HUMAN	Olfactory receptor 4F15	S104	99%	0.99	4	0	0	0	0	0	0	0	0	0	1	1	0	2
2159	O4F15_HUMAN	Olfactory receptor 4F15	T110	96%	0.96	4	0	0	0	0	0	0	0	0	0	1	1	0	2
2160	OR4S1_HUMAN	Olfactory receptor 4S1	S65	22%	0.22	1	0	0	0	1	0	0	0	0	0	0	0	0	1
2161	OR4S1_HUMAN	Olfactory receptor 4S1	Y71	22%	0.22	1	0	0	0	1	0	0	0	0	0	0	0	0	1
2162	OR4S2_HUMAN	Olfactory receptor 4S2	T8	99%	0.99	4	0	0	0	0	0	0	0	0	1	0	0	0	1
2163	O51A2_HUMAN	Olfactory receptor 51A2	T139	34%	0.34	1	0	0	0	0	0	0	0	0	0	0	0	1	1
2164	O51A2_HUMAN	Olfactory receptor 51A2	T140	34%	0.34	1	0	0	0	0	0	0	0	0	0	0	0	1	1
2165	O51A4_HUMAN	Olfactory receptor 51A4	T139	34%	0.34	1	0	0	0	0	0	0	0	0	0	0	0	1	1
2166	O51A4_HUMAN	Olfactory receptor 51A4	T140	34%	0.34	1	0	0	0	0	0	0	0	0	0	0	0	1	1
2167	O51V1_HUMAN	Olfactory receptor 51V1	S199	41%	0.41	1	0	1	0	0	0	0	0	0	0	0	0	0	1
2168	O51V1_HUMAN	Olfactory receptor 51V1	S205	48%	0.48	1	0	1	0	0	0	0	0	0	0	0	0	0	1
2169	O51V1_HUMAN	Olfactory receptor 51V1	S226	36%	0.36	1	0	1	0	0	0	0	0	0	0	0	0	0	1
2170	OR6C4_HUMAN	Olfactory receptor 6C4	S247	50%	0.50	1	0	0	0	0	0	0	0	1	0	0	0	0	1
2171	OR6C4_HUMAN	Olfactory receptor 6C4	S249	50%	0.50	1	0	0	0	0	0	0	0	1	0	0	0	0	1
2172	OR6C4_HUMAN	Olfactory receptor 6C4	S252	50%	0.50	1	0	0	0	0	0	0	0	1	0	0	0	0	1
2173	OR6C4_HUMAN	Olfactory receptor 6C4	S261	50%	0.50	1	0	0	0	0	0	0	0	1	0	0	0	0	1
2174	OR6C4_HUMAN	Olfactory receptor 6C4	Y250	50%	0.50	1	0	0	0	0	0	0	0	1	0	0	0	0	1
2175	OMGP_HUMAN	Oligodendrocyte-myelin glycoprotein	S275	12%	0.12	1	0	1	0	0	0	0	0	0	0	0	0	0	1

2176	OMGP_HUMAN	Oligodendrocyte-myelin glycoprotein	S276	12%	0.12	1	0	1	0	0	0	0	0	0	0	0	0	0	1
2177	OGFR_HUMAN	Opioid growth factor receptor	S484	95%	0.95	18	0	2	2	0	0	1	0	1	0	0	0	0	6
2178	ORC6_HUMAN	Origin recognition complex subunit 6	T195	100%	1.00	54	0	0	1	1	0	0	0	0	0	0	0	0	2
2179	OTOG_HUMAN	Otogelin	S974	97%	0.97	1	0	0	0	0	0	0	0	0	0	0	0	1	1
2180	OTOG_HUMAN	Otogelin	T978	97%	0.97	1	0	0	0	0	0	0	0	0	0	0	0	1	1
2181	OTUD5_HUMAN	OTU domain-containing protein 5	S64	100%	1.00	52	3	3	3	3	2	3	3	3	4	5	4	3	39
2182	OXR1_HUMAN	Oxidation resistance protein 1	S204	86%	0.86	2	0	0	0	0	0	0	0	0	0	0	1	0	1
2183	OSBP1_HUMAN	Oxysterol-binding protein 1	S190	100%	1.00	53	6	4	0	6	6	0	0	0	2	2	4	0	30
2184	OSBP1_HUMAN	Oxysterol-binding protein 1	S193	100%	1.00	53	6	4	0	6	6	0	0	0	2	2	4	0	30
2185	OSBP1_HUMAN	Oxysterol-binding protein 1	S351	100%	1.00	394	11	8	14	10	11	8	9	7	8	16	7	8	117
2186	OSB10_HUMAN	Oxysterol-binding protein-related protein 10	S14	64%	0.64	1	0	0	0	0	0	0	0	0	0	1	0	0	1
2187	OSB10_HUMAN	Oxysterol-binding protein-related protein 10	S18	29%	0.29	1	0	0	0	0	0	0	0	0	0	1	0	0	1
2188	OSB11_HUMAN	Oxysterol-binding protein-related protein 11	S189	100%	1.00	37	2	6	3	0	0	2	2	2	2	4	2	4	29
2189	OSBL3_HUMAN	Oxysterol-binding protein-related protein 3	S303	25%	0.25	5	0	0	1	0	0	0	0	0	0	0	1	0	2
2190	OSBL3_HUMAN	Oxysterol-binding protein-related protein 3	S304	100%	1.00	81	0	1	0	1	2	2	3	1	0	0	1	0	11
2191	OSBL3_HUMAN	Oxysterol-binding protein-related protein 3	S410	100%	1.00	13	0	2	0	0	0	0	1	0	0	0	0	2	5
2192	OSBL3_HUMAN	Oxysterol-binding protein-related protein 3	T310	25%	0.25	5	0	0	0	0	0	0	1	0	0	0	0	0	1
2193	OSBL5_HUMAN	Oxysterol-binding protein-related protein 5	S119	75%	0.75	1	0	0	0	0	0	1	0	0	0	0	0	0	1

2194	OSBL8_HUMAN	Oxysterol-binding protein-related protein 8	S601	22%	0.22	1	0	0	0	0	0	0	0	0	0	1	0	0	1
2195	OSBL8_HUMAN	Oxysterol-binding protein-related protein 8	S622	92%	0.92	1	0	0	0	0	0	0	0	0	0	1	0	0	1
2196	OSBL9_HUMAN	Oxysterol-binding protein-related protein 9	T606	100%	1.00	1	0	0	0	0	0	0	0	0	0	1	0	0	1
2197	P2RY8_HUMAN	P2Y purinoceptor 8	S327	100%	1.00	1	0	0	0	0	0	0	0	0	0	1	0	0	1
2198	PALLD_HUMAN	Palladin	S684	100%	1.00	2	0	0	1	0	0	0	0	0	0	0	1	0	2
2199	PALLD_HUMAN	Palladin	S688	100%	1.00	2	0	0	1	0	0	0	0	0	0	0	1	0	2
2200	PALLD_HUMAN	Palladin	S893	100%	1.00	19	0	0	0	0	0	0	0	0	0	4	2	2	8
2201	PANX2_HUMAN	Pannexin-2	S539	100%	1.00	1	0	0	0	0	0	0	0	0	0	0	0	1	1
2202	VNN1_HUMAN	Pantetheinase	S419	100%	1.00	1	0	0	0	0	0	0	0	0	0	0	0	1	1
2203	VNN1_HUMAN	Pantetheinase	T405	94%	0.94	1	0	0	0	0	0	0	0	0	0	0	0	1	1
2204	VNN1_HUMAN	Pantetheinase	T406	96%	0.96	1	0	0	0	0	0	0	0	0	0	0	0	1	1
2205	VNN1_HUMAN	Pantetheinase	T417	100%	1.00	1	0	0	0	0	0	0	0	0	0	0	0	1	1
2206	VNN1_HUMAN	Pantetheinase	T420	100%	1.00	1	0	0	0	0	0	0	0	0	0	0	0	1	1
2207	PANK2_HUMAN	Pantothenate kinase 2, mitochondrial	S189	75%	0.75	1	0	1	0	0	0	0	0	0	0	0	0	0	1
2208	PALM2_HUMAN	Paralemmin-2	S228	100%	1.00	1	0	0	0	0	0	0	0	0	0	0	0	1	1
2209	PALM2_HUMAN	Paralemmin-2	S230	100%	1.00	1	0	0	0	0	0	0	0	0	0	0	0	1	1
2210	PTMS_HUMAN	Parathymosin	S2	99%	0.99	17	2	2	0	0	1	0	2	2	1	0	0	0	10
2211	PAR6B_HUMAN	Partitioning defective 6 homolog beta	S205	39%	0.39	1	1	0	0	0	0	0	0	0	0	0	0	0	1
2212	PAR6B_HUMAN	Partitioning defective 6 homolog beta	S239	33%	0.33	1	1	0	0	0	0	0	0	0	0	0	0	0	1

2213	PAXI_HUMAN	Paxillin	S170	60%	0.60	42	3	4	0	0	0	2	0	0	0	1	3	0	13
2214	PAXI_HUMAN	Paxillin	S244	99%	0.99	19	0	0	0	0	1	0	0	0	0	0	0	0	1
2215	PAXI_HUMAN	Paxillin	S85	100%	1.00	12	0	0	0	0	0	0	0	0	0	2	0	0	2
2216	PA1_HUMAN	PAXIP1-associated protein 1	S234	2%	0.02	6	1	0	0	0	0	0	0	0	0	0	0	1	2
2217	PA1_HUMAN	PAXIP1-associated protein 1	S237	100%	1.00	119	5	5	3	3	4	4	6	2	2	3	1	2	40
2218	PCTL_HUMAN	PCTP-like protein	T8	97%	0.97	1	0	0	0	0	0	0	0	0	0	1	0	0	1
2219	PDLI2_HUMAN	PDZ and LIM domain protein 2	S134	99%	0.99	5	1	0	0	0	0	0	0	0	0	0	0	0	1
2220	PDLI2_HUMAN	PDZ and LIM domain protein 2	S137	96%	0.96	6	0	0	0	0	0	0	0	0	0	3	2	1	6
2221	PDLI2_HUMAN	PDZ and LIM domain protein 2	S161	51%	0.51	1	0	0	0	0	0	0	0	0	0	0	1	0	1
2222	PDLI2_HUMAN	PDZ and LIM domain protein 2	S197	100%	1.00	207	1	0	0	6	4	5	1	1	3	1	6	1	29
2223	PDLI4_HUMAN	PDZ and LIM domain protein 4	S112	100%	1.00	242	10	13	10	6	6	9	5	13	12	0	0	0	84
2224	PDLI4_HUMAN	PDZ and LIM domain protein 4	S116	56%	0.56	60	2	0	1	3	2	3	1	2	3	0	0	0	17
2225	PDLI4_HUMAN	PDZ and LIM domain protein 4	S120	70%	0.70	20	4	5	0	1	2	0	3	0	1	0	0	0	16
2226	PDLI4_HUMAN	PDZ and LIM domain protein 4	S165	84%	0.84	8	0	0	0	0	0	0	0	1	0	0	0	0	1
2227	PDLI4_HUMAN	PDZ and LIM domain protein 4	T114	53%	0.53	62	2	9	4	4	0	2	3	2	1	0	0	0	27
2228	PDLI4_HUMAN	PDZ and LIM domain protein 4	T115	25%	0.25	11	1	0	0	0	0	0	0	1	0	0	0	0	2
2229	PDLI4_HUMAN	PDZ and LIM domain protein 4	T122	51%	0.51	5	2	0	2	0	0	0	0	1	0	0	0	0	5
2230	GIPC1_HUMAN	PDZ domain-containing protein GIPC1	S225	100%	1.00	1	0	0	0	0	0	0	0	0	0	0	1	0	1
2231	GIPC1_HUMAN	PDZ domain-containing protein GIPC1	T238	94%	0.94	1	0	0	0	0	0	0	0	0	0	0	1	0	1

2232	FKBP5_HUMAN	Peptidyl-prolyl cis-trans isomerase FKBP5	S13	99%	0.99	7	3	0	1	0	0	0	0	0	0	0	0	0	4
2233	FKBP6_HUMAN	Peptidyl-prolyl cis-trans isomerase FKBP6	S20	100%	1.00	1	0	0	0	0	0	0	0	0	1	0	0	0	1
2234	FKBP6_HUMAN	Peptidyl-prolyl cis-trans isomerase FKBP6	Y22	100%	1.00	1	0	0	0	0	0	0	0	0	1	0	0	0	1
2235	PPIL4_HUMAN	Peptidyl-prolyl cis-trans isomerase-like 4	S178	100%	1.00	632	10	15	14	15	11	13	15	12	12	10	14	12	153
2236	PPIL4_HUMAN	Peptidyl-prolyl cis-trans isomerase-like 4	T182	97%	0.97	175	4	3	3	4	4	2	2	2	3	5	2	3	37
2237	PCM1_HUMAN	Pericentriolar material 1 protein	S65	100%	1.00	16	0	1	0	0	1	0	2	0	2	0	0	0	6
2238	PWP1_HUMAN	Periodic tryptophan protein 1 homolog	S50	100%	1.00	345	6	4	7	8	8	6	8	10	7	5	6	10	85
2239	PWP1_HUMAN	Periodic tryptophan protein 1 homolog	S65	99%	0.99	40	1	0	2	1	2	1	1	3	0	0	0	2	13
2240	PWP1_HUMAN	Periodic tryptophan protein 1 homolog	T68	97%	0.97	40	1	0	2	1	2	1	1	3	0	0	0	2	13
2241	POSTN_HUMAN	Periostin	T749	19%	0.19	1	0	0	0	0	0	0	0	0	0	0	0	1	1
2242	PPHLN_HUMAN	Periphilin-1	S133	100%	1.00	213	7	10	7	6	6	5	10	8	8	8	6	7	88
2243	PPHLN_HUMAN	Periphilin-1	S448	52%	0.52	1	0	0	0	0	0	0	1	0	0	0	0	0	1
2244	PPHLN_HUMAN	Periphilin-1	T429	23%	0.23	1	0	0	0	0	0	0	1	0	0	0	0	0	1
2245	PR285_HUMAN	Peroxisomal proliferator-activated receptor A-interacting complex 285 kDa protein	S873	97%	0.97	1	0	0	0	0	0	1	0	0	0	0	0	0	1
2246	PR285_HUMAN	Peroxisomal proliferator-activated receptor A-interacting complex 285 kDa protein	S883	97%	0.97	1	0	0	0	0	0	1	0	0	0	0	0	0	1
2247	PR285_HUMAN	Peroxisomal proliferator-activated receptor A-interacting complex 285 kDa protein	T877	96%	0.96	1	0	0	0	0	0	1	0	0	0	0	0	0	1

2248	PR285_HUMAN	Peroxisomal proliferator-activated receptor A-interacting complex 285 kDa protein	T893	100%	1.00	1	0	0	0	0	0	1	0	0	0	0	0	0	1
2249	PPRC1_HUMAN	Peroxisome proliferator-activated receptor gamma coactivator-related protein 1	S1480	100%	1.00	1	0	0	1	0	0	0	0	0	0	0	0	0	1
2250	PPRC1_HUMAN	Peroxisome proliferator-activated receptor gamma coactivator-related protein 1	S1481	96%	0.96	1	0	0	1	0	0	0	0	0	0	0	0	0	1
2251	PPRC1_HUMAN	Peroxisome proliferator-activated receptor gamma coactivator-related protein 1	S1482	85%	0.85	1	0	0	1	0	0	0	0	0	0	0	0	0	1
2252	PPRC1_HUMAN	Peroxisome proliferator-activated receptor gamma coactivator-related protein 1	S1483	5%	0.05	1	0	0	1	0	0	0	0	0	0	0	0	0	1
2253	PPRC1_HUMAN	Peroxisome proliferator-activated receptor gamma coactivator-related protein 1	S1484	5%	0.05	1	0	0	1	0	0	0	0	0	0	0	0	0	1
2254	PERQ2_HUMAN	PERQ amino acid-rich with GYF domain-containing protein 2	S20	4%	0.04	63	0	0	0	1	0	2	0	3	4	0	1	1	12
2255	PERQ2_HUMAN	PERQ amino acid-rich with GYF domain-containing protein 2	S26	100%	1.00	223	4	4	4	5	7	5	6	0	3	7	9	8	62
2256	PERQ2_HUMAN	PERQ amino acid-rich with GYF domain-containing protein 2	S377	7%	0.07	2	0	0	0	0	0	0	0	0	0	0	1	0	1
2257	PERQ2_HUMAN	PERQ amino acid-rich with GYF domain-containing protein 2	S384	32%	0.32	10	0	0	1	0	1	0	0	0	1	0	0	0	3
2258	PERQ2_HUMAN	PERQ amino acid-rich with GYF domain-containing protein 2	T25	0%	0.00	7	0	0	0	0	1	1	0	0	0	0	0	0	2
2259	PERQ2_HUMAN	PERQ amino acid-rich with GYF domain-containing protein 2	T382	90%	0.90	60	2	1	2	2	2	3	2	1	1	3	2	1	22

2260	PESC_HUMAN	Pescadillo homolog	S280	20%	0.20	14	0	0	1	1	0	0	1	0	0	1	1	0	5
2261	PESC_HUMAN	Pescadillo homolog	S282	51%	0.51	31	0	0	0	0	1	1	0	2	0	0	0	0	4
2262	PESC_HUMAN	Pescadillo homolog	T291	66%	0.66	9	0	0	0	0	0	1	0	0	0	0	0	0	1
2263	PHRF1_HUMAN	PHD and RING finger domain-containing protein 1	S1202	100%	1.00	13	0	0	1	0	0	0	0	0	0	0	0	1	2
2264	PHRF1_HUMAN	PHD and RING finger domain-containing protein 1	S1229	100%	1.00	13	0	0	0	2	0	1	0	0	1	0	0	3	7
2265	PHRF1_HUMAN	PHD and RING finger domain-containing protein 1	S1360	51%	0.51	1	0	0	0	0	0	0	0	0	1	0	0	0	1
2266	PHRF1_HUMAN	PHD and RING finger domain-containing protein 1	S915	100%	1.00	12	0	0	0	2	0	0	2	0	0	0	1	0	5
2267	PHRF1_HUMAN	PHD and RING finger domain-containing protein 1	T917	92%	0.92	241	3	8	6	6	6	3	5	6	4	5	6	2	60
2268	PHRF1_HUMAN	PHD and RING finger domain-containing protein 1	T923	2%	0.02	1	0	0	0	1	0	0	0	0	0	0	0	0	1
2269	LPIN1_HUMAN	Phosphatidate phosphatase LPIN1	S106	100%	1.00	39	2	1	0	0	0	2	2	2	1	5	4	5	24
2270	LPIN1_HUMAN	Phosphatidate phosphatase LPIN1	S110	11%	0.11	3	0	0	0	0	0	0	1	0	0	1	0	0	2
2271	LPIN1_HUMAN	Phosphatidate phosphatase LPIN1	T105	86%	0.86	4	0	0	0	0	0	0	0	1	0	1	1	0	3
2272	SMS1_HUMAN	Phosphatidylcholine:ceramide cholinephosphotransferase 1	T296	10%	0.10	1	0	0	0	0	1	0	0	0	0	0	0	0	1
2273	P85A_HUMAN	Phosphatidylinositol 3-kinase regulatory subunit alpha	S265	100%	1.00	1	0	1	0	0	0	0	0	0	0	0	0	0	1
2274	P85B_HUMAN	Phosphatidylinositol 3-kinase regulatory subunit beta	S263	98%	0.98	12	0	0	0	0	0	0	0	0	0	4	2	3	9
2275	PI4KB_HUMAN	Phosphatidylinositol 4-kinase beta	S428	100%	1.00	22	0	0	0	0	0	0	2	0	1	0	0	0	3
2276	PI4KB_HUMAN	Phosphatidylinositol 4-kinase beta	S511	100%	1.00	67	2	3	2	2	1	3	3	4	4	3	0	3	30
2277	PI42C_HUMAN	Phosphatidylinositol-5-phosphate 4-kinase type-2 gamma	S5	12%	0.13	1	0	0	0	0	0	0	1	0	0	0	0	0	1

2278	AGM1_HUMAN	Phosphoacetylglucosamine mutase	S64	100%	1.00	2	2	0	0	0	0	0	0	0	0	0	0	0	2
2279	AGM1_HUMAN	Phosphoacetylglucosamine mutase	T62	42%	0.42	2	0	0	0	1	0	0	0	0	1	0	0	0	2
2280	PACS1_HUMAN	Phosphofurin acidic cluster sorting protein 1	S804	62%	0.62	1	0	0	0	0	0	0	0	0	0	0	1	0	1
2281	PACS1_HUMAN	Phosphofurin acidic cluster sorting protein 1	S811	62%	0.62	1	0	0	0	0	0	0	0	0	0	0	1	0	1
2282	PACS1_HUMAN	Phosphofurin acidic cluster sorting protein 1	S814	30%	0.30	1	0	0	0	0	0	0	0	0	0	0	1	0	1
2283	PACS1_HUMAN	Phosphofurin acidic cluster sorting protein 1	T793	35%	0.35	1	0	0	0	0	0	0	0	0	0	0	1	0	1
2284	PACS1_HUMAN	Phosphofurin acidic cluster sorting protein 1	Y826	61%	0.61	1	0	0	0	0	0	0	0	0	0	0	1	0	1
2285	PGM1_HUMAN	Phosphoglucomutase-1	S117	100%	1.00	996	16	24	15	14	14	14	16	13	22	18	14	11	191
2286	PGM1_HUMAN	Phosphoglucomutase-1	T115	100%	1.00	243	3	4	5	2	4	1	6	3	2	1	2	1	34
2287	PGM2_HUMAN	Phosphoglucomutase-2	S165	100%	1.00	1	0	0	0	0	0	0	0	0	0	0	1	0	1
2288	PGK1_HUMAN	Phosphoglycerate kinase 1	S203	100%	1.00	786	13	16	12	13	12	14	13	12	12	13	20	18	168
2289	DDHD1_HUMAN	Phospholipase DDHD1	T661	85%	0.85	1	0	0	0	0	0	0	0	0	1	0	0	0	1
2290	KPRB_HUMAN	Phosphoribosyl pyrophosphate synthase-associated protein 2	S219	99%	0.99	13	1	0	0	0	0	1	0	1	1	0	0	1	5
2291	KPRB_HUMAN	Phosphoribosyl pyrophosphate synthase-associated protein 2	S227	100%	1.00	829	18	18	15	14	11	15	14	12	13	18	16	12	176
2292	KPB2_HUMAN	Phosphorylase b kinase regulatory subunit alpha, liver isoform	S1015	100%	1.00	3	2	0	0	0	0	0	1	0	0	0	0	0	3
2293	KPBB_HUMAN	Phosphorylase b kinase regulatory subunit beta	S583	91%	0.91	1	0	0	0	0	0	0	0	1	0	0	0	0	1
2294	PHAX_HUMAN	Phosphorylated adapter RNA export protein	S14	100%	1.00	27	2	0	1	0	2	0	0	0	1	0	3	0	9

2295	PHAX_HUMAN	Phosphorylated adapter RNA export protein	S16	99%	0.99	21	2	0	0	0	2	0	0	0	2	0	1	0	7
2296	PHAX_HUMAN	Phosphorylated adapter RNA export protein	S18	71%	0.71	10	0	0	1	0	0	0	0	0	1	0	2	0	4
2297	PHTNS_HUMAN	Phostensin	S368	100%	1.00	10	0	0	0	0	0	0	0	0	0	1	1	0	2
2298	PHTNS_HUMAN	Phostensin	S432	67%	0.67	4	0	0	0	0	0	0	0	0	0	1	0	1	2
2299	PHTNS_HUMAN	Phostensin	T439	43%	0.43	1	0	0	0	0	0	0	0	0	0	0	0	1	1
2300	PININ_HUMAN	Pinin	S381	100%	1.00	70	1	0	1	3	2	1	1	1	1	5	3	4	23
2301	PININ_HUMAN	Pinin	S443	33%	0.33	13	2	0	1	0	0	0	0	0	0	0	0	0	3
2302	PKP2_HUMAN	Plakophilin-2	S329	100%	1.00	22	0	0	0	0	0	0	0	0	0	2	5	4	11
2303	PKP3_HUMAN	Plakophilin-3	S238	100%	1.00	36	1	4	0	1	0	0	0	0	2	7	5	5	25
2304	PKP3_HUMAN	Plakophilin-3	S240	33%	0.33	4	1	0	0	0	0	0	0	0	0	1	0	0	2
2305	PKP3_HUMAN	Plakophilin-3	S313	100%	1.00	244	1	5	3	6	4	5	2	3	3	8	7	6	53
2306	PKP3_HUMAN	Plakophilin-3	S314	92%	0.92	219	4	1	3	6	5	5	2	3	3	7	8	3	50
2307	PKP3_HUMAN	Plakophilin-3	T235	17%	0.17	2	0	0	0	0	0	0	0	0	0	0	1	1	2
2308	PKP4_HUMAN	Plakophilin-4	S314	100%	1.00	2	0	0	0	0	0	1	0	0	0	0	0	0	1
2309	AT2B1_HUMAN	Plasma membrane calcium-transporting ATPase 1	S1193	100%	1.00	2	1	0	0	0	0	0	0	0	1	0	0	0	2
2310	AT2B3_HUMAN	Plasma membrane calcium-transporting ATPase 3	T68	25%	0.25	1	0	0	0	0	0	0	0	0	0	0	0	1	1
2311	PAIRB_HUMAN	Plasminogen activator inhibitor 1 RNA-binding protein	S234	45%	0.45	2	0	0	0	0	0	0	0	0	0	0	1	0	1
2312	PAIRB_HUMAN	Plasminogen activator inhibitor 1 RNA-binding protein	S25	100%	1.00	25	0	0	4	6	2	0	0	1	0	0	2	0	15

2313	PAIRB_HUMAN	Plasminogen activator inhibitor 1 RNA-binding protein	S392	74%	0.74	2	0	0	0	0	0	0	0	0	0	1	0	0	1
2314	PLSL_HUMAN	Plastin-2	S257	100%	1.00	4	2	1	0	0	0	0	0	0	0	0	0	0	3
2315	PLSL_HUMAN	Plastin-2	S5	100%	1.00	359	8	15	11	14	15	15	17	16	16	0	0	0	127
2316	GI24_HUMAN	Platelet receptor Gi24	S305	100%	1.00	11	0	0	0	0	0	1	1	1	0	1	4	1	9
2317	PKHG3_HUMAN	Pleckstrin homology domain-containing family G member 3	S1037	98%	0.98	1	0	0	0	0	0	0	0	0	0	0	0	1	1
2318	PKHG3_HUMAN	Pleckstrin homology domain-containing family G member 3	S1040	97%	0.97	1	0	0	0	0	0	0	0	0	0	0	0	1	1
2319	PKHG3_HUMAN	Pleckstrin homology domain-containing family G member 3	S1169	83%	0.83	1	0	0	0	1	0	0	0	0	0	0	0	0	1
2320	PKHG3_HUMAN	Pleckstrin homology domain-containing family G member 3	S576	100%	1.00	23	0	0	0	3	3	0	0	0	1	1	1	1	10
2321	PKHG3_HUMAN	Pleckstrin homology domain-containing family G member 3	S577	100%	1.00	23	0	0	0	3	3	0	0	0	1	1	1	1	10
2322	PKHH2_HUMAN	Pleckstrin homology domain-containing family H member 2	S365	100%	1.00	3	1	0	0	0	0	1	0	0	0	0	0	0	2
2323	PHLB2_HUMAN	Pleckstrin homology-like domain family B member 2	S157	100%	1.00	2	0	0	1	0	0	0	0	0	0	0	0	1	2
2324	PHLB2_HUMAN	Pleckstrin homology-like domain family B member 2	S489	100%	1.00	181	4	4	4	5	6	5	4	4	1	3	4	0	44
2325	PHLB2_HUMAN	Pleckstrin homology-like domain family B member 2	S493	83%	0.83	81	2	3	2	4	2	2	1	1	2	1	2	2	24
2326	PHLB2_HUMAN	Pleckstrin homology-like domain family B member 2	S513	60%	0.60	2	0	0	0	1	0	0	0	0	0	1	0	0	2
2327	PHLB2_HUMAN	Pleckstrin homology-like domain family B member 2	T550	100%	1.00	1	0	0	0	0	0	0	0	0	0	0	0	1	1
2328	PLEC_HUMAN	Plectin	S125	100%	1.00	20	0	1	0	0	0	0	1	0	1	3	2	3	11
2329	PLEC_HUMAN	Plectin	S1721	100%	1.00	7	0	0	0	0	0	0	0	0	0	1	3	3	7

2330	PLEC_HUMAN	Plectin	S4382	100%	1.00	103	5	6	3	2	3	4	7	6	6	14	9	3	68
2331	PLEC_HUMAN	Plectin	S4406	76%	0.76	19	0	0	0	0	0	0	0	0	0	1	3	3	7
2332	PLEC_HUMAN	Plectin	S4408	14%	0.14	8	0	0	0	0	0	0	0	0	0	3	1	0	4
2333	PLEC_HUMAN	Plectin	T3785	100%	1.00	1	0	0	0	0	0	0	0	0	0	1	0	0	1
2334	PLEC_HUMAN	Plectin	T4030	100%	1.00	20	0	0	0	0	0	0	0	0	0	2	6	2	10
2335	PLEC_HUMAN	Plectin	T4411	13%	0.13	1	0	0	0	0	0	0	0	0	0	1	0	0	1
2336	PLXC1_HUMAN	Plexin-C1	S368	68%	0.68	1	0	0	0	1	0	0	0	0	0	0	0	0	1
2337	PLXC1_HUMAN	Plexin-C1	S369	68%	0.68	1	0	0	0	1	0	0	0	0	0	0	0	0	1
2338	PLXC1_HUMAN	Plexin-C1	S374	74%	0.74	1	0	0	0	1	0	0	0	0	0	0	0	0	1
2339	PLXC1_HUMAN	Plexin-C1	S378	44%	0.44	1	0	0	0	1	0	0	0	0	0	0	0	0	1
2340	PLXC1_HUMAN	Plexin-C1	T370	68%	0.68	1	0	0	0	1	0	0	0	0	0	0	0	0	1
2341	PLXC1_HUMAN	Plexin-C1	T377	70%	0.70	1	0	0	0	1	0	0	0	0	0	0	0	0	1
2342	POGZ_HUMAN	Pogo transposable element with ZNF domain	S571	42%	0.42	1	0	0	0	0	0	1	0	0	0	0	0	0	1
2343	POGZ_HUMAN	Pogo transposable element with ZNF domain	Y590	86%	0.86	1	0	0	0	0	0	1	0	0	0	0	0	0	1
2344	PARP1_HUMAN	Poly [ADP-ribose] polymerase 1	S776	42%	0.42	5	0	0	0	0	0	2	0	0	0	0	0	0	2
2345	PARP1_HUMAN	Poly [ADP-ribose] polymerase 1	S782	94%	0.94	5	0	0	0	0	0	2	0	0	0	0	0	0	2
2346	PCBP1_HUMAN	Poly(rC)-binding protein 1	S171	33%	0.33	2	0	0	0	0	0	0	0	0	0	0	1	1	2
2347	PCBP1_HUMAN	Poly(rC)-binding protein 1	S173	100%	1.00	74	0	0	0	0	0	0	0	0	0	0	6	9	15
2348	PCBP1_HUMAN	Poly(rC)-binding protein 1	S189	85%	0.85	8	1	0	0	0	0	0	0	0	1	0	0	0	2
2349	PCBP1_HUMAN	Poly(rC)-binding protein 1	S190	100%	1.00	268	7	10	7	5	7	4	5	7	6	11	15	9	93

2350	PCBP1_HUMAN	Poly(rC)-binding protein 1	S262	20%	0.20	2	1	0	0	0	0	0	1	0	0	0	0	0	2
2351	PCBP1_HUMAN	Poly(rC)-binding protein 1	S263	5%	0.05	1	0	1	0	0	0	0	0	0	0	0	0	0	1
2352	PCBP1_HUMAN	Poly(rC)-binding protein 1	S264	89%	0.89	3	2	0	0	0	0	0	1	0	0	0	0	0	3
2353	PCBP4_HUMAN	Poly(rC)-binding protein 4	S68	59%	0.59	1	0	0	1	0	0	0	0	0	0	0	0	0	1
2354	PCBP4_HUMAN	Poly(rC)-binding protein 4	S77	48%	0.48	1	0	0	1	0	0	0	0	0	0	0	0	0	1
2355	PCBP4_HUMAN	Poly(rC)-binding protein 4	T69	48%	0.48	1	0	0	1	0	0	0	0	0	0	0	0	0	1
2356	PABP2_HUMAN	Polyadenylate-binding protein 2	S150	100%	1.00	5	0	0	0	0	0	0	0	0	0	0	0	2	2
2357	PABP2_HUMAN	Polyadenylate-binding protein 2	S19	100%	1.00	6	0	1	0	1	0	0	0	0	0	0	1	0	3
2358	PABP2_HUMAN	Polyadenylate-binding protein 2	S95	66%	0.66	3	0	0	0	0	0	0	0	0	0	0	2	0	2
2359	PKDRE_HUMAN	Polycystic kidney disease and receptor for egg jelly-related protein	S1426	71%	0.71	1	0	1	0	0	0	0	0	0	0	0	0	0	1
2360	PKDRE_HUMAN	Polycystic kidney disease and receptor for egg jelly-related protein	S1433	29%	0.29	1	0	1	0	0	0	0	0	0	0	0	0	0	1
2361	PKDRE_HUMAN	Polycystic kidney disease and receptor for egg jelly-related protein	T1437	29%	0.29	1	0	1	0	0	0	0	0	0	0	0	0	0	1
2362	PKDRE_HUMAN	Polycystic kidney disease and receptor for egg jelly-related protein	Y1423	85%	0.85	1	0	1	0	0	0	0	0	0	0	0	0	0	1
2363	PK1L1_HUMAN	Polycystic kidney disease protein 1-like 1	S1450	61%	0.61	1	0	0	0	0	0	0	0	0	0	0	1	0	1
2364	PK1L1_HUMAN	Polycystic kidney disease protein 1-like 1	S1458	94%	0.94	1	0	0	0	0	0	0	0	0	0	0	1	0	1
2365	PK1L1_HUMAN	Polycystic kidney disease protein 1-like 1	T1447	61%	0.61	1	0	0	0	0	0	0	0	0	0	0	1	0	1
2366	PK1L1_HUMAN	Polycystic kidney disease protein 1-like 1	T1464	61%	0.61	1	0	0	0	0	0	0	0	0	0	0	1	0	1

2367	PKD1_HUMAN	Polycystin-1	S4251	93%	0.93	1	0	0	0	0	0	0	0	0	0	1	0	0	1
2368	PKD1_HUMAN	Polycystin-1	S4252	95%	0.95	1	0	0	0	0	0	0	0	0	0	1	0	0	1
2369	PKD1_HUMAN	Polycystin-1	S4259	93%	0.93	1	0	0	0	0	0	0	0	0	0	1	0	0	1
2370	PHC3_HUMAN	Polyhomeotic-like protein 3	T609	100%	1.00	1	0	0	0	0	0	0	0	1	0	0	0	0	1
2371	PTRF_HUMAN	Polymerase I and transcript release factor	S202	100%	1.00	245	6	6	5	9	7	9	8	4	7	4	5	2	72
2372	PTRF_HUMAN	Polymerase I and transcript release factor	S203	100%	1.00	246	6	6	5	9	7	9	8	4	7	4	5	2	72
2373	PTRF_HUMAN	Polymerase I and transcript release factor	T302	88%	0.88	3	0	1	0	0	0	0	0	0	0	0	0	0	1
2374	PIGR_HUMAN	Polymeric immunoglobulin receptor	S624	100%	1.00	2	0	0	0	0	0	0	1	0	0	0	0	0	1
2375	PIGR_HUMAN	Polymeric immunoglobulin receptor	S627	100%	1.00	2	0	0	0	0	0	0	1	0	0	0	0	0	1
2376	PIGR_HUMAN	Polymeric immunoglobulin receptor	S629	99%	0.99	2	0	0	0	0	0	0	1	0	0	0	0	0	1
2377	PIGR_HUMAN	Polymeric immunoglobulin receptor	S630	99%	0.99	2	0	0	0	0	0	0	1	0	0	0	0	0	1
2378	PIGR_HUMAN	Polymeric immunoglobulin receptor	S636	94%	0.94	2	0	0	0	0	0	0	1	0	0	0	0	0	1
2379	PIGR_HUMAN	Polymeric immunoglobulin receptor	S637	96%	0.96	2	0	0	0	0	0	0	1	0	0	0	0	0	1
2380	PIGR_HUMAN	Polymeric immunoglobulin receptor	T643	58%	0.58	2	0	0	0	0	0	0	1	0	0	0	0	0	1
2381	PTBP1_HUMAN	Polypyrimidine tract-binding protein 1	S140	29%	0.29	1	0	0	0	0	0	0	0	0	0	0	0	1	1
2382	PTBP1_HUMAN	Polypyrimidine tract-binding protein 1	S141	29%	0.29	1	0	0	0	0	0	0	0	0	0	0	0	1	1
2383	PNPT1_HUMAN	Polyribonucleotide nucleotidyltransferase 1, mitochondrial	S474	41%	0.41	1	0	0	0	1	0	0	0	0	0	0	0	0	1
2384	PNPT1_HUMAN	Polyribonucleotide nucleotidyltransferase 1, mitochondrial	S479	39%	0.39	1	0	0	0	1	0	0	0	0	0	0	0	0	1

2385	PNPT1_HUMAN	Polyribonucleotide nucleotidyltransferase 1, mitochondrial	S504	41%	0.41	1	0	0	0	1	0	0	0	0	0	0	0	0	1
2386	PNPT1_HUMAN	Polyribonucleotide nucleotidyltransferase 1, mitochondrial	T473	39%	0.39	1	0	0	0	1	0	0	0	0	0	0	0	0	1
2387	PNPT1_HUMAN	Polyribonucleotide nucleotidyltransferase 1, mitochondrial	T515	51%	0.51	1	0	0	0	1	0	0	0	0	0	0	0	0	1
2388	PO4F2_HUMAN	POU domain, class 4, transcription factor 2	S76	25%	0.25	1	0	1	0	0	0	0	0	0	0	0	0	0	1
2389	PO4F2_HUMAN	POU domain, class 4, transcription factor 2	S78	25%	0.25	1	0	1	0	0	0	0	0	0	0	0	0	0	1
2390	PO4F2_HUMAN	POU domain, class 4, transcription factor 2	S79	46%	0.46	1	0	1	0	0	0	0	0	0	0	0	0	0	1
2391	PRD11_HUMAN	PR domain-containing protein 11	T46	92%	0.92	41	0	1	1	1	0	0	2	2	1	0	0	0	8
2392	PBX2_HUMAN	Pre-B-cell leukemia transcription factor 2	S146	73%	0.73	1	0	0	0	0	0	0	0	1	0	0	0	0	1
2393	PBX2_HUMAN	Pre-B-cell leukemia transcription factor 2	S151	30%	0.30	1	0	0	0	0	0	0	0	1	0	0	0	0	1
2394	PBX2_HUMAN	Pre-B-cell leukemia transcription factor 2	S155	49%	0.49	1	0	0	0	0	0	0	0	1	0	0	0	0	1
2395	PBX2_HUMAN	Pre-B-cell leukemia transcription factor 2	S159	82%	0.82	1	0	0	0	0	0	0	0	1	0	0	0	0	1
2396	LMNA_HUMAN	Prelamin-A/C	S301	100%	1.00	12	0	0	0	0	0	0	0	0	0	1	1	0	2
2397	LMNA_HUMAN	Prelamin-A/C	S307	5%	0.05	1	0	0	0	0	0	0	0	0	0	0	0	1	1
2398	LMNA_HUMAN	Prelamin-A/C	S390	100%	1.00	19	0	0	0	0	0	0	1	0	0	4	2	3	10
2399	LMNA_HUMAN	Prelamin-A/C	S392	14%	0.14	1	0	0	0	0	0	0	0	0	0	0	0	1	1
2400	LMNA_HUMAN	Prelamin-A/C	S613	15%	0.15	8	0	0	0	0	0	0	0	0	0	2	0	0	2

2401	LMNA_HUMAN	Prelamin-A/C	S616	50%	0.50	8	0	0	0	0	0	0	0	0	0	1	3	0	4
2402	LMNA_HUMAN	Prelamin-A/C	S628	99%	0.99	37	0	0	0	1	0	0	0	0	0	5	3	5	14
2403	LMNA_HUMAN	Prelamin-A/C	S632	79%	0.79	31	0	0	0	1	0	0	0	0	0	1	3	1	6
2404	FIP1_HUMAN	Pre-mRNA 3'-end-processing factor FIP1	S492	100%	1.00	73	1	3	0	0	3	2	5	2	4	2	0	0	22
2405	FIP1_HUMAN	Pre-mRNA 3'-end-processing factor FIP1	T494	68%	0.68	34	2	1	3	3	1	2	1	2	2	1	2	1	21
2406	PR38A_HUMAN	Pre-mRNA-splicing factor 38A	S193	100%	1.00	26	1	2	1	3	2	0	1	0	0	1	3	0	14
2407	PR38A_HUMAN	Pre-mRNA-splicing factor 38A	S194	100%	1.00	26	1	2	1	3	2	0	1	0	0	1	3	0	14
2408	PRIC2_HUMAN	Prickle-like protein 2	S414	68%	0.68	1	0	0	0	0	0	0	0	0	0	1	0	0	1
2409	PRIC2_HUMAN	Prickle-like protein 2	S420	68%	0.68	1	0	0	0	0	0	0	0	0	0	1	0	0	1
2410	PRIC2_HUMAN	Prickle-like protein 2	T412	68%	0.68	1	0	0	0	0	0	0	0	0	0	1	0	0	1
2411	PRIC2_HUMAN	Prickle-like protein 2	Y428	49%	0.49	1	0	0	0	0	0	0	0	0	0	1	0	0	1
2412	ALKB5_HUMAN	Probable alpha-ketoglutarate-dependent dioxygenase ABH5	S361	90%	0.90	1	1	0	0	0	0	0	0	0	0	0	0	0	1
2413	HFM1_HUMAN	Probable ATP-dependent DNA helicase HFM1	S179	52%	0.52	1	0	0	0	0	0	0	0	0	0	0	1	0	1
2414	HFM1_HUMAN	Probable ATP-dependent DNA helicase HFM1	Y178	70%	0.70	1	0	0	0	0	0	0	0	0	0	0	1	0	1
2415	DDX27_HUMAN	Probable ATP-dependent RNA helicase DDX27	S166	97%	0.97	1	0	0	0	0	0	0	0	0	0	0	0	1	1
2416	DDX27_HUMAN	Probable ATP-dependent RNA helicase DDX27	S171	58%	0.58	1	0	0	0	0	0	0	0	0	0	0	0	1	1
2417	DDX27_HUMAN	Probable ATP-dependent RNA helicase DDX27	S176	64%	0.64	1	0	0	0	0	0	0	0	0	0	0	0	1	1
2418	DDX27_HUMAN	Probable ATP-dependent RNA helicase DDX27	S177	60%	0.60	1	0	0	0	0	0	0	0	0	0	0	0	1	1

2419	DDX27_HUMAN	Probable ATP-dependent RNA helicase DDX27	T173	50%	0.50	1	0	0	0	0	0	0	0	0	0	0	0	1	1
2420	DDX27_HUMAN	Probable ATP-dependent RNA helicase DDX27	T534	100%	1.00	1	0	0	1	0	0	0	0	0	0	0	0	0	1
2421	DDX27_HUMAN	Probable ATP-dependent RNA helicase DDX27	T539	100%	1.00	1	0	0	1	0	0	0	0	0	0	0	0	0	1
2422	DDX27_HUMAN	Probable ATP-dependent RNA helicase DDX27	T543	100%	1.00	1	0	0	1	0	0	0	0	0	0	0	0	0	1
2423	DDX46_HUMAN	Probable ATP-dependent RNA helicase DDX46	S295	82%	0.82	1	0	0	0	0	0	0	0	0	1	0	0	0	1
2424	DDX46_HUMAN	Probable ATP-dependent RNA helicase DDX46	S296	72%	0.72	1	0	0	0	0	0	0	0	0	1	0	0	0	1
2425	DDX46_HUMAN	Probable ATP-dependent RNA helicase DDX46	S804	100%	1.00	####	34	19	23	27	29	23	21	19	19	19	20	13	266
2426	DDX46_HUMAN	Probable ATP-dependent RNA helicase DDX46	T305	65%	0.65	1	0	0	0	0	0	0	0	0	1	0	0	0	1
2427	DDX46_HUMAN	Probable ATP-dependent RNA helicase DDX46	T312	74%	0.74	1	0	0	0	0	0	0	0	0	1	0	0	0	1
2428	DDX46_HUMAN	Probable ATP-dependent RNA helicase DDX46	Y294	60%	0.60	1	0	0	0	0	0	0	0	0	1	0	0	0	1
2429	DDX47_HUMAN	Probable ATP-dependent RNA helicase DDX47	S14	16%	0.16	1	0	0	0	0	0	0	0	1	0	0	0	0	1
2430	DDX47_HUMAN	Probable ATP-dependent RNA helicase DDX47	S300	66%	0.66	3	0	1	0	0	0	1	0	1	0	0	0	0	3
2431	DDX47_HUMAN	Probable ATP-dependent RNA helicase DDX47	S9	100%	1.00	71	5	4	4	2	1	4	1	1	1	0	1	4	28
2432	DDX47_HUMAN	Probable ATP-dependent RNA helicase DDX47	T11	90%	0.90	6	0	1	0	0	0	0	0	0	0	0	1	0	2
2433	DDX47_HUMAN	Probable ATP-dependent RNA helicase DDX47	T289	51%	0.51	3	0	1	0	0	0	1	0	1	0	0	0	0	3
2434	AT132_HUMAN	Probable cation-transporting ATPase 13A2	S30	16%	0.16	1	0	0	0	1	0	0	0	0	0	0	0	0	1
2435	AT132_HUMAN	Probable cation-transporting ATPase 13A2	S31	16%	0.16	1	0	0	0	1	0	0	0	0	0	0	0	0	1

2436	AT132_HUMAN	Probable cation-transporting ATPase 13A2	S33	16%	0.16	1	0	0	0	1	0	0	0	0	0	0	0	0	1
2437	AT133_HUMAN	Probable cation-transporting ATPase 13A3	S817	100%	1.00	24	0	2	0	0	0	0	0	0	0	8	3	0	13
2438	HERC1_HUMAN	Probable E3 ubiquitin-protein ligase HERC1	S1428	60%	0.60	14	0	1	0	0	1	1	0	0	1	0	0	0	4
2439	HERC1_HUMAN	Probable E3 ubiquitin-protein ligase HERC1	T1429	67%	0.67	5	0	1	0	0	0	0	0	1	0	0	1	0	3
2440	MYCB2_HUMAN	Probable E3 ubiquitin-protein ligase MYCBP2	S1545	86%	0.86	1	0	1	0	0	0	0	0	0	0	0	0	0	1
2441	MYCB2_HUMAN	Probable E3 ubiquitin-protein ligase MYCBP2	S1546	86%	0.86	1	0	1	0	0	0	0	0	0	0	0	0	0	1
2442	MYCB2_HUMAN	Probable E3 ubiquitin-protein ligase MYCBP2	S1547	86%	0.86	1	0	1	0	0	0	0	0	0	0	0	0	0	1
2443	MYCB2_HUMAN	Probable E3 ubiquitin-protein ligase MYCBP2	S1555	86%	0.86	1	0	1	0	0	0	0	0	0	0	0	0	0	1
2444	MYCB2_HUMAN	Probable E3 ubiquitin-protein ligase MYCBP2	S1561	86%	0.86	1	0	1	0	0	0	0	0	0	0	0	0	0	1
2445	MYCB2_HUMAN	Probable E3 ubiquitin-protein ligase MYCBP2	S3467	100%	1.00	26	0	3	0	0	0	2	2	3	5	0	1	1	17
2446	MYCB2_HUMAN	Probable E3 ubiquitin-protein ligase MYCBP2	T1560	86%	0.86	1	0	1	0	0	0	0	0	0	0	0	0	0	1
2447	R144A_HUMAN	Probable E3 ubiquitin-protein ligase RNF144A	T159	51%	0.51	1	0	0	0	0	0	0	0	0	0	0	0	1	1
2448	TRIPC_HUMAN	Probable E3 ubiquitin-protein ligase TRIP12	S310	81%	0.81	2	0	1	0	0	0	0	0	1	0	0	0	0	2
2449	TRIPC_HUMAN	Probable E3 ubiquitin-protein ligase TRIP12	S312	100%	1.00	30	5	1	1	0	5	4	2	1	2	1	0	0	22
2450	TRIPC_HUMAN	Probable E3 ubiquitin-protein ligase TRIP12	S942	100%	1.00	15	0	0	0	1	1	1	0	0	0	0	1	0	4
2451	MYST1_HUMAN	Probable histone acetyltransferase MYST1	S37	77%	0.77	8	3	0	0	0	0	0	0	0	0	0	0	0	3
2452	MYST1_HUMAN	Probable histone acetyltransferase MYST1	S42	65%	0.65	7	3	0	0	0	0	0	1	0	0	0	0	0	4

2453	MYST1_HUMAN	Probable histone acetyltransferase MYST1	T45	46%	0.46	4	1	0	0	0	0	0	1	0	0	0	0	0	2
2454	TARB1_HUMAN	Probable methyltransferase TARBP1	T280	100%	1.00	1	0	0	0	0	0	0	0	0	1	0	0	0	1
2455	RBM46_HUMAN	Probable RNA-binding protein 46	T9	6%	0.06	1	0	0	0	0	0	0	0	1	0	0	0	0	1
2456	HTRA3_HUMAN	Probable serine protease HTRA3	S174	16%	0.16	1	1	0	0	0	0	0	0	0	0	0	0	0	1
2457	HTRA3_HUMAN	Probable serine protease HTRA3	S176	31%	0.31	1	1	0	0	0	0	0	0	0	0	0	0	0	1
2458	HTRA3_HUMAN	Probable serine protease HTRA3	S181	91%	0.91	1	1	0	0	0	0	0	0	0	0	0	0	0	1
2459	USP9X_HUMAN	Probable ubiquitin carboxyl-terminal hydrolase FAF-X	S1600	92%	0.92	1	1	0	0	0	0	0	0	0	0	0	0	0	1
2460	PROF1_HUMAN	Profilin-1	S28	90%	0.90	73	1	1	2	0	2	1	0	1	1	0	2	2	13
2461	PROF1_HUMAN	Profilin-1	S30	60%	0.60	45	0	1	0	0	2	0	0	1	0	0	0	1	5
2462	PROF1_HUMAN	Profilin-1	Y25	92%	0.92	50	1	0	2	0	2	1	0	2	1	0	2	1	12
2463	PDC6I_HUMAN	Programmed cell death 6-interacting protein	S582	74%	0.74	10	1	0	0	0	0	0	1	0	0	0	0	0	2
2464	PDC6I_HUMAN	Programmed cell death 6-interacting protein	T581	69%	0.69	10	1	0	0	0	0	0	1	0	0	0	0	0	2
2465	PDC6I_HUMAN	Programmed cell death 6-interacting protein	T586	56%	0.56	10	1	0	0	0	0	0	1	0	0	0	0	0	2
2466	PA2G4_HUMAN	Proliferation-associated protein 2G4	T11	89%	0.89	32	0	2	1	2	2	0	2	1	0	2	0	0	12
2467	PELP1_HUMAN	Proline-, glutamic acid- and leucine-rich protein 1	S743	82%	0.82	56	0	1	2	1	0	1	1	1	2	1	1	0	11
2468	PELP1_HUMAN	Proline-, glutamic acid- and leucine-rich protein 1	T745	100%	1.00	400	11	13	8	11	13	8	10	9	8	7	7	3	108
2469	PELP1_HUMAN	Proline-, glutamic acid- and leucine-rich protein 1	T749	47%	0.47	15	0	1	0	0	0	0	1	1	0	1	0	0	4
2470	PSRC1_HUMAN	Proline/serine-rich coiled-coil protein 1	S70	100%	1.00	17	0	0	0	0	0	0	0	0	0	0	2	3	5

2471	AKTS1_HUMAN	Proline-rich AKT1 substrate 1	S183	100%	1.00	15	4	8	0	0	0	0	0	0	0	0	0	0	12
2472	PRR5L_HUMAN	Proline-rich protein 5-like	S255	90%	0.90	1	0	0	0	0	0	0	0	1	0	0	0	0	1
2473	PRR5L_HUMAN	Proline-rich protein 5-like	S261	73%	0.73	1	0	0	0	0	1	0	0	0	0	0	0	0	1
2474	PRR5L_HUMAN	Proline-rich protein 5-like	T249	95%	0.95	2	0	0	0	0	1	0	0	1	0	0	0	0	2
2475	PRR5L_HUMAN	Proline-rich protein 5-like	T258	25%	0.25	2	0	0	0	0	1	0	0	1	0	0	0	0	2
2476	PRR5L_HUMAN	Proline-rich protein 5-like	T270	74%	0.74	2	0	0	0	0	1	0	0	1	0	0	0	0	2
2477	PRR5L_HUMAN	Proline-rich protein 5-like	T273	98%	0.98	2	0	0	0	0	1	0	0	1	0	0	0	0	2
2478	PRCC_HUMAN	Proline-rich protein PRCC	S157	100%	1.00	34	0	1	2	1	1	1	1	3	1	1	1	1	14
2479	PRCC_HUMAN	Proline-rich protein PRCC	S159	100%	1.00	33	0	1	2	1	1	1	1	3	1	1	1	1	14
2480	PRCC_HUMAN	Proline-rich protein PRCC	S267	100%	1.00	7	0	0	1	0	0	0	0	0	0	0	0	0	1
2481	PRCC_HUMAN	Proline-rich protein PRCC	T261	33%	0.33	3	0	0	0	0	0	0	0	0	1	0	0	0	1
2482	LRP1_HUMAN	Prolow-density lipoprotein receptor-related protein 1	S2480	71%	0.71	1	0	0	0	0	0	0	0	0	1	0	0	0	1
2483	NPY_HUMAN	Pro-neuropeptide Y	T72	60%	0.60	1	0	0	0	0	1	0	0	0	0	0	0	0	1
2484	TEBP_HUMAN	Prostaglandin E synthase 3	S113	100%	1.00	297	15	14	7	5	10	12	15	10	10	8	12	6	124
2485	TEBP_HUMAN	Prostaglandin E synthase 3	S148	100%	1.00	434	11	17	8	8	10	10	12	14	11	12	13	6	132
2486	TEBP_HUMAN	Prostaglandin E synthase 3	S151	100%	1.00	435	11	17	8	8	10	11	12	14	11	12	13	6	133
2487	PF2R_HUMAN	Prostaglandin F2-alpha receptor	T294	100%	1.00	1	0	0	0	1	0	0	0	0	0	0	0	0	1
2488	PGH2_HUMAN	Prostaglandin G/H synthase 2	S105	21%	0.21	1	0	0	0	0	0	0	1	0	0	0	0	0	1
2489	PGH2_HUMAN	Prostaglandin G/H synthase 2	T104	71%	0.71	1	0	0	0	0	0	0	1	0	0	0	0	0	1
2490	PGH2_HUMAN	Prostaglandin G/H synthase 2	Y116	71%	0.71	1	0	0	0	0	0	0	1	0	0	0	0	0	1

2491	PGH2_HUMAN	Prostaglandin G/H synthase 2	Y120	43%	0.43	1	0	0	0	0	0	0	1	0	0	0	0	0	1
2492	PGH2_HUMAN	Prostaglandin G/H synthase 2	Y122	21%	0.21	1	0	0	0	0	0	0	1	0	0	0	0	0	1
2493	PSME3_HUMAN	Proteasome activator complex subunit 3	S24	100%	1.00	295	5	5	4	9	9	4	4	4	6	8	9	7	74
2494	PSME3_HUMAN	Proteasome activator complex subunit 3	T23	98%	0.98	15	1	1	1	0	0	0	0	0	0	2	0	2	7
2495	PSMF1_HUMAN	Proteasome inhibitor PI31 subunit	S153	100%	1.00	2	0	0	0	0	0	0	0	0	0	1	0	1	2
2496	PSMF1_HUMAN	Proteasome inhibitor PI31 subunit	S252	100%	1.00	12	6	3	1	2	0	0	0	0	0	0	0	0	12
2497	PSA3_HUMAN	Proteasome subunit alpha type-3	S250	100%	1.00	6	0	0	0	0	2	0	0	0	3	0	1	0	6
2498	PSA5_HUMAN	Proteasome subunit alpha type-5	S56	100%	1.00	41	0	0	0	0	0	0	0	0	0	4	5	4	13
2499	PSB4_HUMAN	Proteasome subunit beta type-4	S77	56%	0.56	1	0	0	0	0	0	1	0	0	0	0	0	0	1
2500	PSB4_HUMAN	Proteasome subunit beta type-4	T46	45%	0.45	1	0	0	0	0	0	1	0	0	0	0	0	0	1
2501	AATF_HUMAN	Protein AATF	S203	100%	1.00	33	1	2	5	2	1	1	0	1	2	1	0	0	16
2502	AHNK2_HUMAN	Protein AHNAK2	S1350	44%	0.44	1	0	1	0	0	0	0	0	0	0	0	0	0	1
2503	AHNK2_HUMAN	Protein AHNAK2	S1354	32%	0.32	1	0	1	0	0	0	0	0	0	0	0	0	0	1
2504	AHNK2_HUMAN	Protein AHNAK2	S1363	93%	0.93	1	0	1	0	0	0	0	0	0	0	0	0	0	1
2505	AHNK2_HUMAN	Protein AHNAK2	S294	100%	1.00	201	1	0	1	6	8	2	7	5	3	8	5	7	53
2506	AHNK2_HUMAN	Protein AHNAK2	S297	17%	0.17	4	0	0	0	0	0	0	0	0	0	1	0	1	2
2507	AHNK2_HUMAN	Protein AHNAK2	S4419	90%	0.90	94	0	0	0	0	0	0	0	0	0	2	1	1	4
2508	AHNK2_HUMAN	Protein AHNAK2	S4477	70%	0.70	1	0	0	0	0	0	0	0	0	0	1	0	0	1
2509	AHNK2_HUMAN	Protein AHNAK2	S4894	100%	1.00	3	0	0	0	0	0	0	0	0	0	0	1	0	1
2510	AHNK2_HUMAN	Protein AHNAK2	S4897	100%	1.00	12	0	0	0	0	0	0	0	0	0	4	2	0	6

2511	AHNK2_HUMAN	Protein AHNAK2	S5238	51%	0.51	1	0	1	0	0	0	0	0	0	0	0	0	0	1
2512	AHNK2_HUMAN	Protein AHNAK2	S5240	86%	0.86	1	0	1	0	0	0	0	0	0	0	0	0	0	1
2513	AHNK2_HUMAN	Protein AHNAK2	S5260	72%	0.72	1	0	1	0	0	0	0	0	0	0	0	0	0	1
2514	AHNK2_HUMAN	Protein AHNAK2	S5262	70%	0.70	1	0	1	0	0	0	0	0	0	0	0	0	0	1
2515	AHNK2_HUMAN	Protein AHNAK2	S595	63%	0.63	1	0	0	0	0	0	0	0	0	0	0	1	0	1
2516	AHNK2_HUMAN	Protein AHNAK2	S842	100%	1.00	22	0	0	0	0	0	0	0	2	0	0	0	2	4
2517	AHNK2_HUMAN	Protein AHNAK2	T296	17%	0.17	12	0	0	0	0	0	1	0	0	1	0	1	0	3
2518	JUB_HUMAN	Protein ajuba	S230	92%	0.92	9	0	1	0	1	0	2	0	0	0	0	0	0	4
2519	AMCL3_HUMAN	Protein AMAC1L3	S24	81%	0.81	1	0	0	0	0	0	0	0	0	0	0	1	0	1
2520	AMCL3_HUMAN	Protein AMAC1L3	S4	29%	0.29	1	0	0	0	0	0	0	0	0	0	0	1	0	1
2521	ANM1_HUMAN	Protein arginine N-methyltransferase 1	S13	29%	0.29	115	5	4	4	2	4	2	3	2	6	1	0	2	35
2522	ANM1_HUMAN	Protein arginine N-methyltransferase 1	T7	73%	0.73	104	5	3	4	2	3	2	5	6	4	0	0	0	34
2523	ANM3_HUMAN	Protein arginine N-methyltransferase 3	S25	100%	1.00	29	0	1	0	0	2	0	2	0	0	0	1	0	6
2524	ANM3_HUMAN	Protein arginine N-methyltransferase 3	S27	100%	1.00	29	0	1	0	0	2	0	2	0	0	0	1	0	6
2525	AGO1_HUMAN	Protein argonaute-1	S621	28%	0.28	1	0	0	0	0	0	0	0	0	0	0	0	1	1
2526	AGO1_HUMAN	Protein argonaute-1	T597	61%	0.61	1	0	0	0	0	0	0	0	0	0	0	0	1	1
2527	BICD2_HUMAN	Protein bicaudal D homolog 2	S329	99%	0.99	61	0	0	0	0	0	0	0	0	0	4	3	3	10
2528	BICD2_HUMAN	Protein bicaudal D homolog 2	S331	83%	0.83	56	0	0	0	0	3	0	0	0	0	3	5	1	12
2529	BICD2_HUMAN	Protein bicaudal D homolog 2	S334	59%	0.59	65	0	0	0	0	0	1	0	0	1	3	4	2	11

2530	CIC_HUMAN	Protein capicua homolog	S1373	92%	0.92	70	1	0	1	3	1	1	0	2	1	3	1	0	14
2531	CIC_HUMAN	Protein capicua homolog	S1382	100%	1.00	90	2	0	2	3	2	1	2	3	3	3	3	2	26
2532	CIC_HUMAN	Protein capicua homolog	S299	49%	0.49	9	0	0	0	2	0	0	0	0	0	1	0	0	3
2533	CIC_HUMAN	Protein capicua homolog	S301	50%	0.50	13	0	0	0	0	0	0	1	0	0	2	0	0	3
2534	CIC_HUMAN	Protein capicua homolog	S313	7%	0.07	1	0	0	0	0	0	1	0	0	0	0	0	0	1
2535	CIC_HUMAN	Protein capicua homolog	T1375	85%	0.85	20	1	0	1	0	1	0	2	1	2	0	2	2	12
2536	CIC_HUMAN	Protein capicua homolog	T303	13%	0.13	6	0	0	0	0	0	0	0	1	0	1	0	0	2
2537	CIC_HUMAN	Protein capicua homolog	T305	41%	0.41	18	2	0	0	0	1	0	1	0	0	0	0	0	4
2538	CASC3_HUMAN	Protein CASC3	S18	72%	0.72	1	0	0	0	1	0	0	0	0	0	0	0	0	1
2539	MTG8_HUMAN	Protein CBFA2T1	S277	35%	0.35	2	0	0	0	0	1	0	0	0	0	0	0	0	1
2540	MTG8_HUMAN	Protein CBFA2T1	S283	94%	0.94	2	0	0	0	0	1	0	0	0	0	0	0	0	1
2541	MTG8_HUMAN	Protein CBFA2T1	T293	84%	0.84	2	0	0	0	0	1	0	0	0	0	0	0	0	1
2542	MTG8_HUMAN	Protein CBFA2T1	Y300	84%	0.84	2	0	0	0	0	1	0	0	0	0	0	0	0	1
2543	CDV3_HUMAN	Protein CDV3 homolog	S30	20%	0.20	6	2	0	0	0	0	0	0	0	0	0	1	1	4
2544	CWC15_HUMAN	Protein CWC15 homolog	S121	100%	1.00	27	0	1	0	0	0	0	1	1	0	0	0	0	3
2545	CWC15_HUMAN	Protein CWC15 homolog	T110	100%	1.00	31	0	1	0	0	0	0	1	1	0	0	0	0	3
2546	DDI2_HUMAN	Protein DDI1 homolog 2	S106	100%	1.00	23	0	0	0	0	0	0	0	0	0	2	2	2	6
2547	DDI2_HUMAN	Protein DDI1 homolog 2	S194	100%	1.00	2	0	0	0	1	0	0	0	0	0	0	1	0	2
2548	DEK_HUMAN	Protein DEK	T13	98%	0.98	59	3	0	0	1	2	1	0	0	2	2	0	1	12
2549	DEK_HUMAN	Protein DEK	T15	96%	0.96	21	1	0	0	0	0	1	0	0	0	0	0	0	2

2550	DTX4_HUMAN	Protein deltex-4	T410	100%	1.00	1	0	0	0	0	0	0	1	0	0	0	0	0	1
2551	DIAP3_HUMAN	Protein diaphanous homolog 3	S624	100%	1.00	1	0	1	0	0	0	0	0	0	0	0	0	0	1
2552	DOS_HUMAN	Protein Dos	T306	46%	0.46	1	0	0	0	0	0	0	0	0	0	1	0	0	1
2553	DOS_HUMAN	Protein Dos	T312	54%	0.54	1	0	0	0	0	0	0	0	0	0	1	0	0	1
2554	EFR3A_HUMAN	Protein EFR3 homolog A	T723	16%	0.16	1	0	0	0	0	0	0	1	0	0	0	0	0	1
2555	EFR3A_HUMAN	Protein EFR3 homolog A	T727	20%	0.20	1	0	0	0	0	0	0	1	0	0	0	0	0	1
2556	ELYS_HUMAN	Protein ELYS	S1232	100%	1.00	14	0	1	0	0	1	0	0	0	0	0	0	0	2
2557	F1142_HUMAN	Protein FAM114A2	S145	63%	0.63	12	0	0	0	0	1	0	0	0	0	0	1	1	3
2558	F1142_HUMAN	Protein FAM114A2	S146	67%	0.67	47	0	0	0	2	0	2	1	0	0	6	1	1	13
2559	F122A_HUMAN	Protein FAM122A	S147	100%	1.00	7	0	1	0	0	0	0	0	0	0	0	0	0	1
2560	F122A_HUMAN	Protein FAM122A	S248	12%	0.12	1	0	0	0	0	0	0	0	0	0	0	0	1	1
2561	F122A_HUMAN	Protein FAM122A	S270	100%	1.00	10	0	0	0	0	2	0	0	0	2	1	1	2	8
2562	F122A_HUMAN	Protein FAM122A	S37	87%	0.87	31	0	0	0	1	1	1	1	1	1	1	2	0	9
2563	F122A_HUMAN	Protein FAM122A	S48	7%	0.07	1	0	0	0	1	0	0	0	0	0	0	0	0	1
2564	F122A_HUMAN	Protein FAM122A	S76	100%	1.00	53	0	1	0	0	0	0	0	0	0	1	4	6	12
2565	F122A_HUMAN	Protein FAM122A	T149	99%	0.99	10	0	0	0	0	0	0	0	0	0	0	1	0	1
2566	F122A_HUMAN	Protein FAM122A	T243	58%	0.58	1	0	0	0	0	0	0	0	0	0	0	0	1	1
2567	F122A_HUMAN	Protein FAM122A	T47	25%	0.25	9	0	0	1	0	1	0	0	0	0	1	0	1	4
2568	F135A_HUMAN	Protein FAM135A	T326	38%	0.38	1	0	0	0	1	0	0	0	0	0	0	0	0	1
2569	FA13B_HUMAN	Protein FAM13B	S10	91%	0.91	7	0	0	0	0	0	0	0	0	1	0	0	0	1

2570	F18B1_HUMAN	Protein FAM18B1	S6	97%	0.97	1	0	0	0	0	0	0	0	0	0	0	1	0	1
2571	F18B2_HUMAN	Protein FAM18B2	S6	97%	0.97	1	0	0	0	0	0	0	0	0	0	0	1	0	1
2572	F193A_HUMAN	Protein FAM193A	S293	100%	1.00	1	1	0	0	0	0	0	0	0	0	0	0	0	1
2573	F195B_HUMAN	Protein FAM195B	S17	50%	0.50	28	0	0	1	2	1	0	2	1	1	2	1	0	11
2574	F195B_HUMAN	Protein FAM195B	S18	60%	0.60	28	0	0	1	2	1	0	2	1	1	2	1	0	11
2575	F195B_HUMAN	Protein FAM195B	S21	100%	1.00	61	0	0	3	4	2	0	4	2	1	4	3	0	23
2576	F195B_HUMAN	Protein FAM195B	T16	54%	0.54	6	0	0	0	0	0	0	0	0	1	0	0	0	1
2577	F198A_HUMAN	Protein FAM198A	T123	100%	1.00	1	0	0	0	0	0	0	0	0	0	1	0	0	1
2578	FA40A_HUMAN	Protein FAM40A	S335	100%	1.00	134	2	5	2	0	2	3	4	4	2	0	0	0	24
2579	FA65A_HUMAN	Protein FAM65A	S200	86%	0.86	3	0	0	0	0	1	0	0	0	0	0	0	0	1
2580	FA65A_HUMAN	Protein FAM65A	S351	44%	0.44	1	0	0	0	0	0	0	0	0	0	1	0	0	1
2581	FA65A_HUMAN	Protein FAM65A	T192	99%	0.99	3	0	0	0	0	1	0	0	0	0	0	0	0	1
2582	FA65A_HUMAN	Protein FAM65A	Y191	98%	0.98	3	0	0	0	0	1	0	0	0	0	0	0	0	1
2583	FA69A_HUMAN	Protein FAM69A	S230	97%	0.97	13	0	0	0	0	0	1	0	0	0	0	0	0	1
2584	FA69A_HUMAN	Protein FAM69A	S235	94%	0.94	13	0	0	0	0	0	1	0	0	0	0	0	0	1
2585	FA69A_HUMAN	Protein FAM69A	S240	88%	0.88	13	0	0	0	0	0	1	0	0	0	0	0	0	1
2586	FA69A_HUMAN	Protein FAM69A	S251	88%	0.88	13	0	0	0	0	0	1	0	0	0	0	0	0	1
2587	FA69A_HUMAN	Protein FAM69A	T234	96%	0.96	13	0	0	0	0	0	1	0	0	0	0	0	0	1
2588	FA69A_HUMAN	Protein FAM69A	Y237	90%	0.90	12	0	0	0	0	0	1	0	0	0	0	0	0	1
2589	FA71B_HUMAN	Protein FAM71B	T265	100%	1.00	1	0	0	0	0	1	0	0	0	0	0	0	0	1

2590	FA71B_HUMAN	Protein FAM71B	T300	23%	0.23	1	0	0	0	0	1	0	0	0	0	0	0	0	1
2591	FA71B_HUMAN	Protein FAM71B	T301	23%	0.23	1	0	0	0	0	1	0	0	0	0	0	0	0	1
2592	FA83B_HUMAN	Protein FAM83B	S664	100%	1.00	6	1	0	0	0	2	1	0	0	0	1	0	1	6
2593	FA83C_HUMAN	Protein FAM83C	S504	95%	0.95	1	0	0	0	0	0	0	0	0	0	1	0	0	1
2594	FA83D_HUMAN	Protein FAM83D	S17	100%	1.00	3	0	0	0	0	0	0	0	0	0	1	1	0	2
2595	FA83G_HUMAN	Protein FAM83G	S127	100%	1.00	4	0	0	0	0	0	0	0	0	0	0	1	0	1
2596	FA83H_HUMAN	Protein FAM83H	S1003	100%	1.00	121	2	6	6	6	2	2	3	0	1	0	2	1	31
2597	FA83H_HUMAN	Protein FAM83H	S1025	23%	0.23	3	0	0	0	0	0	0	0	0	0	0	0	1	1
2598	FA83H_HUMAN	Protein FAM83H	S647	100%	1.00	20	0	0	0	0	1	1	0	0	0	4	4	4	14
2599	FA83H_HUMAN	Protein FAM83H	S914	100%	1.00	1	0	0	0	0	1	0	0	0	0	0	0	0	1
2600	FA83H_HUMAN	Protein FAM83H	S936	100%	1.00	195	0	4	2	2	5	4	0	0	2	6	7	6	38
2601	FA83H_HUMAN	Protein FAM83H	S942	20%	0.20	5	0	0	0	0	1	0	0	0	0	0	1	0	2
2602	FA83H_HUMAN	Protein FAM83H	S945	20%	0.20	1	0	0	0	0	0	0	1	0	0	0	0	0	1
2603	FA83H_HUMAN	Protein FAM83H	S998	100%	1.00	30	0	0	0	0	0	0	0	0	0	1	0	0	1
2604	FA83H_HUMAN	Protein FAM83H	T938	80%	0.80	29	0	1	1	1	0	1	0	0	0	0	0	1	5
2605	FA83H_HUMAN	Protein FAM83H	T940	20%	0.20	5	1	0	0	0	0	0	0	0	1	0	0	0	2
2606	FEM1A_HUMAN	Protein fem-1 homolog A	S428	79%	0.79	2	0	0	0	0	0	0	0	1	0	0	0	0	1
2607	FEM1A_HUMAN	Protein fem-1 homolog A	S433	83%	0.83	2	0	0	0	0	0	0	0	1	0	0	0	0	1
2608	FEM1A_HUMAN	Protein fem-1 homolog A	S434	84%	0.84	2	0	0	0	0	0	0	0	1	0	0	0	0	1
2609	FEM1A_HUMAN	Protein fem-1 homolog A	S437	88%	0.88	2	0	0	0	0	0	0	0	1	0	0	0	0	1

2610	FEM1A_HUMAN	Protein fem-1 homolog A	T431	92%	0.92	2	0	0	0	0	0	0	0	1	0	0	0	0	1
2611	FEM1A_HUMAN	Protein fem-1 homolog A	Y415	71%	0.71	1	0	0	0	0	0	0	0	1	0	0	0	0	1
2612	FEM1A_HUMAN	Protein fem-1 homolog A	Y444	71%	0.71	2	0	0	0	0	0	0	0	1	0	0	0	0	1
2613	FLII_HUMAN	Protein flightless-1 homolog	S856	100%	1.00	27	0	0	1	1	0	3	0	0	1	0	6	4	16
2614	FRY_HUMAN	Protein furry homolog	S903	33%	0.33	5	0	0	0	0	0	0	0	0	0	0	0	1	1
2615	FRY_HUMAN	Protein furry homolog	S907	88%	0.88	4	0	0	0	0	0	0	0	0	0	0	0	1	1
2616	FRYL_HUMAN	Protein furry homolog-like	T1959	99%	0.99	1	0	0	0	0	0	0	1	0	0	0	0	0	1
2617	HAIR_HUMAN	Protein hairless	S453	88%	0.88	1	0	0	0	0	0	0	0	0	0	0	0	1	1
2618	HAIR_HUMAN	Protein hairless	T452	84%	0.84	1	0	0	0	0	0	0	0	0	0	0	0	1	1
2619	K0284_HUMAN	Protein KIAA0284	S1179	100%	1.00	13	0	0	0	0	0	1	0	0	0	4	1	4	10
2620	K0284_HUMAN	Protein KIAA0284	S1548	100%	1.00	62	0	0	0	0	0	0	0	0	0	3	2	1	6
2621	K0284_HUMAN	Protein KIAA0284	S1551	92%	0.92	9	0	0	0	0	0	0	0	0	0	1	2	0	3
2622	K0284_HUMAN	Protein KIAA0284	S853	100%	1.00	8	0	1	0	0	0	0	0	0	0	0	0	1	2
2623	K0284_HUMAN	Protein KIAA0284	T1181	1%	0.01	1	0	0	0	0	0	0	0	0	0	0	1	0	1
2624	K0284_HUMAN	Protein KIAA0284	T1304	100%	1.00	3	0	0	1	1	0	0	0	0	0	0	1	0	3
2625	K0284_HUMAN	Protein KIAA0284	T542	63%	0.63	2	0	0	0	0	0	0	0	0	0	1	1	0	2
2626	KPCA_HUMAN	Protein kinase C alpha type	T638	100%	1.00	3	0	1	0	0	0	0	1	1	0	0	0	0	3
2627	PACN3_HUMAN	Protein kinase C and casein kinase substrate in neurons protein 3	S354	98%	0.98	3	1	0	0	0	0	0	0	0	0	0	0	0	1
2628	KPCD_HUMAN	Protein kinase C delta type	S302	74%	0.74	2	0	1	0	0	0	0	0	0	0	0	0	0	1

2629	PRDBP_HUMAN	Protein kinase C delta-binding protein	S165	100%	1.00	125	2	5	3	5	4	0	5	0	0	8	4	0	36
2630	PRDBP_HUMAN	Protein kinase C delta-binding protein	S166	100%	1.00	119	2	5	3	4	4	0	5	0	0	6	4	0	33
2631	KPCI_HUMAN	Protein kinase C iota type	T564	100%	1.00	10	2	1	0	0	0	1	0	0	0	1	0	0	5
2632	PKCB1_HUMAN	Protein kinase C-binding protein 1	S1089	7%	0.07	1	0	0	0	0	0	0	0	0	0	0	0	1	1
2633	KRI1_HUMAN	Protein KRI1 homolog	S177	100%	1.00	228	6	2	0	1	6	2	5	0	4	0	2	0	28
2634	LIN52_HUMAN	Protein lin-52 homolog	S28	100%	1.00	2	0	2	0	0	0	0	0	0	0	0	0	0	2
2635	LS14A_HUMAN	Protein LSM14 homolog A	S192	100%	1.00	707	9	11	16	19	12	14	14	12	16	11	9	13	156
2636	LS14A_HUMAN	Protein LSM14 homolog A	S203	25%	0.25	15	0	0	0	1	0	0	2	0	0	0	0	0	3
2637	LS14A_HUMAN	Protein LSM14 homolog A	T194	88%	0.88	517	12	10	12	11	5	10	7	8	10	7	6	7	105
2638	LS14A_HUMAN	Protein LSM14 homolog A	T201	26%	0.26	10	0	1	1	0	1	0	0	1	0	0	0	0	4
2639	LTV1_HUMAN	Protein LTV1 homolog	S331	100%	1.00	106	3	2	3	0	3	3	3	3	3	1	1	0	25
2640	LYRIC_HUMAN	Protein LYRIC	S216	7%	0.07	1	0	0	0	0	0	0	0	0	0	1	0	0	1
2641	M21D2_HUMAN	Protein MB21D2	S436	63%	0.63	2	0	0	0	0	0	0	0	0	0	1	0	0	1
2642	M21D2_HUMAN	Protein MB21D2	T435	48%	0.48	2	0	0	0	1	0	0	0	0	0	0	0	0	1
2643	NDRG1_HUMAN	Protein NDRG1	S2	100%	1.00	132	6	6	3	4	3	4	2	1	3	9	5	3	49
2644	NDRG1_HUMAN	Protein NDRG1	S364	71%	0.71	13	0	1	0	1	2	0	1	0	0	1	1	2	9
2645	NDRG1_HUMAN	Protein NDRG1	S367	17%	0.17	7	0	1	0	0	0	0	0	1	0	1	2	0	5
2646	NDRG1_HUMAN	Protein NDRG1	S378	52%	0.52	1	0	0	0	0	0	0	0	0	0	0	0	1	1
2647	NDRG1_HUMAN	Protein NDRG1	T366	85%	0.85	135	5	7	4	6	5	6	7	4	6	9	8	8	75
2648	NXP20_HUMAN	Protein NOXP20	S196	98%	0.98	5	0	0	0	0	0	0	0	0	0	1	0	0	1

2649	NXP20_HUMAN	Protein NOXP20	T199	10%	0.10	5	0	0	0	0	0	0	0	0	0	1	0	0	1
2650	NPAT_HUMAN	Protein NPAT	S1324	18%	0.18	1	1	0	0	0	0	0	0	0	0	0	0	0	1
2651	NPAT_HUMAN	Protein NPAT	S1327	32%	0.32	1	1	0	0	0	0	0	0	0	0	0	0	0	1
2652	NPAT_HUMAN	Protein NPAT	S774	23%	0.23	1	1	0	0	0	0	0	0	0	0	0	0	0	1
2653	NPAT_HUMAN	Protein NPAT	S775	27%	0.27	1	0	0	0	0	0	1	0	0	0	0	0	0	1
2654	NPAT_HUMAN	Protein NPAT	T770	35%	0.35	1	1	0	0	0	0	0	0	0	0	0	0	0	1
2655	NPAT_HUMAN	Protein NPAT	T777	13%	0.13	1	0	0	0	0	0	1	0	0	0	0	0	0	1
2656	OS9_HUMAN	Protein OS-9	T5	93%	0.93	5	0	1	0	0	0	0	0	0	0	0	0	0	1
2657	MYPT1_HUMAN	Protein phosphatase 1 regulatory subunit 12A	S509	33%	0.33	1	0	0	0	0	0	0	0	0	0	0	0	1	1
2658	PP14B_HUMAN	Protein phosphatase 1 regulatory subunit 14B	S32	100%	1.00	14	0	0	0	0	1	1	0	0	1	4	4	2	13
2659	PPR1B_HUMAN	Protein phosphatase 1 regulatory subunit 1B	S102	100%	1.00	57	0	0	0	0	0	0	0	0	0	7	6	4	17
2660	PPR3C_HUMAN	Protein phosphatase 1 regulatory subunit 3C	S213	73%	0.73	1	0	0	0	0	1	0	0	0	0	0	0	0	1
2661	PPR3C_HUMAN	Protein phosphatase 1 regulatory subunit 3C	S217	81%	0.81	1	0	0	0	0	1	0	0	0	0	0	0	0	1
2662	PPR3C_HUMAN	Protein phosphatase 1 regulatory subunit 3C	T215	58%	0.58	1	0	0	0	0	1	0	0	0	0	0	0	0	1
2663	PPR3C_HUMAN	Protein phosphatase 1 regulatory subunit 3C	Y196	35%	0.35	1	0	0	0	0	1	0	0	0	0	0	0	0	1
2664	PPR3C_HUMAN	Protein phosphatase 1 regulatory subunit 3C	Y208	35%	0.35	1	0	0	0	0	1	0	0	0	0	0	0	0	1
2665	IPP2_HUMAN	Protein phosphatase inhibitor 2	S87	69%	0.69	5	1	0	0	0	1	0	0	2	0	0	0	0	4
2666	IPP2_HUMAN	Protein phosphatase inhibitor 2	T89	20%	0.20	7	0	0	2	0	0	0	0	1	0	1	2	0	6
2667	IPP2_HUMAN	Protein phosphatase inhibitor 2	T92	6%	0.06	1	0	0	0	0	0	0	0	0	0	1	0	0	1

2668	SSH1_HUMAN	Protein phosphatase Slingshot homolog 1	S515	99%	0.99	8	0	0	0	0	0	0	0	0	1	2	2	0	5
2669	SSH2_HUMAN	Protein phosphatase Slingshot homolog 2	S784	100%	1.00	10	0	0	0	0	0	1	1	0	1	0	0	0	3
2670	SSH3_HUMAN	Protein phosphatase Slingshot homolog 3	S9	15%	0.15	2	0	1	0	0	0	0	0	0	0	0	0	0	1
2671	PIEZ2_HUMAN	Protein PIEZO2	T2582	45%	0.45	1	0	0	0	0	0	0	0	1	0	0	0	0	1
2672	PML_HUMAN	Protein PML	S518	100%	1.00	10	0	0	1	0	0	0	1	0	0	0	0	0	2
2673	PML_HUMAN	Protein PML	S527	100%	1.00	10	0	0	1	0	0	0	1	0	0	0	0	0	2
2674	PML_HUMAN	Protein PML	S530	100%	1.00	30	0	0	0	0	0	0	0	0	1	0	0	0	1
2675	PML_HUMAN	Protein PML	S535	72%	0.72	1	0	0	0	0	0	0	1	0	0	0	0	0	1
2676	PB1_HUMAN	Protein polybromo-1	S10	82%	0.82	2	1	0	0	1	0	0	0	0	0	0	0	0	2
2677	PB1_HUMAN	Protein polybromo-1	S12	4%	0.04	1	1	0	0	0	0	0	0	0	0	0	0	0	1
2678	PB1_HUMAN	Protein polybromo-1	S39	100%	1.00	43	0	0	0	0	0	0	0	0	0	0	2	0	2
2679	PB1_HUMAN	Protein polybromo-1	T43	68%	0.68	17	0	0	0	0	0	0	0	0	0	1	1	0	2
2680	PRCA1_HUMAN	Protein PROCA1	S48	75%	0.75	1	0	0	0	0	1	0	0	0	0	0	0	0	1
2681	PRCA1_HUMAN	Protein PROCA1	S49	19%	0.19	1	0	0	0	0	0	0	1	0	0	0	0	0	1
2682	PRCA1_HUMAN	Protein PROCA1	T50	44%	0.44	2	0	0	0	0	1	0	1	0	0	0	0	0	2
2683	PRC2A_HUMAN	Protein PRRC2A	S1147	100%	1.00	1	0	0	0	0	0	0	0	0	0	0	0	1	1
2684	PRC2A_HUMAN	Protein PRRC2A	S1219	100%	1.00	58	0	0	0	0	0	0	0	0	0	4	3	2	9
2685	PRC2A_HUMAN	Protein PRRC2A	T610	100%	1.00	101	0	0	0	2	1	1	0	1	1	1	1	0	8
2686	PRC2B_HUMAN	Protein PRRC2B	S226	95%	0.95	5	0	0	0	1	1	0	0	0	0	0	0	0	2

2687	PRC2B_HUMAN	Protein PRRC2B	S388	100%	1.00	100	2	0	0	6	1	0	6	4	2	3	1	2	27
2688	PRC2C_HUMAN	Protein PRRC2C	S779	100%	1.00	1	0	0	0	0	0	0	0	0	0	1	0	0	1
2689	PRC2C_HUMAN	Protein PRRC2C	S878	99%	0.99	55	2	0	3	2	0	0	1	1	0	2	1	0	12
2690	PRC2C_HUMAN	Protein PRRC2C	S893	24%	0.24	2	0	0	0	0	0	1	1	0	0	0	0	0	2
2691	PRC2C_HUMAN	Protein PRRC2C	T2673	100%	1.00	6	0	0	0	0	1	0	0	0	0	0	0	0	1
2692	PRC2C_HUMAN	Protein PRRC2C	T887	91%	0.91	9	1	0	0	0	0	1	0	0	0	0	2	0	4
2693	PTHB1_HUMAN	Protein PTHB1	S820	0%	0.00	2	0	0	0	0	0	0	0	2	0	0	0	0	2
2694	RER1_HUMAN	Protein RER1	S100	10%	0.10	2	0	1	0	0	0	0	1	0	0	0	0	0	2
2695	RER1_HUMAN	Protein RER1	S2	100%	1.00	27	1	0	0	2	1	1	0	2	2	0	1	2	12
2696	RER1_HUMAN	Protein RER1	S95	100%	1.00	55	4	2	2	2	3	2	5	6	0	6	6	0	38
2697	S10AD_HUMAN	Protein S100-A13	S32	100%	1.00	1	0	0	0	0	0	0	0	0	0	0	0	1	1
2698	S10AG_HUMAN	Protein S100-A16	S2	97%	0.97	1	0	0	0	0	0	0	0	0	0	1	0	0	1
2699	SAAL1_HUMAN	Protein SAAL1	S6	100%	1.00	195	4	7	10	10	9	7	10	5	6	3	7	6	84
2700	SCRIB_HUMAN	Protein scribble homolog	S1348	100%	1.00	17	0	1	0	0	0	1	0	2	0	8	2	1	15
2701	SCRIB_HUMAN	Protein scribble homolog	S1353	10%	0.10	4	0	0	1	0	0	1	0	0	0	0	0	0	2
2702	SCRIB_HUMAN	Protein scribble homolog	S1378	100%	1.00	4	0	0	0	0	0	0	0	0	0	0	1	1	2
2703	SCRIB_HUMAN	Protein scribble homolog	S1448	95%	0.95	2	0	0	0	0	0	0	0	0	0	1	1	0	2
2704	SCRIB_HUMAN	Protein scribble homolog	S1561	15%	0.15	4	0	0	0	0	0	0	0	0	1	0	1	0	2
2705	SCRIB_HUMAN	Protein scribble homolog	S1566	98%	0.98	83	1	0	0	2	0	1	0	0	2	0	3	0	9
2706	SCRIB_HUMAN	Protein scribble homolog	S504	100%	1.00	9	0	0	0	0	0	0	0	0	0	5	1	1	7

2707	SCRIB_HUMAN	Protein scribble homolog	S853	100%	1.00	7	0	0	0	0	0	0	0	0	0	3	4	0	7
2708	SDA1_HUMAN	Protein SDA1 homolog	S585	100%	1.00	76	2	0	1	1	1	1	1	0	3	1	0	0	11
2709	SHRM1_HUMAN	Protein Shroom1	S133	98%	0.98	3	1	0	0	0	0	0	0	0	0	0	0	0	1
2710	SHRM3_HUMAN	Protein Shroom3	S910	82%	0.82	1	0	0	0	0	0	0	0	0	1	0	0	0	1
2711	SLMO1_HUMAN	Protein slowmo homolog 1	S56	75%	0.75	1	0	0	0	0	0	0	0	1	0	0	0	0	1
2712	SLMO1_HUMAN	Protein slowmo homolog 1	S63	99%	0.99	1	0	0	0	0	0	0	0	1	0	0	0	0	1
2713	SLMO1_HUMAN	Protein slowmo homolog 1	T57	61%	0.61	1	0	0	0	0	0	0	0	1	0	0	0	0	1
2714	SMAG2_HUMAN	Protein Smaug homolog 2	S592	100%	1.00	10	0	0	0	0	0	0	0	0	0	1	0	1	2
2715	SMG5_HUMAN	Protein SMG5	S11	92%	0.92	1	0	0	0	0	0	0	0	0	0	0	0	1	1
2716	SMG9_HUMAN	Protein SMG9	S7	86%	0.86	3	0	0	1	0	0	0	0	0	0	0	0	0	1
2717	SON_HUMAN	Protein SON	S1556	100%	1.00	305	8	5	6	4	5	4	5	6	7	7	5	5	67
2718	SON_HUMAN	Protein SON	S1697	100%	1.00	336	3	5	7	4	7	6	4	5	7	8	5	7	68
2719	SOX15_HUMAN	Protein SOX-15	S37	100%	1.00	171	9	11	8	8	8	6	0	0	0	2	6	3	61
2720	SBNO1_HUMAN	Protein strawberry notch homolog 1	S212	16%	0.16	3	1	0	0	0	0	0	0	2	0	0	0	0	3
2721	SBNO1_HUMAN	Protein strawberry notch homolog 1	S214	97%	0.97	3	1	0	0	0	0	0	0	2	0	0	0	0	3
2722	TANC1_HUMAN	Protein TANC1	S732	85%	0.85	1	0	0	0	0	0	0	0	0	0	0	1	0	1
2723	TFG_HUMAN	Protein TFG	S197	100%	1.00	1	0	0	0	0	0	0	1	0	0	0	0	0	1
2724	SC16A_HUMAN	Protein transport protein Sec16A	S1786	100%	1.00	172	0	2	1	4	3	7	2	3	3	10	8	6	49
2725	SC16A_HUMAN	Protein transport protein Sec16A	S1905	100%	1.00	82	0	1	1	2	4	3	2	1	3	0	0	1	18
2726	SC16A_HUMAN	Protein transport protein Sec16A	T1810	98%	0.98	1	0	1	0	0	0	0	0	0	0	0	0	0	1

2727	SC31A_HUMAN	Protein transport protein Sec31A	S799	100%	1.00	1	0	0	0	0	0	0	1	0	0	0	0	0	1
2728	SC61B_HUMAN	Protein transport protein Sec61 subunit beta	S13	48%	0.48	1	0	0	0	0	0	1	0	0	0	0	0	0	1
2729	SC61B_HUMAN	Protein transport protein Sec61 subunit beta	S19	13%	0.13	1	0	0	0	0	0	0	0	0	0	1	0	0	1
2730	UNC79_HUMAN	Protein unc-79 homolog	S1977	36%	0.36	1	0	0	0	0	0	0	0	1	0	0	0	0	1
2731	UNC79_HUMAN	Protein unc-79 homolog	S1978	36%	0.36	1	0	0	0	0	0	0	0	1	0	0	0	0	1
2732	UNC79_HUMAN	Protein unc-79 homolog	T1975	85%	0.85	1	0	0	0	0	0	0	0	1	0	0	0	0	1
2733	UNC79_HUMAN	Protein unc-79 homolog	T2005	18%	0.18	1	0	0	0	0	0	0	0	1	0	0	0	0	1
2734	VKIND_HUMAN	Protein very KIND	S1715	98%	0.98	1	0	0	0	0	0	0	0	0	0	0	1	0	1
2735	VKIND_HUMAN	Protein very KIND	S1721	93%	0.93	1	0	0	0	0	0	0	0	0	0	0	1	0	1
2736	VKIND_HUMAN	Protein very KIND	T1716	98%	0.98	1	0	0	0	0	0	0	0	0	0	0	1	0	1
2737	VIR_HUMAN	Protein virilizer homolog	S133	97%	0.97	58	5	3	2	4	5	3	4	2	2	1	2	0	33
2738	VIR_HUMAN	Protein virilizer homolog	S138	93%	0.93	94	4	4	1	4	1	3	2	3	3	6	2	4	37
2739	VIR_HUMAN	Protein virilizer homolog	S173	100%	1.00	55	4	1	3	3	1	2	0	3	4	3	2	2	28
2740	VPRBP_HUMAN	Protein VPRBP	S1000	100%	1.00	23	0	3	0	0	0	0	0	0	0	0	1	1	5
2741	VPRBP_HUMAN	Protein VPRBP	T1003	36%	0.36	1	0	0	0	0	0	0	0	0	0	0	0	1	1
2742	YIPF2_HUMAN	Protein YIPF2	S3	81%	0.81	9	0	2	0	0	0	0	0	0	0	0	3	0	5
2743	YIPF2_HUMAN	Protein YIPF2	T16	12%	0.12	1	0	0	0	0	0	0	0	1	0	0	0	0	1
2744	YIPF2_HUMAN	Protein YIPF2	T8	36%	0.36	7	0	0	0	0	1	0	0	0	0	3	0	1	5
2745	PAR1_HUMAN	Proteinase-activated receptor 1	S197	99%	0.99	1	0	0	0	0	0	0	0	0	0	0	1	0	1
2746	PAR1_HUMAN	Proteinase-activated receptor 1	T179	100%	1.00	1	0	0	0	0	0	0	0	0	0	0	1	0	1

2747	PAR1_HUMAN	Proteinase-activated receptor 1	T194	96%	0.96	1	0	0	0	0	0	0	0	0	0	0	1	0	1
2748	PAR1_HUMAN	Proteinase-activated receptor 1	Y183	99%	0.99	1	0	0	0	0	0	0	0	0	0	0	1	0	1
2749	TGM3_HUMAN	Protein-glutamine gamma-glutamyltransferase E	S249	100%	1.00	1	0	0	1	0	0	0	0	0	0	0	0	0	1
2750	TGM3_HUMAN	Protein-glutamine gamma-glutamyltransferase E	S253	100%	1.00	1	0	0	1	0	0	0	0	0	0	0	0	0	1
2751	TGM7_HUMAN	Protein-glutamine gamma-glutamyltransferase Z	S92	27%	0.27	1	0	0	0	0	0	1	0	0	0	0	0	0	1
2752	TGM7_HUMAN	Protein-glutamine gamma-glutamyltransferase Z	Y116	11%	0.11	1	0	0	0	0	0	1	0	0	0	0	0	0	1
2753	FAK2_HUMAN	Protein-tyrosine kinase 2-beta	S375	100%	1.00	27	0	0	1	1	1	0	0	0	0	0	0	1	4
2754	PRG4_HUMAN	Proteoglycan 4	T548	9%	0.09	1	0	0	0	0	0	0	0	0	0	0	0	1	1
2755	PCDA4_HUMAN	Protocadherin alpha-4	S760	98%	0.98	15	1	0	1	1	0	0	0	0	1	0	0	0	4
2756	PCDA4_HUMAN	Protocadherin alpha-4	S773	100%	1.00	19	1	0	1	1	0	1	0	0	1	0	0	0	5
2757	PCDA4_HUMAN	Protocadherin alpha-4	S775	98%	0.98	19	1	0	1	1	0	1	0	0	1	0	0	0	5
2758	PCDA4_HUMAN	Protocadherin alpha-4	T767	24%	0.24	4	0	0	0	0	0	1	0	0	0	0	0	0	1
2759	PCDH1_HUMAN	Protocadherin-1	S962	100%	1.00	4	0	0	0	0	0	0	0	0	0	2	0	0	2
2760	PCD16_HUMAN	Protocadherin-16	S1753	47%	0.47	1	0	0	0	0	0	0	0	0	1	0	0	0	1
2761	PCD16_HUMAN	Protocadherin-16	T942	53%	0.53	5	0	0	0	0	0	0	1	0	0	1	0	1	3
2762	PCD16_HUMAN	Protocadherin-16	T946	75%	0.75	4	0	0	0	0	0	0	1	0	0	1	0	1	3
2763	PCD19_HUMAN	Protocadherin-19	S803	48%	0.48	1	0	0	0	0	0	0	1	0	0	0	0	0	1
2764	PCD19_HUMAN	Protocadherin-19	S809	48%	0.48	1	0	0	0	0	0	0	1	0	0	0	0	0	1
2765	PCD19_HUMAN	Protocadherin-19	S810	48%	0.48	1	0	0	0	0	0	0	1	0	0	0	0	0	1

2766	PCD19_HUMAN	Protocadherin-19	T808	48%	0.48	1	0	0	0	0	0	0	1	0	0	0	0	0	1
2767	PCD19_HUMAN	Protocadherin-19	T820	71%	0.71	1	0	0	0	0	0	0	1	0	0	0	0	0	1
2768	PRTG_HUMAN	Protogenin	S37	100%	1.00	19	1	0	1	0	0	0	0	0	0	0	0	1	3
2769	PRTG_HUMAN	Protogenin	S40	100%	1.00	19	1	0	1	0	0	0	0	0	0	0	0	1	3
2770	PCFT_HUMAN	Proton-coupled folate transporter	S187	100%	1.00	1	0	0	0	0	0	0	1	0	0	0	0	0	1
2771	PCFT_HUMAN	Proton-coupled folate transporter	T178	100%	1.00	1	0	0	0	0	0	0	1	0	0	0	0	0	1
2772	SRC_HUMAN	Proto-oncogene tyrosine-protein kinase Src	S17	100%	1.00	181	2	1	0	1	2	1	1	1	0	0	0	1	10
2773	GULP1_HUMAN	PTB domain-containing engulfment adapter protein 1	S213	12%	0.13	2	0	0	0	0	0	0	0	0	0	1	0	0	1
2774	PUM1_HUMAN	Pumilio homolog 1	S206	42%	0.42	12	0	0	1	1	1	0	1	0	2	1	0	1	8
2775	PUM1_HUMAN	Pumilio homolog 1	S209	100%	1.00	50	3	4	4	1	1	2	4	5	6	1	0	1	32
2776	PDPK2_HUMAN	Putative 3-phosphoinositide-dependent protein kinase 2	S214	100%	1.00	104	2	3	2	1	0	0	3	1	0	4	2	0	18
2777	AATC2_HUMAN	Putative aspartate aminotransferase, cytoplasmic 2	S19	100%	1.00	1	0	0	0	0	0	0	0	0	0	0	1	0	1
2778	AATC2_HUMAN	Putative aspartate aminotransferase, cytoplasmic 2	S5	100%	1.00	1	0	0	0	0	0	0	0	0	0	0	1	0	1
2779	AATC2_HUMAN	Putative aspartate aminotransferase, cytoplasmic 2	T3	100%	1.00	1	0	0	0	0	0	0	0	0	0	0	1	0	1
2780	HS905_HUMAN	Putative heat shock protein HSP 90-alpha A5	S89	100%	1.00	196	3	1	3	6	5	5	3	5	3	2	2	5	43
2781	H90B2_HUMAN	Putative heat shock protein HSP 90-beta 2	S177	100%	1.00	18	0	0	0	0	0	0	0	0	0	0	18	0	18
2782	H90B2_HUMAN	Putative heat shock protein HSP 90-beta 2	T79	36%	0.36	1	0	0	0	0	0	0	0	0	0	0	1	0	1

2783	H90B2_HUMAN	Putative heat shock protein HSP 90-beta 2	T89	87%	0.87	1	0	0	0	0	0	0	0	0	0	0	1	0	1
2784	HIN1L_HUMAN	Putative HIN1-like protein	S202	74%	0.74	1	0	0	0	0	0	0	1	0	0	0	0	0	1
2785	HIN1L_HUMAN	Putative HIN1-like protein	S208	25%	0.25	1	0	0	0	0	0	0	1	0	0	0	0	0	1
2786	NBPF7_HUMAN	Putative neuroblastoma breakpoint family member 7	S140	100%	1.00	1	0	1	0	0	0	0	0	0	0	0	0	0	1
2787	NBPF7_HUMAN	Putative neuroblastoma breakpoint family member 7	S142	96%	0.96	1	0	1	0	0	0	0	0	0	0	0	0	0	1
2788	NDK8_HUMAN	Putative nucleoside diphosphate kinase	T79	99%	0.99	1	0	0	0	0	0	0	0	0	0	0	0	1	1
2789	YI035_HUMAN	Putative olfactory receptor ENSP00000348552	S3	71%	0.71	1	0	0	0	0	0	1	0	0	0	0	0	0	1
2790	YI035_HUMAN	Putative olfactory receptor ENSP00000348552	T7	44%	0.44	1	0	0	0	0	0	1	0	0	0	0	0	0	1
2791	PBPL2_HUMAN	Putative platelet basic protein-like 2	T79	99%	0.99	4	0	0	0	1	0	0	0	0	0	0	0	0	1
2792	ASXL3_HUMAN	Putative Polycomb group protein ASXL3	S296	58%	0.58	1	0	0	0	0	0	0	1	0	0	0	0	0	1
2793	ASXL3_HUMAN	Putative Polycomb group protein ASXL3	S305	58%	0.58	1	0	0	0	0	0	0	1	0	0	0	0	0	1
2794	ASXL3_HUMAN	Putative Polycomb group protein ASXL3	Y314	66%	0.66	1	0	0	0	0	0	0	1	0	0	0	0	0	1
2795	F90AM_HUMAN	Putative protein FAM90A22	S190	43%	0.43	1	0	0	0	0	0	0	0	1	0	0	0	0	1
2796	F90AM_HUMAN	Putative protein FAM90A22	S192	43%	0.43	1	0	0	0	0	0	0	0	1	0	0	0	0	1
2797	F90AM_HUMAN	Putative protein FAM90A22	S193	43%	0.43	1	0	0	0	0	0	0	0	1	0	0	0	0	1
2798	NOP2_HUMAN	Putative ribosomal RNA methyltransferase NOP2	S786	100%	1.00	34	0	0	0	5	0	1	0	0	1	4	1	4	16
2799	RBM15_HUMAN	Putative RNA-binding protein 15	S294	99%	0.99	4	0	0	0	0	0	0	0	0	0	0	4	0	4
2800	RBM15_HUMAN	Putative RNA-binding protein 15	S659	80%	0.80	1	0	0	1	0	0	0	0	0	0	0	0	0	1

2801	RBM15_HUMAN	Putative RNA-binding protein 15	S700	100%	1.00	14	0	0	0	0	0	0	0	0	0	1	0	2	3
2802	YE016_HUMAN	Putative TAF11-like protein ENSP00000332601	T3	38%	0.38	2	0	0	0	0	0	0	1	0	0	0	1	0	2
2803	CA200_HUMAN	Putative uncharacterized protein C1orf200	S98	36%	0.36	1	0	0	0	0	0	0	0	1	0	0	0	0	1
2804	CA200_HUMAN	Putative uncharacterized protein C1orf200	T86	36%	0.36	1	0	0	0	0	0	0	0	1	0	0	0	0	1
2805	CA200_HUMAN	Putative uncharacterized protein C1orf200	Y116	36%	0.36	1	0	0	0	0	0	0	0	1	0	0	0	0	1
2806	CA200_HUMAN	Putative uncharacterized protein C1orf200	Y90	36%	0.36	1	0	0	0	0	0	0	0	1	0	0	0	0	1
2807	YS038_HUMAN	Putative uncharacterized protein DKFZp434G1729	S10	56%	0.56	3	0	0	0	0	0	0	0	1	0	0	0	0	1
2808	YS038_HUMAN	Putative uncharacterized protein DKFZp434G1729	S13	64%	0.64	2	0	0	0	0	0	0	0	1	0	0	0	0	1
2809	YS038_HUMAN	Putative uncharacterized protein DKFZp434G1729	S25	20%	0.20	2	0	0	0	0	0	0	0	1	0	0	0	0	1
2810	YS038_HUMAN	Putative uncharacterized protein DKFZp434G1729	S9	83%	0.83	3	0	0	0	0	0	0	0	1	0	0	0	0	1
2811	YS038_HUMAN	Putative uncharacterized protein DKFZp434G1729	S99	100%	1.00	1	0	0	0	0	0	0	0	0	0	0	1	0	1
2812	CR020_HUMAN	Putative uncharacterized protein encoded by NCRNA00305	T65	100%	1.00	1	0	0	0	0	0	0	0	0	0	0	1	0	1
2813	HUG1_HUMAN	Putative uncharacterized protein HUG-1	S51	99%	0.99	1	0	0	0	0	0	1	0	0	0	0	0	0	1
2814	HUG1_HUMAN	Putative uncharacterized protein HUG-1	S52	99%	0.99	1	0	0	0	0	0	1	0	0	0	0	0	0	1
2815	HUG1_HUMAN	Putative uncharacterized protein HUG-1	Y39	100%	1.00	1	0	0	0	0	0	1	0	0	0	0	0	0	1
2816	HUG1_HUMAN	Putative uncharacterized protein HUG-1	Y69	51%	0.51	1	0	0	0	0	0	1	0	0	0	0	0	0	1
2817	ZN724_HUMAN	Putative zinc finger protein 724	T23	43%	0.43	1	1	0	0	0	0	0	0	0	0	0	0	0	1

2818	ZN840_HUMAN	Putative zinc finger protein 840	S2	100%	1.00	1	0	1	0	0	0	0	0	0	0	0	0	0	1
2819	ZN840_HUMAN	Putative zinc finger protein 840	Y14	100%	1.00	1	0	1	0	0	0	0	0	0	0	0	0	0	1
2820	PWP2A_HUMAN	PWWP domain-containing protein 2A	S81	100%	1.00	26	0	0	1	1	0	0	0	0	1	0	0	0	3
2821	CF145_HUMAN	PX domain-containing protein C6orf145	S176	34%	0.34	2	0	0	0	0	0	0	0	0	0	1	0	0	1
2822	CF145_HUMAN	PX domain-containing protein C6orf145	T168	38%	0.38	1	0	0	0	0	0	0	0	0	0	1	0	0	1
2823	PDXD1_HUMAN	Pyridoxal-dependent decarboxylase domain-containing protein 1	T743	4%	0.04	1	0	0	1	0	0	0	0	0	0	0	0	0	1
2824	PNPO_HUMAN	Pyridoxine-5'-phosphate oxidase	S241	95%	0.95	2	0	0	0	0	0	0	0	0	0	0	1	0	1
2825	PYC_HUMAN	Pyruvate carboxylase, mitochondrial	S910	100%	1.00	2	0	0	0	0	0	0	0	0	0	0	1	0	1
2826	PYC_HUMAN	Pyruvate carboxylase, mitochondrial	S927	100%	1.00	2	0	0	0	0	0	0	0	0	0	0	1	0	1
2827	PYC_HUMAN	Pyruvate carboxylase, mitochondrial	T908	100%	1.00	2	0	0	0	0	0	0	0	0	0	0	1	0	1
2828	KPYM_HUMAN	Pyruvate kinase isozymes M1/M2	S2	84%	0.84	2	0	0	0	0	0	0	1	0	0	0	0	0	1
2829	KPYM_HUMAN	Pyruvate kinase isozymes M1/M2	S37	100%	1.00	241	8	8	11	0	6	2	0	2	1	11	12	11	72
2830	RFIP1_HUMAN	Rab11 family-interacting protein 1	T358	17%	0.17	1	0	1	0	0	0	0	0	0	0	0	0	0	1
2831	RB3GP_HUMAN	Rab3 GTPase-activating protein catalytic subunit	S9	17%	0.17	1	1	0	0	0	0	0	0	0	0	0	0	0	1
2832	RAF1_HUMAN	RAF proto-oncogene serine/threonine-protein kinase	S257	7%	0.07	1	0	1	0	0	0	0	0	0	0	0	0	0	1
2833	RAF1_HUMAN	RAF proto-oncogene serine/threonine-protein kinase	S259	57%	0.57	7	1	0	1	0	0	0	0	0	0	0	1	1	4
2834	RAF1_HUMAN	RAF proto-oncogene serine/threonine-protein kinase	T260	12%	0.12	1	0	0	0	0	0	0	0	0	1	0	0	0	1

2835	RGPA1_HUMAN	Ral GTPase-activating protein subunit alpha-1	S775	75%	0.75	2	0	0	0	0	0	0	0	0	0	1	0	0	1
2836	RGPA1_HUMAN	Ral GTPase-activating protein subunit alpha-1	T778	33%	0.33	13	1	1	1	0	1	0	1	0	0	1	2	2	10
2837	RAGP1_HUMAN	Ran GTPase-activating protein 1	S427	79%	0.79	38	0	0	0	2	1	1	0	0	0	1	1	0	6
2838	RAGP1_HUMAN	Ran GTPase-activating protein 1	S428	99%	0.99	336	3	3	3	5	6	7	4	1	5	12	10	12	71
2839	RAGP1_HUMAN	Ran GTPase-activating protein 1	S435	86%	0.86	40	2	0	1	2	0	0	0	2	1	3	1	0	12
2840	RAGP1_HUMAN	Ran GTPase-activating protein 1	S442	100%	1.00	774	12	15	14	16	12	9	5	5	8	11	10	8	125
2841	RAGP1_HUMAN	Ran GTPase-activating protein 1	T436	99%	0.99	102	3	3	2	3	4	3	2	2	3	6	4	2	37
2842	RANB3_HUMAN	Ran-binding protein 3	S333	100%	1.00	432	8	8	16	9	4	6	6	10	12	5	4	6	94
2843	RPGF6_HUMAN	Rap guanine nucleotide exchange factor 6	S230	100%	1.00	4	2	0	0	0	0	0	0	0	0	0	0	0	2
2844	RPGP2_HUMAN	Rap1 GTPase-activating protein 2	S45	58%	0.58	1	0	0	0	0	0	0	0	0	0	1	0	0	1
2845	RIN1_HUMAN	Ras and Rab interactor 1	S210	88%	0.88	16	0	0	0	1	0	1	0	0	0	3	2	3	10
2846	RIN1_HUMAN	Ras and Rab interactor 1	S258	100%	1.00	11	0	0	0	0	0	0	0	0	0	1	1	0	2
2847	RIN1_HUMAN	Ras and Rab interactor 1	T218	60%	0.60	4	0	0	0	0	0	0	0	0	0	0	1	0	1
2848	NGAP_HUMAN	Ras GTPase-activating protein nGAP	S887	42%	0.42	1	0	0	0	0	1	0	0	0	0	0	0	0	1
2849	NGAP_HUMAN	Ras GTPase-activating protein nGAP	S891	27%	0.27	1	0	0	0	0	1	0	0	0	0	0	0	0	1
2850	NGAP_HUMAN	Ras GTPase-activating protein nGAP	S904	27%	0.27	1	0	0	0	0	1	0	0	0	0	0	0	0	1
2851	NGAP_HUMAN	Ras GTPase-activating protein nGAP	S916	96%	0.96	1	0	0	0	0	1	0	0	0	0	0	0	0	1
2852	G3BP1_HUMAN	Ras GTPase-activating protein-binding protein 1	S149	100%	1.00	663	20	12	9	13	11	13	10	8	11	7	7	7	128
2853	G3BP1_HUMAN	Ras GTPase-activating protein-binding protein 1	S231	25%	0.25	19	1	1	0	0	0	0	0	1	0	0	0	0	3

2854	G3BP1_HUMAN	Ras GTPase-activating protein-binding protein 1	S232	98%	0.98	####	22	23	19	19	26	22	18	19	18	19	22	17	244
2855	IQGA1_HUMAN	Ras GTPase-activating-like protein IQGAP1	S19	96%	0.96	1	0	0	0	0	0	0	0	1	0	0	0	0	1
2856	IQGA1_HUMAN	Ras GTPase-activating-like protein IQGAP1	Y17	97%	0.97	1	0	0	0	0	0	0	0	1	0	0	0	0	1
2857	RAPH1_HUMAN	Ras-associated and pleckstrin homology domains-containing protein 1	S1098	94%	0.94	5	0	0	0	0	0	0	0	0	0	1	1	0	2
2858	RAPH1_HUMAN	Ras-associated and pleckstrin homology domains-containing protein 1	S1154	17%	0.17	23	0	0	0	0	0	0	0	0	0	2	2	0	4
2859	RAPH1_HUMAN	Ras-associated and pleckstrin homology domains-containing protein 1	S17	100%	1.00	7	0	0	0	1	0	0	0	0	0	0	0	0	1
2860	RAPH1_HUMAN	Ras-associated and pleckstrin homology domains-containing protein 1	S5	100%	1.00	7	0	0	0	1	0	0	0	0	0	0	0	0	1
2861	RAPH1_HUMAN	Ras-associated and pleckstrin homology domains-containing protein 1	S894	100%	1.00	17	0	0	0	0	1	0	0	0	0	1	1	1	4
2862	RAPH1_HUMAN	Ras-associated and pleckstrin homology domains-containing protein 1	T1153	86%	0.86	8	0	0	0	0	0	0	0	0	0	0	0	1	1
2863	RADIL_HUMAN	Ras-associating and dilute domain-containing protein	S10	99%	0.99	1	0	0	0	0	0	0	0	0	0	0	1	0	1
2864	RADIL_HUMAN	Ras-associating and dilute domain-containing protein	T13	98%	0.98	1	0	0	0	0	0	0	0	0	0	0	1	0	1
2865	RAB6A_HUMAN	Ras-related protein Rab-6A	S2	50%	0.50	4	0	0	0	0	0	0	0	0	0	2	0	0	2
2866	RAB7L_HUMAN	Ras-related protein Rab-7L1	S183	61%	0.61	1	0	0	0	0	0	0	0	0	0	0	1	0	1
2867	RAB7L_HUMAN	Ras-related protein Rab-7L1	S185	66%	0.66	1	0	0	0	0	0	0	0	0	0	0	1	0	1

2868	RAB7L_HUMAN	Ras-related protein Rab-7L1	T186	60%	0.60	1	0	0	0	0	0	0	0	0	0	0	1	0	1
2869	RRAS2_HUMAN	Ras-related protein R-Ras2	S186	100%	1.00	10	0	1	0	0	1	1	0	0	0	1	1	0	5
2870	RRAS2_HUMAN	Ras-related protein R-Ras2	T190	3%	0.03	5	0	0	0	0	0	1	0	1	0	0	0	0	2
2871	RREB1_HUMAN	Ras-responsive element-binding protein 1	S1167	100%	1.00	1	0	0	0	0	1	0	0	0	0	0	0	0	1
2872	RREB1_HUMAN	Ras-responsive element-binding protein 1	S22	100%	1.00	1	0	0	1	0	0	0	0	0	0	0	0	0	1
2873	RREB1_HUMAN	Ras-responsive element-binding protein 1	S26	100%	1.00	1	0	0	1	0	0	0	0	0	0	0	0	0	1
2874	RREB1_HUMAN	Ras-responsive element-binding protein 1	T19	69%	0.69	1	0	0	1	0	0	0	0	0	0	0	0	0	1
2875	RGPS2_HUMAN	Ras-specific guanine nucleotide-releasing factor RalGPS2	S329	100%	1.00	2	0	0	0	0	1	0	0	0	0	0	1	0	2
2876	RCAS1_HUMAN	Receptor-binding cancer antigen expressed on SiSo cells	S36	100%	1.00	37	1	2	0	0	4	0	0	1	0	0	0	0	8
2877	RPRD2_HUMAN	Regulation of nuclear pre-mRNA domain-containing protein 2	S1099	100%	1.00	15	0	0	0	0	0	0	0	0	0	1	0	1	2
2878	RPRD2_HUMAN	Regulation of nuclear pre-mRNA domain-containing protein 2	T664	60%	0.60	1	0	0	0	0	1	0	0	0	0	0	0	0	1
2879	RPRD2_HUMAN	Regulation of nuclear pre-mRNA domain-containing protein 2	T723	100%	1.00	20	2	2	1	1	1	1	1	0	0	0	0	0	9
2880	RCC1_HUMAN	Regulator of chromosome condensation	S11	100%	1.00	7	0	1	0	2	0	1	2	0	0	0	0	0	6
2881	RGS6_HUMAN	Regulator of G-protein signaling 6	T34	100%	1.00	1	0	0	0	0	0	0	0	0	0	0	1	0	1
2882	RMD3_HUMAN	Regulator of microtubule dynamics protein 3	S212	100%	1.00	4	3	0	0	0	0	0	0	0	0	0	0	0	3
2883	RMD3_HUMAN	Regulator of microtubule dynamics protein 3	S221	15%	0.15	1	1	0	0	0	0	0	0	0	0	0	0	0	1

2884	RMD3_HUMAN	Regulator of microtubule dynamics protein 3	S224	15%	0.15	1	0	0	0	0	0	0	0	1	0	0	0	0	1
2885	RMD3_HUMAN	Regulator of microtubule dynamics protein 3	S225	19%	0.19	2	1	0	0	0	0	0	0	0	0	0	0	0	1
2886	REN3A_HUMAN	Regulator of nonsense transcripts 3A	S192	25%	0.25	2	0	0	0	0	0	0	0	1	0	0	0	0	1
2887	RTEL1_HUMAN	Regulator of telomere elongation helicase 1	S804	17%	0.17	1	0	0	0	0	0	1	0	0	0	0	0	0	1
2888	RPTOR_HUMAN	Regulatory-associated protein of mTOR	S520	100%	1.00	1	1	0	0	0	0	0	0	0	0	0	0	0	1
2889	RPTOR_HUMAN	Regulatory-associated protein of mTOR	S859	1%	0.01	2	0	0	0	0	0	1	0	0	0	0	0	0	1
2890	RPTOR_HUMAN	Regulatory-associated protein of mTOR	S863	100%	1.00	103	2	4	7	5	5	6	6	5	5	2	0	0	47
2891	RPTOR_HUMAN	Regulatory-associated protein of mTOR	S877	59%	0.59	1	1	0	0	0	0	0	0	0	0	0	0	0	1
2892	RPTOR_HUMAN	Regulatory-associated protein of mTOR	T865	14%	0.14	6	1	0	0	0	1	0	1	0	1	0	0	0	4
2893	RPTOR_HUMAN	Regulatory-associated protein of mTOR	Y517	4%	0.04	1	1	0	0	0	0	0	0	0	0	0	0	0	1
2894	IASPP_HUMAN	RelA-associated inhibitor	S113	35%	0.35	1	0	0	0	0	0	0	0	0	0	0	1	0	1
2895	IASPP_HUMAN	RelA-associated inhibitor	S187	100%	1.00	19	0	0	0	2	0	0	0	0	0	0	1	3	6
2896	IASPP_HUMAN	RelA-associated inhibitor	S357	50%	0.50	2	0	0	0	0	0	0	0	0	0	1	0	0	1
2897	IASPP_HUMAN	RelA-associated inhibitor	S358	100%	1.00	17	0	0	0	0	0	0	0	0	0	1	2	2	5
2898	IASPP_HUMAN	RelA-associated inhibitor	S526	100%	1.00	13	3	2	1	1	1	2	2	1	0	0	0	0	13
2899	IASPP_HUMAN	RelA-associated inhibitor	S567	100%	1.00	85	0	0	1	0	2	2	0	0	1	3	4	4	17
2900	IASPP_HUMAN	RelA-associated inhibitor	T570	71%	0.71	24	0	0	0	0	0	1	0	0	1	2	2	1	7
2901	RSF1_HUMAN	Remodeling and spacing factor 1	S1345	100%	1.00	240	4	7	7	4	6	6	6	6	6	4	4	2	62

2902	RSF1_HUMAN	Remodeling and spacing factor 1	S604	100%	1.00	70	1	3	4	3	1	1	1	1	3	0	0	1	19
2903	RFA3_HUMAN	Replication protein A 14 kDa subunit	S44	100%	1.00	26	0	1	0	0	0	0	1	0	1	0	0	0	3
2904	RFA3_HUMAN	Replication protein A 14 kDa subunit	T52	100%	1.00	26	0	1	0	0	0	0	1	0	1	0	0	0	3
2905	RFA2_HUMAN	Replication protein A 32 kDa subunit	S33	14%	0.14	1	0	0	0	0	0	0	1	0	0	0	0	0	1
2906	RFA2_HUMAN	Replication protein A 32 kDa subunit	S4	20%	0.20	1	0	0	0	0	0	0	1	0	0	0	0	0	1
2907	RCOR1_HUMAN	REST corepressor 1	S257	100%	1.00	3	0	0	0	0	0	0	0	0	1	0	0	0	1
2908	RTN3_HUMAN	Reticulon-3	S30	100%	1.00	2	2	0	0	0	0	0	0	0	0	0	0	0	2
2909	RTN4_HUMAN	Reticulon-4	S11	63%	0.63	7	0	0	0	0	0	0	1	0	0	0	0	0	1
2910	RTN4_HUMAN	Reticulon-4	S12	57%	0.57	58	0	2	1	1	0	2	1	1	0	2	1	0	11
2911	RTN4_HUMAN	Reticulon-4	S13	60%	0.60	80	2	2	1	1	1	1	2	0	0	1	0	0	11
2912	RTN4_HUMAN	Reticulon-4	S15	100%	1.00	####	55	52	37	59	50	51	58	59	47	58	49	37	612
2913	RTN4_HUMAN	Reticulon-4	S239	96%	0.96	6	0	0	0	0	0	0	0	0	0	3	2	0	5
2914	RTN4_HUMAN	Reticulon-4	S775	83%	0.83	1	0	0	0	0	0	0	0	0	1	0	0	0	1
2915	RTN4_HUMAN	Reticulon-4	T763	81%	0.81	1	0	0	0	0	0	0	0	0	1	0	0	0	1
2916	RTN4_HUMAN	Reticulon-4	T772	33%	0.33	1	0	0	0	0	0	0	0	0	1	0	0	0	1
2917	RTN4_HUMAN	Reticulon-4	T774	89%	0.89	1	0	0	0	0	0	0	0	0	1	0	0	0	1
2918	RB_HUMAN	Retinoblastoma-associated protein	S37	100%	1.00	164	6	5	2	5	4	2	1	1	2	6	4	3	41
2919	RBL1_HUMAN	Retinoblastoma-like protein 1	S640	100%	1.00	6	0	0	0	1	1	0	0	0	0	0	0	0	2
2920	TIG3_HUMAN	Retinoic acid receptor responder protein 3	S79	68%	0.68	1	0	0	0	0	0	1	0	0	0	0	0	0	1

2921	RAI1_HUMAN	Retinoic acid-induced protein 1	S1374	100%	1.00	3	0	0	0	1	0	0	0	0	0	1	0	0	2
2922	RAI1_HUMAN	Retinoic acid-induced protein 1	T1068	100%	1.00	8	0	1	0	0	0	0	0	0	0	0	0	0	1
2923	RGAG1_HUMAN	Retrotransposon gag domain-containing protein 1	S650	15%	0.15	1	0	0	0	0	1	0	0	0	0	0	0	0	1
2924	RGAG1_HUMAN	Retrotransposon gag domain-containing protein 1	S654	94%	0.94	1	0	0	0	0	1	0	0	0	0	0	0	0	1
2925	RGAG1_HUMAN	Retrotransposon gag domain-containing protein 1	T659	78%	0.78	1	0	0	0	0	1	0	0	0	0	0	0	0	1
2926	GDIR3_HUMAN	Rho GDP-dissociation inhibitor 3	S100	99%	0.99	1	0	1	0	0	0	0	0	0	0	0	0	0	1
2927	GDIR3_HUMAN	Rho GDP-dissociation inhibitor 3	T97	98%	0.98	1	0	1	0	0	0	0	0	0	0	0	0	0	1
2928	RHG01_HUMAN	Rho GTPase-activating protein 1	S17	95%	0.95	8	1	0	1	0	0	0	0	0	0	0	0	0	2
2929	RHG01_HUMAN	Rho GTPase-activating protein 1	S27	58%	0.58	6	1	0	1	0	0	0	0	0	0	0	0	0	2
2930	RHG12_HUMAN	Rho GTPase-activating protein 12	S176	100%	1.00	13	0	0	0	0	0	0	0	0	0	0	2	2	4
2931	RHG17_HUMAN	Rho GTPase-activating protein 17	S575	95%	0.95	2	1	0	0	0	0	0	0	0	0	0	0	0	1
2932	RHG20_HUMAN	Rho GTPase-activating protein 20	S912	86%	0.86	1	0	1	0	0	0	0	0	0	0	0	0	0	1
2933	RHG20_HUMAN	Rho GTPase-activating protein 20	T919	60%	0.60	1	0	1	0	0	0	0	0	0	0	0	0	0	1
2934	RHG29_HUMAN	Rho GTPase-activating protein 29	S1019	100%	1.00	11	0	0	0	0	0	0	0	0	0	2	3	0	5
2935	RHG29_HUMAN	Rho GTPase-activating protein 29	S1029	100%	1.00	20	0	0	0	0	0	0	0	0	0	3	0	2	5
2936	RHG29_HUMAN	Rho GTPase-activating protein 29	S1185	99%	0.99	54	0	0	0	0	0	0	0	0	0	3	3	4	10
2937	RHG29_HUMAN	Rho GTPase-activating protein 29	S1187	48%	0.48	8	0	0	0	0	0	0	0	0	0	2	0	0	2
2938	RHG29_HUMAN	Rho GTPase-activating protein 29	S190	100%	1.00	39	0	0	0	0	0	0	0	0	0	2	4	5	11
2939	RHG29_HUMAN	Rho GTPase-activating protein 29	S499	100%	1.00	29	0	0	0	0	0	0	1	0	0	6	2	3	12

2940	RHG29_HUMAN	Rho GTPase-activating protein 29	S521	100%	1.00	1	0	0	0	0	0	0	0	0	0	0	0	1	1
2941	RHG29_HUMAN	Rho GTPase-activating protein 29	S559	86%	0.86	5	0	0	0	0	0	0	0	0	0	0	1	0	1
2942	RHG29_HUMAN	Rho GTPase-activating protein 29	S913	100%	1.00	125	0	0	0	0	0	0	0	0	0	16	16	2	34
2943	RHG29_HUMAN	Rho GTPase-activating protein 29	S941	25%	0.25	1	0	0	0	0	0	0	0	0	1	0	0	0	1
2944	RHG31_HUMAN	Rho GTPase-activating protein 31	S457	32%	0.32	1	0	0	0	0	0	0	0	0	0	1	0	0	1
2945	RHG31_HUMAN	Rho GTPase-activating protein 31	S460	54%	0.54	1	0	0	0	0	0	0	0	0	0	1	0	0	1
2946	RHG31_HUMAN	Rho GTPase-activating protein 31	S476	34%	0.34	1	0	0	0	0	0	0	0	0	0	1	0	0	1
2947	RHG31_HUMAN	Rho GTPase-activating protein 31	T456	32%	0.32	1	0	0	0	0	0	0	0	0	0	1	0	0	1
2948	RHG32_HUMAN	Rho GTPase-activating protein 32	S871	100%	1.00	39	0	0	0	0	0	0	1	0	0	4	4	3	12
2949	RHG32_HUMAN	Rho GTPase-activating protein 32	T873	25%	0.25	1	0	0	0	0	0	0	0	0	0	1	0	0	1
2950	RHG35_HUMAN	Rho GTPase-activating protein 35	S970	100%	1.00	5	0	0	2	0	2	0	0	0	0	0	0	0	4
2951	ARHG1_HUMAN	Rho guanine nucleotide exchange factor 1	S863	100%	1.00	5	0	0	0	0	0	0	0	0	0	2	1	0	3
2952	ARHGG_HUMAN	Rho guanine nucleotide exchange factor 16	S578	25%	0.25	1	0	0	0	0	0	0	0	0	0	0	1	0	1
2953	ARHGH_HUMAN	Rho guanine nucleotide exchange factor 17	S463	17%	0.17	3	1	0	0	0	0	0	0	0	0	0	0	0	1
2954	ARHG2_HUMAN	Rho guanine nucleotide exchange factor 2	S149	20%	0.20	6	1	0	0	0	0	0	0	0	0	0	0	0	1
2955	ARHG2_HUMAN	Rho guanine nucleotide exchange factor 2	S151	89%	0.89	106	9	10	2	3	4	1	3	2	0	8	4	1	47
2956	ARHG2_HUMAN	Rho guanine nucleotide exchange factor 2	S174	100%	1.00	116	5	6	5	0	3	1	0	0	0	10	6	8	44
2957	ARHG2_HUMAN	Rho guanine nucleotide exchange factor 2	S691	96%	0.96	73	0	1	0	5	2	0	1	0	1	3	1	2	16
2958	ARHG2_HUMAN	Rho guanine nucleotide exchange factor 2	S696	100%	1.00	162	1	2	3	4	5	3	1	0	3	3	3	3	31

2959	ARHG2_HUMAN	Rho guanine nucleotide exchange factor 2	S886	100%	1.00	297	5	12	6	3	5	8	7	3	8	4	1	2	64
2960	ARHG2_HUMAN	Rho guanine nucleotide exchange factor 2	S956	64%	0.64	3	0	0	0	0	0	0	0	0	0	0	1	0	1
2961	ARHG2_HUMAN	Rho guanine nucleotide exchange factor 2	S960	74%	0.74	3	0	0	0	0	0	0	0	0	0	0	1	0	1
2962	ARHG2_HUMAN	Rho guanine nucleotide exchange factor 2	T153	20%	0.20	3	1	0	0	0	0	0	0	0	0	0	0	0	1
2963	ARHG2_HUMAN	Rho guanine nucleotide exchange factor 2	T679	100%	1.00	42	0	0	0	0	0	0	0	0	0	2	1	1	4
2964	ARHG2_HUMAN	Rho guanine nucleotide exchange factor 2	T695	63%	0.63	58	0	0	0	0	0	1	0	0	0	0	1	0	2
2965	ARHG5_HUMAN	Rho guanine nucleotide exchange factor 5	S11	100%	1.00	42	0	2	0	1	3	0	0	1	0	1	1	1	10
2966	ARHG5_HUMAN	Rho guanine nucleotide exchange factor 5	S1126	99%	0.99	100	2	3	3	3	2	1	3	0	3	2	2	4	28
2967	ARHG5_HUMAN	Rho guanine nucleotide exchange factor 5	S450	100%	1.00	42	0	0	0	2	2	0	0	0	0	0	0	0	4
2968	ARHG6_HUMAN	Rho guanine nucleotide exchange factor 6	S769	9%	0.09	1	0	0	0	0	1	0	0	0	0	0	0	0	1
2969	ARHG6_HUMAN	Rho guanine nucleotide exchange factor 6	S770	99%	0.99	1	0	0	0	0	1	0	0	0	0	0	0	0	1
2970	ARHG6_HUMAN	Rho guanine nucleotide exchange factor 6	S773	100%	1.00	1	0	0	0	0	1	0	0	0	0	0	0	0	1
2971	ARHG6_HUMAN	Rho guanine nucleotide exchange factor 6	T772	98%	0.98	1	0	0	0	0	1	0	0	0	0	0	0	0	1
2972	ARHG7_HUMAN	Rho guanine nucleotide exchange factor 7	S123	99%	0.99	3	0	0	0	0	0	0	0	0	1	0	1	0	2
2973	ARHG7_HUMAN	Rho guanine nucleotide exchange factor 7	S124	90%	0.90	3	0	0	0	0	0	0	0	0	1	0	1	0	2
2974	ARHG7_HUMAN	Rho guanine nucleotide exchange factor 7	S703	100%	1.00	1	0	0	0	0	0	1	0	0	0	0	0	0	1
2975	RTKN_HUMAN	Rhotekin	S520	100%	1.00	123	15	6	5	1	7	9	5	3	1	6	7	1	66

2976	RTKN_HUMAN	Rhotekin	T518	92%	0.92	7	0	1	0	0	0	0	0	1	0	0	0	0	2
2977	POP1_HUMAN	Ribonucleases P/MRP protein subunit POP1	S730	99%	0.99	13	0	1	0	0	0	0	0	0	0	0	0	0	1
2978	RAVR1_HUMAN	Ribonucleoprotein PTB-binding 1	S14	100%	1.00	955	23	20	25	18	21	19	22	22	23	20	19	19	251
2979	RAVR1_HUMAN	Ribonucleoprotein PTB-binding 1	T463	100%	1.00	62	1	4	2	0	0	1	0	0	0	2	0	1	11
2980	RIR2_HUMAN	Ribonucleoside-diphosphate reductase subunit M2	S20	100%	1.00	982	12	9	13	17	13	14	8	10	9	16	21	18	160
2981	RIR2_HUMAN	Ribonucleoside-diphosphate reductase subunit M2	S3	100%	1.00	27	0	0	0	0	1	1	0	1	0	0	2	0	5
2982	RL1D1_HUMAN	Ribosomal L1 domain-containing protein 1	S361	100%	1.00	510	8	10	9	12	9	10	8	9	11	8	7	4	105
2983	RL1D1_HUMAN	Ribosomal L1 domain-containing protein 1	T358	85%	0.85	33	0	2	1	1	1	0	1	0	0	0	0	1	7
2984	KS6A4_HUMAN	Ribosomal protein S6 kinase alpha-4	S347	100%	1.00	6	0	0	0	2	0	0	0	0	0	0	0	0	2
2985	RRP1B_HUMAN	Ribosomal RNA processing protein 1 homolog B	S245	100%	1.00	64	5	7	4	5	2	4	0	0	0	1	0	0	28
2986	RRP1B_HUMAN	Ribosomal RNA processing protein 1 homolog B	S458	52%	0.52	4	3	0	0	0	1	0	0	0	0	0	0	0	4
2987	BOP1_HUMAN	Ribosome biogenesis protein BOP1	S126	100%	1.00	50	0	0	0	0	2	0	0	0	1	0	0	0	3
2988	BOP1_HUMAN	Ribosome biogenesis protein BOP1	S127	100%	1.00	50	0	0	0	0	2	0	0	0	1	0	0	0	3
2989	RIM3A_HUMAN	RIMS-binding protein 3A	S1104	98%	0.98	1	0	0	1	0	0	0	0	0	0	0	0	0	1
2990	RIM3A_HUMAN	RIMS-binding protein 3A	S1111	90%	0.90	1	0	0	1	0	0	0	0	0	0	0	0	0	1
2991	RIM3A_HUMAN	RIMS-binding protein 3A	T1100	95%	0.95	1	0	0	1	0	0	0	0	0	0	0	0	0	1
2992	RIM3A_HUMAN	RIMS-binding protein 3A	T1106	100%	1.00	1	0	0	1	0	0	0	0	0	0	0	0	0	1
2993	RIM3A_HUMAN	RIMS-binding protein 3A	Y1105	98%	0.98	1	0	0	1	0	0	0	0	0	0	0	0	0	1
2994	RIM3B_HUMAN	RIMS-binding protein 3B	S1104	98%	0.98	1	0	0	1	0	0	0	0	0	0	0	0	0	1

2995	RIM3B_HUMAN	RIMS-binding protein 3B	S1111	90%	0.90	1	0	0	1	0	0	0	0	0	0	0	0	0	1
2996	RIM3B_HUMAN	RIMS-binding protein 3B	T1100	95%	0.95	1	0	0	1	0	0	0	0	0	0	0	0	0	1
2997	RIM3B_HUMAN	RIMS-binding protein 3B	T1106	100%	1.00	1	0	0	1	0	0	0	0	0	0	0	0	0	1
2998	RIM3B_HUMAN	RIMS-binding protein 3B	Y1105	98%	0.98	1	0	0	1	0	0	0	0	0	0	0	0	0	1
2999	RIM3C_HUMAN	RIMS-binding protein 3C	S1104	98%	0.98	1	0	0	1	0	0	0	0	0	0	0	0	0	1
3000	RIM3C_HUMAN	RIMS-binding protein 3C	S1111	90%	0.90	1	0	0	1	0	0	0	0	0	0	0	0	0	1
3001	RIM3C_HUMAN	RIMS-binding protein 3C	T1100	95%	0.95	1	0	0	1	0	0	0	0	0	0	0	0	0	1
3002	RIM3C_HUMAN	RIMS-binding protein 3C	T1106	100%	1.00	1	0	0	1	0	0	0	0	0	0	0	0	0	1
3003	RIM3C_HUMAN	RIMS-binding protein 3C	Y1105	98%	0.98	1	0	0	1	0	0	0	0	0	0	0	0	0	1
3004	R113A_HUMAN	RING finger protein 113A	S6	100%	1.00	158	2	0	1	0	1	0	0	2	0	4	0	0	10
3005	RN148_HUMAN	RING finger protein 148	S15	90%	0.90	1	1	0	0	0	0	0	0	0	0	0	0	0	1
3006	RN148_HUMAN	RING finger protein 148	S16	86%	0.86	1	1	0	0	0	0	0	0	0	0	0	0	0	1
3007	RN148_HUMAN	RING finger protein 148	S2	48%	0.48	1	1	0	0	0	0	0	0	0	0	0	0	0	1
3008	RN148_HUMAN	RING finger protein 148	S9	70%	0.70	1	1	0	0	0	0	0	0	0	0	0	0	0	1
3009	RN148_HUMAN	RING finger protein 148	T7	48%	0.48	1	1	0	0	0	0	0	0	0	0	0	0	0	1
3010	RN214_HUMAN	RING finger protein 214	S511	99%	0.99	3	1	0	0	0	0	0	0	0	0	0	1	0	2
3011	REXO4_HUMAN	RNA exonuclease 4	S82	100%	1.00	1	0	0	0	0	0	0	0	0	1	0	0	0	1
3012	REXO4_HUMAN	RNA exonuclease 4	S92	100%	1.00	1	0	0	0	0	0	0	0	0	1	0	0	0	1
3013	RPAP1_HUMAN	RNA polymerase II-associated protein 1	S72	100%	1.00	24	0	0	1	0	0	0	0	0	0	4	3	1	9
3014	CTR9_HUMAN	RNA polymerase-associated protein CTR9 homolog	T925	100%	1.00	138	11	11	9	4	9	6	9	2	8	5	1	1	76

3015	RBM10_HUMAN	RNA-binding protein 10	S89	100%	1.00	49	0	2	0	5	2	3	5	4	5	2	1	0	29
3016	RBM14_HUMAN	RNA-binding protein 14	T206	100%	1.00	2	2	0	0	0	0	0	0	0	0	0	0	0	2
3017	RBM25_HUMAN	RNA-binding protein 25	S675	0%	0.00	1	0	0	0	0	0	0	0	0	0	0	1	0	1
3018	RBM25_HUMAN	RNA-binding protein 25	S677	100%	1.00	10	0	0	2	0	0	0	0	0	0	5	1	2	10
3019	RBM5_HUMAN	RNA-binding protein 5	S624	100%	1.00	21	1	0	0	2	0	0	0	0	0	0	0	0	3
3020	RBM6_HUMAN	RNA-binding protein 6	S362	92%	0.92	1	0	0	0	0	0	0	0	0	1	0	0	0	1
3021	RBM8A_HUMAN	RNA-binding protein 8A	S24	100%	1.00	1	0	1	0	0	0	0	0	0	0	0	0	0	1
3022	RALY_HUMAN	RNA-binding protein Raly	S135	100%	1.00	77	2	1	1	0	3	5	3	4	4	6	6	6	41
3023	RC3H1_HUMAN	Roquin	S535	88%	0.88	1	0	0	0	0	0	1	0	0	0	0	0	0	1
3024	RC3H1_HUMAN	Roquin	S772	99%	0.99	1	0	0	0	1	0	0	0	0	0	0	0	0	1
3025	RC3H1_HUMAN	Roquin	S779	43%	0.43	1	0	0	0	0	0	0	0	0	0	0	1	0	1
3026	RSBNL_HUMAN	Round spermatid basic protein 1-like protein	S6	100%	1.00	131	6	6	3	0	3	0	1	2	0	5	1	1	28
3027	UTP23_HUMAN	rRNA-processing protein UTP23 homolog	S219	45%	0.45	1	0	0	1	0	0	0	0	0	0	0	0	0	1
3028	UTP23_HUMAN	rRNA-processing protein UTP23 homolog	S222	65%	0.65	1	0	0	1	0	0	0	0	0	0	0	0	0	1
3029	RRP12_HUMAN	RRP12-like protein	S1080	100%	1.00	29	0	0	0	1	0	2	2	0	0	0	0	0	5
3030	RUVB2_HUMAN	RuvB-like 2	S114	22%	0.22	1	0	0	0	0	0	0	0	1	0	0	0	0	1
3031	RYR1_HUMAN	Ryanodine receptor 1	S125	100%	1.00	1	0	0	0	0	0	0	1	0	0	0	0	0	1
3032	RYR1_HUMAN	Ryanodine receptor 1	S153	100%	1.00	1	0	0	0	0	0	0	1	0	0	0	0	0	1
3033	RYR1_HUMAN	Ryanodine receptor 1	T127	100%	1.00	1	0	0	0	0	0	0	1	0	0	0	0	0	1

3034	RYR1_HUMAN	Ryanodine receptor 1	T141	100%	1.00	1	0	0	0	0	0	0	1	0	0	0	0	0	1
3035	RYR1_HUMAN	Ryanodine receptor 1	T148	100%	1.00	1	0	0	0	0	0	0	1	0	0	0	0	0	1
3036	SLTM_HUMAN	SAFB-like transcription modulator	S553	100%	1.00	72	0	7	7	4	4	0	2	2	6	0	0	6	38
3037	SASH1_HUMAN	SAM and SH3 domain-containing protein 1	S721	100%	1.00	1	0	0	0	0	0	0	0	0	0	0	0	1	1
3038	SASH1_HUMAN	SAM and SH3 domain-containing protein 1	S90	100%	1.00	11	1	2	0	0	0	0	0	0	0	0	0	0	3
3039	SAGE1_HUMAN	Sarcoma antigen 1	S617	22%	0.22	1	1	0	0	0	0	0	0	0	0	0	0	0	1
3040	SAGE1_HUMAN	Sarcoma antigen 1	T618	50%	0.50	1	1	0	0	0	0	0	0	0	0	0	0	0	1
3041	SAFB1_HUMAN	Scaffold attachment factor B1	S344	100%	1.00	21	1	0	2	0	0	0	0	0	0	0	0	0	3
3042	SAFB2_HUMAN	Scaffold attachment factor B2	S343	100%	1.00	2	0	0	2	0	0	0	0	0	0	0	0	0	2
3043	SCEL_HUMAN	Sciellin	S289	100%	1.00	4	0	0	0	0	0	0	0	0	0	1	0	2	3
3044	PTTG1_HUMAN	Securin	S165	100%	1.00	5	0	0	0	0	0	0	0	0	0	1	1	2	4
3045	PTTG2_HUMAN	Securin-2	S165	100%	1.00	5	0	0	0	0	0	0	0	0	0	1	1	2	4
3046	SPS2_HUMAN	Selenide, water dikinase 2	S180	91%	0.91	1	0	0	1	0	0	0	0	0	0	0	0	0	1
3047	SPS2_HUMAN	Selenide, water dikinase 2	S182	83%	0.83	1	0	0	1	0	0	0	0	0	0	0	0	0	1
3048	SPS2_HUMAN	Selenide, water dikinase 2	S184	45%	0.45	1	0	0	1	0	0	0	0	0	0	0	0	0	1
3049	SPS2_HUMAN	Selenide, water dikinase 2	Y163	45%	0.45	1	0	0	1	0	0	0	0	0	0	0	0	0	1
3050	SELV_HUMAN	Selenoprotein V	S12	100%	1.00	1	0	1	0	0	0	0	0	0	0	0	0	0	1
3051	SELV_HUMAN	Selenoprotein V	S16	100%	1.00	1	0	1	0	0	0	0	0	0	0	0	0	0	1
3052	SELV_HUMAN	Selenoprotein V	S18	100%	1.00	1	0	1	0	0	0	0	0	0	0	0	0	0	1
3053	SELV_HUMAN	Selenoprotein V	T15	100%	1.00	1	0	1	0	0	0	0	0	0	0	0	0	0	1

3054	SELV_HUMAN	Selenoprotein V	T17	100%	1.00	1	0	1	0	0	0	0	0	0	0	0	0	0	1
3055	SELV_HUMAN	Selenoprotein V	T7	87%	0.87	1	0	1	0	0	0	0	0	0	0	0	0	0	1
3056	SENP3_HUMAN	Sentrin-specific protease 3	S212	100%	1.00	3	0	0	0	1	0	0	0	2	0	0	0	0	3
3057	SENP3_HUMAN	Sentrin-specific protease 3	S232	83%	0.83	5	0	0	0	0	0	0	0	0	0	0	1	0	1
3058	SEP11_HUMAN	Septin-11	S9	100%	1.00	24	0	0	0	2	1	1	0	2	1	0	0	1	8
3059	SEPT2_HUMAN	Septin-2	S218	100%	1.00	####	31	29	22	19	21	24	28	20	23	24	21	19	281
3060	SEPT9_HUMAN	Septin-9	S30	100%	1.00	255	13	8	10	3	3	3	14	10	6	4	4	6	84
3061	SQSTM_HUMAN	Sequestosome-1	S24	96%	0.96	1	0	0	0	0	0	0	0	1	0	0	0	0	1
3062	SQSTM_HUMAN	Sequestosome-1	S272	99%	0.99	14	0	0	0	0	0	0	0	0	0	3	3	3	9
3063	SQSTM_HUMAN	Sequestosome-1	S365	76%	0.76	20	0	0	0	0	0	0	1	0	0	1	0	2	4
3064	SQSTM_HUMAN	Sequestosome-1	S366	81%	0.81	157	1	0	0	2	0	0	0	2	2	1	3	1	12
3065	SQSTM_HUMAN	Sequestosome-1	S370	25%	0.25	9	1	0	0	0	0	0	0	0	0	0	0	0	1
3066	SRRM1_HUMAN	Serine/arginine repetitive matrix protein 1	S260	100%	1.00	####	31	29	22	25	24	26	26	20	20	35	24	20	302
3067	SRRM1_HUMAN	Serine/arginine repetitive matrix protein 1	S874	100%	1.00	552	16	11	11	11	12	9	12	11	9	9	10	9	130
3068	SRRM1_HUMAN	Serine/arginine repetitive matrix protein 1	T220	100%	1.00	39	0	3	2	0	0	1	2	0	2	0	1	0	11
3069	SRRM1_HUMAN	Serine/arginine repetitive matrix protein 1	T846	100%	1.00	12	0	0	2	0	1	0	0	0	0	1	1	0	5
3070	SRRM1_HUMAN	Serine/arginine repetitive matrix protein 1	T856	25%	0.25	3	0	1	0	0	0	0	0	0	0	0	0	0	1
3071	SRRM1_HUMAN	Serine/arginine repetitive matrix protein 1	T872	100%	1.00	38	0	2	0	0	1	0	0	0	0	0	0	0	3
3072	SRRM2_HUMAN	Serine/arginine repetitive matrix protein 2	S1124	100%	1.00	219	6	12	12	7	8	6	9	9	6	8	9	8	100

3073	SRRM2_HUMAN	Serine/arginine repetitive matrix protein 2	S1179	100%	1.00	713	29	36	36	15	22	14	14	11	2	39	38	39	295
3074	SRRM2_HUMAN	Serine/arginine repetitive matrix protein 2	S1188	100%	1.00	17	0	0	0	0	0	0	0	0	1	2	1	1	5
3075	SRRM2_HUMAN	Serine/arginine repetitive matrix protein 2	S1318	75%	0.75	12	1	1	1	0	1	0	0	0	0	0	0	0	4
3076	SRRM2_HUMAN	Serine/arginine repetitive matrix protein 2	S1320	100%	1.00	271	9	8	9	7	7	7	7	7	7	4	8	4	84
3077	SRRM2_HUMAN	Serine/arginine repetitive matrix protein 2	S1329	100%	1.00	24	3	1	2	0	1	0	0	0	1	0	0	0	8
3078	SRRM2_HUMAN	Serine/arginine repetitive matrix protein 2	S1378	93%	0.93	49	0	1	1	1	1	0	0	0	1	0	0	1	6
3079	SRRM2_HUMAN	Serine/arginine repetitive matrix protein 2	S1379	68%	0.68	49	0	1	1	1	1	0	0	0	1	0	0	1	6
3080	SRRM2_HUMAN	Serine/arginine repetitive matrix protein 2	S1382	87%	0.87	49	0	1	1	1	1	0	0	0	1	0	0	1	6
3081	SRRM2_HUMAN	Serine/arginine repetitive matrix protein 2	S1383	74%	0.74	48	0	1	1	1	1	0	0	0	1	0	0	1	6
3082	SRRM2_HUMAN	Serine/arginine repetitive matrix protein 2	S1384	70%	0.70	48	0	1	1	1	1	0	0	0	1	0	0	1	6
3083	SRRM2_HUMAN	Serine/arginine repetitive matrix protein 2	S1404	100%	1.00	120	0	2	2	3	2	0	0	0	2	0	0	2	13
3084	SRRM2_HUMAN	Serine/arginine repetitive matrix protein 2	S2132	100%	1.00	47	0	0	2	0	0	0	0	2	0	2	0	2	8
3085	SRRM2_HUMAN	Serine/arginine repetitive matrix protein 2	S2272	100%	1.00	5	0	0	0	0	0	0	0	0	0	0	0	1	1
3086	SRRM2_HUMAN	Serine/arginine repetitive matrix protein 2	S2382	100%	1.00	40	0	0	0	0	0	0	0	0	0	4	5	3	12
3087	SRRM2_HUMAN	Serine/arginine repetitive matrix protein 2	S2449	99%	0.99	326	11	11	12	5	7	7	11	10	6	5	7	8	100
3088	SRRM2_HUMAN	Serine/arginine repetitive matrix protein 2	S2453	64%	0.64	70	1	2	2	3	0	1	1	1	3	3	1	2	20
3089	SRRM2_HUMAN	Serine/arginine repetitive matrix protein 2	S2456	26%	0.26	8	1	1	1	0	0	0	0	0	0	0	0	0	3

3090	SRRM2_HUMAN	Serine/arginine repetitive matrix protein 2	S2581	100%	1.00	22	1	1	0	2	2	1	1	6	8	0	0	0	22
3091	SRRM2_HUMAN	Serine/arginine repetitive matrix protein 2	S317	86%	0.86	11	0	1	0	0	0	0	0	0	0	0	0	0	1
3092	SRRM2_HUMAN	Serine/arginine repetitive matrix protein 2	S322	96%	0.96	100	5	0	3	1	2	2	5	1	1	1	2	2	25
3093	SRRM2_HUMAN	Serine/arginine repetitive matrix protein 2	S323	99%	0.99	338	12	5	5	9	6	6	8	7	9	8	6	7	88
3094	SRRM2_HUMAN	Serine/arginine repetitive matrix protein 2	S351	18%	0.18	1	0	0	0	0	0	0	0	0	1	0	0	0	1
3095	SRRM2_HUMAN	Serine/arginine repetitive matrix protein 2	S353	70%	0.70	1	0	0	0	0	0	0	0	0	1	0	0	0	1
3096	SRRM2_HUMAN	Serine/arginine repetitive matrix protein 2	S377	100%	1.00	238	7	6	4	8	4	5	6	3	4	3	4	3	57
3097	SRRM2_HUMAN	Serine/arginine repetitive matrix protein 2	S395	43%	0.43	5	0	0	1	0	0	0	0	0	0	0	0	0	1
3098	SRRM2_HUMAN	Serine/arginine repetitive matrix protein 2	S398	100%	1.00	93	2	0	3	4	1	4	1	2	1	0	0	1	19
3099	SRRM2_HUMAN	Serine/arginine repetitive matrix protein 2	S876	100%	1.00	64	5	4	2	1	1	2	3	3	3	2	1	2	29
3100	SRRM2_HUMAN	Serine/arginine repetitive matrix protein 2	T1177	100%	1.00	26	0	1	2	3	1	0	0	0	2	1	1	1	12
3101	SRRM2_HUMAN	Serine/arginine repetitive matrix protein 2	T2583	50%	0.50	1	0	0	0	0	0	0	0	0	0	0	0	1	1
3102	SRRM2_HUMAN	Serine/arginine repetitive matrix protein 2	T315	6%	0.06	1	0	0	0	0	0	0	0	0	0	0	1	0	1
3103	SRRM2_HUMAN	Serine/arginine repetitive matrix protein 2	T318	79%	0.79	13	1	0	0	0	0	0	1	0	1	1	0	0	4
3104	SRRM2_HUMAN	Serine/arginine repetitive matrix protein 2	T326	56%	0.56	68	2	1	2	3	4	3	1	2	2	3	2	1	26
3105	SRRM2_HUMAN	Serine/arginine repetitive matrix protein 2	T328	14%	0.14	41	0	0	2	0	0	0	3	0	0	0	2	1	8
3106	SRRM2_HUMAN	Serine/arginine repetitive matrix protein 2	T400	95%	0.95	249	7	6	4	8	4	5	6	3	6	3	4	3	59

3107	SRS11_HUMAN	Serine/arginine-rich splicing factor 11	S207	100%	1.00	75	0	0	0	0	0	0	0	0	0	6	6	6	18
3108	SRS11_HUMAN	Serine/arginine-rich splicing factor 11	S449	100%	1.00	2	0	0	0	0	0	1	0	0	1	0	0	0	2
3109	SRSF2_HUMAN	Serine/arginine-rich splicing factor 2	S2	25%	0.25	2	1	0	0	0	0	0	1	0	0	0	0	0	2
3110	SRSF2_HUMAN	Serine/arginine-rich splicing factor 2	S26	100%	1.00	13	1	2	0	0	0	0	0	0	0	3	3	0	9
3111	SRSF9_HUMAN	Serine/arginine-rich splicing factor 9	S211	100%	1.00	30	4	4	0	0	0	0	0	0	0	0	0	3	11
3112	STK10_HUMAN	Serine/threonine-protein kinase 10	S455	99%	0.99	4	0	0	0	0	0	0	0	0	0	1	0	0	1
3113	ST17A_HUMAN	Serine/threonine-protein kinase 17A	S28	100%	1.00	6	0	0	0	1	0	0	0	0	0	2	1	2	6
3114	ARAF_HUMAN	Serine/threonine-protein kinase A-Raf	T215	8%	0.08	1	1	0	0	0	0	0	0	0	0	0	0	0	1
3115	ATR_HUMAN	Serine/threonine-protein kinase ATR	S2186	17%	0.17	1	0	0	0	0	0	0	0	0	1	0	0	0	1
3116	BRAF_HUMAN	Serine/threonine-protein kinase B-raf	S365	25%	0.25	2	0	0	1	0	0	0	0	0	0	0	1	0	2
3117	KPCD1_HUMAN	Serine/threonine-protein kinase D1	S205	99%	0.99	9	0	1	0	0	0	0	0	0	0	0	0	0	1
3118	KPCD3_HUMAN	Serine/threonine-protein kinase D3	S41	81%	0.81	1	0	0	0	0	0	0	0	0	0	0	0	1	1
3119	KPCD3_HUMAN	Serine/threonine-protein kinase D3	S44	72%	0.72	1	0	0	0	0	0	0	0	0	0	0	0	1	1
3120	KPCD3_HUMAN	Serine/threonine-protein kinase D3	S53	98%	0.98	1	0	0	0	0	0	0	0	0	0	0	0	1	1
3121	MARK2_HUMAN	Serine/threonine-protein kinase MARK2	S456	100%	1.00	264	7	9	5	6	8	6	9	7	10	6	8	5	86
3122	MST4_HUMAN	Serine/threonine-protein kinase MST4	S4	100%	1.00	105	0	0	0	0	0	0	0	0	0	11	12	3	26
3123	MTOR_HUMAN	Serine/threonine-protein kinase mTOR	S1584	55%	0.55	1	0	0	1	0	0	0	0	0	0	0	0	0	1
3124	MTOR_HUMAN	Serine/threonine-protein kinase mTOR	S1592	100%	1.00	1	0	0	1	0	0	0	0	0	0	0	0	0	1

3125	MTOR_HUMAN	Serine/threonine-protein kinase mTOR	S1597	99%	0.99	1	0	0	1	0	0	0	0	0	0	0	0	0	1
3126	MTOR_HUMAN	Serine/threonine-protein kinase mTOR	T1576	83%	0.83	1	0	0	1	0	0	0	0	0	0	0	0	0	1
3127	MTOR_HUMAN	Serine/threonine-protein kinase mTOR	Y1583	55%	0.55	1	0	0	1	0	0	0	0	0	0	0	0	0	1
3128	MTOR_HUMAN	Serine/threonine-protein kinase mTOR	Y1605	99%	0.99	1	0	0	1	0	0	0	0	0	0	0	0	0	1
3129	PKN2_HUMAN	Serine/threonine-protein kinase N2	S535	47%	0.47	2	0	0	0	0	0	0	0	0	0	1	1	0	2
3130	PKN2_HUMAN	Serine/threonine-protein kinase N2	S582	65%	0.65	3	0	0	0	0	0	0	0	1	0	0	0	0	1
3131	PKN2_HUMAN	Serine/threonine-protein kinase N2	S583	92%	0.92	175	4	3	4	4	5	4	4	4	3	4	5	4	48
3132	PKN2_HUMAN	Serine/threonine-protein kinase N2	T527	9%	0.09	1	0	0	0	0	0	0	0	0	0	1	0	0	1
3133	PKN2_HUMAN	Serine/threonine-protein kinase N2	T958	99%	0.99	1	0	0	0	0	0	0	0	0	0	1	0	0	1
3134	NEK11_HUMAN	Serine/threonine-protein kinase Nek11	S213	14%	0.14	2	0	0	0	1	0	0	0	0	0	0	0	0	1
3135	PAK2_HUMAN	Serine/threonine-protein kinase PAK 2	S141	100%	1.00	113	5	7	2	0	3	2	1	1	5	7	9	1	43
3136	PAK2_HUMAN	Serine/threonine-protein kinase PAK 2	S2	100%	1.00	302	6	5	8	8	6	4	6	9	9	8	5	7	81
3137	PAK2_HUMAN	Serine/threonine-protein kinase PAK 2	T143	20%	0.20	4	0	0	0	0	0	0	0	0	0	0	1	0	1
3138	PAK4_HUMAN	Serine/threonine-protein kinase PAK 4	S163	100%	1.00	1	0	0	0	0	0	0	0	0	0	1	0	0	1
3139	PAK4_HUMAN	Serine/threonine-protein kinase PAK 4	S181	100%	1.00	273	7	6	5	5	7	8	6	6	8	5	9	6	78
3140	PAK4_HUMAN	Serine/threonine-protein kinase PAK 4	S474	100%	1.00	49	2	6	2	0	0	1	4	4	4	0	4	2	29
3141	PRP4B_HUMAN	Serine/threonine-protein kinase PRP4 homolog	S144	99%	0.99	7	0	0	0	0	0	1	0	0	0	0	0	0	1
3142	PRP4B_HUMAN	Serine/threonine-protein kinase PRP4 homolog	S277	100%	1.00	1	0	0	0	0	0	0	0	0	1	0	0	0	1

3143	PRP4B_HUMAN	Serine/threonine-protein kinase PRP4 homolog	S837	96%	0.96	5	1	0	0	1	0	0	0	0	0	0	0	0	2
3144	PRP4B_HUMAN	Serine/threonine-protein kinase PRP4 homolog	S839	52%	0.52	2	0	0	1	0	0	0	0	0	0	0	0	0	1
3145	PRP4B_HUMAN	Serine/threonine-protein kinase PRP4 homolog	S852	16%	0.16	26	0	0	0	1	0	0	2	0	0	0	0	0	3
3146	PRP4B_HUMAN	Serine/threonine-protein kinase PRP4 homolog	T847	56%	0.56	47	2	1	1	0	1	1	1	1	1	0	0	0	9
3147	PRP4B_HUMAN	Serine/threonine-protein kinase PRP4 homolog	Y140	75%	0.75	3	0	0	0	0	0	1	0	0	0	0	0	0	1
3148	PRP4B_HUMAN	Serine/threonine-protein kinase PRP4 homolog	Y849	100%	1.00	151	5	2	3	3	1	3	5	2	3	3	2	3	35
3149	RIOK1_HUMAN	Serine/threonine-protein kinase RIO1	S21	33%	0.33	8	0	0	1	0	1	0	0	0	0	0	0	0	2
3150	RIOK1_HUMAN	Serine/threonine-protein kinase RIO1	S22	100%	1.00	16	0	0	0	2	0	0	0	0	0	0	0	0	2
3151	SIK1_HUMAN	Serine/threonine-protein kinase SIK1	S644	97%	0.97	1	0	0	1	0	0	0	0	0	0	0	0	0	1
3152	SIK2_HUMAN	Serine/threonine-protein kinase SIK2	S587	93%	0.93	6	0	0	0	0	2	0	0	0	0	0	0	0	2
3153	SIK3_HUMAN	Serine/threonine-protein kinase SIK3	S493	100%	1.00	11	0	3	1	1	0	1	0	1	1	0	1	0	9
3154	TNI3K_HUMAN	Serine/threonine-protein kinase TNNI3K	T314	33%	0.33	1	0	0	0	1	0	0	0	0	0	0	0	0	1
3155	TLK1_HUMAN	Serine/threonine-protein kinase tousled-like 1	S2	100%	1.00	1	0	0	0	0	0	0	0	0	0	1	0	0	1
3156	TLK1_HUMAN	Serine/threonine-protein kinase tousled-like 1	S33	11%	0.11	1	0	0	0	0	0	0	0	0	0	1	0	0	1
3157	TLK1_HUMAN	Serine/threonine-protein kinase tousled-like 1	S41	66%	0.66	1	0	0	0	0	0	0	0	0	0	1	0	0	1
3158	TLK1_HUMAN	Serine/threonine-protein kinase tousled-like 1	S5	20%	0.20	1	0	0	0	0	0	0	0	0	0	1	0	0	1
3159	TLK1_HUMAN	Serine/threonine-protein kinase tousled-like 1	T38	66%	0.66	1	0	0	0	0	0	0	0	0	0	1	0	0	1

3160	TLK2_HUMAN	Serine/threonine-protein kinase tousled-like 2	S307	100%	1.00	1	0	0	0	0	0	0	0	0	1	0	0	0	1
3161	TLK2_HUMAN	Serine/threonine-protein kinase tousled-like 2	T309	100%	1.00	1	0	0	0	0	0	0	0	0	1	0	0	0	1
3162	ULK4_HUMAN	Serine/threonine-protein kinase ULK4	S835	100%	1.00	3	0	0	0	0	0	0	0	1	1	0	1	0	3
3163	ULK4_HUMAN	Serine/threonine-protein kinase ULK4	T607	100%	1.00	1	0	0	0	0	0	0	0	0	0	0	0	1	1
3164	ULK4_HUMAN	Serine/threonine-protein kinase ULK4	Y606	100%	1.00	1	0	0	0	0	0	0	0	0	0	0	0	1	1
3165	WNK1_HUMAN	Serine/threonine-protein kinase WNK1	S1978	100%	1.00	505	9	11	7	16	8	6	8	11	8	28	20	11	143
3166	WNK1_HUMAN	Serine/threonine-protein kinase WNK1	S2002	93%	0.93	1	1	0	0	0	0	0	0	0	0	0	0	0	1
3167	PP1RA_HUMAN	Serine/threonine-protein phosphatase 1 regulatory subunit 10	S313	100%	1.00	397	8	8	8	8	9	10	10	8	8	8	6	5	96
3168	PP1RA_HUMAN	Serine/threonine-protein phosphatase 1 regulatory subunit 10	S591	87%	0.87	14	1	1	0	0	0	0	0	0	0	0	0	0	2
3169	PP1RA_HUMAN	Serine/threonine-protein phosphatase 1 regulatory subunit 10	T315	1%	0.01	13	0	0	1	0	1	1	1	0	0	0	1	0	5
3170	2A5D_HUMAN	Serine/threonine-protein phosphatase 2A 56 kDa regulatory subunit delta isoform	S23	39%	0.39	19	1	0	1	3	1	0	1	0	1	0	0	0	8
3171	2A5D_HUMAN	Serine/threonine-protein phosphatase 2A 56 kDa regulatory subunit delta isoform	S573	100%	1.00	3	0	1	0	0	1	0	0	1	0	0	0	0	3
3172	2A5D_HUMAN	Serine/threonine-protein phosphatase 2A 56 kDa regulatory subunit delta isoform	T18	43%	0.43	5	0	0	0	1	0	0	0	0	0	0	0	1	2

3173	2AAB_HUMAN	Serine/threonine-protein phosphatase 2A 65 kDa regulatory subunit A beta isoform	T9	96%	0.96	3	0	0	0	0	0	1	0	0	1	0	0	1	3
3174	P4R3A_HUMAN	Serine/threonine-protein phosphatase 4 regulatory subunit 3A	S127	43%	0.43	1	1	0	0	0	0	0	0	0	0	0	0	0	1
3175	PPP5_HUMAN	Serine/threonine-protein phosphatase 5	S224	61%	0.61	1	1	0	0	0	0	0	0	0	0	0	0	0	1
3176	PPP5_HUMAN	Serine/threonine-protein phosphatase 5	T225	61%	0.61	1	1	0	0	0	0	0	0	0	0	0	0	0	1
3177	PP6R1_HUMAN	Serine/threonine-protein phosphatase 6 regulatory subunit 1	S759	100%	1.00	18	0	0	0	0	0	0	0	0	0	3	0	0	3
3178	PP6R3_HUMAN	Serine/threonine-protein phosphatase 6 regulatory subunit 3	S617	100%	1.00	14	3	4	0	0	0	0	0	2	0	0	0	0	9
3179	STRAP_HUMAN	Serine-threonine kinase receptor-associated protein	S335	100%	1.00	32	3	0	0	1	1	0	0	0	1	1	0	0	7
3180	STRAP_HUMAN	Serine-threonine kinase receptor-associated protein	S338	100%	1.00	32	3	0	0	1	1	0	0	0	1	1	0	0	7
3181	SDPR_HUMAN	Serum deprivation-response protein	S366	22%	0.22	2	0	0	0	0	0	0	0	1	0	0	0	0	1
3182	SRF_HUMAN	Serum response factor	S224	99%	0.99	5	0	0	1	0	0	0	0	0	0	0	0	0	1
3183	SRFB1_HUMAN	Serum response factor-binding protein 1	S203	58%	0.58	2	0	0	0	0	1	0	0	0	0	0	0	0	1
3184	SH2B3_HUMAN	SH2B adapter protein 3	S150	82%	0.82	1	1	0	0	0	0	0	0	0	0	0	0	0	1
3185	SPD2A_HUMAN	SH3 and PX domain-containing protein 2A	S175	19%	0.19	1	1	0	0	0	0	0	0	0	0	0	0	0	1
3186	SPD2A_HUMAN	SH3 and PX domain-containing protein 2A	Y171	41%	0.41	1	1	0	0	0	0	0	0	0	0	0	0	0	1
3187	SPD2B_HUMAN	SH3 and PX domain-containing protein 2B	S291	98%	0.98	4	0	0	0	0	0	0	0	0	0	0	1	0	1
3188	SPD2B_HUMAN	SH3 and PX domain-containing protein 2B	S293	92%	0.92	4	0	0	0	0	0	0	0	0	0	0	0	1	1

3189	3BP1_HUMAN	SH3 domain-binding protein 1	S550	100%	1.00	30	0	1	1	0	0	1	0	1	0	0	0	0	4
3190	3BP1_HUMAN	SH3 domain-binding protein 1	S612	100%	1.00	1	0	0	0	0	0	0	0	0	0	0	1	0	1
3191	SH3B4_HUMAN	SH3 domain-binding protein 4	S42	99%	0.99	3	0	0	0	0	0	0	0	0	0	1	0	0	1
3192	SH3B4_HUMAN	SH3 domain-binding protein 4	S44	98%	0.98	4	0	0	0	0	0	0	0	0	0	1	0	0	1
3193	3BP5L_HUMAN	SH3 domain-binding protein 5-like	S358	7%	0.07	1	0	0	0	0	0	0	0	0	0	0	0	1	1
3194	3BP5L_HUMAN	SH3 domain-binding protein 5-like	S362	100%	1.00	9	1	0	1	0	1	0	2	1	1	0	0	0	7
3195	SH3K1_HUMAN	SH3 domain-containing kinase-binding protein 1	S587	100%	1.00	7	0	0	0	0	1	0	0	0	0	2	1	2	6
3196	SH3K1_HUMAN	SH3 domain-containing kinase-binding protein 1	T593	0%	0.00	1	0	0	0	0	0	0	0	0	0	1	0	0	1
3197	SHRPN_HUMAN	Sharpin	S165	99%	0.99	2	0	0	0	0	0	0	0	0	2	0	0	0	2
3198	SHC1_HUMAN	SHC-transforming protein 1	S426	50%	0.50	7	0	0	0	1	0	0	0	0	0	0	0	0	1
3199	SHC1_HUMAN	SHC-transforming protein 1	Y427	100%	1.00	14	0	0	0	0	0	1	0	0	0	0	0	0	1
3200	SHOT1_HUMAN	Shootin-1	S372	100%	1.00	10	1	0	0	1	0	0	0	0	0	0	0	0	2
3201	SHOT1_HUMAN	Shootin-1	S375	100%	1.00	10	1	0	0	1	0	0	0	0	0	0	0	0	2
3202	SHOT1_HUMAN	Shootin-1	T496	7%	0.07	1	0	0	0	0	0	1	0	0	0	0	0	0	1
3203	SCOC_HUMAN	Short coiled-coil protein	S43	92%	0.92	1	0	0	0	0	0	0	1	0	0	0	0	0	1
3204	SCOC_HUMAN	Short coiled-coil protein	S48	84%	0.84	1	0	0	0	0	0	0	1	0	0	0	0	0	1
3205	SCOC_HUMAN	Short coiled-coil protein	S51	85%	0.85	1	0	0	0	0	0	0	1	0	0	0	0	0	1
3206	TRPC5_HUMAN	Short transient receptor potential channel 5	S444	98%	0.98	1	0	1	0	0	0	0	0	0	0	0	0	0	1
3207	TRPC5_HUMAN	Short transient receptor potential channel 5	T428	17%	0.17	1	0	1	0	0	0	0	0	0	0	0	0	0	1

3208	TRPC5_HUMAN	Short transient receptor potential channel 5	Y446	17%	0.17	1	0	1	0	0	0	0	0	0	0	0	0	0	1
3209	SKT_HUMAN	Sickle tail protein homolog	S1044	100%	1.00	3	0	0	0	0	0	0	0	0	0	0	1	2	3
3210	SKT_HUMAN	Sickle tail protein homolog	S1461	100%	1.00	31	3	0	2	2	2	1	3	0	2	3	2	0	20
3211	SKT_HUMAN	Sickle tail protein homolog	S357	100%	1.00	6	1	0	0	0	1	1	1	0	0	0	0	0	4
3212	SKT_HUMAN	Sickle tail protein homolog	T1462	1%	0.01	1	0	0	0	0	0	1	0	0	0	0	0	0	1
3213	PSL1_HUMAN	Signal peptide peptidase-like 2B	Y445	97%	0.97	1	0	0	1	0	0	0	0	0	0	0	0	0	1
3214	PSL1_HUMAN	Signal peptide peptidase-like 2B	Y453	100%	1.00	1	0	0	1	0	0	0	0	0	0	0	0	0	1
3215	SRPRB_HUMAN	Signal recognition particle receptor subunit beta	S112	98%	0.98	4	0	0	0	0	0	1	0	0	0	0	0	0	1
3216	STAT3_HUMAN	Signal transducer and activator of transcription 3	S727	100%	1.00	166	0	2	1	8	2	4	5	5	2	6	5	5	45
3217	SI1L1_HUMAN	Signal-induced proliferation-associated 1-like protein 1	S1549	100%	1.00	84	0	6	3	0	5	2	0	4	0	0	0	0	20
3218	SI1L1_HUMAN	Signal-induced proliferation-associated 1-like protein 1	T1551	3%	0.03	5	0	0	1	0	1	0	0	0	0	0	0	0	2
3219	SI1L3_HUMAN	Signal-induced proliferation-associated 1-like protein 3	S146	100%	1.00	1	0	0	0	0	0	0	0	0	1	0	0	0	1
3220	SI1L3_HUMAN	Signal-induced proliferation-associated 1-like protein 3	S960	25%	0.25	2	0	0	0	0	0	0	0	0	0	1	0	0	1
3221	SI1L3_HUMAN	Signal-induced proliferation-associated 1-like protein 3	T959	8%	0.08	1	0	1	0	0	0	0	0	0	0	0	0	0	1
3222	PDS5B_HUMAN	Sister chromatid cohesion protein PDS5 homolog B	S1283	100%	1.00	4	0	1	2	0	0	0	0	0	0	0	0	0	3
3223	SLAI2_HUMAN	SLAIN motif-containing protein 2	S391	98%	0.98	4	1	0	0	0	0	0	1	0	0	0	0	0	2
3224	SLAI2_HUMAN	SLAIN motif-containing protein 2	S87	88%	0.88	26	1	0	0	0	0	1	2	1	0	0	0	0	5
3225	SLAI2_HUMAN	SLAIN motif-containing protein 2	S88	91%	0.91	28	1	0	0	0	0	1	2	1	0	0	0	0	5

3226	SLAI2_HUMAN	SLAIN motif-containing protein 2	T86	85%	0.85	26	1	0	0	0	0	1	2	1	0	0	0	0	5
3227	SRGP1_HUMAN	SLIT-ROBO Rho GTPase-activating protein 1	S1027	28%	0.28	1	0	0	0	0	1	0	0	0	0	0	0	0	1
3228	SRGP1_HUMAN	SLIT-ROBO Rho GTPase-activating protein 1	S1036	28%	0.28	1	0	0	0	0	1	0	0	0	0	0	0	0	1
3229	SRGP1_HUMAN	SLIT-ROBO Rho GTPase-activating protein 1	T1037	28%	0.28	1	0	0	0	0	1	0	0	0	0	0	0	0	1
3230	SMAP_HUMAN	Small acidic protein	S15	25%	0.25	6	0	0	0	0	0	0	0	0	0	0	1	0	1
3231	SMAP_HUMAN	Small acidic protein	S17	35%	0.35	86	2	5	5	0	2	2	0	2	2	0	0	0	20
3232	SGSM1_HUMAN	Small G protein signaling modulator 1	S407	22%	0.22	1	0	0	0	0	1	0	0	0	0	0	0	0	1
3233	SGSM1_HUMAN	Small G protein signaling modulator 1	T415	22%	0.22	1	0	0	0	0	1	0	0	0	0	0	0	0	1
3234	RUXF_HUMAN	Small nuclear ribonucleoprotein F	S2	100%	1.00	2	1	0	0	0	0	0	0	0	0	0	0	0	1
3235	SPR2B_HUMAN	Small proline-rich protein 2B	S60	100%	1.00	6	0	0	0	0	0	0	0	0	0	2	1	1	4
3236	SPR2D_HUMAN	Small proline-rich protein 2D	S60	100%	1.00	6	0	0	0	0	0	0	0	0	0	2	1	1	4
3237	UTP20_HUMAN	Small subunit processome component 20 homolog	S356	25%	0.25	1	0	0	0	0	1	0	0	0	0	0	0	0	1
3238	UTP20_HUMAN	Small subunit processome component 20 homolog	S358	31%	0.31	1	0	0	0	0	1	0	0	0	0	0	0	0	1
3239	UTP20_HUMAN	Small subunit processome component 20 homolog	T351	33%	0.33	1	0	0	0	0	1	0	0	0	0	0	0	0	1
3240	SUMO1_HUMAN	Small ubiquitin-related modifier 1	S2	100%	1.00	45	2	4	3	2	5	4	4	3	2	3	3	3	38
3241	SUMO3_HUMAN	Small ubiquitin-related modifier 3	S102	41%	0.41	62	0	0	3	0	1	1	1	2	3	1	0	3	15
3242	SUMO3_HUMAN	Small ubiquitin-related modifier 3	S96	14%	0.14	17	0	0	0	0	0	0	0	0	0	0	0	1	1
3243	SUMO3_HUMAN	Small ubiquitin-related modifier 3	S97	15%	0.15	6	0	0	0	0	0	2	0	0	0	0	0	0	2
3244	SUMO3_HUMAN	Small ubiquitin-related modifier 3	T90	24%	0.24	91	0	0	1	2	1	1	1	3	3	0	2	1	15

3245	SMTN_HUMAN	Smoothelin	S576	100%	1.00	5	0	0	0	0	0	0	0	0	0	1	1	0	2
3246	SMTN_HUMAN	Smoothelin	T645	65%	0.65	1	0	0	0	0	0	0	0	0	0	0	0	1	1
3247	SMTN_HUMAN	Smoothelin	T651	73%	0.73	1	0	0	0	0	0	0	0	0	0	0	0	1	1
3248	SNPC2_HUMAN	snRNA-activating protein complex subunit 2	S169	69%	0.69	1	0	0	0	1	0	0	0	0	0	0	0	0	1
3249	SNPC2_HUMAN	snRNA-activating protein complex subunit 2	S176	83%	0.83	1	0	0	0	1	0	0	0	0	0	0	0	0	1
3250	SNPC2_HUMAN	snRNA-activating protein complex subunit 2	S177	77%	0.77	1	0	0	0	1	0	0	0	0	0	0	0	0	1
3251	SCNBA_HUMAN	Sodium channel protein type 11 subunit alpha	S985	12%	0.12	1	1	0	0	0	0	0	0	0	0	0	0	0	1
3252	SCNBA_HUMAN	Sodium channel protein type 11 subunit alpha	S992	96%	0.96	3	1	0	0	0	0	0	0	0	0	0	0	0	1
3253	SL9A1_HUMAN	Sodium/hydrogen exchanger 1	S703	100%	1.00	41	0	0	0	0	0	6	0	0	0	0	3	1	10
3254	S38A1_HUMAN	Sodium-coupled neutral amino acid transporter 1	S52	69%	0.69	3	0	0	0	0	0	1	0	0	0	0	1	1	3
3255	S38A1_HUMAN	Sodium-coupled neutral amino acid transporter 1	S56	33%	0.33	1	0	0	0	0	0	0	0	0	0	1	0	0	1
3256	S38A1_HUMAN	Sodium-coupled neutral amino acid transporter 1	T54	81%	0.81	5	0	0	0	0	0	1	1	1	0	1	0	1	5
3257	S6A15_HUMAN	Sodium-dependent neutral amino acid transporter B(0)AT2	S675	100%	1.00	7	2	1	0	0	0	0	0	0	0	0	0	4	7
3258	S6A15_HUMAN	Sodium-dependent neutral amino acid transporter B(0)AT2	S687	100%	1.00	176	3	9	5	4	8	6	6	6	10	9	9	8	83
3259	NPT1_HUMAN	Sodium-dependent phosphate transport protein 1	S118	26%	0.26	1	0	1	0	0	0	0	0	0	0	0	0	0	1
3260	NPT1_HUMAN	Sodium-dependent phosphate transport protein 1	S122	85%	0.85	1	0	1	0	0	0	0	0	0	0	0	0	0	1
3261	GTR14_HUMAN	Solute carrier family 2, facilitated glucose transporter member 14	S370	62%	0.62	4	0	0	0	0	0	0	0	0	0	0	0	1	1

3262	GTR3_HUMAN	Solute carrier family 2, facilitated glucose transporter member 3	S346	62%	0.62	4	0	0	0	0	0	0	0	0	0	0	0	1	1
3263	GTR7_HUMAN	Solute carrier family 2, facilitated glucose transporter member 7	S505	52%	0.52	1	1	0	0	0	0	0	0	0	0	0	0	0	1
3264	S35F2_HUMAN	Solute carrier family 35 member F2	S5	100%	1.00	22	0	0	0	0	0	0	0	0	0	8	6	0	14
3265	S35F4_HUMAN	Solute carrier family 35 member F4	S339	47%	0.47	1	0	0	0	1	0	0	0	0	0	0	0	0	1
3266	S35F4_HUMAN	Solute carrier family 35 member F4	S341	47%	0.47	1	0	0	0	1	0	0	0	0	0	0	0	0	1
3267	S35F4_HUMAN	Solute carrier family 35 member F4	T342	47%	0.47	1	0	0	0	1	0	0	0	0	0	0	0	0	1
3268	S35F4_HUMAN	Solute carrier family 35 member F4	Y321	59%	0.59	1	0	0	0	1	0	0	0	0	0	0	0	0	1
3269	SO3A1_HUMAN	Solute carrier organic anion transporter family member 3A1	S414	100%	1.00	1	0	0	0	0	0	1	0	0	0	0	0	0	1
3270	SAS10_HUMAN	Something about silencing protein 10	S37	38%	0.38	1	1	0	0	0	0	0	0	0	0	0	0	0	1
3271	SNX1_HUMAN	Sorting nexin-1	S32	100%	1.00	256	7	6	12	8	6	6	7	5	2	6	4	3	72
3272	SNX1_HUMAN	Sorting nexin-1	S39	100%	1.00	220	6	3	8	5	5	4	7	4	1	4	3	3	53
3273	SNX1_HUMAN	Sorting nexin-1	T41	100%	1.00	144	2	4	4	3	1	4	3	2	1	3	4	1	32
3274	SNX2_HUMAN	Sorting nexin-2	S185	100%	1.00	12	1	2	0	0	1	0	0	1	0	0	0	0	5
3275	SARG_HUMAN	Specifically androgen-regulated gene protein	S385	100%	1.00	3	0	0	0	0	0	0	0	0	0	1	1	1	3
3276	SARG_HUMAN	Specifically androgen-regulated gene protein	S462	42%	0.42	6	0	0	0	0	3	2	1	0	0	0	0	0	6
3277	SARG_HUMAN	Specifically androgen-regulated gene protein	T456	49%	0.49	7	0	0	0	1	0	0	0	1	0	0	0	0	2
3278	SPTA2_HUMAN	Spectrin alpha chain, brain	S1029	57%	0.57	15	0	0	0	2	0	0	2	0	1	0	0	0	5
3279	SPTA2_HUMAN	Spectrin alpha chain, brain	S1031	95%	0.95	14	0	0	0	0	0	0	0	1	3	0	0	0	4

3280	SPTB2_HUMAN	Spectrin beta chain, brain 1	S2102	100%	1.00	1	0	0	0	0	0	0	0	0	1	0	0	0	1
3281	SPTB2_HUMAN	Spectrin beta chain, brain 1	S2160	65%	0.65	1	0	0	1	0	0	0	0	0	0	0	0	0	1
3282	SPTB2_HUMAN	Spectrin beta chain, brain 1	S2172	87%	0.87	1	0	0	1	0	0	0	0	0	0	0	0	0	1
3283	SPTB2_HUMAN	Spectrin beta chain, brain 1	T2159	51%	0.51	1	0	0	1	0	0	0	0	0	0	0	0	0	1
3284	SPTN2_HUMAN	Spectrin beta chain, brain 2	S2171	100%	1.00	17	1	0	0	0	0	0	3	0	3	0	0	0	7
3285	SPAG1_HUMAN	Sperm-associated antigen 1	S423	100%	1.00	93	2	10	5	3	5	6	5	2	2	0	0	2	42
3286	SPEE_HUMAN	Spermidine synthase	S11	100%	1.00	1	0	0	0	0	0	0	0	0	0	0	1	0	1
3287	SSFA2_HUMAN	Sperm-specific antigen 2	S92	98%	0.98	9	1	0	0	0	1	1	2	1	0	0	0	0	6
3288	SGPL1_HUMAN	Sphingosine-1-phosphate lyase 1	S559	24%	0.24	2	0	1	0	0	0	0	0	0	0	0	1	0	2
3289	SF3A1_HUMAN	Splicing factor 3A subunit 1	S329	100%	1.00	93	4	6	9	3	6	6	6	5	4	3	2	3	57
3290	SF3B1_HUMAN	Splicing factor 3B subunit 1	T436	22%	0.22	1	0	0	0	0	0	1	0	0	0	0	0	0	1
3291	SF3B2_HUMAN	Splicing factor 3B subunit 2	T780	90%	0.90	4	1	2	1	0	0	0	0	0	0	0	0	0	4
3292	SPF45_HUMAN	Splicing factor 45	S222	92%	0.92	29	1	0	0	1	0	1	0	0	0	0	0	1	4
3293	SPF45_HUMAN	Splicing factor 45	S229	51%	0.51	15	0	0	0	1	1	0	1	1	0	0	0	0	4
3294	SPF45_HUMAN	Splicing factor 45	T224	57%	0.57	214	3	3	7	4	4	5	4	6	6	3	3	3	51
3295	U2AF2_HUMAN	Splicing factor U2AF 65 kDa subunit	S2	100%	1.00	69	8	6	7	6	6	6	6	6	6	3	5	4	69
3296	U2AF2_HUMAN	Splicing factor U2AF 65 kDa subunit	T126	82%	0.82	1	0	1	0	0	0	0	0	0	0	0	0	0	1
3297	SFR15_HUMAN	Splicing factor, arginine/serine-rich 15	S154	100%	1.00	####	24	28	32	34	25	20	26	27	23	23	24	20	306
3298	SFR15_HUMAN	Splicing factor, arginine/serine-rich 15	T149	100%	1.00	101	2	3	2	1	0	1	4	1	3	3	3	2	25

3299	SFR19_HUMAN	Splicing factor, arginine/serine-rich 19	S239	100%	1.00	45	1	2	1	1	1	1	2	1	1	1	1	1	14
3300	SFR19_HUMAN	Splicing factor, arginine/serine-rich 19	S552	79%	0.79	1	0	1	0	0	0	0	0	0	0	0	0	0	1
3301	SPON1_HUMAN	Spondin-1	S253	28%	0.28	1	0	0	0	0	0	0	0	0	0	1	0	0	1
3302	SPON1_HUMAN	Spondin-1	S264	56%	0.56	1	0	0	0	0	0	0	0	0	0	1	0	0	1
3303	ERG1_HUMAN	Squalene monooxygenase	S301	100%	1.00	1	0	0	1	0	0	0	0	0	0	0	0	0	1
3304	ERG1_HUMAN	Squalene monooxygenase	S303	100%	1.00	1	0	0	1	0	0	0	0	0	0	0	0	0	1
3305	FDFT_HUMAN	Squalene synthase	S184	100%	1.00	16	1	0	0	0	0	0	0	0	1	1	2	1	6
3306	FDFT_HUMAN	Squalene synthase	S188	100%	1.00	16	1	0	0	0	0	0	0	0	1	1	2	1	6
3307	FDFT_HUMAN	Squalene synthase	S190	100%	1.00	16	1	0	0	0	0	0	0	0	1	1	2	1	6
3308	FDFT_HUMAN	Squalene synthase	Y171	100%	1.00	16	1	0	0	0	0	0	0	0	1	1	2	1	6
3309	FDFT_HUMAN	Squalene synthase	Y174	100%	1.00	16	1	0	0	0	0	0	0	0	1	1	2	1	6
3310	SRC8_HUMAN	Src substrate cortactin	S405	100%	1.00	85	0	0	0	3	3	1	1	1	1	3	9	8	30
3311	SRC8_HUMAN	Src substrate cortactin	S417	95%	0.95	64	0	1	2	0	0	1	3	1	1	2	2	3	16
3312	SRC8_HUMAN	Src substrate cortactin	S418	100%	1.00	597	9	11	8	13	10	11	9	13	11	13	12	11	131
3313	SRC8_HUMAN	Src substrate cortactin	T399	96%	0.96	42	0	0	0	2	0	0	0	0	0	2	6	6	16
3314	SRC8_HUMAN	Src substrate cortactin	T401	100%	1.00	373	5	8	7	14	8	8	8	5	7	8	5	8	91
3315	STAR9_HUMAN	StAR-related lipid transfer protein 9	S2327	24%	0.24	1	0	0	0	0	1	0	0	0	0	0	0	0	1
3316	STAR9_HUMAN	StAR-related lipid transfer protein 9	S2332	24%	0.24	1	0	0	0	0	1	0	0	0	0	0	0	0	1
3317	STAR9_HUMAN	StAR-related lipid transfer protein 9	S2351	100%	1.00	1	0	0	0	0	1	0	0	0	0	0	0	0	1
3318	STAR9_HUMAN	StAR-related lipid transfer protein 9	S2353	98%	0.98	1	0	0	0	0	1	0	0	0	0	0	0	0	1

3319	STAR9_HUMAN	StAR-related lipid transfer protein 9	S2355	100%	1.00	1	0	0	0	0	1	0	0	0	0	0	0	0	1
3320	STAR9_HUMAN	StAR-related lipid transfer protein 9	T2354	100%	1.00	1	0	0	0	0	1	0	0	0	0	0	0	0	1
3321	STMN1_HUMAN	Stathmin	S16	100%	1.00	330	2	7	0	15	11	8	4	4	4	4	1	0	60
3322	STMN1_HUMAN	Stathmin	S25	100%	1.00	326	0	0	0	0	0	0	0	0	0	27	20	21	68
3323	STMN1_HUMAN	Stathmin	S38	100%	1.00	263	4	2	4	9	5	3	4	2	5	1	3	4	46
3324	SLK_HUMAN	STE20-like serine/threonine-protein kinase	S189	55%	0.55	1	0	1	0	0	0	0	0	0	0	0	0	0	1
3325	SAMD9_HUMAN	Sterile alpha motif domain-containing protein 9	S129	25%	0.25	1	0	0	0	0	0	0	0	0	0	0	0	1	1
3326	SRA1_HUMAN	Steroid receptor RNA activator 1	S87	98%	0.98	109	5	0	1	0	1	3	4	0	1	5	1	1	22
3327	SRA1_HUMAN	Steroid receptor RNA activator 1	S92	51%	0.51	12	0	0	0	0	0	0	0	0	0	1	0	0	1
3328	SRA1_HUMAN	Steroid receptor RNA activator 1	S96	91%	0.91	22	0	0	1	0	1	0	0	2	0	0	1	0	5
3329	STIP1_HUMAN	Stress-induced-phosphoprotein 1	S16	100%	1.00	2	0	0	0	0	0	0	0	0	0	0	0	2	2
3330	STRN_HUMAN	Striatin	S245	100%	1.00	15	2	1	0	0	2	0	0	1	0	0	2	0	8
3331	SMC4_HUMAN	Structural maintenance of chromosomes protein 4	S27	82%	0.82	14	0	0	1	0	0	1	0	1	0	0	0	0	3
3332	SMC4_HUMAN	Structural maintenance of chromosomes protein 4	S28	100%	1.00	97	3	2	2	3	3	1	3	2	1	0	0	0	20
3333	SMC4_HUMAN	Structural maintenance of chromosomes protein 4	S41	20%	0.20	116	5	2	6	0	6	2	3	0	3	2	4	3	36
3334	SMC4_HUMAN	Structural maintenance of chromosomes protein 4	T39	89%	0.89	28	0	1	0	1	1	1	2	3	0	1	1	1	12
3335	QSOX2_HUMAN	Sulfhydryl oxidase 2	S578	79%	0.79	1	0	0	0	0	0	0	0	1	0	0	0	0	1
3336	ST1A1_HUMAN	Sulfotransferase 1A1	T95	98%	0.98	1	0	0	0	0	1	0	0	0	0	0	0	0	1
3337	SUN1_HUMAN	SUN domain-containing protein 1	S647	54%	0.54	1	0	1	0	0	0	0	0	0	0	0	0	0	1

3338	SUN1_HUMAN	SUN domain-containing protein 1	Y633	19%	0.19	1	0	1	0	0	0	0	0	0	0	0	0	0	1
3339	SVIL_HUMAN	Supervillin	S1545	66%	0.66	1	0	0	0	0	0	0	0	0	0	1	0	0	1
3340	ST18_HUMAN	Suppression of tumorigenicity 18 protein	S532	100%	1.00	2	1	0	0	0	0	0	0	0	0	1	0	0	2
3341	SSF1_HUMAN	Suppressor of SWI4 1 homolog	S238	100%	1.00	519	7	3	9	11	5	12	6	2	11	6	11	7	90
3342	SSF1_HUMAN	Suppressor of SWI4 1 homolog	S240	100%	1.00	168	2	3	7	3	3	4	0	0	3	2	3	1	31
3343	SSF1_HUMAN	Suppressor of SWI4 1 homolog	T233	36%	0.36	2	0	0	1	0	0	0	0	0	0	0	0	0	1
3344	SUGP1_HUMAN	SURP and G-patch domain-containing protein 1	S485	64%	0.64	2	0	0	0	0	0	0	0	0	2	0	0	0	2
3345	SMN_HUMAN	Survival motor neuron protein	S28	100%	1.00	94	3	2	5	7	2	1	4	5	4	1	0	1	35
3346	SMN_HUMAN	Survival motor neuron protein	S31	100%	1.00	19	0	0	0	0	0	0	0	0	0	0	1	0	1
3347	SMN_HUMAN	Survival motor neuron protein	T25	66%	0.66	14	1	0	1	1	0	0	0	1	0	1	2	1	8
3348	SMRC2_HUMAN	SWI/SNF complex subunit SMARCC2	S347	100%	1.00	108	1	1	1	4	2	1	2	2	1	3	3	2	23
3349	SMCA5_HUMAN	SWI/SNF-related matrix-associated actin-dependent regulator of chromatin subfamily A member 5	S66	100%	1.00	580	17	13	15	10	15	12	11	9	8	12	10	10	142
3350	SMCE1_HUMAN	SWI/SNF-related matrix-associated actin-dependent regulator of chromatin subfamily E member 1	S2	53%	0.53	2	1	0	0	0	0	0	0	0	0	0	0	0	1
3351	SMCE1_HUMAN	SWI/SNF-related matrix-associated actin-dependent regulator of chromatin subfamily E member 1	S21	71%	0.71	2	1	0	0	0	0	0	0	0	0	0	0	0	1
3352	SMCE1_HUMAN	SWI/SNF-related matrix-associated actin-dependent regulator of chromatin subfamily E member 1	S32	71%	0.71	2	1	0	0	0	0	0	0	0	0	0	0	0	1

3353	SMCE1_HUMAN	SWI/SNF-related matrix-associated actin-dependent regulator of chromatin subfamily E member 1	T12	32%	0.32	1	0	0	1	0	0	0	0	0	0	0	0	0	1
3354	SMCE1_HUMAN	SWI/SNF-related matrix-associated actin-dependent regulator of chromatin subfamily E member 1	T22	56%	0.56	2	1	0	0	0	0	0	0	0	0	0	0	0	1
3355	SMCE1_HUMAN	SWI/SNF-related matrix-associated actin-dependent regulator of chromatin subfamily E member 1	Y28	41%	0.41	2	0	0	1	0	0	0	0	0	0	0	0	0	1
3356	SMCE1_HUMAN	SWI/SNF-related matrix-associated actin-dependent regulator of chromatin subfamily E member 1	Y31	46%	0.46	2	0	0	1	0	0	0	0	0	0	0	0	0	1
3357	SMCE1_HUMAN	SWI/SNF-related matrix-associated actin-dependent regulator of chromatin subfamily E member 1	Y36	74%	0.74	4	1	0	1	0	0	0	0	0	0	0	0	0	2
3358	SMCE1_HUMAN	SWI/SNF-related matrix-associated actin-dependent regulator of chromatin subfamily E member 1	Y39	42%	0.42	2	1	0	0	0	0	0	0	0	0	0	0	0	1
3359	SYMPK_HUMAN	Symplekin	S494	15%	0.15	2	0	0	0	0	0	0	0	0	0	2	0	0	2
3360	SVOP_HUMAN	Synaptic vesicle 2-related protein	T448	3%	0.03	1	0	0	0	0	0	0	0	0	0	0	1	0	1
3361	SYNJ1_HUMAN	Synaptojanin-1	S1385	66%	0.66	1	0	1	0	0	0	0	0	0	0	0	0	0	1
3362	SYNJ1_HUMAN	Synaptojanin-1	S1392	73%	0.73	1	0	1	0	0	0	0	0	0	0	0	0	0	1
3363	SYT8_HUMAN	Synaptotagmin-8	S157	69%	0.69	1	0	1	0	0	0	0	0	0	0	0	0	0	1
3364	SYTL4_HUMAN	Synaptotagmin-like protein 4	S289	86%	0.86	1	0	0	0	0	0	0	0	0	1	0	0	0	1
3365	STXB3_HUMAN	Syntaxin-binding protein 3	T52	97%	0.97	1	0	0	0	0	0	0	0	0	0	0	1	0	1
3366	STXB3_HUMAN	Syntaxin-binding protein 3	Y66	98%	0.98	1	0	0	0	0	0	0	0	0	0	0	1	0	1

3367	STXB5_HUMAN	Syntaxin-binding protein 5	S759	100%	1.00	16	2	0	0	0	0	0	1	0	0	0	0	0	3
3368	SDCB2_HUMAN	Syntenin-2	S266	21%	0.21	2	0	0	0	0	0	1	0	0	0	0	0	1	2
3369	SDCB2_HUMAN	Syntenin-2	Y269	67%	0.67	2	0	0	0	0	0	1	0	0	0	0	0	1	2
3370	TLN1_HUMAN	Talin-1	S1225	98%	0.98	3	0	0	0	0	0	0	0	0	0	0	3	0	3
3371	TLN1_HUMAN	Talin-1	S405	67%	0.67	2	0	0	0	0	0	0	1	0	0	0	0	0	1
3372	TPSNR_HUMAN	Tapasin-related protein	T132	77%	0.77	2	1	0	0	0	0	0	0	0	0	0	0	0	1
3373	TPSNR_HUMAN	Tapasin-related protein	T134	71%	0.71	1	1	0	0	0	0	0	0	0	0	0	0	0	1
3374	TPSNR_HUMAN	Tapasin-related protein	T135	56%	0.56	1	1	0	0	0	0	0	0	0	0	0	0	0	1
3375	TPSNR_HUMAN	Tapasin-related protein	T138	56%	0.56	1	1	0	0	0	0	0	0	0	0	0	0	0	1
3376	TPSNR_HUMAN	Tapasin-related protein	Y127	63%	0.63	1	1	0	0	0	0	0	0	0	0	0	0	0	1
3377	TOM1_HUMAN	Target of Myb protein 1	S160	98%	0.98	1	0	0	0	0	0	0	0	0	0	1	0	0	1
3378	TOM1_HUMAN	Target of Myb protein 1	S462	95%	0.95	16	0	0	0	1	0	0	0	0	0	2	1	2	6
3379	TOM1_HUMAN	Target of Myb protein 1	S464	8%	0.08	1	0	0	0	0	0	0	0	0	1	0	0	0	1
3380	TPX2_HUMAN	Targeting protein for Xklp2	S738	100%	1.00	19	0	0	0	0	0	0	0	0	0	0	1	0	1
3381	T2R45_HUMAN	Taste receptor type 2 member 45	S54	86%	0.86	44	0	3	4	2	3	2	3	6	5	0	0	0	28
3382	T2R45_HUMAN	Taste receptor type 2 member 45	T49	97%	0.97	44	0	3	4	2	3	2	3	6	5	0	0	0	28
3383	BTAF1_HUMAN	TATA-binding protein-associated factor 172	S261	2%	0.02	1	0	0	0	0	0	0	1	0	0	0	0	0	1
3384	TX1B3_HUMAN	Tax1-binding protein 3	S61	100%	1.00	1	0	0	0	0	0	0	0	0	0	0	0	1	1
3385	TB10B_HUMAN	TBC1 domain family member 10B	S132	98%	0.98	11	1	1	0	0	0	2	0	1	0	2	0	0	7
3386	TB10B_HUMAN	TBC1 domain family member 10B	S687	100%	1.00	156	8	13	8	2	4	5	6	6	3	2	4	3	64

3387	TB10B_HUMAN	TBC1 domain family member 10B	T135	12%	0.12	3	1	0	0	1	0	0	0	0	0	1	0	0	3
3388	TB10B_HUMAN	TBC1 domain family member 10B	T697	22%	0.22	12	0	0	1	0	0	0	0	0	0	0	0	0	1
3389	TBD2A_HUMAN	TBC1 domain family member 2A	S436	100%	1.00	14	1	0	0	0	0	1	0	0	0	0	1	0	3
3390	TBCD4_HUMAN	TBC1 domain family member 4	S106	83%	0.83	2	0	0	0	0	0	0	0	0	0	0	1	1	2
3391	TBCD4_HUMAN	TBC1 domain family member 4	S809	36%	0.36	1	0	0	0	0	0	0	0	1	0	0	0	0	1
3392	TBX2_HUMAN	T-box transcription factor TBX2	S355	98%	0.98	2	0	0	0	0	1	0	0	0	0	0	0	1	2
3393	TFPT_HUMAN	TCF3 fusion partner	S180	100%	1.00	64	4	4	5	1	4	3	5	5	2	5	2	1	41
3394	TCPG_HUMAN	T-complex protein 1 subunit gamma	S166	22%	0.22	3	0	0	1	0	0	0	0	0	0	0	0	0	1
3395	TSH3_HUMAN	Teashirt homolog 3	S570	34%	0.34	6	0	0	0	0	0	0	0	0	0	1	1	0	2
3396	TSH3_HUMAN	Teashirt homolog 3	S596	76%	0.76	6	0	0	0	0	0	0	0	0	0	1	1	0	2
3397	TSH3_HUMAN	Teashirt homolog 3	S597	61%	0.61	6	0	0	0	0	0	0	0	0	0	1	1	0	2
3398	TSH3_HUMAN	Teashirt homolog 3	S600	85%	0.85	6	0	0	0	0	0	0	0	0	0	1	1	0	2
3399	TSH3_HUMAN	Teashirt homolog 3	S818	18%	0.18	1	0	0	0	0	0	0	0	0	0	0	1	0	1
3400	TSH3_HUMAN	Teashirt homolog 3	S819	18%	0.18	1	0	0	0	0	0	0	0	0	0	0	1	0	1
3401	TSH3_HUMAN	Teashirt homolog 3	T599	92%	0.92	6	0	0	0	0	0	0	0	0	0	1	1	0	2
3402	TSH3_HUMAN	Teashirt homolog 3	T817	36%	0.36	1	0	0	0	0	0	0	0	0	0	0	1	0	1
3403	TKT4M_HUMAN	Tektin-4 like protein LOC389833	S18	73%	0.73	1	0	0	0	0	0	0	0	0	0	0	1	0	1
3404	WAP53_HUMAN	Telomerase Cajal body protein 1	S491	100%	1.00	621	14	16	10	14	19	12	12	13	7	5	1	3	126
3405	WAP53_HUMAN	Telomerase Cajal body protein 1	S54	100%	1.00	43	0	0	0	2	2	1	0	1	1	0	0	0	7
3406	WAP53_HUMAN	Telomerase Cajal body protein 1	S90	100%	1.00	11	0	0	0	0	1	0	0	0	0	0	0	0	1

3407	WAP53_HUMAN	Telomerase Cajal body protein 1	T489	62%	0.62	16	1	1	1	2	0	1	0	2	1	0	0	0	9
3408	RIF1_HUMAN	Telomere-associated protein RIF1	S2144	100%	1.00	36	0	0	0	0	1	0	0	0	0	0	0	0	1
3409	RIF1_HUMAN	Telomere-associated protein RIF1	S983	39%	0.39	1	0	0	0	0	0	0	0	0	0	0	1	0	1
3410	RIF1_HUMAN	Telomere-associated protein RIF1	S989	94%	0.94	1	0	0	0	0	0	0	0	0	0	0	1	0	1
3411	RIF1_HUMAN	Telomere-associated protein RIF1	T986	33%	0.33	1	0	0	0	0	0	0	0	0	0	0	1	0	1
3412	TERF2_HUMAN	Telomeric repeat-binding factor 2	S323	100%	1.00	42	0	0	0	1	0	0	1	1	0	1	0	0	4
3413	TE2IP_HUMAN	Telomeric repeat-binding factor 2-interacting protein 1	S203	100%	1.00	29	2	2	0	1	1	0	0	0	0	0	1	0	7
3414	TE2IP_HUMAN	Telomeric repeat-binding factor 2-interacting protein 1	S205	10%	0.10	4	0	0	0	0	1	0	0	0	0	0	1	0	2
3415	TEN1_HUMAN	Teneurin-1	S2071	25%	0.25	1	0	0	0	1	0	0	0	0	0	0	0	0	1
3416	TEN1_HUMAN	Teneurin-1	Y2067	25%	0.25	1	0	0	0	1	0	0	0	0	0	0	0	0	1
3417	TENS3_HUMAN	Tensin-3	S690	99%	0.99	16	0	0	1	1	0	1	0	0	1	0	1	0	5
3418	TENS3_HUMAN	Tensin-3	T692	56%	0.56	3	0	0	1	0	0	0	0	0	0	0	0	0	1
3419	TENS4_HUMAN	Tensin-4	S385	55%	0.55	11	1	1	0	1	0	1	0	0	0	0	0	0	4
3420	TENS4_HUMAN	Tensin-4	S386	99%	0.99	65	3	2	5	7	4	3	2	1	0	2	2	1	32
3421	TENS4_HUMAN	Tensin-4	S416	11%	0.11	1	0	0	0	0	1	0	0	0	0	0	0	0	1
3422	TINF2_HUMAN	TERF1-interacting nuclear factor 2	S301	100%	1.00	116	3	1	4	4	3	2	2	3	0	3	1	2	28
3423	TINF2_HUMAN	TERF1-interacting nuclear factor 2	S305	92%	0.92	116	3	1	4	4	3	2	2	3	0	3	1	2	28
3424	TINF2_HUMAN	TERF1-interacting nuclear factor 2	T297	78%	0.78	116	3	1	4	4	3	2	2	3	0	3	1	2	28
3425	TINF2_HUMAN	TERF1-interacting nuclear factor 2	T313	94%	0.94	116	3	1	4	4	3	2	2	3	0	3	1	2	28
3426	TINF2_HUMAN	TERF1-interacting nuclear factor 2	T320	99%	0.99	116	3	1	4	4	3	2	2	3	0	3	1	2	28

3427	TEX2_HUMAN	Testis-expressed sequence 2 protein	S295	100%	1.00	8	1	1	0	0	0	0	1	0	0	1	0	0	4
3428	TSKS_HUMAN	Testis-specific serine kinase substrate	S556	99%	0.99	2	0	1	0	0	0	0	0	0	0	1	0	0	2
3429	TSKS_HUMAN	Testis-specific serine kinase substrate	S562	100%	1.00	2	0	1	0	0	0	0	0	0	0	1	0	0	2
3430	TSKS_HUMAN	Testis-specific serine kinase substrate	S569	93%	0.93	2	0	1	0	0	0	0	0	0	0	1	0	0	2
3431	TSKS_HUMAN	Testis-specific serine kinase substrate	S588	62%	0.62	2	0	1	0	0	0	0	0	0	0	1	0	0	2
3432	TSKS_HUMAN	Testis-specific serine kinase substrate	T570	97%	0.97	2	0	1	0	0	0	0	0	0	0	1	0	0	2
3433	TSYL2_HUMAN	Testis-specific Y-encoded-like protein 2	T12	100%	1.00	1	0	0	0	0	0	0	0	1	0	0	0	0	1
3434	TSYL6_HUMAN	Testis-specific Y-encoded-like protein 6	S110	32%	0.32	1	0	0	0	0	0	0	0	1	0	0	0	0	1
3435	ASPC1_HUMAN	Tether containing UBX domain for GLUT4	S500	100%	1.00	11	0	0	0	0	0	0	0	0	0	0	1	1	2
3436	ASPC1_HUMAN	Tether containing UBX domain for GLUT4	S502	98%	0.98	8	0	0	0	0	0	0	0	0	0	0	0	2	2
3437	TSN10_HUMAN	Tetraspanin-10	S83	26%	0.26	1	0	0	0	0	0	0	0	0	0	0	1	0	1
3438	TSN10_HUMAN	Tetraspanin-10	Y78	82%	0.82	1	0	0	0	0	0	0	0	0	0	0	1	0	1
3439	TTC15_HUMAN	Tetratricopeptide repeat protein 15	S276	100%	1.00	4	0	0	0	0	0	0	0	0	0	0	1	0	1
3440	TT21A_HUMAN	Tetratricopeptide repeat protein 21A	S210	59%	0.59	2	0	0	0	0	0	0	0	0	0	0	0	1	1
3441	TT21A_HUMAN	Tetratricopeptide repeat protein 21A	T209	47%	0.47	2	0	0	0	0	0	0	0	0	0	0	0	1	1
3442	TTC26_HUMAN	Tetratricopeptide repeat protein 26	S530	100%	1.00	1	0	0	0	0	0	0	0	0	0	0	0	1	1
3443	TTC26_HUMAN	Tetratricopeptide repeat protein 26	T531	100%	1.00	1	0	0	0	0	0	0	0	0	0	0	0	1	1
3444	TTC26_HUMAN	Tetratricopeptide repeat protein 26	T534	100%	1.00	1	0	0	0	0	0	0	0	0	0	0	0	1	1

3445	TTC7A_HUMAN	Tetratricopeptide repeat protein 7A	S51	90%	0.90	2	0	2	0	0	0	0	0	0	0	0	0	0	2
3446	TTC7A_HUMAN	Tetratricopeptide repeat protein 7A	S647	100%	1.00	1	0	0	0	0	0	0	1	0	0	0	0	0	1
3447	TGFR2_HUMAN	TGF-beta receptor type-2	S548	100%	1.00	1	0	0	0	0	0	0	1	0	0	0	0	0	1
3448	TAB2_HUMAN	TGF-beta-activated kinase 1 and MAP3K7-binding protein 2	S524	69%	0.69	5	0	0	1	0	1	0	0	1	0	0	0	0	3
3449	THAP2_HUMAN	THAP domain-containing protein 2	T13	80%	0.80	6	1	0	0	0	0	0	0	0	1	0	0	0	2
3450	THAP2_HUMAN	THAP domain-containing protein 2	T3	50%	0.50	6	1	0	0	0	0	0	0	0	1	0	0	0	2
3451	THIO_HUMAN	Thioredoxin	T9	92%	0.92	107	1	2	3	1	2	2	2	3	1	3	4	6	30
3452	TXD16_HUMAN	Thioredoxin domain-containing protein 16	S13	62%	0.62	1	0	0	0	0	0	1	0	0	0	0	0	0	1
3453	TXD16_HUMAN	Thioredoxin domain-containing protein 16	S3	62%	0.62	1	0	0	0	0	0	1	0	0	0	0	0	0	1
3454	TXD16_HUMAN	Thioredoxin domain-containing protein 16	S32	92%	0.92	1	0	0	0	0	0	1	0	0	0	0	0	0	1
3455	TXD16_HUMAN	Thioredoxin domain-containing protein 16	T24	62%	0.62	1	0	0	0	0	0	1	0	0	0	0	0	0	1
3456	THOC4_HUMAN	THO complex subunit 4	S8	100%	1.00	3	0	2	0	0	0	0	0	0	0	0	1	0	3
3457	KITH_HUMAN	Thymidine kinase, cytosolic	S15	25%	0.25	3	0	0	0	0	0	1	0	0	0	0	0	0	1
3458	TR150_HUMAN	Thyroid hormone receptor-associated protein 3	S575	100%	1.00	3	0	0	0	0	0	0	0	0	0	2	1	0	3
3459	TR150_HUMAN	Thyroid hormone receptor-associated protein 3	S682	100%	1.00	402	14	15	12	0	9	4	6	0	0	21	22	28	131
3460	TR150_HUMAN	Thyroid hormone receptor-associated protein 3	S684	46%	0.46	89	5	5	5	0	3	2	2	0	0	5	5	1	33
3461	TR150_HUMAN	Thyroid hormone receptor-associated protein 3	T685	33%	0.33	6	0	0	0	0	0	0	0	0	0	1	0	0	1
3462	TR150_HUMAN	Thyroid hormone receptor-associated protein 3	T941	92%	0.92	9	0	0	0	0	0	0	0	0	1	0	0	0	1

3463	TRIPB_HUMAN	Thyroid receptor-interacting protein 11	S1891	100%	1.00	1	0	0	0	0	0	0	0	0	0	0	0	1	1
3464	TRIP6_HUMAN	Thyroid receptor-interacting protein 6	S101	38%	0.38	7	0	0	0	0	1	0	0	0	1	0	0	0	2
3465	TRIP6_HUMAN	Thyroid receptor-interacting protein 6	S92	100%	1.00	63	0	4	0	0	3	1	3	3	1	3	3	1	22
3466	TIGD2_HUMAN	Tigger transposable element-derived protein 2	S321	99%	0.99	4	0	0	0	0	1	0	0	0	0	0	0	0	1
3467	TIGD2_HUMAN	Tigger transposable element-derived protein 2	S327	93%	0.93	1	0	0	0	0	1	0	0	0	0	0	0	0	1
3468	TIGD2_HUMAN	Tigger transposable element-derived protein 2	T320	100%	1.00	4	0	0	0	0	1	0	0	0	0	0	0	0	1
3469	TIGD2_HUMAN	Tigger transposable element-derived protein 2	Y314	100%	1.00	4	0	0	0	0	1	0	0	0	0	0	0	0	1
3470	TIGD3_HUMAN	Tigger transposable element-derived protein 3	S327	47%	0.47	1	0	0	0	0	0	0	1	0	0	0	0	0	1
3471	TIGD3_HUMAN	Tigger transposable element-derived protein 3	T336	53%	0.53	1	0	0	0	0	0	0	1	0	0	0	0	0	1
3472	ZO1_HUMAN	Tight junction protein ZO-1	S1617	97%	0.97	11	0	0	0	0	0	0	0	0	0	0	0	3	3
3473	ZO1_HUMAN	Tight junction protein ZO-1	S617	99%	0.99	9	0	0	1	0	0	2	0	0	2	2	2	0	9
3474	ZO2_HUMAN	Tight junction protein ZO-2	S130	100%	1.00	818	6	12	5	21	17	20	17	11	20	25	31	24	209
3475	ZO2_HUMAN	Tight junction protein ZO-2	S398	100%	1.00	11	0	0	0	0	0	0	0	0	0	0	1	0	1
3476	ZO2_HUMAN	Tight junction protein ZO-2	S400	100%	1.00	11	0	0	0	0	0	0	0	0	0	0	1	0	1
3477	ZO2_HUMAN	Tight junction protein ZO-2	S702	100%	1.00	146	1	2	1	0	0	2	1	2	3	6	12	7	37
3478	ZO2_HUMAN	Tight junction protein ZO-2	S978	100%	1.00	5	0	0	0	0	0	0	0	0	0	0	1	1	2
3479	ZO2_HUMAN	Tight junction protein ZO-2	S986	100%	1.00	315	4	6	6	11	10	14	10	5	5	12	8	9	100
3480	ZO2_HUMAN	Tight junction protein ZO-2	T707	11%	0.11	7	0	0	0	0	0	0	0	0	0	0	2	0	2

3481	ZO2_HUMAN	Tight junction protein ZO-2	T933	98%	0.98	6	0	0	0	0	0	1	0	0	0	0	0	0	1
3482	ZO3_HUMAN	Tight junction protein ZO-3	S164	100%	1.00	26	0	0	0	0	0	0	1	4	0	2	0	2	9
3483	TJAP1_HUMAN	Tight junction-associated protein 1	S300	100%	1.00	47	0	0	1	2	2	0	0	0	0	1	4	1	11
3484	TJAP1_HUMAN	Tight junction-associated protein 1	S545	100%	1.00	9	0	0	0	0	1	0	0	1	1	0	0	0	3
3485	TJAP1_HUMAN	Tight junction-associated protein 1	T422	100%	1.00	3	0	0	0	0	0	0	0	0	0	1	1	1	3
3486	TITIN_HUMAN	Titin	S19	6%	0.06	1	0	0	0	0	0	0	0	0	0	0	1	0	1
3487	TITIN_HUMAN	Titin	T25149	38%	0.38	1	0	0	1	0	0	0	0	0	0	0	0	0	1
3488	TRAF1_HUMAN	TNF receptor-associated factor 1	T25	94%	0.94	1	0	0	0	0	0	0	0	0	1	0	0	0	1
3489	TRAF4_HUMAN	TNF receptor-associated factor 4	S426	100%	1.00	1	0	0	0	0	0	0	0	0	0	0	0	1	1
3490	TNIP1_HUMAN	TNFAIP3-interacting protein 1	S77	100%	1.00	103	0	0	0	4	0	0	0	0	0	1	1	1	7
3491	TNIP1_HUMAN	TNFAIP3-interacting protein 1	S79	95%	0.95	9	0	0	0	0	0	1	0	0	0	0	0	0	1
3492	TLR8_HUMAN	Toll-like receptor 8	S71	76%	0.76	1	0	0	0	0	0	1	0	0	0	0	0	0	1
3493	TLR8_HUMAN	Toll-like receptor 8	T66	41%	0.41	1	0	0	0	0	0	1	0	0	0	0	0	0	1
3494	TLR8_HUMAN	Toll-like receptor 8	T76	61%	0.61	1	0	0	0	0	0	1	0	0	0	0	0	0	1
3495	TLR8_HUMAN	Toll-like receptor 8	T79	70%	0.70	1	0	0	0	0	0	1	0	0	0	0	0	0	1
3496	TM1L2_HUMAN	TOM1-like protein 2	S424	33%	0.33	5	0	0	0	0	0	0	0	0	0	0	1	0	1
3497	TPPC9_HUMAN	Trafficking protein particle complex subunit 9	S953	100%	1.00	1	0	0	0	0	0	0	0	0	0	0	0	1	1
3498	TRAD1_HUMAN	TRAF-type zinc finger domain-containing protein 1	S415	100%	1.00	133	4	4	3	4	3	3	5	3	3	3	2	3	40
3499	SMCA4_HUMAN	Transcription activator BRG1	S1452	100%	1.00	3	1	2	0	0	0	0	0	0	0	0	0	0	3
3500	SMCA4_HUMAN	Transcription activator BRG1	S662	10%	0.10	1	1	0	0	0	0	0	0	0	0	0	0	0	1

3501	TCEA1_HUMAN	Transcription elongation factor A protein 1	S100	100%	1.00	3	0	0	1	0	0	0	0	0	2	0	0	0	3
3502	SPT5H_HUMAN	Transcription elongation factor SPT5	S666	100%	1.00	76	1	4	3	1	3	3	1	2	1	3	3	1	26
3503	SPT6H_HUMAN	Transcription elongation factor SPT6	S1708	14%	0.14	1	0	1	0	0	0	0	0	0	0	0	0	0	1
3504	SPT6H_HUMAN	Transcription elongation factor SPT6	T1718	100%	1.00	3	0	3	0	0	0	0	0	0	0	0	0	0	3
3505	TF7L2_HUMAN	Transcription factor 7-like 2	S509	23%	0.23	1	0	0	0	0	0	1	0	0	0	0	0	0	1
3506	TF7L2_HUMAN	Transcription factor 7-like 2	Y489	22%	0.22	1	0	0	0	0	0	1	0	0	0	0	0	0	1
3507	BTF3_HUMAN	Transcription factor BTF3	S30	15%	0.15	1	0	1	0	0	0	0	0	0	0	0	0	0	1
3508	TFE3_HUMAN	Transcription factor E3	S556	100%	1.00	52	0	0	1	0	0	0	2	0	2	0	2	3	10
3509	TFEB_HUMAN	Transcription factor EB	S332	91%	0.91	3	0	0	0	0	0	0	2	1	0	0	0	0	3
3510	TFEB_HUMAN	Transcription factor EB	S452	8%	0.08	1	0	0	0	0	0	0	0	1	0	0	0	0	1
3511	TFEB_HUMAN	Transcription factor EB	S455	97%	0.97	5	0	0	0	0	0	0	0	2	1	1	1	0	5
3512	TFEB_HUMAN	Transcription factor EB	S459	16%	0.16	2	0	0	0	0	1	0	0	0	1	0	0	0	2
3513	TFEB_HUMAN	Transcription factor EB	T331	25%	0.25	2	0	0	0	0	0	0	0	0	1	0	0	1	2
3514	JUND_HUMAN	Transcription factor jun-D	S251	45%	0.45	3	0	1	0	1	0	0	0	0	0	0	1	0	3
3515	JUND_HUMAN	Transcription factor jun-D	S255	100%	1.00	28	0	1	2	1	3	0	1	0	0	2	3	0	13
3516	JUND_HUMAN	Transcription factor jun-D	S259	100%	1.00	31	0	2	2	2	3	0	1	0	0	2	4	0	16
3517	JUND_HUMAN	Transcription factor jun-D	S43	34%	0.34	1	0	0	0	0	0	0	0	0	1	0	0	0	1
3518	JUND_HUMAN	Transcription factor jun-D	T3	30%	0.30	1	0	0	0	0	0	0	0	0	1	0	0	0	1
3519	JUND_HUMAN	Transcription factor jun-D	Y6	30%	0.30	1	0	0	0	0	0	0	0	0	1	0	0	0	1

3520	SOX1_HUMAN	Transcription factor SOX-1	S12	83%	0.83	3	0	0	0	0	0	0	0	0	0	0	0	1	1
3521	SOX1_HUMAN	Transcription factor SOX-1	S23	83%	0.83	4	0	0	0	0	0	0	0	0	0	0	0	2	2
3522	SOX1_HUMAN	Transcription factor SOX-1	S3	72%	0.72	2	0	0	0	0	0	0	0	0	0	0	0	2	2
3523	SOX1_HUMAN	Transcription factor SOX-1	T20	51%	0.51	2	0	0	0	0	0	0	0	0	0	0	0	1	1
3524	SOX1_HUMAN	Transcription factor SOX-1	T8	92%	0.92	3	0	0	0	0	0	0	0	0	0	0	0	1	1
3525	SOX2_HUMAN	Transcription factor SOX-2	S25	43%	0.43	1	0	0	1	0	0	0	0	0	0	0	0	0	1
3526	SOX2_HUMAN	Transcription factor SOX-2	S37	43%	0.43	1	0	0	1	0	0	0	0	0	0	0	0	0	1
3527	SOX2_HUMAN	Transcription factor SOX-2	T17	22%	0.22	2	0	0	0	1	0	0	0	0	0	0	0	0	1
3528	SOX2_HUMAN	Transcription factor SOX-2	T26	43%	0.43	1	0	0	1	0	0	0	0	0	0	0	0	0	1
3529	SOX2_HUMAN	Transcription factor SOX-2	T7	62%	0.62	2	0	0	0	1	0	0	0	0	0	0	0	0	1
3530	SP1_HUMAN	Transcription factor Sp1	S2	100%	1.00	8	0	0	1	0	1	0	0	0	0	0	0	0	2
3531	TAF12_HUMAN	Transcription initiation factor TFIID subunit 12	S51	100%	1.00	27	3	3	2	0	1	0	4	1	0	0	1	0	15
3532	SUPT3_HUMAN	Transcription initiation protein SPT3 homolog	S7	80%	0.80	1	1	0	0	0	0	0	0	0	0	0	0	0	1
3533	TIF1B_HUMAN	Transcription intermediary factor 1-beta	S17	99%	0.99	177	4	5	6	4	2	3	4	4	7	5	5	4	53
3534	TIF1B_HUMAN	Transcription intermediary factor 1-beta	S19	100%	1.00	####	55	55	48	55	46	53	57	51	53	39	46	42	600
3535	TIF1B_HUMAN	Transcription intermediary factor 1-beta	S26	100%	1.00	212	6	5	7	5	4	4	8	6	4	1	3	3	56
3536	TIF1B_HUMAN	Transcription intermediary factor 1-beta	S471	3%	0.03	1	0	0	0	0	0	0	0	0	0	0	1	0	1
3537	TIF1B_HUMAN	Transcription intermediary factor 1-beta	S473	100%	1.00	7	0	1	0	0	0	0	0	0	0	1	1	2	5
3538	TIF1B_HUMAN	Transcription intermediary factor 1-beta	S49	75%	0.75	18	1	0	0	0	0	0	1	1	2	0	0	0	5

3539	TIF1B_HUMAN	Transcription intermediary factor 1-beta	S50	99%	0.99	271	5	5	6	5	5	7	5	4	4	5	3	2	56
3540	TIF1B_HUMAN	Transcription intermediary factor 1-beta	S594	98%	0.98	324	10	5	5	6	5	7	5	3	5	2	3	4	60
3541	TIF1B_HUMAN	Transcription intermediary factor 1-beta	S596	64%	0.64	35	1	0	0	0	1	0	0	0	0	0	0	1	3
3542	TIF1B_HUMAN	Transcription intermediary factor 1-beta	S598	75%	0.75	9	0	0	0	1	0	0	0	0	0	0	0	0	1
3543	TIF1B_HUMAN	Transcription intermediary factor 1-beta	S600	39%	0.39	13	0	0	1	0	0	0	0	0	0	0	0	0	1
3544	TIF1B_HUMAN	Transcription intermediary factor 1-beta	S601	53%	0.53	16	1	0	2	0	0	0	0	0	0	0	1	0	4
3545	PURB_HUMAN	Transcriptional activator protein Pur-beta	S100	0%	0.00	1	0	0	0	0	0	0	0	0	0	0	1	0	1
3546	PURB_HUMAN	Transcriptional activator protein Pur-beta	S101	100%	1.00	15	2	0	2	0	0	0	0	0	1	1	1	0	7
3547	TEAD1_HUMAN	Transcriptional enhancer factor TEF-1	S323	40%	0.40	1	0	0	0	0	1	0	0	0	0	0	0	0	1
3548	TEAD1_HUMAN	Transcriptional enhancer factor TEF-1	T309	42%	0.42	1	0	0	0	0	1	0	0	0	0	0	0	0	1
3549	TEAD1_HUMAN	Transcriptional enhancer factor TEF-1	T324	40%	0.40	1	0	0	0	0	1	0	0	0	0	0	0	0	1
3550	TEAD1_HUMAN	Transcriptional enhancer factor TEF-1	Y312	40%	0.40	1	0	0	0	0	1	0	0	0	0	0	0	0	1
3551	ATRX_HUMAN	Transcriptional regulator ATRX	S634	100%	1.00	6	0	0	0	0	1	0	1	0	1	0	0	0	3
3552	ATRX_HUMAN	Transcriptional regulator ATRX	S677	100%	1.00	18	0	0	0	0	0	1	3	0	1	0	0	0	5
3553	ATRX_HUMAN	Transcriptional regulator ATRX	T684	12%	0.12	1	0	0	0	0	1	0	0	0	0	0	0	0	1
3554	P66B_HUMAN	Transcriptional repressor p66-beta	S334	17%	0.17	1	0	0	0	0	0	0	0	0	0	0	1	0	1
3555	TBL2_HUMAN	Transducin beta-like protein 2	S13	75%	0.75	1	0	0	0	0	0	0	1	0	0	0	0	0	1
3556	TBL2_HUMAN	Transducin beta-like protein 2	S7	75%	0.75	1	0	0	0	0	0	0	1	0	0	0	0	0	1

3557	TBL2_HUMAN	Transducin beta-like protein 2	T24	75%	0.75	1	0	0	0	0	0	0	1	0	0	0	0	0	1
3558	TRA2A_HUMAN	Transformer-2 protein homolog alpha	T204	8%	0.08	1	0	0	0	0	1	0	0	0	0	0	0	0	1
3559	TACC2_HUMAN	Transforming acidic coiled-coil-containing protein 2	S2317	100%	1.00	27	0	0	2	0	0	0	0	1	0	0	0	1	4
3560	TACC2_HUMAN	Transforming acidic coiled-coil-containing protein 2	S2321	100%	1.00	22	0	0	0	0	0	0	0	1	0	0	0	1	2
3561	TACC2_HUMAN	Transforming acidic coiled-coil-containing protein 2	S453	100%	1.00	1	0	0	0	0	0	0	0	0	0	1	0	0	1
3562	TACC3_HUMAN	Transforming acidic coiled-coil-containing protein 3	S250	100%	1.00	58	0	0	0	0	0	0	0	0	0	4	6	6	16
3563	TACC3_HUMAN	Transforming acidic coiled-coil-containing protein 3	T249	97%	0.97	3	0	0	0	0	0	0	0	0	0	0	1	0	1
3564	BGH3_HUMAN	Transforming growth factor-beta-induced protein ig-h3	T454	95%	0.95	1	0	0	0	1	0	0	0	0	0	0	0	0	1
3565	BGH3_HUMAN	Transforming growth factor-beta-induced protein ig-h3	T457	88%	0.88	1	0	0	0	1	0	0	0	0	0	0	0	0	1
3566	BGH3_HUMAN	Transforming growth factor-beta-induced protein ig-h3	Y448	45%	0.45	1	0	0	0	1	0	0	0	0	0	0	0	0	1
3567	TGON2_HUMAN	Trans-Golgi network integral membrane protein 2	S25	100%	1.00	5	0	0	0	0	1	2	0	0	0	0	0	0	3
3568	TGON2_HUMAN	Trans-Golgi network integral membrane protein 2	T23	100%	1.00	5	0	0	0	0	1	2	0	0	0	0	0	0	3
3569	TERA_HUMAN	Transitional endoplasmic reticulum ATPase	S3	98%	0.98	2	0	0	0	0	0	0	0	0	1	0	1	0	2
3570	EI2BD_HUMAN	Translation initiation factor eIF-2B subunit delta	S328	25%	0.25	1	0	0	0	0	1	0	0	0	0	0	0	0	1
3571	EI2BD_HUMAN	Translation initiation factor eIF-2B subunit delta	S343	18%	0.18	1	0	0	0	0	1	0	0	0	0	0	0	0	1
3572	EI2BE_HUMAN	Translation initiation factor eIF-2B subunit epsilon	S532	14%	0.14	1	0	0	0	0	0	0	0	0	0	0	0	1	1
3573	EI2BE_HUMAN	Translation initiation factor eIF-2B subunit epsilon	S540	76%	0.76	1	0	0	0	0	0	0	0	0	0	0	0	1	1

3574	SSRA_HUMAN	Translocon-associated protein subunit alpha	S268	44%	0.44	19	0	2	0	0	1	0	0	1	1	2	2	0	9
3575	SSRA_HUMAN	Translocon-associated protein subunit alpha	T260	94%	0.94	7	0	1	0	0	0	0	0	2	0	1	0	1	5
3576	TMBI4_HUMAN	Transmembrane BAX inhibitor motif-containing protein 4	S69	87%	0.87	1	0	1	0	0	0	0	0	0	0	0	0	0	1
3577	TM115_HUMAN	Transmembrane protein 115	T329	100%	1.00	7	0	1	0	0	1	0	0	0	0	1	0	0	3
3578	TM131_HUMAN	Transmembrane protein 131	S1606	30%	0.30	1	0	0	0	0	0	0	0	0	0	0	0	1	1
3579	TM131_HUMAN	Transmembrane protein 131	S1619	31%	0.31	1	0	0	0	0	0	0	0	0	0	0	0	1	1
3580	TM131_HUMAN	Transmembrane protein 131	S1621	30%	0.30	1	0	0	0	0	0	0	0	0	0	0	0	1	1
3581	TM131_HUMAN	Transmembrane protein 131	S1622	30%	0.30	1	0	0	0	0	0	0	0	0	0	0	0	1	1
3582	TM131_HUMAN	Transmembrane protein 131	S1626	30%	0.30	1	0	0	0	0	0	0	0	0	0	0	0	1	1
3583	T132A_HUMAN	Transmembrane protein 132A	S529	100%	1.00	71	4	5	2	3	3	1	4	4	1	0	0	0	27
3584	T151B_HUMAN	Transmembrane protein 151B	S11	55%	0.55	1	0	0	0	0	0	0	0	0	1	0	0	0	1
3585	T151B_HUMAN	Transmembrane protein 151B	S25	49%	0.49	1	0	0	0	0	0	0	0	0	1	0	0	0	1
3586	T151B_HUMAN	Transmembrane protein 151B	T29	61%	0.61	1	0	0	0	0	0	0	0	0	1	0	0	0	1
3587	TMM17_HUMAN	Transmembrane protein 17	S22	82%	0.82	8	0	0	0	0	0	0	0	0	0	0	1	2	3
3588	T170B_HUMAN	Transmembrane protein 170B	S14	34%	0.34	1	0	0	0	0	0	0	0	0	0	0	0	1	1
3589	T170B_HUMAN	Transmembrane protein 170B	S20	66%	0.66	1	0	0	0	0	0	0	0	0	0	0	0	1	1
3590	T183A_HUMAN	Transmembrane protein 183A	S183	100%	1.00	4	0	0	1	0	0	2	0	1	0	0	0	0	4
3591	T183A_HUMAN	Transmembrane protein 183A	T179	100%	1.00	4	0	0	1	0	0	2	0	1	0	0	0	0	4
3592	T183A_HUMAN	Transmembrane protein 183A	Y178	100%	1.00	4	0	0	1	0	0	2	0	1	0	0	0	0	4

3593	T183B_HUMAN	Transmembrane protein 183B	S183	100%	1.00	4	0	0	1	0	0	2	0	1	0	0	0	0	4
3594	T183B_HUMAN	Transmembrane protein 183B	T179	100%	1.00	4	0	0	1	0	0	2	0	1	0	0	0	0	4
3595	T183B_HUMAN	Transmembrane protein 183B	Y178	100%	1.00	4	0	0	1	0	0	2	0	1	0	0	0	0	4
3596	TM201_HUMAN	Transmembrane protein 201	S450	32%	0.32	3	0	0	2	0	0	0	0	0	0	0	0	0	2
3597	TM201_HUMAN	Transmembrane protein 201	S454	17%	0.17	1	0	0	1	0	0	0	0	0	0	0	0	0	1
3598	TM206_HUMAN	Transmembrane protein 206	S53	33%	0.33	1	0	0	0	0	1	0	0	0	0	0	0	0	1
3599	TM206_HUMAN	Transmembrane protein 206	S54	33%	0.33	1	0	0	0	0	1	0	0	0	0	0	0	0	1
3600	TM231_HUMAN	Transmembrane protein 231	S155	30%	0.30	1	0	0	0	0	0	0	1	0	0	0	0	0	1
3601	TM231_HUMAN	Transmembrane protein 231	S168	97%	0.97	1	0	0	0	0	0	0	1	0	0	0	0	0	1
3602	TM231_HUMAN	Transmembrane protein 231	T150	24%	0.24	1	0	0	0	0	0	0	1	0	0	0	0	0	1
3603	TMM40_HUMAN	Transmembrane protein 40	S137	100%	1.00	11	0	0	0	0	0	0	0	0	0	2	1	0	3
3604	TMM40_HUMAN	Transmembrane protein 40	S141	98%	0.98	3	0	0	0	0	0	0	0	0	0	0	0	1	1
3605	TM87A_HUMAN	Transmembrane protein 87A	S540	100%	1.00	99	1	1	0	1	1	0	1	2	0	0	2	3	12
3606	TNPO3_HUMAN	Transportin-3	S158	60%	0.60	1	0	0	0	0	0	0	0	0	0	0	0	1	1
3607	TNPO3_HUMAN	Transportin-3	T148	77%	0.77	1	0	0	0	0	0	0	0	0	0	0	0	1	1
3608	TNPO3_HUMAN	Transportin-3	Y133	19%	0.19	1	0	0	0	0	0	0	0	0	0	0	0	1	1
3609	TCOF_HUMAN	Treacle protein	S1228	100%	1.00	148	3	5	3	4	3	3	3	2	3	5	5	3	42
3610	TCOF_HUMAN	Treacle protein	S1230	9%	0.09	2	0	0	1	0	0	0	0	1	0	0	0	0	2
3611	TCOF_HUMAN	Treacle protein	S1350	100%	1.00	11	0	0	0	0	0	0	0	0	0	4	0	7	11
3612	TCOF_HUMAN	Treacle protein	S1376	13%	0.13	4	0	0	0	0	0	0	0	1	0	0	1	0	2

3613	TCOF_HUMAN	Treacle protein	S1378	100%	1.00	246	6	10	11	9	11	8	7	3	9	6	5	8	93
3614	TCOF_HUMAN	Treacle protein	S1386	14%	0.14	4	1	0	1	0	1	0	0	0	0	0	1	0	4
3615	TCOF_HUMAN	Treacle protein	S503	100%	1.00	6	0	0	0	0	0	0	0	0	0	0	1	2	3
3616	TCOF_HUMAN	Treacle protein	S906	100%	1.00	15	0	0	0	0	0	0	0	0	0	6	4	5	15
3617	TNC18_HUMAN	Trinucleotide repeat-containing gene 18 protein	S1857	100%	1.00	11	0	2	0	0	1	0	0	0	0	2	0	0	5
3618	TNR6A_HUMAN	Trinucleotide repeat-containing gene 6A protein	S1561	90%	0.90	1	0	0	0	0	0	1	0	0	0	0	0	0	1
3619	TNR6A_HUMAN	Trinucleotide repeat-containing gene 6A protein	S1562	90%	0.90	1	0	0	0	0	0	1	0	0	0	0	0	0	1
3620	TNR6A_HUMAN	Trinucleotide repeat-containing gene 6A protein	S1569	85%	0.85	1	0	0	0	0	0	1	0	0	0	0	0	0	1
3621	TNR6A_HUMAN	Trinucleotide repeat-containing gene 6A protein	S1579	50%	0.50	1	0	0	0	0	0	1	0	0	0	0	0	0	1
3622	TNR6A_HUMAN	Trinucleotide repeat-containing gene 6A protein	S1580	40%	0.40	1	0	0	0	0	0	1	0	0	0	0	0	0	1
3623	TNR6A_HUMAN	Trinucleotide repeat-containing gene 6A protein	T1581	63%	0.63	1	0	0	0	0	0	1	0	0	0	0	0	0	1
3624	TPIS_HUMAN	Triosephosphate isomerase	S21	100%	1.00	####	89	77	80	69	56	54	68	56	54	56	49	51	759
3625	TPIS_HUMAN	Triosephosphate isomerase	S80	89%	0.89	7	0	1	0	0	0	0	0	0	0	1	0	1	3
3626	TPIS_HUMAN	Triosephosphate isomerase	T28	0%	0.00	1	0	0	1	0	0	0	0	0	0	0	0	0	1
3627	TRI29_HUMAN	Tripartite motif-containing protein 29	S23	20%	0.20	2	0	0	0	0	0	0	0	0	0	1	0	1	2
3628	TRI47_HUMAN	Tripartite motif-containing protein 47	S588	100%	1.00	29	0	0	0	0	0	1	0	0	0	0	3	3	7
3629	TRIO_HUMAN	Triple functional domain protein	S1905	28%	0.28	3	0	0	0	0	0	0	0	0	0	0	3	0	3
3630	TRM1L_HUMAN	TRMT1-like protein	S66	52%	0.52	2	0	0	0	0	0	0	0	0	0	0	0	1	1

3631	TRM1L_HUMAN	TRMT1-like protein	T26	100%	1.00	1	0	0	0	0	0	0	0	0	1	0	0	0	1
3632	NSUN2_HUMAN	tRNA (cytosine(34)-C(5))-methyltransferase	S743	100%	1.00	####	31	39	37	27	28	25	26	31	22	26	22	24	338
3633	NSUN2_HUMAN	tRNA (cytosine(34)-C(5))-methyltransferase	S751	100%	1.00	736	21	24	22	18	17	14	16	14	14	10	14	11	195
3634	TRMT5_HUMAN	tRNA (guanine-N(1)-)-methyltransferase	S256	100%	1.00	1	0	0	0	0	0	0	0	0	0	0	1	0	1
3635	TRMT5_HUMAN	tRNA (guanine-N(1)-)-methyltransferase	T263	100%	1.00	1	0	0	0	0	0	0	0	0	0	0	1	0	1
3636	WDR4_HUMAN	tRNA (guanine-N(7)-)-methyltransferase subunit WDR4	S20	57%	0.57	1	0	0	0	0	0	0	1	0	0	0	0	0	1
3637	WDR4_HUMAN	tRNA (guanine-N(7)-)-methyltransferase subunit WDR4	S4	68%	0.68	1	0	0	0	0	0	0	1	0	0	0	0	0	1
3638	TRM2A_HUMAN	tRNA (uracil-5-)-methyltransferase homolog A	S602	69%	0.69	2	0	0	0	0	0	0	0	0	0	2	0	0	2
3639	TYW2_HUMAN	tRNA wybutosine-synthesizing protein 2 homolog	S8	100%	1.00	6	0	1	1	0	0	0	0	0	0	1	1	0	4
3640	SYWC_HUMAN	Tryptophanyl-tRNA synthetase, cytoplasmic	S4	66%	0.66	7	0	2	0	0	0	0	0	0	0	3	1	0	6
3641	SYWC_HUMAN	Tryptophanyl-tRNA synthetase, cytoplasmic	S8	15%	0.15	1	0	0	0	0	1	0	0	0	0	0	0	0	1
3642	SYWM_HUMAN	Tryptophanyl-tRNA synthetase, mitochondrial	S173	100%	1.00	27	0	0	0	2	0	0	0	0	0	0	0	0	2
3643	SYWM_HUMAN	Tryptophanyl-tRNA synthetase, mitochondrial	T174	100%	1.00	27	0	0	0	2	0	0	0	0	0	0	0	0	2
3644	TULP1_HUMAN	Tubby-related protein 1	T192	100%	1.00	1	0	0	0	0	0	0	0	0	0	1	0	0	1
3645	TBA1B_HUMAN	Tubulin alpha-1B chain	S340	100%	1.00	8	3	0	0	1	0	0	0	0	0	0	0	0	4
3646	TBA1B_HUMAN	Tubulin alpha-1B chain	S48	93%	0.93	9	1	0	0	2	1	0	0	0	0	0	0	0	4
3647	TBA1C_HUMAN	Tubulin alpha-1C chain	S48	93%	0.93	9	1	0	0	2	1	0	0	0	0	0	0	0	4

3648	TTL12_HUMAN	Tubulin--tyrosine ligase-like protein 12	S15	92%	0.92	9	0	1	1	0	0	0	0	0	0	0	0	0	2
3649	TTL12_HUMAN	Tubulin--tyrosine ligase-like protein 12	S16	97%	0.97	240	4	6	4	4	3	4	5	3	6	3	8	6	56
3650	TDRD3_HUMAN	Tudor domain-containing protein 3	S256	100%	1.00	40	2	0	0	0	0	0	0	0	1	5	3	1	12
3651	TDRD5_HUMAN	Tudor domain-containing protein 5	S164	58%	0.58	1	0	0	0	0	0	0	0	0	0	0	0	1	1
3652	TDRD5_HUMAN	Tudor domain-containing protein 5	S174	94%	0.94	1	0	0	0	0	0	0	0	0	0	0	0	1	1
3653	TDRD5_HUMAN	Tudor domain-containing protein 5	S183	86%	0.86	1	0	0	0	0	0	0	0	0	0	0	0	1	1
3654	TDRD5_HUMAN	Tudor domain-containing protein 5	S187	89%	0.89	1	0	0	0	0	0	0	0	0	0	0	0	1	1
3655	TDRD5_HUMAN	Tudor domain-containing protein 5	Y167	30%	0.30	1	0	0	0	0	0	0	0	0	0	0	0	1	1
3656	TDRD7_HUMAN	Tudor domain-containing protein 7	S862	17%	0.17	1	0	0	0	0	0	0	0	0	0	1	0	0	1
3657	TFP11_HUMAN	Tuftelin-interacting protein 11	S98	100%	1.00	1	0	0	0	0	0	0	1	0	0	0	0	0	1
3658	TNR3_HUMAN	Tumor necrosis factor receptor superfamily member 3	S298	66%	0.66	2	0	0	0	0	0	0	0	0	0	0	1	0	1
3659	TNR3_HUMAN	Tumor necrosis factor receptor superfamily member 3	S305	15%	0.15	4	0	0	0	0	0	0	0	0	0	0	1	0	1
3660	TNF6B_HUMAN	Tumor necrosis factor receptor superfamily member 6B	S291	100%	1.00	12	1	4	3	0	1	0	0	0	0	0	0	0	9
3661	P63_HUMAN	Tumor protein 63	S395	100%	1.00	1	1	0	0	0	0	0	0	0	0	0	0	0	1
3662	P63_HUMAN	Tumor protein 63	S624	52%	0.52	1	0	1	0	0	0	0	0	0	0	0	0	0	1
3663	P63_HUMAN	Tumor protein 63	S627	40%	0.40	1	0	1	0	0	0	0	0	0	0	0	0	0	1
3664	P63_HUMAN	Tumor protein 63	T625	30%	0.30	1	0	1	0	0	0	0	0	0	0	0	0	0	1
3665	TPD54_HUMAN	Tumor protein D54	S12	100%	1.00	150	3	3	3	4	3	4	0	1	0	3	4	2	30
3666	TPD54_HUMAN	Tumor protein D54	S166	98%	0.98	1	0	0	1	0	0	0	0	0	0	0	0	0	1

3667	TPD54_HUMAN	Tumor protein D54	S21	100%	1.00	41	0	0	1	1	0	0	0	0	0	0	0	0	2
3668	TPD54_HUMAN	Tumor protein D54	S3	35%	0.35	6	0	0	0	0	0	0	0	0	0	0	1	1	2
3669	TPD54_HUMAN	Tumor protein D54	T163	2%	0.02	4	1	2	0	0	0	0	0	0	0	0	0	0	3
3670	TP53B_HUMAN	Tumor suppressor p53-binding protein 1	S1113	80%	0.80	5	1	1	1	0	1	0	0	0	0	0	0	0	4
3671	TP53B_HUMAN	Tumor suppressor p53-binding protein 1	S1114	100%	1.00	115	1	4	6	3	3	4	6	5	4	3	2	1	42
3672	TP53B_HUMAN	Tumor suppressor p53-binding protein 1	S1462	81%	0.81	31	2	3	0	2	0	1	5	1	1	1	1	1	18
3673	TP53B_HUMAN	Tumor suppressor p53-binding protein 1	S294	100%	1.00	5	0	0	0	1	0	0	0	0	1	0	0	0	2
3674	TP53B_HUMAN	Tumor suppressor p53-binding protein 1	S380	92%	0.92	29	2	2	0	1	0	0	1	3	2	0	1	2	14
3675	TP53B_HUMAN	Tumor suppressor p53-binding protein 1	S500	100%	1.00	9	0	0	0	1	0	0	1	0	0	0	0	0	2
3676	TP53B_HUMAN	Tumor suppressor p53-binding protein 1	S552	100%	1.00	8	0	0	0	3	0	0	4	0	0	0	0	0	7
3677	TP53B_HUMAN	Tumor suppressor p53-binding protein 1	T382	15%	0.15	8	0	0	0	0	0	0	0	0	0	2	0	0	2
3678	ABL1_HUMAN	Tyrosine-protein kinase ABL1	S569	100%	1.00	4	1	0	0	0	0	0	0	0	0	0	0	0	1
3679	BLK_HUMAN	Tyrosine-protein kinase Blk	S163	100%	1.00	1	0	0	0	0	0	0	0	0	0	0	0	1	1
3680	FER_HUMAN	Tyrosine-protein kinase Fer	T58	35%	0.35	1	0	0	0	0	1	0	0	0	0	0	0	0	1
3681	FER_HUMAN	Tyrosine-protein kinase Fer	Y42	35%	0.35	1	0	0	0	0	1	0	0	0	0	0	0	0	1
3682	FER_HUMAN	Tyrosine-protein kinase Fer	Y63	54%	0.54	1	0	0	0	0	1	0	0	0	0	0	0	0	1
3683	PTN12_HUMAN	Tyrosine-protein phosphatase non-receptor type 12	S323	24%	0.24	3	0	0	0	0	0	0	0	0	0	0	1	0	1
3684	PTN12_HUMAN	Tyrosine-protein phosphatase non-receptor type 12	S324	25%	0.25	15	1	1	0	0	0	0	0	0	0	0	1	0	3

3685	PTN12_HUMAN	Tyrosine-protein phosphatase non-receptor type 12	S332	100%	1.00	104	1	2	2	4	7	0	0	3	2	7	4	2	34
3686	PTN12_HUMAN	Tyrosine-protein phosphatase non-receptor type 12	S661	11%	0.11	5	0	0	0	0	0	0	0	1	0	1	0	0	2
3687	PTN12_HUMAN	Tyrosine-protein phosphatase non-receptor type 12	S670	27%	0.27	11	0	1	0	0	0	0	0	0	0	0	0	1	2
3688	PTN12_HUMAN	Tyrosine-protein phosphatase non-receptor type 12	S673	100%	1.00	198	5	3	1	10	8	8	4	7	4	4	6	7	67
3689	PTN12_HUMAN	Tyrosine-protein phosphatase non-receptor type 12	T318	74%	0.74	6	0	0	0	0	1	0	0	0	0	0	0	1	2
3690	PTN12_HUMAN	Tyrosine-protein phosphatase non-receptor type 12	T509	95%	0.95	52	0	0	0	0	1	0	1	1	0	0	2	2	7
3691	PTN13_HUMAN	Tyrosine-protein phosphatase non-receptor type 13	S345	33%	0.33	6	0	0	0	1	0	2	0	0	0	0	1	1	5
3692	PTN13_HUMAN	Tyrosine-protein phosphatase non-receptor type 13	S348	33%	0.33	1	0	0	0	0	0	1	0	0	0	0	0	0	1
3693	PTN14_HUMAN	Tyrosine-protein phosphatase non-receptor type 14	S642	100%	1.00	2	0	0	0	2	0	0	0	0	0	0	0	0	2
3694	PTN23_HUMAN	Tyrosine-protein phosphatase non-receptor type 23	S1126	89%	0.89	2	0	1	1	0	0	0	0	0	0	0	0	0	2
3695	RU17_HUMAN	U1 small nuclear ribonucleoprotein 70 kDa	S226	100%	1.00	355	10	12	13	11	12	12	12	10	10	12	12	11	137
3696	MPP10_HUMAN	U3 small nucleolar ribonucleoprotein protein MPP10	S242	100%	1.00	2	0	0	0	1	0	0	0	0	0	0	0	0	1
3697	UT14A_HUMAN	U3 small nucleolar RNA-associated protein 14 homolog A	S341	91%	0.91	1	0	1	0	0	0	0	0	0	0	0	0	0	1
3698	UT14A_HUMAN	U3 small nucleolar RNA-associated protein 14 homolog A	S343	95%	0.95	1	0	1	0	0	0	0	0	0	0	0	0	0	1
3699	U520_HUMAN	U5 small nuclear ribonucleoprotein 200 kDa helicase	S225	100%	1.00	261	7	8	8	9	8	6	8	6	6	7	5	6	84
3700	UIF_HUMAN	UAP56-interacting factor	S15	12%	0.12	1	0	0	0	0	0	0	0	0	0	0	0	1	1

3701	UIF_HUMAN	UAP56-interacting factor	S16	98%	0.98	4	0	0	0	0	0	0	0	0	0	3	1	0	4
3702	UBN1_HUMAN	Ubinuclein-1	S1012	80%	0.80	1	0	0	0	0	1	0	0	0	0	0	0	0	1
3703	UBN1_HUMAN	Ubinuclein-1	S1025	22%	0.22	1	0	0	1	0	0	0	0	0	0	0	0	0	1
3704	UBN1_HUMAN	Ubinuclein-1	S990	100%	1.00	1	0	0	1	0	0	0	0	0	0	0	0	0	1
3705	UBN1_HUMAN	Ubinuclein-1	S992	36%	0.36	1	0	0	1	0	0	0	0	0	0	0	0	0	1
3706	UBN1_HUMAN	Ubinuclein-1	S996	36%	0.36	1	0	0	1	0	0	0	0	0	0	0	0	0	1
3707	UBN1_HUMAN	Ubinuclein-1	S999	49%	0.49	1	0	0	1	0	0	0	0	0	0	0	0	0	1
3708	UBN1_HUMAN	Ubinuclein-1	T989	100%	1.00	1	0	0	1	0	0	0	0	0	0	0	0	0	1
3709	UBP10_HUMAN	Ubiquitin carboxyl-terminal hydrolase 10	S365	25%	0.25	9	0	0	0	0	0	0	0	1	1	0	0	0	2
3710	UBP10_HUMAN	Ubiquitin carboxyl-terminal hydrolase 10	S5	90%	0.90	8	0	0	0	0	0	0	0	0	0	2	2	2	6
3711	UBP10_HUMAN	Ubiquitin carboxyl-terminal hydrolase 10	S576	100%	1.00	29	1	4	2	0	0	0	5	1	4	0	0	0	17
3712	UBP14_HUMAN	Ubiquitin carboxyl-terminal hydrolase 14	S143	100%	1.00	256	2	6	4	7	6	5	4	5	5	13	13	19	89
3713	UBP14_HUMAN	Ubiquitin carboxyl-terminal hydrolase 14	S148	59%	0.59	1	0	0	0	0	0	0	0	0	0	0	1	0	1
3714	UBP15_HUMAN	Ubiquitin carboxyl-terminal hydrolase 15	S229	100%	1.00	20	0	0	0	0	0	2	0	0	0	0	0	0	2
3715	UBP16_HUMAN	Ubiquitin carboxyl-terminal hydrolase 16	S552	100%	1.00	23	0	1	1	2	1	0	1	0	0	0	2	0	8
3716	UBP16_HUMAN	Ubiquitin carboxyl-terminal hydrolase 16	T532	22%	0.22	5	0	0	0	1	0	0	0	0	0	0	0	0	1
3717	UBP16_HUMAN	Ubiquitin carboxyl-terminal hydrolase 16	T554	5%	0.05	1	0	0	0	0	0	0	0	0	0	1	0	0	1
3718	UBP24_HUMAN	Ubiquitin carboxyl-terminal hydrolase 24	S2561	20%	0.20	6	0	0	0	0	0	0	1	0	0	0	0	0	1

3719	UBP24_HUMAN	Ubiquitin carboxyl-terminal hydrolase 24	S2604	97%	0.97	1	0	0	0	0	0	0	1	0	0	0	0	0	1
3720	UBP24_HUMAN	Ubiquitin carboxyl-terminal hydrolase 24	T2559	20%	0.20	2	0	0	0	0	0	0	1	0	0	0	0	0	1
3721	UBP36_HUMAN	Ubiquitin carboxyl-terminal hydrolase 36	S716	28%	0.28	1	0	0	0	0	1	0	0	0	0	0	0	0	1
3722	UBP7_HUMAN	Ubiquitin carboxyl-terminal hydrolase 7	S18	100%	1.00	27	3	4	1	0	2	2	1	0	1	0	0	0	14
3723	BAP1_HUMAN	Ubiquitin carboxyl-terminal hydrolase BAP1	S327	88%	0.88	11	2	2	0	2	0	0	2	0	1	0	0	0	9
3724	UCHL3_HUMAN	Ubiquitin carboxyl-terminal hydrolase isozyme L3	S128	63%	0.63	2	0	0	0	0	0	1	0	0	0	0	0	0	1
3725	UCHL3_HUMAN	Ubiquitin carboxyl-terminal hydrolase isozyme L3	S130	100%	1.00	26	0	0	0	0	0	1	0	1	0	0	0	2	4
3726	UBE4B_HUMAN	Ubiquitin conjugation factor E4 B	S213	99%	0.99	1	0	0	0	0	0	0	0	0	1	0	0	0	1
3727	UBE4B_HUMAN	Ubiquitin conjugation factor E4 B	S229	60%	0.60	1	0	0	0	0	0	0	0	0	1	0	0	0	1
3728	UBE4B_HUMAN	Ubiquitin conjugation factor E4 B	T214	99%	0.99	1	0	0	0	0	0	0	0	0	1	0	0	0	1
3729	UBE4B_HUMAN	Ubiquitin conjugation factor E4 B	T216	100%	1.00	1	0	0	0	0	0	0	0	0	1	0	0	0	1
3730	UBE4B_HUMAN	Ubiquitin conjugation factor E4 B	T226	85%	0.85	1	0	0	0	0	0	0	0	0	1	0	0	0	1
3731	UBE4B_HUMAN	Ubiquitin conjugation factor E4 B	T228	60%	0.60	1	0	0	0	0	0	0	0	0	1	0	0	0	1
3732	UFD1_HUMAN	Ubiquitin fusion degradation protein 1 homolog	S247	100%	1.00	17	0	0	0	0	0	0	0	0	0	0	0	1	1
3733	UBAP2_HUMAN	Ubiquitin-associated protein 2	S633	50%	0.50	1	0	0	0	0	0	1	0	0	0	0	0	0	1
3734	UBAP2_HUMAN	Ubiquitin-associated protein 2	S634	50%	0.50	1	0	0	0	0	0	1	0	0	0	0	0	0	1
3735	UBAP2_HUMAN	Ubiquitin-associated protein 2	S635	50%	0.50	1	0	0	0	0	0	1	0	0	0	0	0	0	1
3736	UBAP2_HUMAN	Ubiquitin-associated protein 2	S637	94%	0.94	1	0	0	0	0	0	1	0	0	0	0	0	0	1
3737	UBAP2_HUMAN	Ubiquitin-associated protein 2	S651	83%	0.83	1	0	0	0	0	0	1	0	0	0	0	0	0	1

3738	UBAP2_HUMAN	Ubiquitin-associated protein 2	T641	100%	1.00	1	0	0	0	0	0	1	0	0	0	0	0	0	1
3739	UBAP2_HUMAN	Ubiquitin-associated protein 2	Y628	84%	0.84	1	0	0	0	0	0	1	0	0	0	0	0	0	1
3740	UBP2L_HUMAN	Ubiquitin-associated protein 2-like	S453	78%	0.78	22	1	0	0	1	1	0	0	0	0	0	2	1	6
3741	UBP2L_HUMAN	Ubiquitin-associated protein 2-like	S454	98%	0.98	173	3	4	3	3	4	3	3	4	3	6	6	4	46
3742	UBP2L_HUMAN	Ubiquitin-associated protein 2-like	S458	6%	0.06	3	0	0	0	0	0	1	1	0	0	0	1	0	3
3743	UB2J1_HUMAN	Ubiquitin-conjugating enzyme E2 J1	S266	95%	0.95	17	0	1	0	0	0	0	3	1	0	2	2	0	9
3744	UB2J1_HUMAN	Ubiquitin-conjugating enzyme E2 J1	S268	33%	0.33	21	0	2	1	1	0	0	3	2	1	3	3	0	16
3745	UB2J1_HUMAN	Ubiquitin-conjugating enzyme E2 J1	T267	33%	0.33	3	0	0	0	0	0	0	0	0	0	0	0	1	1
3746	UBE2O_HUMAN	Ubiquitin-conjugating enzyme E2 O	S839	99%	0.99	86	0	2	2	2	3	1	0	2	1	2	3	4	22
3747	UBE2O_HUMAN	Ubiquitin-conjugating enzyme E2 O	T841	7%	0.07	1	0	0	0	0	0	0	0	0	0	1	0	0	1
3748	UBA1_HUMAN	Ubiquitin-like modifier-activating enzyme 1	S46	100%	1.00	7	0	0	0	1	0	0	1	0	0	4	1	0	7
3749	UBL7_HUMAN	Ubiquitin-like protein 7	S230	100%	1.00	40	0	0	0	0	0	0	3	1	1	0	0	0	5
3750	UBXN1_HUMAN	UBX domain-containing protein 1	S182	43%	0.43	1	0	0	0	0	1	0	0	0	0	0	0	0	1
3751	UBXN1_HUMAN	UBX domain-containing protein 1	S199	62%	0.62	1	0	0	0	0	1	0	0	0	0	0	0	0	1
3752	UBXN1_HUMAN	UBX domain-containing protein 1	S202	43%	0.43	1	0	0	0	0	1	0	0	0	0	0	0	0	1
3753	UBXN1_HUMAN	UBX domain-containing protein 1	T207	96%	0.96	1	0	0	0	0	1	0	0	0	0	0	0	0	1
3754	UBX2B_HUMAN	UBX domain-containing protein 2B	S235	69%	0.69	14	0	0	0	1	0	0	0	0	0	0	0	0	1
3755	UBX2B_HUMAN	UBX domain-containing protein 2B	S242	67%	0.67	12	0	0	0	0	0	0	0	1	0	0	0	0	1
3756	CJ047_HUMAN	Uncharacterized protein C10orf47	S179	100%	1.00	3	1	2	0	0	0	0	0	0	0	0	0	0	3
3757	CJ047_HUMAN	Uncharacterized protein C10orf47	S212	100%	1.00	34	0	0	0	0	0	0	0	0	0	4	5	3	12

3758	CJ047_HUMAN	Uncharacterized protein C10orf47	S311	96%	0.96	23	2	1	0	1	0	1	0	1	0	0	0	0	6
3759	CJ047_HUMAN	Uncharacterized protein C10orf47	S312	97%	0.97	138	4	3	4	3	2	5	2	3	3	4	3	3	39
3760	CJ047_HUMAN	Uncharacterized protein C10orf47	S43	100%	1.00	4	0	0	0	2	0	0	0	0	0	0	0	0	2
3761	CK084_HUMAN	Uncharacterized protein C11orf84	S251	90%	0.90	1	1	0	0	0	0	0	0	0	0	0	0	0	1
3762	CK084_HUMAN	Uncharacterized protein C11orf84	S308	100%	1.00	24	0	6	5	0	2	0	0	2	0	0	0	0	15
3763	CL043_HUMAN	Uncharacterized protein C12orf43	S179	98%	0.98	17	2	0	0	0	1	0	0	0	0	0	0	0	3
3764	CL043_HUMAN	Uncharacterized protein C12orf43	T182	18%	0.18	8	0	0	1	0	0	0	0	0	0	0	0	0	1
3765	CL055_HUMAN	Uncharacterized protein C12orf55	S10	71%	0.71	1	0	1	0	0	0	0	0	0	0	0	0	0	1
3766	CL055_HUMAN	Uncharacterized protein C12orf55	S12	51%	0.51	1	0	1	0	0	0	0	0	0	0	0	0	0	1
3767	CL055_HUMAN	Uncharacterized protein C12orf55	S15	38%	0.38	1	0	1	0	0	0	0	0	0	0	0	0	0	1
3768	CL055_HUMAN	Uncharacterized protein C12orf55	S18	30%	0.30	1	0	1	0	0	0	0	0	0	0	0	0	0	1
3769	CL055_HUMAN	Uncharacterized protein C12orf55	S20	30%	0.30	1	0	1	0	0	0	0	0	0	0	0	0	0	1
3770	CL055_HUMAN	Uncharacterized protein C12orf55	T16	39%	0.39	1	0	1	0	0	0	0	0	0	0	0	0	0	1
3771	CL055_HUMAN	Uncharacterized protein C12orf55	T17	30%	0.30	1	0	1	0	0	0	0	0	0	0	0	0	0	1
3772	CL055_HUMAN	Uncharacterized protein C12orf55	T27	30%	0.30	1	0	1	0	0	0	0	0	0	0	0	0	0	1
3773	CO052_HUMAN	Uncharacterized protein C15orf52	S201	56%	0.56	2	0	0	0	0	0	0	0	0	0	1	0	0	1
3774	CO052_HUMAN	Uncharacterized protein C15orf52	S35	67%	0.67	1	0	0	0	1	0	0	0	0	0	0	0	0	1
3775	CO052_HUMAN	Uncharacterized protein C15orf52	S382	22%	0.22	4	0	0	0	0	0	0	0	0	0	0	1	0	1
3776	CO052_HUMAN	Uncharacterized protein C15orf52	S39	67%	0.67	1	0	0	0	1	0	0	0	0	0	0	0	0	1
3777	CO052_HUMAN	Uncharacterized protein C15orf52	S47	67%	0.67	1	0	0	0	1	0	0	0	0	0	0	0	0	1

3778	CO052_HUMAN	Uncharacterized protein C15orf52	S58	67%	0.67	1	0	0	0	1	0	0	0	0	0	0	0	0	1
3779	CO052_HUMAN	Uncharacterized protein C15orf52	T204	17%	0.17	1	0	0	0	0	0	0	0	0	0	1	0	0	1
3780	CP003_HUMAN	Uncharacterized protein C16orf3	S11	83%	0.83	1	0	0	0	0	0	0	0	0	0	0	0	1	1
3781	CP003_HUMAN	Uncharacterized protein C16orf3	S18	85%	0.85	1	0	0	0	0	0	0	0	0	0	0	0	1	1
3782	CP003_HUMAN	Uncharacterized protein C16orf3	S5	79%	0.79	1	0	0	0	0	0	0	0	0	0	0	0	1	1
3783	CP003_HUMAN	Uncharacterized protein C16orf3	T23	75%	0.75	1	0	0	0	0	0	0	0	0	0	0	0	1	1
3784	CQ085_HUMAN	Uncharacterized protein C17orf85	S25	100%	1.00	40	3	0	0	1	0	0	0	0	0	0	1	0	5
3785	CS021_HUMAN	Uncharacterized protein C19orf21	S394	100%	1.00	491	7	9	7	11	12	10	13	10	11	14	11	10	125
3786	CS021_HUMAN	Uncharacterized protein C19orf21	S395	100%	1.00	26	1	1	0	1	0	0	0	0	1	1	2	0	7
3787	CS021_HUMAN	Uncharacterized protein C19orf21	S397	90%	0.90	12	0	1	0	0	0	0	0	0	0	1	0	0	2
3788	CS043_HUMAN	Uncharacterized protein C19orf43	S39	99%	0.99	39	4	5	0	0	0	0	2	2	0	1	1	1	16
3789	CS043_HUMAN	Uncharacterized protein C19orf43	S45	14%	0.14	25	1	2	0	0	0	0	0	1	0	1	1	0	6
3790	CS043_HUMAN	Uncharacterized protein C19orf43	S49	53%	0.53	12	1	0	0	0	0	0	0	1	0	0	1	0	3
3791	CS043_HUMAN	Uncharacterized protein C19orf43	S50	69%	0.69	1	0	0	0	0	0	0	0	0	0	0	1	0	1
3792	CS047_HUMAN	Uncharacterized protein C19orf47	S151	20%	0.20	1	0	1	0	0	0	0	0	0	0	0	0	0	1
3793	CA170_HUMAN	Uncharacterized protein C1orf170	S65	67%	0.67	1	0	0	0	0	0	0	0	0	0	0	1	0	1
3794	CA170_HUMAN	Uncharacterized protein C1orf170	S87	21%	0.21	1	0	0	0	0	0	0	0	0	0	0	1	0	1
3795	CU070_HUMAN	Uncharacterized protein C21orf70	T34	100%	1.00	8	0	0	0	1	0	0	0	0	0	0	1	2	4
3796	CB055_HUMAN	Uncharacterized protein C2orf55	S92	99%	0.99	3	0	0	0	1	0	0	1	0	0	0	0	0	2
3797	CC024_HUMAN	Uncharacterized protein C3orf24	S28	91%	0.91	1	0	0	0	0	0	0	1	0	0	0	0	0	1

3798	CC024_HUMAN	Uncharacterized protein C3orf24	S29	90%	0.90	1	0	0	0	0	0	0	1	0	0	0	0	0	1
3799	CC024_HUMAN	Uncharacterized protein C3orf24	T23	95%	0.95	1	0	0	0	0	0	0	1	0	0	0	0	0	1
3800	CC024_HUMAN	Uncharacterized protein C3orf24	T24	94%	0.94	1	0	0	0	0	0	0	1	0	0	0	0	0	1
3801	CC024_HUMAN	Uncharacterized protein C3orf24	T26	92%	0.92	1	0	0	0	0	0	0	1	0	0	0	0	0	1
3802	CC024_HUMAN	Uncharacterized protein C3orf24	Y4	34%	0.34	1	0	0	0	0	0	0	1	0	0	0	0	0	1
3803	CC063_HUMAN	Uncharacterized protein C3orf63	S1193	30%	0.30	8	0	0	0	0	0	0	1	0	0	0	0	0	1
3804	CC063_HUMAN	Uncharacterized protein C3orf63	T1190	30%	0.30	2	1	0	0	0	0	0	0	0	0	0	0	0	1
3805	CE025_HUMAN	Uncharacterized protein C5orf25	T622	97%	0.97	2	0	0	0	1	0	0	0	0	0	0	0	0	1
3806	CF106_HUMAN	Uncharacterized protein C6orf106	S215	100%	1.00	12	0	0	0	1	0	1	0	0	0	1	0	1	4
3807	CG033_HUMAN	Uncharacterized protein C7orf33	S122	13%	0.13	1	0	0	0	0	0	0	0	0	0	0	1	0	1
3808	CG033_HUMAN	Uncharacterized protein C7orf33	Y133	22%	0.22	1	0	0	0	0	0	0	0	0	0	0	1	0	1
3809	CG033_HUMAN	Uncharacterized protein C7orf33	Y141	85%	0.85	1	0	0	0	0	0	0	0	0	0	0	1	0	1
3810	CG050_HUMAN	Uncharacterized protein C7orf50	S175	100%	1.00	6	0	0	0	0	0	0	0	0	2	0	1	2	5
3811	CI142_HUMAN	Uncharacterized protein C9orf142	S148	100%	1.00	3	0	0	0	0	0	0	0	0	0	0	0	2	2
3812	CI142_HUMAN	Uncharacterized protein C9orf142	S152	100%	1.00	4	0	0	0	1	0	0	0	0	0	0	3	0	4
3813	CI171_HUMAN	Uncharacterized protein C9orf171	T37	100%	1.00	1	0	0	0	0	0	0	0	0	0	0	1	0	1
3814	CI078_HUMAN	Uncharacterized protein C9orf78	S261	100%	1.00	1	0	0	0	0	0	0	0	0	1	0	0	0	1
3815	CX066_HUMAN	Uncharacterized protein CXorf66	S174	30%	0.30	1	0	0	0	0	0	0	0	0	0	0	0	1	1
3816	CX066_HUMAN	Uncharacterized protein CXorf66	S176	33%	0.33	1	0	0	0	0	0	0	0	0	0	0	0	1	1
3817	CX066_HUMAN	Uncharacterized protein CXorf66	S177	33%	0.33	1	0	0	0	0	0	0	0	0	0	1	0	0	1

3818	YJ005_HUMAN	Uncharacterized protein FLJ45252	S93	100%	1.00	13	3	0	0	1	1	1	0	0	0	1	1	0	8
3819	K0528_HUMAN	Uncharacterized protein KIAA0528	T818	75%	0.75	5	0	0	0	0	0	0	1	0	0	0	0	0	1
3820	K0754_HUMAN	Uncharacterized protein KIAA0754	S17	80%	0.80	1	0	0	0	0	0	1	0	0	0	0	0	0	1
3821	K0754_HUMAN	Uncharacterized protein KIAA0754	S20	80%	0.80	1	0	0	0	0	0	1	0	0	0	0	0	0	1
3822	K0754_HUMAN	Uncharacterized protein KIAA0754	S25	80%	0.80	1	0	0	0	0	0	1	0	0	0	0	0	0	1
3823	K0754_HUMAN	Uncharacterized protein KIAA0754	S28	80%	0.80	1	0	0	0	0	0	1	0	0	0	0	0	0	1
3824	K0930_HUMAN	Uncharacterized protein KIAA0930	T293	100%	1.00	3	0	0	0	0	0	0	0	0	0	0	0	1	1
3825	K1210_HUMAN	Uncharacterized protein KIAA1210	S272	83%	0.83	3	1	0	0	0	0	0	0	0	0	0	0	0	1
3826	K1210_HUMAN	Uncharacterized protein KIAA1210	S279	76%	0.76	3	1	0	0	0	0	0	0	0	0	0	0	0	1
3827	K1210_HUMAN	Uncharacterized protein KIAA1210	S283	85%	0.85	3	1	0	0	0	0	0	0	0	0	0	0	0	1
3828	K1210_HUMAN	Uncharacterized protein KIAA1210	S293	99%	0.99	3	1	0	0	0	0	0	0	0	0	0	0	0	1
3829	K1522_HUMAN	Uncharacterized protein KIAA1522	S545	100%	1.00	1	0	0	0	0	0	0	0	0	0	1	0	0	1
3830	K1522_HUMAN	Uncharacterized protein KIAA1522	S620	100%	1.00	1	0	0	0	0	0	0	0	0	0	1	0	0	1
3831	K1522_HUMAN	Uncharacterized protein KIAA1522	S669	100%	1.00	2	0	0	0	0	0	0	0	0	0	1	0	0	1
3832	K1522_HUMAN	Uncharacterized protein KIAA1522	S673	100%	1.00	2	0	0	0	0	0	0	0	0	0	1	0	0	1
3833	K1522_HUMAN	Uncharacterized protein KIAA1522	S862	100%	1.00	7	0	0	0	2	0	0	0	0	0	2	1	1	6
3834	K1522_HUMAN	Uncharacterized protein KIAA1522	S906	41%	0.41	3	0	0	0	1	0	0	0	0	0	0	0	0	1
3835	K1614_HUMAN	Uncharacterized protein KIAA1614	S1065	77%	0.77	1	0	0	0	0	0	0	0	0	0	0	0	1	1
3836	K1614_HUMAN	Uncharacterized protein KIAA1614	S1075	85%	0.85	1	0	0	0	0	0	0	0	0	0	0	0	1	1
3837	K1671_HUMAN	Uncharacterized protein KIAA1671	S1063	2%	0.02	2	1	1	0	0	0	0	0	0	0	0	0	0	2

3838	K1671_HUMAN	Uncharacterized protein KIAA1671	S465	91%	0.91	25	0	0	0	1	0	0	0	0	0	1	0	0	2
3839	K1671_HUMAN	Uncharacterized protein KIAA1671	S467	23%	0.23	7	0	0	0	1	0	0	0	0	0	0	0	0	1
3840	K1671_HUMAN	Uncharacterized protein KIAA1671	S508	89%	0.89	1	0	0	0	0	0	0	0	0	0	1	0	0	1
3841	K1671_HUMAN	Uncharacterized protein KIAA1671	T1059	99%	0.99	4	0	1	1	0	0	0	0	0	1	0	0	0	3
3842	K1704_HUMAN	Uncharacterized protein KIAA1704	S105	100%	1.00	108	4	1	2	2	1	0	2	2	2	2	2	0	20
3843	CA144_HUMAN	UPF0485 protein C1orf144	S107	100%	1.00	227	1	1	4	4	4	2	4	3	3	7	6	6	45
3844	K1430_HUMAN	UPF0501 protein KIAA1430	S15	47%	0.47	2	0	0	0	0	0	1	0	0	0	1	0	0	2
3845	K1430_HUMAN	UPF0501 protein KIAA1430	S19	100%	1.00	18	2	2	0	0	1	1	1	0	0	1	2	0	10
3846	K1430_HUMAN	UPF0501 protein KIAA1430	S218	96%	0.96	1	0	0	0	0	0	0	0	0	0	0	0	1	1
3847	U627A_HUMAN	UPF0627 protein ENSP00000364708	S92	98%	0.98	1	0	0	0	0	0	0	0	0	0	0	0	1	1
3848	CA055_HUMAN	UPF0667 protein C1orf55	S278	29%	0.29	6	0	1	1	1	1	1	0	0	0	0	0	0	5
3849	CG047_HUMAN	UPF0683 protein C7orf47	S109	50%	0.50	3	0	0	0	0	0	0	0	0	0	1	1	0	2
3850	CG047_HUMAN	UPF0683 protein C7orf47	S52	100%	1.00	15	1	0	0	1	0	0	0	0	1	1	0	0	4
3851	CA174_HUMAN	UPF0688 protein C1orf174	S213	56%	0.56	1	0	0	0	0	0	0	0	1	0	0	0	0	1
3852	CA174_HUMAN	UPF0688 protein C1orf174	S214	42%	0.42	1	0	0	0	0	0	0	0	1	0	0	0	0	1
3853	CA052_HUMAN	UPF0690 protein C1orf52	S158	91%	0.91	13	0	1	0	0	0	0	0	0	0	0	0	0	1
3854	CB029_HUMAN	UPF0760 protein C2orf29	S10	89%	0.89	4	0	0	0	0	1	0	0	1	2	0	0	0	4
3855	CB029_HUMAN	UPF0760 protein C2orf29	S7	98%	0.98	2	0	0	0	0	0	0	0	2	0	0	0	0	2
3856	UBIP1_HUMAN	Upstream-binding protein 1	T268	82%	0.82	1	0	0	0	0	1	0	0	0	0	0	0	0	1
3857	UBIP1_HUMAN	Upstream-binding protein 1	T269	68%	0.68	1	0	0	0	0	1	0	0	0	0	0	0	0	1

3858	UNG_HUMAN	Uracil-DNA glycosylase	S23	100%	1.00	83	1	3	2	3	3	3	1	2	3	0	0	0	21
3859	UNG_HUMAN	Uracil-DNA glycosylase	T31	11%	0.11	20	2	0	1	0	0	0	0	0	0	0	0	0	3
3860	RD23A_HUMAN	UV excision repair protein RAD23 homolog A	S205	98%	0.98	33	3	2	0	0	2	1	2	1	0	0	2	2	15
3861	RD23A_HUMAN	UV excision repair protein RAD23 homolog A	S212	28%	0.28	13	0	2	0	0	0	0	0	1	0	0	0	0	3
3862	RD23A_HUMAN	UV excision repair protein RAD23 homolog A	S216	93%	0.93	30	1	0	1	2	0	0	0	0	2	1	0	1	8
3863	RD23A_HUMAN	UV excision repair protein RAD23 homolog A	T200	8%	0.08	3	0	0	0	0	0	0	0	1	0	0	0	0	1
3864	RD23B_HUMAN	UV excision repair protein RAD23 homolog B	S160	100%	1.00	107	3	3	3	2	3	2	2	2	3	4	5	5	37
3865	RD23B_HUMAN	UV excision repair protein RAD23 homolog B	S166	9%	0.09	5	0	0	0	0	0	1	0	1	0	0	1	0	3
3866	RD23B_HUMAN	UV excision repair protein RAD23 homolog B	S168	8%	0.08	2	0	0	0	0	1	0	1	0	0	0	0	0	2
3867	RD23B_HUMAN	UV excision repair protein RAD23 homolog B	T155	28%	0.28	4	0	0	0	0	0	0	0	0	0	2	1	1	4
3868	RD23B_HUMAN	UV excision repair protein RAD23 homolog B	T159	44%	0.44	7	0	0	0	0	1	0	0	0	0	1	0	1	3
3869	RD23B_HUMAN	UV excision repair protein RAD23 homolog B	T162	14%	0.14	7	0	0	0	0	0	0	1	0	1	0	0	0	2
3870	RD23B_HUMAN	UV excision repair protein RAD23 homolog B	T164	35%	0.35	16	1	0	1	0	1	0	0	0	0	1	0	0	4
3871	RD23B_HUMAN	UV excision repair protein RAD23 homolog B	T167	17%	0.17	16	1	0	1	2	1	2	0	0	0	1	0	1	9
3872	RAG2_HUMAN	V(D)J recombination-activating protein 2	S2	98%	0.98	14	0	0	0	0	1	0	0	1	0	0	0	0	2
3873	RAG2_HUMAN	V(D)J recombination-activating protein 2	S20	100%	1.00	14	0	0	0	0	1	0	0	1	0	0	0	0	2
3874	VP13A_HUMAN	Vacuolar protein sorting-associated protein 13A	S2301	43%	0.43	1	0	0	0	0	0	0	1	0	0	0	0	0	1

3875	VP13A_HUMAN	Vacuolar protein sorting-associated protein 13A	S2302	18%	0.18	1	0	0	0	0	0	0	1	0	0	0	0	0	1
3876	VP13A_HUMAN	Vacuolar protein sorting-associated protein 13A	S833	63%	0.63	1	0	1	0	0	0	0	0	0	0	0	0	0	1
3877	VP13A_HUMAN	Vacuolar protein sorting-associated protein 13A	S835	77%	0.77	1	0	1	0	0	0	0	0	0	0	0	0	0	1
3878	VP13A_HUMAN	Vacuolar protein sorting-associated protein 13A	S839	14%	0.14	1	1	0	0	0	0	0	0	0	0	0	0	0	1
3879	VP13A_HUMAN	Vacuolar protein sorting-associated protein 13A	Y2315	18%	0.18	1	0	0	0	0	0	0	1	0	0	0	0	0	1
3880	VP13D_HUMAN	Vacuolar protein sorting-associated protein 13D	S926	100%	1.00	16	1	0	0	0	0	1	1	1	1	0	1	0	6
3881	VTA1_HUMAN	Vacuolar protein sorting-associated protein VTA1 homolog	S117	35%	0.35	1	0	0	0	0	0	0	0	0	0	0	1	0	1
3882	VCAM1_HUMAN	Vascular cell adhesion protein 1	S101	72%	0.72	1	0	0	0	0	0	0	0	0	0	0	1	0	1
3883	VCAM1_HUMAN	Vascular cell adhesion protein 1	S12	100%	1.00	2	0	0	0	0	0	0	0	0	0	1	0	1	2
3884	VCAM1_HUMAN	Vascular cell adhesion protein 1	S22	100%	1.00	2	0	0	0	0	0	0	0	0	0	1	0	1	2
3885	VCAM1_HUMAN	Vascular cell adhesion protein 1	S78	25%	0.25	1	0	0	0	0	0	0	0	0	0	0	1	0	1
3886	VCAM1_HUMAN	Vascular cell adhesion protein 1	T72	66%	0.66	1	0	0	0	0	0	0	0	0	0	0	1	0	1
3887	VCAM1_HUMAN	Vascular cell adhesion protein 1	T76	69%	0.69	1	0	0	0	0	0	0	0	0	0	0	1	0	1
3888	VCAM1_HUMAN	Vascular cell adhesion protein 1	T77	86%	0.86	1	0	0	0	0	0	0	0	0	0	0	1	0	1
3889	VCAM1_HUMAN	Vascular cell adhesion protein 1	T79	72%	0.72	1	0	0	0	0	0	0	0	0	0	0	1	0	1
3890	MELT_HUMAN	Ventricular zone-expressed PH domain-containing protein homolog 1	T533	12%	0.12	1	0	0	0	0	0	0	0	0	0	0	0	1	1
3891	CSPG2_HUMAN	Versican core protein	S1557	49%	0.49	1	0	0	0	0	0	1	0	0	0	0	0	0	1

3892	VAMP4_HUMAN	Vesicle-associated membrane protein 4	S30	100%	1.00	20	1	1	0	0	0	1	0	1	0	2	0	0	6
3893	VAPB_HUMAN	Vesicle-associated membrane protein-associated protein B/C	S100	81%	0.81	1	0	0	0	0	0	0	0	0	0	1	0	0	1
3894	VAPB_HUMAN	Vesicle-associated membrane protein-associated protein B/C	S92	47%	0.47	1	0	0	0	0	0	0	0	0	0	1	0	0	1
3895	VAPB_HUMAN	Vesicle-associated membrane protein-associated protein B/C	T99	84%	0.84	1	0	0	0	0	0	0	0	0	0	1	0	0	1
3896	SC22B_HUMAN	Vesicle-trafficking protein SEC22b	S137	100%	1.00	4	1	1	0	0	0	0	2	0	0	0	0	0	4
3897	VIGLN_HUMAN	Vigilin	S31	100%	1.00	7	2	1	0	0	2	0	1	0	0	0	0	0	6
3898	VIME_HUMAN	Vimentin	S409	51%	0.51	1	0	0	0	0	0	0	1	0	0	0	0	0	1
3899	VINC_HUMAN	Vinculin	S290	90%	0.90	3	1	2	0	0	0	0	0	0	0	0	0	0	3
3900	VINEX_HUMAN	Vinexin	S197	100%	1.00	1	0	0	0	0	0	0	0	0	0	0	1	0	1
3901	VINEX_HUMAN	Vinexin	S201	100%	1.00	1	0	0	0	0	0	0	0	0	0	0	1	0	1
3902	VINEX_HUMAN	Vinexin	S207	100%	1.00	1	0	0	0	0	0	0	0	0	0	0	1	0	1
3903	VINEX_HUMAN	Vinexin	S217	100%	1.00	1	0	0	0	0	0	0	0	0	0	0	1	0	1
3904	VINEX_HUMAN	Vinexin	S223	100%	1.00	1	0	0	0	0	0	0	0	0	0	0	1	0	1
3905	VINEX_HUMAN	Vinexin	S530	100%	1.00	118	3	3	3	2	3	2	3	2	2	3	3	6	35
3906	VINEX_HUMAN	Vinexin	S563	100%	1.00	39	0	0	0	0	0	0	1	0	1	0	0	0	2
3907	VINEX_HUMAN	Vinexin	T208	100%	1.00	1	0	0	0	0	0	0	0	0	0	0	1	0	1
3908	VINEX_HUMAN	Vinexin	T218	100%	1.00	1	0	0	0	0	0	0	0	0	0	0	1	0	1
3909	VINEX_HUMAN	Vinexin	Y211	100%	1.00	1	0	0	0	0	0	0	0	0	0	0	1	0	1
3910	PROZ_HUMAN	Vitamin K-dependent protein Z	S364	100%	1.00	1	0	0	0	0	1	0	0	0	0	0	0	0	1

3911	PROZ_HUMAN	Vitamin K-dependent protein Z	S373	100%	1.00	1	0	0	0	0	1	0	0	0	0	0	0	0	1
3912	PROZ_HUMAN	Vitamin K-dependent protein Z	T368	100%	1.00	1	0	0	0	0	1	0	0	0	0	0	0	0	1
3913	PROZ_HUMAN	Vitamin K-dependent protein Z	T386	100%	1.00	1	0	0	0	0	1	0	0	0	0	0	0	0	1
3914	VDAC1_HUMAN	Voltage-dependent anion-selective channel protein 1	S104	13%	0.13	5	0	0	0	0	0	0	0	0	0	0	2	0	2
3915	VDAC2_HUMAN	Voltage-dependent anion-selective channel protein 2	S115	20%	0.20	1	0	0	0	0	0	0	0	0	0	1	0	0	1
3916	VDAC3_HUMAN	Voltage-dependent anion-selective channel protein 3	S35	85%	0.85	1	0	0	0	0	0	0	1	0	0	0	0	0	1
3917	VDAC3_HUMAN	Voltage-dependent anion-selective channel protein 3	S37	86%	0.86	1	0	0	0	0	0	0	1	0	0	0	0	0	1
3918	VDAC3_HUMAN	Voltage-dependent anion-selective channel protein 3	S42	89%	0.89	1	0	0	0	0	0	0	1	0	0	0	0	0	1
3919	VDAC3_HUMAN	Voltage-dependent anion-selective channel protein 3	S44	90%	0.90	1	0	0	0	0	0	0	1	0	0	0	0	0	1
3920	VDAC3_HUMAN	Voltage-dependent anion-selective channel protein 3	S55	94%	0.94	1	0	0	0	0	0	0	1	0	0	0	0	0	1
3921	VDAC3_HUMAN	Voltage-dependent anion-selective channel protein 3	T43	90%	0.90	1	0	0	0	0	0	0	1	0	0	0	0	0	1
3922	VDAC3_HUMAN	Voltage-dependent anion-selective channel protein 3	T49	95%	0.95	1	0	0	0	0	0	0	1	0	0	0	0	0	1
3923	VDAC3_HUMAN	Voltage-dependent anion-selective channel protein 3	T51	97%	0.97	1	0	0	0	0	0	0	1	0	0	0	0	0	1
3924	VDAC3_HUMAN	Voltage-dependent anion-selective channel protein 3	T60	81%	0.81	1	0	0	0	0	0	0	1	0	0	0	0	0	1
3925	CAC1D_HUMAN	Voltage-dependent L-type calcium channel subunit alpha-1D	S480	20%	0.20	1	0	0	1	0	0	0	0	0	0	0	0	0	1
3926	CAC1D_HUMAN	Voltage-dependent L-type calcium channel subunit alpha-1D	T476	20%	0.20	1	0	0	1	0	0	0	0	0	0	0	0	0	1

3927	CAC1E_HUMAN	Voltage-dependent R-type calcium channel subunit alpha-1E	S792	15%	0.15	2	0	0	0	1	0	0	0	0	0	0	0	0	1
3928	CAC1I_HUMAN	Voltage-dependent T-type calcium channel subunit alpha-1I	S4	78%	0.78	1	0	0	0	0	0	0	0	0	0	0	0	1	1
3929	CAC1I_HUMAN	Voltage-dependent T-type calcium channel subunit alpha-1I	S6	78%	0.78	1	0	0	0	0	0	0	0	0	0	0	0	1	1
3930	CAC1I_HUMAN	Voltage-dependent T-type calcium channel subunit alpha-1I	T23	29%	0.29	1	0	0	0	0	0	0	0	0	0	0	0	1	1
3931	WFKN1_HUMAN	WAP, kazal, immunoglobulin, kunitz and NTR domain-containing protein 1	S244	54%	0.54	1	0	1	0	0	0	0	0	0	0	0	0	0	1
3932	WFKN1_HUMAN	WAP, kazal, immunoglobulin, kunitz and NTR domain-containing protein 1	T262	54%	0.54	1	0	1	0	0	0	0	0	0	0	0	0	0	1
3933	WFKN1_HUMAN	WAP, kazal, immunoglobulin, kunitz and NTR domain-containing protein 1	T264	54%	0.54	1	0	1	0	0	0	0	0	0	0	0	0	0	1
3934	WFKN1_HUMAN	WAP, kazal, immunoglobulin, kunitz and NTR domain-containing protein 1	Y261	54%	0.54	1	0	1	0	0	0	0	0	0	0	0	0	0	1
3935	WIPF3_HUMAN	WAS/WASL-interacting protein family member 3	S32	19%	0.19	1	0	0	0	0	1	0	0	0	0	0	0	0	1
3936	WIPF3_HUMAN	WAS/WASL-interacting protein family member 3	T33	19%	0.19	1	0	0	0	0	1	0	0	0	0	0	0	0	1
3937	FA21A_HUMAN	WASH complex subunit FAM21A	S158	100%	1.00	4	0	0	0	0	0	0	1	0	0	0	0	0	1
3938	FA21A_HUMAN	WASH complex subunit FAM21A	S160	100%	1.00	4	0	0	0	0	0	0	1	0	0	0	0	0	1
3939	FA21A_HUMAN	WASH complex subunit FAM21A	S284	100%	1.00	20	0	0	0	3	1	1	1	0	1	1	2	2	12
3940	FA21A_HUMAN	WASH complex subunit FAM21A	S333	100%	1.00	54	7	4	0	2	2	4	6	3	1	1	0	0	30
3941	FA21A_HUMAN	WASH complex subunit FAM21A	S352	100%	1.00	36	0	0	0	1	0	0	0	0	0	1	2	3	7

3942	FA21A_HUMAN	WASH complex subunit FAM21A	S539	100%	1.00	11	3	0	1	0	1	2	1	0	2	0	0	0	10
3943	FA21A_HUMAN	WASH complex subunit FAM21A	S619	100%	1.00	7	0	1	0	0	0	0	0	0	0	0	0	0	1
3944	FA21A_HUMAN	WASH complex subunit FAM21A	T331	0%	0.00	1	0	1	0	0	0	0	0	0	0	0	0	0	1
3945	WDTC1_HUMAN	WD and tetratricopeptide repeats protein 1	S511	100%	1.00	6	0	0	0	0	0	0	0	0	0	2	0	0	2
3946	WDFY3_HUMAN	WD repeat and FYVE domain-containing protein 3	S262	30%	0.30	15	0	1	1	0	0	0	0	0	0	0	0	0	2
3947	WDFY3_HUMAN	WD repeat and FYVE domain-containing protein 3	Y254	71%	0.71	12	0	0	0	1	0	1	0	0	0	0	0	1	3
3948	WDR17_HUMAN	WD repeat-containing protein 17	T619	29%	0.29	1	0	0	0	0	0	0	0	0	0	1	0	0	1
3949	WDR17_HUMAN	WD repeat-containing protein 17	T639	46%	0.46	1	0	0	0	0	0	0	0	0	0	1	0	0	1
3950	WDR17_HUMAN	WD repeat-containing protein 17	Y632	29%	0.29	1	0	0	0	0	0	0	0	0	0	1	0	0	1
3951	WDR3_HUMAN	WD repeat-containing protein 3	S241	85%	0.85	10	0	0	1	0	1	0	0	0	0	0	0	0	2
3952	WDR41_HUMAN	WD repeat-containing protein 41	S341	45%	0.45	1	0	0	1	0	0	0	0	0	0	0	0	0	1
3953	WDR44_HUMAN	WD repeat-containing protein 44	S262	100%	1.00	1	0	0	0	0	0	0	0	1	0	0	0	0	1
3954	WDR44_HUMAN	WD repeat-containing protein 44	T158	14%	0.14	2	1	0	0	0	0	0	0	0	0	1	0	0	2
3955	WDR44_HUMAN	WD repeat-containing protein 44	T160	29%	0.29	4	0	0	0	0	0	0	0	0	0	0	0	2	2
3956	WDR44_HUMAN	WD repeat-containing protein 44	T163	55%	0.55	14	0	0	0	1	0	0	0	0	0	2	2	0	5
3957	WDR52_HUMAN	WD repeat-containing protein 52	S427	93%	0.93	1	0	0	1	0	0	0	0	0	0	0	0	0	1
3958	WDR59_HUMAN	WD repeat-containing protein 59	S778	65%	0.65	1	0	0	1	0	0	0	0	0	0	0	0	0	1
3959	WDR59_HUMAN	WD repeat-containing protein 59	S780	65%	0.65	1	0	0	1	0	0	0	0	0	0	0	0	0	1
3960	WDR59_HUMAN	WD repeat-containing protein 59	S784	56%	0.56	1	0	0	1	0	0	0	0	0	0	0	0	0	1

3961	WDR59_HUMAN	WD repeat-containing protein 59	S787	65%	0.65	1	0	0	1	0	0	0	0	0	0	0	0	0	1
3962	WDR59_HUMAN	WD repeat-containing protein 59	S788	63%	0.63	1	0	0	1	0	0	0	0	0	0	0	0	0	1
3963	WDR59_HUMAN	WD repeat-containing protein 59	S790	56%	0.56	1	0	0	1	0	0	0	0	0	0	0	0	0	1
3964	WDR59_HUMAN	WD repeat-containing protein 59	S792	56%	0.56	1	0	0	1	0	0	0	0	0	0	0	0	0	1
3965	WDR59_HUMAN	WD repeat-containing protein 59	T786	57%	0.57	1	0	0	1	0	0	0	0	0	0	0	0	0	1
3966	WDR59_HUMAN	WD repeat-containing protein 59	Y782	57%	0.57	1	0	0	1	0	0	0	0	0	0	0	0	0	1
3967	WDR62_HUMAN	WD repeat-containing protein 62	S1226	25%	0.25	22	0	0	0	1	1	0	2	0	1	0	1	1	7
3968	WDR62_HUMAN	WD repeat-containing protein 62	S1228	25%	0.25	95	0	0	0	1	1	1	2	0	3	0	0	0	8
3969	WDR62_HUMAN	WD repeat-containing protein 62	S1232	25%	0.25	1	0	0	0	0	0	0	0	0	0	0	1	0	1
3970	WDR62_HUMAN	WD repeat-containing protein 62	S1248	78%	0.78	29	1	0	2	0	1	2	1	0	0	0	1	1	9
3971	WDR62_HUMAN	WD repeat-containing protein 62	S1249	88%	0.88	122	6	3	3	5	4	1	2	5	5	3	1	0	38
3972	WDR62_HUMAN	WD repeat-containing protein 62	S1255	60%	0.60	50	0	1	0	3	1	0	0	0	0	0	0	0	5
3973	WDR75_HUMAN	WD repeat-containing protein 75	S796	100%	1.00	75	0	0	2	1	1	0	0	0	0	1	0	0	5
3974	WT1_HUMAN	Wilms tumor protein	S208	39%	0.39	1	0	0	0	1	0	0	0	0	0	0	0	0	1
3975	WT1_HUMAN	Wilms tumor protein	T206	58%	0.58	1	0	0	0	1	0	0	0	0	0	0	0	0	1
3976	WT1_HUMAN	Wilms tumor protein	Y193	58%	0.58	1	0	0	0	1	0	0	0	0	0	0	0	0	1
3977	WAPL_HUMAN	Wings apart-like protein homolog	S221	100%	1.00	17	1	2	1	1	1	2	2	1	2	0	0	0	13
3978	WAPL_HUMAN	Wings apart-like protein homolog	S223	90%	0.90	14	0	1	2	1	2	1	2	2	1	0	0	2	14
3979	WAPL_HUMAN	Wings apart-like protein homolog	S77	100%	1.00	28	5	1	3	6	0	2	0	2	0	0	0	0	19

3980	WAC_HUMAN	WW domain-containing adapter protein with coiled-coil	S445	4%	0.04	1	0	1	0	0	0	0	0	0	0	0	0	0	1
3981	WAC_HUMAN	WW domain-containing adapter protein with coiled-coil	S446	89%	0.89	10	3	5	0	0	0	0	0	1	0	0	1	0	10
3982	XIRP1_HUMAN	Xin actin-binding repeat-containing protein 1	S1174	72%	0.72	1	0	0	0	0	0	0	0	0	0	1	0	0	1
3983	XIRP1_HUMAN	Xin actin-binding repeat-containing protein 1	S1188	99%	0.99	1	0	0	0	0	0	0	0	0	0	1	0	0	1
3984	XIRP1_HUMAN	Xin actin-binding repeat-containing protein 1	T1189	99%	0.99	1	0	0	0	0	0	0	0	0	0	1	0	0	1
3985	YETS2_HUMAN	YEATS domain-containing protein 2	S447	100%	1.00	1	0	0	0	0	0	0	0	0	0	0	0	1	1
3986	YJEN3_HUMAN	YjeF N-terminal domain-containing protein 3	S38	84%	0.84	1	0	0	0	0	0	0	0	1	0	0	0	0	1
3987	YJEN3_HUMAN	YjeF N-terminal domain-containing protein 3	S43	95%	0.95	1	0	0	0	0	0	0	0	1	0	0	0	0	1
3988	YJEN3_HUMAN	YjeF N-terminal domain-containing protein 3	T41	90%	0.90	1	0	0	0	0	0	0	0	1	0	0	0	0	1
3989	YJEN3_HUMAN	YjeF N-terminal domain-containing protein 3	T42	94%	0.94	1	0	0	0	0	0	0	0	1	0	0	0	0	1
3990	YAP1_HUMAN	Yorkie homolog	S109	97%	0.97	82	4	4	5	3	5	3	4	3	4	1	2	1	39
3991	YAP1_HUMAN	Yorkie homolog	S127	100%	1.00	149	7	7	3	4	1	3	1	5	4	5	2	1	43
3992	YAP1_HUMAN	Yorkie homolog	S128	38%	0.38	52	1	4	2	2	2	1	1	2	0	0	3	1	19
3993	YAP1_HUMAN	Yorkie homolog	S131	68%	0.68	77	2	4	4	3	0	4	1	3	3	1	0	1	26
3994	YAP1_HUMAN	Yorkie homolog	S138	90%	0.90	43	0	1	2	2	0	1	0	0	1	0	0	2	9
3995	YAP1_HUMAN	Yorkie homolog	S149	14%	0.14	7	0	0	0	1	0	0	0	0	0	0	0	0	1
3996	YAP1_HUMAN	Yorkie homolog	S61	100%	1.00	669	18	30	19	19	17	7	17	20	10	12	12	12	193
3997	YAP1_HUMAN	Yorkie homolog	T143	29%	0.29	2	0	0	0	0	0	0	0	0	0	0	1	0	1

3998	YAP1_HUMAN	Yorkie homolog	T145	97%	0.97	17	0	0	1	0	0	0	0	0	0	0	0	0	1
3999	YAP1_HUMAN	Yorkie homolog	T63	100%	1.00	495	16	10	12	15	19	16	12	13	17	10	9	8	157
4000	YRDC_HUMAN	YrdC domain-containing protein, mitochondrial	S37	100%	1.00	137	0	0	0	0	0	0	0	0	0	16	14	12	42
4001	YTDC1_HUMAN	YTH domain-containing protein 1	S308	100%	1.00	13	0	0	0	0	0	0	0	0	0	0	4	4	8
4002	ZBT7A_HUMAN	Zinc finger and BTB domain-containing protein 7A	S525	86%	0.86	24	1	1	1	1	0	0	1	0	0	0	1	1	7
4003	ZBT7A_HUMAN	Zinc finger and BTB domain-containing protein 7A	S526	83%	0.83	164	9	8	5	5	6	5	5	5	4	7	4	3	66
4004	ZBT7A_HUMAN	Zinc finger and BTB domain-containing protein 7A	S549	100%	1.00	52	6	6	2	4	5	2	3	0	0	1	3	0	32
4005	ZC11A_HUMAN	Zinc finger CCCH domain-containing protein 11A	S758	67%	0.67	85	1	2	3	1	2	2	3	2	2	4	3	1	26
4006	ZC11A_HUMAN	Zinc finger CCCH domain-containing protein 11A	S759	53%	0.53	10	1	0	0	0	0	0	0	1	0	0	0	0	2
4007	ZC11A_HUMAN	Zinc finger CCCH domain-containing protein 11A	S761	90%	0.90	14	0	0	0	1	0	0	0	0	1	0	0	1	3
4008	ZC11A_HUMAN	Zinc finger CCCH domain-containing protein 11A	S768	100%	1.00	4	0	0	0	0	0	0	0	0	0	1	0	0	1
4009	ZC11A_HUMAN	Zinc finger CCCH domain-containing protein 11A	T762	67%	0.67	17	0	0	0	0	1	0	1	0	0	0	1	1	4
4010	ZC3HE_HUMAN	Zinc finger CCCH domain-containing protein 14	S515	100%	1.00	1	0	0	1	0	0	0	0	0	0	0	0	0	1
4011	ZC3H4_HUMAN	Zinc finger CCCH domain-containing protein 4	S1110	10%	0.10	2	0	0	0	0	1	0	0	0	0	0	0	0	1
4012	ZC3H4_HUMAN	Zinc finger CCCH domain-containing protein 4	S1114	100%	1.00	76	4	6	5	1	1	3	4	4	1	0	2	0	31
4013	Z3H7B_HUMAN	Zinc finger CCCH domain-containing protein 7B	S233	99%	0.99	9	0	3	0	0	0	0	0	0	0	0	0	0	3
4014	ZCCHV_HUMAN	Zinc finger CCCH-type antiviral protein 1	S257	100%	1.00	9	0	0	0	4	2	1	0	0	0	1	0	0	8

4015	ZCCHV_HUMAN	Zinc finger CCCH-type antiviral protein 1	S284	100%	1.00	214	3	4	2	6	6	6	4	6	6	2	4	6	55
4016	ZCCHV_HUMAN	Zinc finger CCCH-type antiviral protein 1	S390	17%	0.17	1	0	0	0	0	0	0	0	0	0	0	1	0	1
4017	ZCRB1_HUMAN	Zinc finger CCHC-type and RNA-binding motif-containing protein 1	S155	100%	1.00	9	0	0	2	0	0	0	0	0	0	0	0	0	2
4018	ZFY19_HUMAN	Zinc finger FYVE domain-containing protein 19	S354	100%	1.00	12	2	0	0	0	0	0	4	2	2	2	0	0	12
4019	ZMYM2_HUMAN	Zinc finger MYM-type protein 2	T22	15%	0.15	1	0	0	0	1	0	0	0	0	0	0	0	0	1
4020	ZMYM2_HUMAN	Zinc finger MYM-type protein 2	T26	15%	0.15	1	0	0	0	1	0	0	0	0	0	0	0	0	1
4021	ZF106_HUMAN	Zinc finger protein 106 homolog	S1370	100%	1.00	43	1	1	1	2	0	1	0	1	2	0	0	0	9
4022	ZN142_HUMAN	Zinc finger protein 142	S800	100%	1.00	9	0	0	0	0	0	0	0	0	0	0	1	0	1
4023	ZN142_HUMAN	Zinc finger protein 142	T785	100%	1.00	9	0	0	0	0	0	0	0	0	0	0	1	0	1
4024	ZN142_HUMAN	Zinc finger protein 142	T786	100%	1.00	9	0	0	0	0	0	0	0	0	0	0	1	0	1
4025	ZN185_HUMAN	Zinc finger protein 185	S465	100%	1.00	49	0	0	1	0	6	0	0	1	1	9	6	4	28
4026	ZN185_HUMAN	Zinc finger protein 185	S468	100%	1.00	23	0	0	1	0	4	0	0	1	1	6	5	2	20
4027	ZN185_HUMAN	Zinc finger protein 185	S469	100%	1.00	23	0	0	1	0	4	0	0	1	1	6	5	2	20
4028	ZN185_HUMAN	Zinc finger protein 185	T447	100%	1.00	13	0	0	0	0	0	0	0	0	2	3	0	2	7
4029	ZN281_HUMAN	Zinc finger protein 281	S20	53%	0.53	3	0	0	0	0	0	0	0	0	1	0	0	0	1
4030	ZN281_HUMAN	Zinc finger protein 281	S22	47%	0.47	2	0	0	0	0	0	0	0	0	1	0	0	0	1
4031	ZN281_HUMAN	Zinc finger protein 281	S5	68%	0.68	3	0	0	0	0	0	0	0	0	0	0	0	1	1
4032	ZN281_HUMAN	Zinc finger protein 281	S9	17%	0.17	2	0	0	0	0	0	0	0	0	0	0	0	1	1
4033	ZN295_HUMAN	Zinc finger protein 295	S1003	78%	0.78	3	0	0	0	1	0	0	0	0	0	0	0	0	1

4034	ZN316_HUMAN	Zinc finger protein 316	S10	100%	1.00	43	2	0	1	4	2	0	0	0	2	1	0	0	12
4035	ZN316_HUMAN	Zinc finger protein 316	T7	1%	0.01	5	0	0	1	0	0	0	0	0	0	1	0	0	2
4036	ZN318_HUMAN	Zinc finger protein 318	S173	100%	1.00	36	4	1	2	3	3	4	1	0	1	1	0	0	20
4037	ZN318_HUMAN	Zinc finger protein 318	T2107	100%	1.00	7	0	0	1	0	0	1	0	0	1	0	0	1	4
4038	ZN318_HUMAN	Zinc finger protein 318	T2110	100%	1.00	7	0	0	1	0	0	1	0	0	1	0	0	1	4
4039	TISD_HUMAN	Zinc finger protein 36, C3H1 type-like 2	S490	99%	0.99	2	0	0	0	0	0	2	0	0	0	0	0	0	2
4040	ZN444_HUMAN	Zinc finger protein 444	S150	95%	0.95	2	0	0	1	0	0	0	0	0	0	0	0	0	1
4041	ZN444_HUMAN	Zinc finger protein 444	T124	51%	0.51	2	0	0	1	0	0	0	0	0	0	0	0	0	1
4042	ZN444_HUMAN	Zinc finger protein 444	Y156	81%	0.81	2	0	0	1	0	0	0	0	0	0	0	0	0	1
4043	ZNF45_HUMAN	Zinc finger protein 45	S174	25%	0.25	1	0	0	0	0	0	0	0	0	0	1	0	0	1
4044	ZN469_HUMAN	Zinc finger protein 469	S1993	80%	0.80	1	0	0	1	0	0	0	0	0	0	0	0	0	1
4045	ZN469_HUMAN	Zinc finger protein 469	T1964	58%	0.58	1	0	0	1	0	0	0	0	0	0	0	0	0	1
4046	ZN469_HUMAN	Zinc finger protein 469	T1969	58%	0.58	1	0	0	1	0	0	0	0	0	0	0	0	0	1
4047	ZN469_HUMAN	Zinc finger protein 469	T1984	58%	0.58	1	0	0	1	0	0	0	0	0	0	0	0	0	1
4048	ZN483_HUMAN	Zinc finger protein 483	S22	86%	0.86	1	0	0	0	0	0	0	0	0	1	0	0	0	1
4049	ZN483_HUMAN	Zinc finger protein 483	T19	61%	0.61	1	0	0	0	0	0	0	0	0	1	0	0	0	1
4050	Z512B_HUMAN	Zinc finger protein 512B	S297	92%	0.92	1	0	0	0	1	0	0	0	0	0	0	0	0	1
4051	Z512B_HUMAN	Zinc finger protein 512B	S309	88%	0.88	1	0	0	0	1	0	0	0	0	0	0	0	0	1
4052	Z512B_HUMAN	Zinc finger protein 512B	S315	93%	0.93	1	0	0	0	1	0	0	0	0	0	0	0	0	1
4053	Z512B_HUMAN	Zinc finger protein 512B	T283	100%	1.00	1	0	0	0	1	0	0	0	0	0	0	0	0	1

4054	Z512B_HUMAN	Zinc finger protein 512B	T285	100%	1.00	1	0	0	0	1	0	0	0	0	0	0	0	0	1
4055	Z512B_HUMAN	Zinc finger protein 512B	T291	98%	0.98	1	0	0	0	1	0	0	0	0	0	0	0	0	1
4056	Z512B_HUMAN	Zinc finger protein 512B	T295	97%	0.97	1	0	0	0	1	0	0	0	0	0	0	0	0	1
4057	ZN555_HUMAN	Zinc finger protein 555	S161	83%	0.83	1	0	0	0	1	0	0	0	0	0	0	0	0	1
4058	ZN555_HUMAN	Zinc finger protein 555	S182	84%	0.84	1	0	0	0	1	0	0	0	0	0	0	0	0	1
4059	ZN555_HUMAN	Zinc finger protein 555	T167	83%	0.83	1	0	0	0	1	0	0	0	0	0	0	0	0	1
4060	ZN555_HUMAN	Zinc finger protein 555	Y172	83%	0.83	1	0	0	0	1	0	0	0	0	0	0	0	0	1
4061	ZN555_HUMAN	Zinc finger protein 555	Y181	84%	0.84	1	0	0	0	1	0	0	0	0	0	0	0	0	1
4062	ZN567_HUMAN	Zinc finger protein 567	S429	88%	0.88	1	0	0	1	0	0	0	0	0	0	0	0	0	1
4063	ZN567_HUMAN	Zinc finger protein 567	S431	87%	0.87	1	0	0	1	0	0	0	0	0	0	0	0	0	1
4064	ZN567_HUMAN	Zinc finger protein 567	T414	74%	0.74	1	0	0	1	0	0	0	0	0	0	0	0	0	1
4065	ZN567_HUMAN	Zinc finger protein 567	T416	74%	0.74	1	0	0	1	0	0	0	0	0	0	0	0	0	1
4066	ZN579_HUMAN	Zinc finger protein 579	S483	100%	1.00	1	0	0	0	0	0	0	1	0	0	0	0	0	1
4067	ZN592_HUMAN	Zinc finger protein 592	S1264	64%	0.64	1	0	1	0	0	0	0	0	0	0	0	0	0	1
4068	ZN592_HUMAN	Zinc finger protein 592	T6	100%	1.00	27	0	0	0	0	0	0	0	0	0	0	1	0	1
4069	ZN593_HUMAN	Zinc finger protein 593	T134	17%	0.17	1	0	0	1	0	0	0	0	0	0	0	0	0	1
4070	ZN609_HUMAN	Zinc finger protein 609	S252	94%	0.94	10	0	0	0	0	0	0	0	0	0	2	1	0	3
4071	ZN615_HUMAN	Zinc finger protein 615	S75	99%	0.99	1	0	0	0	0	0	0	0	0	1	0	0	0	1
4072	ZN615_HUMAN	Zinc finger protein 615	T68	90%	0.90	1	0	0	0	0	0	0	0	0	1	0	0	0	1
4073	ZN615_HUMAN	Zinc finger protein 615	T69	97%	0.97	1	0	0	0	0	0	0	0	0	1	0	0	0	1

4074	ZN615_HUMAN	Zinc finger protein 615	Y74	98%	0.98	1	0	0	0	0	0	0	0	0	1	0	0	0	1
4075	ZN687_HUMAN	Zinc finger protein 687	S180	20%	0.20	1	0	0	0	0	0	0	1	0	0	0	0	0	1
4076	ZN687_HUMAN	Zinc finger protein 687	S183	69%	0.69	3	0	0	0	0	0	0	0	0	1	0	2	0	3
4077	ZN687_HUMAN	Zinc finger protein 687	S251	100%	1.00	13	0	0	0	0	0	1	0	1	0	0	0	1	3
4078	ZN687_HUMAN	Zinc finger protein 687	S253	100%	1.00	232	1	1	2	3	3	3	6	2	6	6	5	7	45
4079	ZN687_HUMAN	Zinc finger protein 687	T185	58%	0.58	3	1	0	0	0	0	0	1	0	0	1	0	0	3
4080	ZN691_HUMAN	Zinc finger protein 691	S2	52%	0.52	1	0	0	1	0	0	0	0	0	0	0	0	0	1
4081	ZN691_HUMAN	Zinc finger protein 691	S27	91%	0.91	1	0	0	1	0	0	0	0	0	0	0	0	0	1
4082	ZN691_HUMAN	Zinc finger protein 691	S30	88%	0.88	1	0	0	1	0	0	0	0	0	0	0	0	0	1
4083	ZN691_HUMAN	Zinc finger protein 691	S34	74%	0.74	1	0	0	1	0	0	0	0	0	0	0	0	0	1
4084	ZN691_HUMAN	Zinc finger protein 691	S5	52%	0.52	1	0	0	1	0	0	0	0	0	0	0	0	0	1
4085	ZN691_HUMAN	Zinc finger protein 691	T7	52%	0.52	1	0	0	1	0	0	0	0	0	0	0	0	0	1
4086	ZN697_HUMAN	Zinc finger protein 697	T404	96%	0.96	1	0	0	0	0	0	0	0	0	0	0	1	0	1
4087	ZN746_HUMAN	Zinc finger protein 746	S474	80%	0.80	1	0	0	0	0	0	0	0	0	0	0	0	1	1
4088	ZN746_HUMAN	Zinc finger protein 746	S481	83%	0.83	1	0	0	0	0	0	0	0	0	0	0	0	1	1
4089	ZN746_HUMAN	Zinc finger protein 746	S490	71%	0.71	1	0	0	0	0	0	0	0	0	0	0	0	1	1
4090	ZN746_HUMAN	Zinc finger protein 746	S492	76%	0.76	1	0	0	0	0	0	0	0	0	0	0	0	1	1
4091	ZN746_HUMAN	Zinc finger protein 746	S508	75%	0.75	1	0	0	0	0	0	0	0	0	0	0	0	1	1
4092	ZN746_HUMAN	Zinc finger protein 746	T485	80%	0.80	1	0	0	0	0	0	0	0	0	0	0	0	1	1
4093	ZN793_HUMAN	Zinc finger protein 793	S212	35%	0.35	1	0	0	0	0	1	0	0	0	0	0	0	0	1

4094	ZN793_HUMAN	Zinc finger protein 793	T198	35%	0.35	1	0	0	0	0	1	0	0	0	0	0	0	0	1
4095	ZN793_HUMAN	Zinc finger protein 793	Y215	96%	0.96	1	0	0	0	0	1	0	0	0	0	0	0	0	1
4096	Z804B_HUMAN	Zinc finger protein 804B	S877	100%	1.00	2	0	0	0	0	1	0	0	1	0	0	0	0	2
4097	AEBP2_HUMAN	Zinc finger protein AEBP2	S18	100%	1.00	541	20	13	15	21	18	11	14	16	7	10	10	7	162
4098	AEBP2_HUMAN	Zinc finger protein AEBP2	S24	99%	0.99	507	17	13	15	16	17	11	14	15	7	7	9	6	147
4099	AEBP2_HUMAN	Zinc finger protein AEBP2	S27	56%	0.56	27	1	0	0	2	1	0	0	0	0	3	1	1	9
4100	DZIP1_HUMAN	Zinc finger protein DZIP1	S134	43%	0.43	1	0	0	0	0	0	0	1	0	0	0	0	0	1
4101	DZIP1_HUMAN	Zinc finger protein DZIP1	S140	73%	0.73	1	0	0	0	0	0	0	1	0	0	0	0	0	1
4102	DZIP1_HUMAN	Zinc finger protein DZIP1	T127	81%	0.81	1	0	0	0	0	0	0	1	0	0	0	0	0	1
4103	DZIP1_HUMAN	Zinc finger protein DZIP1	Y130	83%	0.83	1	0	0	0	0	0	0	1	0	0	0	0	0	1
4104	REQU_HUMAN	Zinc finger protein ubi-d4	S142	100%	1.00	19	0	3	1	0	0	0	0	0	0	1	2	0	7
4105	RNZ2_HUMAN	Zinc phosphodiesterase ELAC protein 2	S208	100%	1.00	12	0	0	1	0	0	0	0	1	0	0	0	2	4
4106	RNZ2_HUMAN	Zinc phosphodiesterase ELAC protein 2	S212	24%	0.24	5	0	0	1	0	0	0	0	1	0	0	0	1	3
4107	RNZ2_HUMAN	Zinc phosphodiesterase ELAC protein 2	S213	97%	0.97	9	0	0	0	0	0	0	0	0	0	0	0	1	1
4108	ZACN_HUMAN	Zinc-activated ligand-gated ion channel	S358	100%	1.00	1	0	0	0	0	0	0	0	0	0	0	0	1	1
4109	ZYX_HUMAN	Zyxin	S142	95%	0.95	38	0	0	0	0	3	0	0	0	0	1	0	1	5
4110	ZYX_HUMAN	Zyxin	S143	67%	0.67	104	3	1	0	1	4	4	2	1	2	2	5	2	27
4111	ZYX_HUMAN	Zyxin	S308	93%	0.93	45	2	1	0	0	0	0	1	0	0	5	5	3	17
4112	ZYX_HUMAN	Zyxin	S344	100%	1.00	169	1	5	4	7	5	4	4	4	3	6	7	7	57

4113	ZYX_HUMAN	Zyxin	T306	60%	0.60	17	0	0	1	1	1	0	0	0	0	1	1	1	6

Table S3. Protein names for nodes shown in Figure 1, listed by cluster.

Cluster Number	Number of Proteins in Cluster	Proteins in Cluster (listed as gene names)
1	54	ABLIM3,ANXA1,ANXA2,ARHGAP32,ARHGEF1,BIN1,CD44,COL17A1,CTNNA1,CTNNB1,CTTN,EGFR,EPHA2, EPN1,EPS15,EPS8L2,FCHO2,FLII,FLNA,HOMER3,ITGB4,KANK1,KLC3,LIMA1,LRRFIP1,MAP3K2,MAP4K4, NCK1,PAK2,PAK4,PDPK1,PDPK2P,PGRMC1,PI4KB,PIK3R2,PLEC,PRKCI,PTK2,PTPN12,PTPN13,PXN,RTN4, SH3KBP1,SLC9A1,SPAG9,SPTAN1,SRC,TJP1,TJP2,TJP3,TLN1,TRIO,VCL,ZYX
2	45	ACSS2,AKT1S1,BAD,BSG,CDC37,CRTC2,CTBP2,DUSP7,EIF4EBP1,HCFC1,HDAC2,HSF1,HSP90AB2P,IL1A,IRS1, JUND,KRT6A,KRT8,MAPK1,MAPK3,MCRS1,MEF2D,NCOR2,PEA15,PNN,PPP1R1B,PPP2R5D,PRKAR1A,RIN1, RPTOR,SCRIB,SIRT1,SMARCA4,SPEN,SQSTM1,STMN1,TAF12,TFE3,TFEB,TOP2A,UBR5,YAP1,YWHAZ, ZBTB7A,ZNF318
3	28	DDX21,DHX9,DIDO1,EIF3B,ELAVL1,GAPDH,HNRNPC,HNRNPD,HNRNPF,HNRNPH1,HNRNPH2,HNRNPK, HNRNPU,HNRNPUL1,IGF2BP2,ILF3,KHSRP,PCBP1,RBM10,RBM25,SF3B2,SRRM1,SRRM2,SRSF11,SRSF2, SRSF9,THRAP3,YBX1
4	28	AEBP2,ARGLU1,ATRX,BCL7C,BCLAF1,CD3EAP,CSRP1,DAXX,DPF2,DSG2,DSP,EZH2,FAF1,GTF3C1,IFI16, IRF2BP2,KRT14,KRT15,KRT17,KRT5,KRT7,MVP,PKP2,PKP3,PRMT1,SP100,STAT3,TNIP1
5	25	AAAS,CHD3,CLASP2,HJURP,MEPCE,NCL,NPM1,NUMA1,NUP155,NUP214,NUP98,PDS5B,RANBP2,RANGAP1, RCC1,RNF4,RSF1,SMN1,SUMO3,TP53BP1,TPR,TRIM28,TRIP12,WAPAL,WDR77
6	18	BYSL,CNOT3,DCP1A,DDX24,EDC4,EIF2S2,EIF4G1,EIF4G2,EIF5B,NOP2,NOP56,NOP58,RPL14,RPLP2,RPS3, SSR1,TNKS1BP1,ZC3H7B
7	11	ANAPC4,BAG3,HMGCS1,PSMA5,PSMD2,PSME3,PSMF1,PTTG1,PTTG2,RFFL,USP7
8	9	ARHGEF2,DYNC1LI1,DYNC1LI2,KLC2,PPP1R2,PPP1R9B,SPTBN2,TUBA1B,TUBA1C
9	4	EEF1B2,EEF1D,SAFB,SUPT6H
10	3	H1FX,HIST1H1B,HIST1H1C
